# 3D revelation of phenotypic variation, evolutionary allometry, and ancestral states of corolla shape: a case study of clade Corytholoma (subtribe Ligeriinae, family Gesneriaceae)

**DOI:** 10.1093/gigascience/giz155

**Published:** 2020-01-22

**Authors:** Hao-Chun Hsu, Wen-Chieh Chou, Yan-Fu Kuo

**Affiliations:** Department of Biomechatronics Engineering, National Taiwan University, No. 1, Sec. 4, Roosevelt Road, Taipei 106, Taiwan

**Keywords:** Corytholoma, corolla shape variations, evolutionary allometry, geometric morphometrics (GM), generalized Procrustes analysis (GPA), Ligeriinae, X-ray micro-computed tomography (µCT)

## Abstract

**Background:**

Quantification of corolla shape variations helps biologists to investigate plant diversity and evolution. 3D images capture the genuine structure and provide comprehensive spatial information.

**Results:**

This study applied X-ray micro-computed tomography (µCT) to acquire 3D structures of the corollas of clade Corytholoma and extracted a set of 415 3D landmarks from each specimen. By applying the geometric morphometrics (GM) to the landmarks, the first 4 principal components (PCs) in the 3D shape and 3D form analyses, respectively, accounted for 87.86% and 96.34% of the total variance. The centroid sizes of the corollas only accounted for 5.46% of the corolla shape variation, suggesting that the evolutionary allometry was weak. The 4 morphological traits corresponding to the 4 shape PCs were defined as tube curvature, lobe area, tube dilation, and lobe recurvation. Tube curvature and tube dilation were strongly associated with the pollination type and contained phylogenetic signals in clade Corytholoma. The landmarks were further used to reconstruct corolla shapes at the ancestral states.

**Conclusions:**

With the integration of µCT imaging into GM, the proposed approach boosted the precision in quantifying corolla traits and improved the understanding of the morphological traits corresponding to the pollination type, impact of size on shape variation, and evolution of corolla shape in clade Corytholoma.

## Background

**Figure 1:**
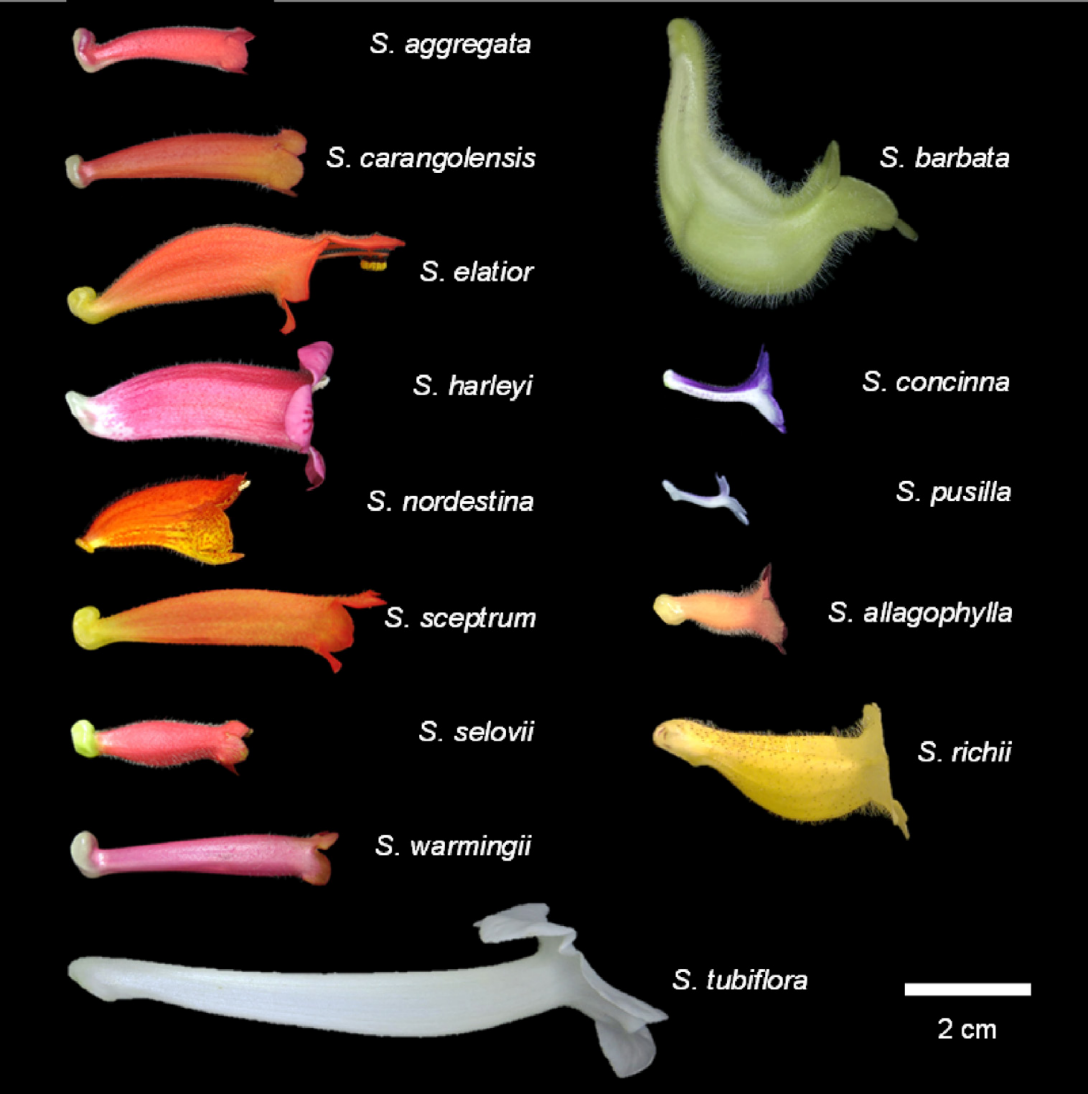
Side view of the corolla of the species in clade Corytholoma.

The variation in corolla shapes and forms (i.e., shape and size together [1]) in angiosperms has received considerable research attention [2, 3]. It was believed that this variation could be principally attributed to the specialization in animal-mediated pollination. Particularly, the species in clade Corytholoma of subtribe Ligeriinae (family Gesneriaceae) yield flowers with assorted shapes (tubular, funnel, and bell-shaped; Fig. 1) and various sizes (1–9 cm in length) and are associated with different pollinators [4]. Because of the rapid change in optimized corolla morphologies in a monophyletic group, the corollas serve as excellent materials for studying pollinator association and identifying the shape transition of the corollas. Corollas are complex 3D objects, an approach should be developed for appropriately assessing their shape and size. This study applied X-ray micro-computed tomography (µCT) and 3D geometric morphometrics (GM) [5, 6] for identifying the major shape and form variations of the corollas, revealing the association between the corolla shape and pollination type, and elucidating the evolution of corolla shape in clade Corytholoma.

In the past decade, landmark-based GM has been frequently applied to quantify shape and form variations of corollas (Table S1). Through landmark-based GM, the spatial variations of corolla landmarks (i.e., characteristic points of interest) can be extracted using generalized Procrustes analysis (GPA) [7, 8] and the major variations between the landmarks can be summarized using dimensionality reduction techniques (e.g., principal component analysis [PCA] or linear discriminant analysis [LDA]). Landmark-based GM can be 2D or 3D. In studies using 2D GM, the identification of major shape and form variations was restricted by the imaging views (e.g., side, face, or dissected) of the corollas. However, corollas are objects with a complex 3D geometry. None of the 3 views provides complete information of the corolla structures [9]. This shortcoming can be overcome by combining 3D imaging techniques with GM [10]. In studies using 3D GM [9, 11, 12], the structural information of the whole corollas was comprehensively captured and retained.

The 3D corolla information can promote specificity for studying the allometry of corolla shape. Allometry refers to the change in target traits in response to change in size [13]. Conventionally, allometry studies are limited to investigating the relationship between 2 distance-based measurements, such as length and width, of objects [14]. Since the emergence of GM techniques, some studies have investigated the allometry of corollas in the geometry aspect using 2D images [15, 16]. However, these studies still faced the aforementioned shortcoming that 2D images inadequately capture the structural information of 3D objects. Additionally, 2D images are usually acquired manually, which may introduce error or artifacts due to inconsistent adjustments during the operation. Therefore, the allometry of corolla shape can be assessed more accurately and comprehensively by using the precision and integrity of 3D imaging.

The major shape variations of the corollas identified through GM can also help in examining the association between corolla shape and pollinators. Corolla shape is one of the most prominent indicators associated with pollinator type [17, 18]. Conventionally, distance-based traits (e.g., diameter of corolla orifices and length of corolla tube) were used to evaluate pollination association [19–21]. However, distance-based traits are typically proposed on the basis of manual observation and can be subjective. Additionally, these traits could be oversimplified and may not adequately describe the geometric properties of the corollas [1, 22, 23]. By contrast, the shape variations obtained using GM were identified through a series of statistical procedures; thus, they could adequately represent the principal shape differences among the corollas. Gómez et al. [24] and Kaczorowski et al. [25] used the corolla shape variations quantified using 2D GM to examine the association between plant species and pollinators in *Erysimum* and *Nicotiana*, respectively. Traits identified using 3D GM precisely describe the leading variations in the geometric properties of corollas; thus, they can serve as excellent candidates in the tests of pollinator association.

Corolla shapes at the ancestral states is another intriguing research topic for biologists. To infer history and interpret the evolution of species, the characteristics of the species and their transitions along phylogeny are reconstructed and evaluated [26, 27]. The corolla shapes at the ancestral states can be reconstructed using a given phylogeny and corolla landmarks of the extant species [28]. Gómez et al. [29] reconstructed the corolla shapes in *Erysimum* and visualized the changes in shape at the ancestral states using GM and 2D landmarks in the face view. Joly et al. [30] identified the evolutionary constraints on corolla shape in Gesneriaceae using GM and 2D landmarks in the side view. The corollas reconstructed using face or side views only provide part of the structural information of the corollas. By contrast, the corollas reconstructed using 3D landmarks show complete structural information. Thus, 3D images may reveal more information regarding the transition of the corolla shapes at the ancestral states.

This study scrutinized the 3D corolla shapes and forms of the species in clade Corytholoma. We used the 3D approach to acquire the images of the corollas; thus, the complete structural information of the corollas was retained. We selected 415 landmarks for each corolla; thus, the structures of the corollas were genuinely represented. We performed GM analyses on the landmarks to identify both the 3D shape and 3D form variations of the corollas; thus, the impact of corolla size on corolla shape could also be examined. We defined morphological traits of the corollas on the basis of the GM results and quantified the traits directly using the 3D corolla images; thus, the traits were proposed statistically rather than manually. The proposed traits were subsequently used for investigating the association between pollination type and corolla shapes; thus, the leading shape variations could be used and interpreted in the association tests. We further evaluated the phylogenetic signals of corolla size and morphological traits; thus, the tempo and mode of corolla evolution could be assessed. Last, we reconstructed corolla shapes at the ancestral states using 3D landmarks; thus, more information regarding the shape transition of the corolla could be revealed.

## Data Description

### Flower materials

The germplasms of 15 species (Table 1) in clade Corytholoma were obtained from Dr. Cecilia Koo Botanic Conservation Center (KBCC), Pingtung, Taiwan, and were maintained by establishing inbred lines. The plant individuals were cultivated under natural lighting, 70–80% humidity, and at 22–28°C in a greenhouse (Technology Commons X, College of Life Science, National Taiwan University, Taiwan). For each species, 6–16 flowers were collected from 2–5 plant individuals between August 2015 and August 2016, resulting in a total of 153 specimens (Table S2). The specimens of the same species were collected in the same flowering season to alleviate the shape variations caused by different flowering seasons. The Corytholoma species are protandrous, which means the anther matures before the stigma. To minimize the developmental variations, the collection was conducted at the developmental stage between anther and stigma anthesis. The specimens were prepared fresh or were fixed in 70% ethanol solution (Table 1).

**Table 1:** Species list and dimension of the slice images

Species	Pollination type^a^	Specimen type^b^	KBCC and inbred line accession
*Sinningia aggregata*	H	F	K039091, K039092, K039093
*Sinningia allagophylla*	H	F	K039099, L039110, HC0909-d
*Sinningia barbata*	B	E/F	K039104, K039105, HC1206-a, HC1206-d
*Sinningia carangolensis*	H	F	K039112, HC1912-2, HC1912-b
*Sinningia concinna*	B	F	K039117, K039118, HC2202-t
*Sinningia elatior*	H	E	K039126, K039127, K039129, K039131
*Sinningia harleyi*	H	F	K039135, HC3403-3, HC3403-8
*Sinningia nordestina*	H	F	K039168, HC5504-1, HC5504-3
*Sinningia pusilla*	B	F	K039169, K039170, K039171, K039172, HC5803-2
*Sinningia richii*	B	E/F	K039174, K039175, K039176, K039177
*Sinningia sceptrum*	H	E	K039178, K039179, K039181
*Sinningia sellovii*	H	E	K039184, K039185, K039186
*Sinningia tubiflora*	M	F	K039197, K039198, K039199, K039200, K039201
*Sinningia valsuganensis*	H	F	K039203, K039204
*Sinningia warmingii*	H	E	K039205, K039209, K039216

a B: bee pollination (melittophily); H: hummingbird pollination (ornithophily); M: moth pollination (phalaenophily).

b E: 70% ethanol–fixed specimen; F: fresh specimen.

All 153 specimens were used in the analyses of 3D shape and form variations and of evolutionary allometry. To avoid being dominated by the species with larger sample size, 5 specimens with shape scores near the median of each species were selected. These specimens (a total of 75) were used in the analyses of phylogenetic signals and of ancestral state reconstruction (see Methods for the calculation of shape score and see Table S2 for the specimen information).

The information on pollination types was obtained from Perret et al. [4]. The species were associated with 3 pollination types: hummingbird, bee, and moth (Table 1). The hummingbird-pollinated species have tubular corollas (Fig. 1). The bee-pollinated species have campanulate or salverform corollas. The moth-pollinated species have narrow and long tubular corollas.

### 3D flower image data

**Figure 2:**
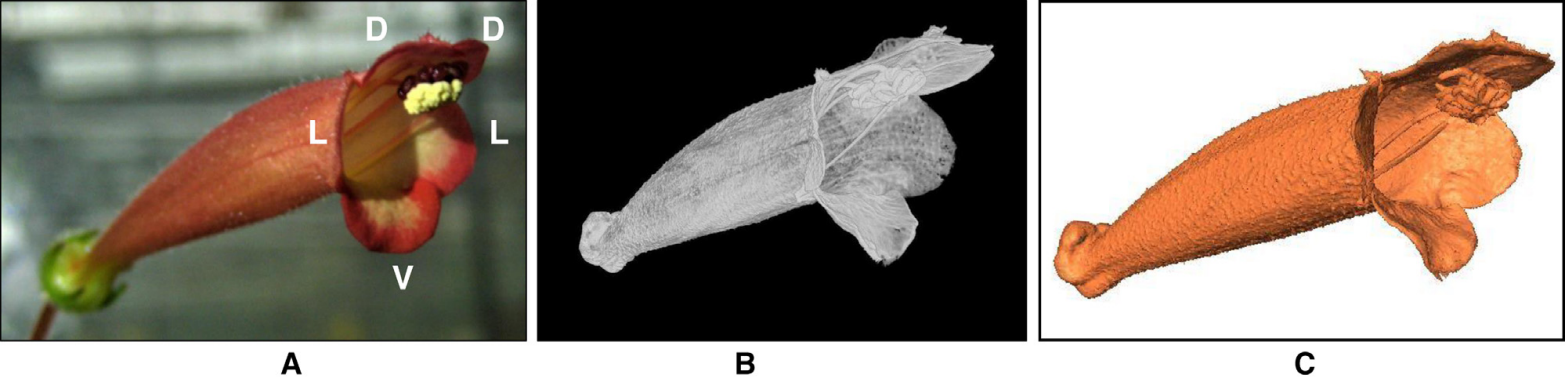
(A) Photograph, (B) volumetric image, and (C) surface image of a corolla of *S. sceptrum*. D denotes dorsal position, L denotes lateral position, and V denotes ventral position.

The 3D images of the flowers were acquired using an X-ray µCT scanner (SkyScan 1076, Bruker; Kontich, Belgium). The spatial resolution of the scanner was 36.547 μm in each dimension (Table S2). A 3D image was composed of hundreds or thousands of 2D slice images along the longitudinal axis. In each 2D slice image, image thresholding, morphological operation, and connected component labeling were performed to reduce the noise of the images and to separate the region of corolla from the background [9]. The processed 2D slice images of the same specimen were then integrated into a 3D volumetric image [9] (Fig. 2B). The volumetric images were next converted into surface images [9] (Fig. 2C), in which the surfaces of the corollas were covered by triangular meshes. The surface images were saved in polygon (PLY) file format for the subsequent landmark identification [9]. The 2D slice images, volumetric images, and the surface images of the specimens are available in the GigaScience database repository. The demonstration video of the program for generating a 3D volumetric image from 2D slice images can be found in Wang et al. [9].

### Landmark identification

**Figure 3:**
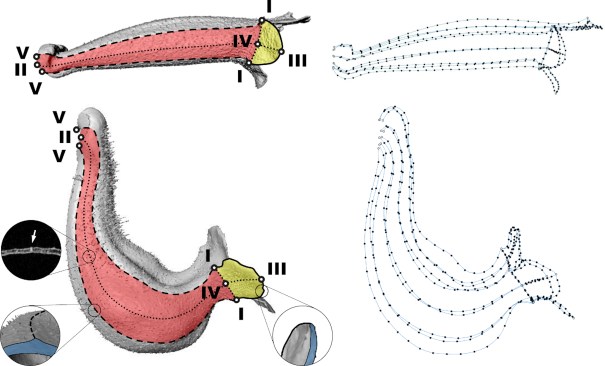
Landmarks of *S. sceptrum* (top panel) and *S. barbata* (bottom panel). A total of 415 landmarks, including 25 primary (Roman numerals) and 390 secondary, were identified on each corolla on the basis of the homologous features of the petals. White points with Roman numerals: I: the intersections of adjacent lobes (share with connected petals); II and III: the proximal and distal points of petal midribs; IV: the intersection of the lobe-tube rims and midribs; V: the proximal points of the tube-tube rims (share with connected petals). Solid line: lobe contour; round dotted line: petal midrib contour; square dotted line (at the junction of yellow and red colors): contours of lobe-tube connected rims; dashed line: contour of tube-tube connected rim (share with connected petals); Yellow indicates lobe region; red indicates tube region. White arrow indicates the location of the midrib in the 2D slice image.

Landmarks were defined on the basis of the homologous and anatomically recognizable features of the corollas. The corollas of the Corytholoma species consist of 2 dorsal, 2 lateral, and 1 ventral petals (Fig. 2A). Each petal has a lobe region (hanging part) and a tube region (part connecting to other petals). The petal also has a 3-nerved venation extending from the proximal end of the tube region to the distal end of the lobe region. The homologous features of a petal (Fig. 3) include the intersections of adjacent lobes, the lobe contour, the petal midrib (main vein), the lobe-tube connected rims, and the tube–tube connected rim. Twenty-five primary landmarks (Roman numerals in Fig. 3) were defined on the basis of the features, including 5 intersections of adjacent lobes (I), 5 proximal points of petal midribs (II), 5 distal points of petal midribs (III), 5 intersections of the lobe-tube rims and petal midribs (IV), and 5 proximal points of the tube-tube rims (V). A total of 390 secondary landmarks were defined as 15 equally distributed points on each lobe contour, 7 equally distributed points on each lobe-lobe rim, 7 equally distributed points on each lobe midrib, 25 equally distributed points on each tube midrib, and 25 equally distributed points on each tube-tube rim.

The landmarks were selected semi-automatically from the 3D surface images. The selection of the intersections of adjacent lobes, the contours of lobe-tube connected rims, and the contours of tube-tube connected rims was performed manually using Landmark software [9, 12, 31]. The contours and midribs were pre-identified automatically using software developed by the authors’ team [32]. The secondary landmarks were then automatically determined on the basis of the selected contours, midribs, and rims using a program developed in MATLAB (MathWorks, Natick, MA, USA) [9]. The landmarks of the specimens in this study are available in the GigaScience database repository.

## Analyses

### Corolla centroid size

**Figure 4:**
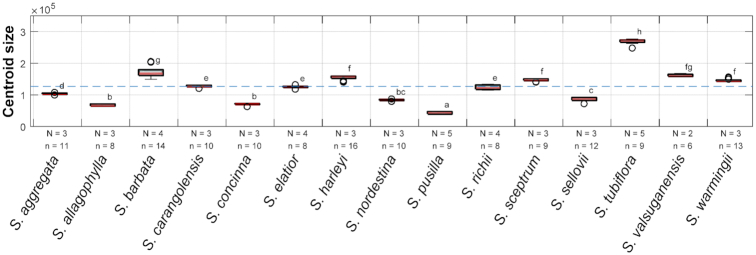
Centroid sizes of the corollas of the extant species. The blue dashed line denotes the average centroid size. The lowercase letters at the right of the box plots denote groups of Scheffé’s multiple comparison tests performed with a confidence level of 0.99. N: number of plant individuals; n: number of specimens. The results of the ANOVA assumption tests and ANOVA are listed in Table S3. The empty circles in the boxplot denote the data point of outliers.

The centroid sizes of the corollas of the Corytholoma species are illustrated in Fig. 4. The figure shows that the corolla sizes of *S. tubiflora* were much greater than the average. The within-species variance of the corolla size of *S. barbata* and *S. tubiflora* was larger than that of other species.

### Major 3D shape and form variations of the corollas

Major 3D shape variations among the flowers were identified using the full-GPA GM procedure described in the Methods section. The first 4 shape principal components (PCs), referred to as shape PC1 (sPC1) to sPC4, accounted for 52.57%, 21.39%, 7.99%, and 5.91%, respectively, of the total variance. Fig. 4A illustrates the major shape variations using virtual flowers. The virtual flower of the mean sPC values is illustrated in grey, and the virtual flowers with sPC values of mean ± 2 standard deviations (SD) are illustrated in pink.

**Figure 5:**
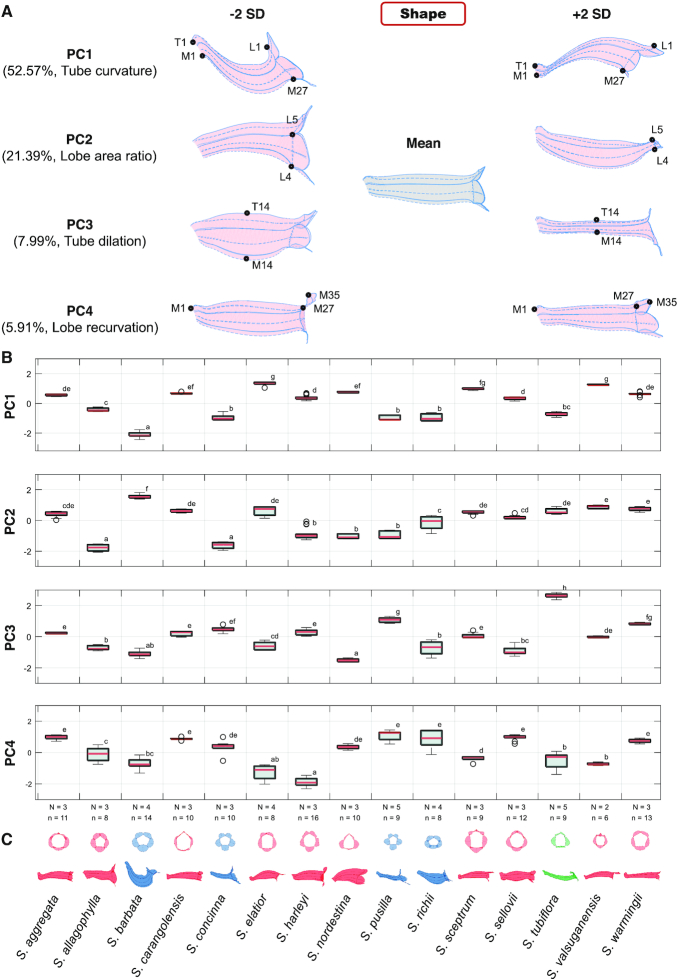
Major 3D shape variations of the Corytholoma flowers. (A) virtual flowers with an sPC value of mean ± 2 SD, (B) distributions of the sPC scores, and (C) mean corolla shapes. In (A), black dots represent labeled landmarks. L, M, and T represent landmarks on lobe contour, midrib, and tube–tube rim, respectively. In (B), the sPC scores are standardized to zero mean and unit variance. In (C), the corollas are colored by pollination type. Red, blue, and green represent hummingbird-pollinated, bee-pollinated, and moth-pollinated species, respectively. The lowercase letters at the right of the box plots denote groups of Scheffé’s multiple comparison tests performed at a confidence level of 0.99. N: number of plant individuals; n: number of specimens. The results of the ANOVA assumption tests and ANOVA are listed in Table S3. The empty circles in the boxplot denote the data point of outliers.

The 4 sPCs were linked to 4 specific shape transitions. sPC1 primarily corresponded to tube curvature. The tube of the corolla with a small sPC1 value was bent upward at a considerable degree (Fig. 5A). By contrast, the tube of the corolla with a large sPC1 value was bent downward. sPC2 principally corresponded to the lobe area size. The line connecting landmarks L4–L5 separates the lobe (right) and tube (left). The corolla with a small sPC2 value had a larger lobe area than that with a large sPC2 value. Particularly, the lobe area of the corolla with an sPC2 value of mean + 2 SD was nearly absent. sPC3 particularly corresponded to tube dilation (the distance between landmarks T14 and M14). The corolla with a small sPC3 value dilated in the tube, whereas the corolla with a large sPC3 value shrank in the tube. sPC4 principally corresponded to lobe recurvation. The lobe midrib (the line connecting landmarks M27–M35) of the corolla with a small sPC4 value was bent outward. By contrast, the lobe midrib of the corolla with a large sPC4 value was almost parallel to the tube midrib (the line connecting landmarks M1–M27).

Fig. 5B and C illustrates the distributions of the sPC scores and the face and side views of the mean corolla shape for each species. The sPC scores were standardized to zero mean and unit variance. Note that the within-species variance of sPC scores in most species increased from sPC1 to sPC4 (Fig. S1A). Particularly, the within-species variances of species *S. allagophylla, S. elatior, S. richii*, and *S. tubiflora* in sPC4 were larger than those of the other species.

**Figure 6:**
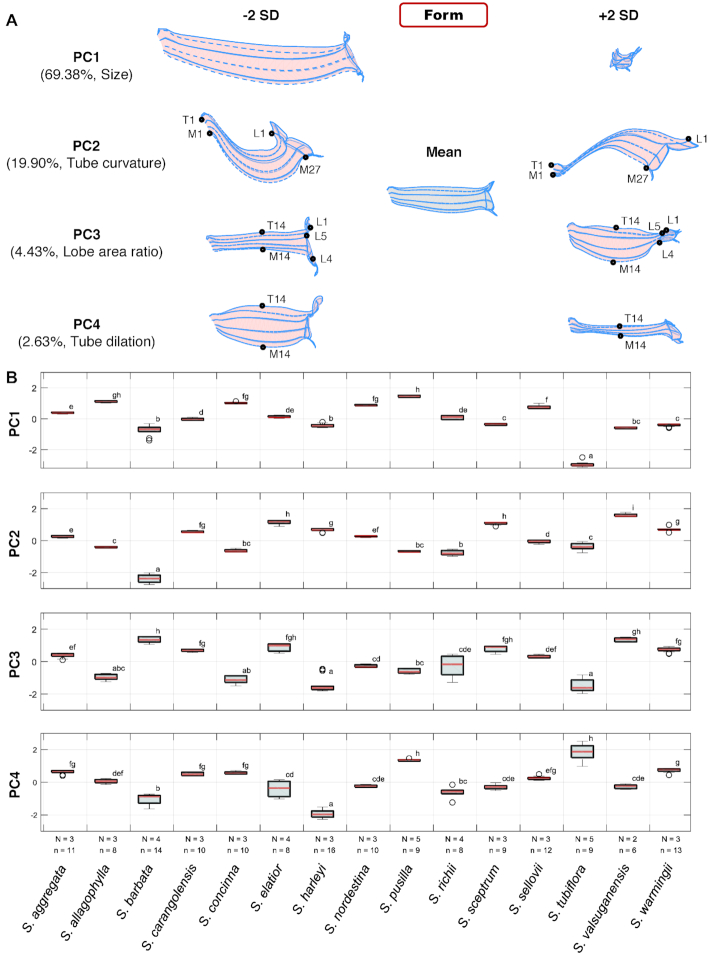
Major 3D form variations of the Corytholoma flowers: (A) virtual flowers with an fPC value of mean ± 2 SD and (B) distributions of the fPC scores. In (A), black dots represent labeled landmarks. L, M, and T represent landmarks on lobe contour, midrib, and tube-tube rim, respectively. In (B), the fPC scores are standardized to zero mean and unit variance. The lowercase letters at the right of the box plots denote groups of Scheffé’s multiple comparison tests performed at a confidence level of 0.99. N: number of plant individuals; n: number of specimens. The results of the ANOVA assumption tests and ANOVA are listed in Table S3. The empty circles in the boxplot denote the data point of outliers.

**Figure 7:**
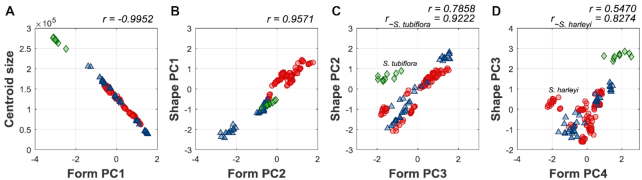
Analysis of correlation between the sPC and fPC scores. The PC scores are standardized to zero mean and unit variance. The correlation coefficients are provided at the upper right corners of scatter plots. Red circles, blue triangles, and green diamonds represent hummingbird-pollinated, bee-pollinated, and moth-pollinated species, respectively.

Major 3D form variations among the flowers were identified using the partial-GPA GM procedure. The first 4 form PCs, referred to as form PC1 to form PC4 (fPC1–fPC4), accounted for 69.38%, 19.90%, 4.43%, and 2.63%, respectively, of the total variance. Fig. 6A illustrates the major form variations using virtual flowers. fPC1 primarily corresponded to the corolla size. The corolla with a small fPC1 value had a large corolla size, whereas the corolla with a large fPC1 value had a small corolla size. In fact, fPC1 was negatively correlated with centroid size (*r* = −0.9952; Fig. 7A) and accounted for 73.69% of the total form variation. Notably, fPC2, fPC3, and fPC4, respectively, correlated with sPC1, sPC2, and sPC3 (*r* = 0.9571; *r* = 0.7858; *r* = 0.5470; Fig. 7B–D). However, *S. tubiflora* and *S. harleyi* did not follow the sPC2–fPC3 and sPC3–fPC4 correlations, respectively. The correlation coefficients increased considerably when these 2 species were excluded from the analyses (*r* = 0.9222 and *r* = 0.8274; Fig. 7C and D).

### Evolutionary allometry of the 3D corolla shape

**Figure 8:**
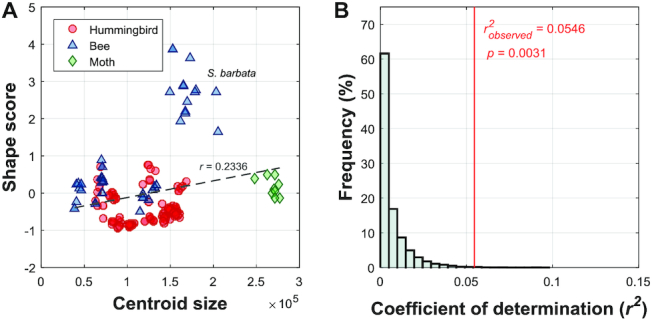
Evolutionary allometry of the corolla shapes in clade Corytholoma. Red circles, blue triangles, and green diamonds represent hummingbird-pollinated, bee-pollinated, and moth-pollinated species, respectively.

The evolutionary allometry of the corolla shape was assessed. To summarize the overall shape variation, the shape scores were calculated using full-GPA landmarks and multivariate regression (see Methods for details). The correlation coefficient of 0.2336 between the shape scores and centroid sizes revealed that the centroid size of the corollas accounted for only 5.46% of the shape variation (Fig. 8A). In addition, the permutation test indicated that the correlation was weak but statistically significant (Fig. 8B, *P* = 0.0031). Moreover, the low to medium levels of the correlations between sPCs and centroid size (Fig. S2) also supported that the allometry between shapes and size in Corytholoma was weak but significant.

### Morphological traits and their association with pollination type

**Figure 9:**
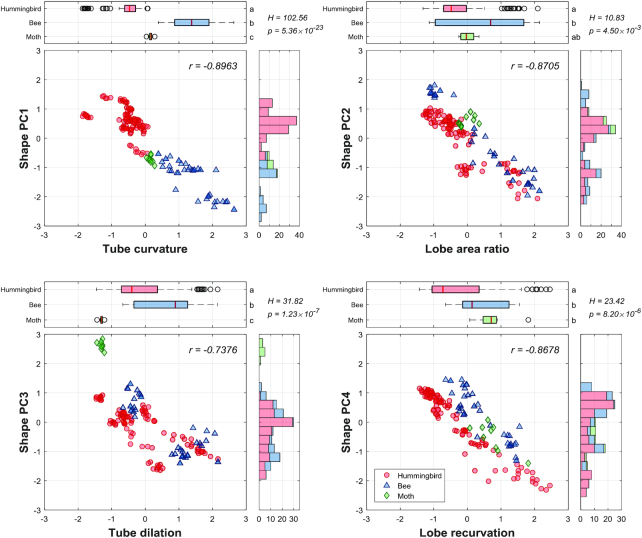
Scatter plots of the morphological traits and sPC scores. The trait and sPC scores are standardized to zero mean and unit variance. The correlation coefficients (*r*) are provided at the upper right corners of the scatter plots. The right panel of the boxplots presents the results of Kruskal-Wallis tests (*H* values). The lowercase letters at the right of the box plots denote groups of Scheffé’s multiple comparison tests performed with a confidence level of 0.99. The empty circles in the boxplot denote the data point of outliers.

Four morphological traits—tube curvature, lobe area ratio, tube dilation, and lobe recurvation—were defined on the basis of the variations of the first 4 sPCs. The traits were subsequently quantified from the 3D images of the corollas (see the Methods section for the calculation of shape scores). Correlation analyses indicated that the defined traits adequately describe the major shape variations (*r* ≥ 0.7376; Fig. 9). The pairwise correlations between the morphological traits indicated that the morphological traits were weakly correlated with each other (*r* ≤ 0.3109).

The association between the traits and pollination types was examined. The centroid size was also included in the analyses. Kruskal-Wallis test results indicated that the 4 traits and centroid size significantly differed between the pollination types (*P* < 4.50 × 10^−3^, Table 2). Scheffé’s multiple comparison test results suggested that tube curvature and tube dilation formed 3 clusters corresponding to the 3 pollination types (*P* < 3.09 × 10^−4^ and *P* < 3.42 × 10^−4^, respectively; Fig. 9; Table 2). The permutation test for logarithm of the odds (LOD) scores indicated that the centroid size and the tube curvature was significantly associated with the 3 pollination types (LOD = 21.71 and LOD = 45.52, *P* = 1.32 × 10^−14^ and *P* = 1.19 × 10^−22^, respectively).

**Table 2:** Kruskal-Wallis test results, Scheffé’s multiple comparison test results, and LOD scores of the morphological traits by pollination type

Morphological traits	Kruskal-Wallis test		Scheffé’s multiple comparison test						LOD score	*P*-value
	*H*-value	*P*-value	Hummingbird vs bee		Hummingbird vs moth		Bee vs moth			
			*T*-value	*P*-value	*T*-value	*P*-value	*T*-value	*P*-value		
Centroid size	26.81	1.51 × 10^–6^	1.76	2.15 × 10^–1^	11.23	1.11 × 10^–16^	11.49	1.11 × 10^–16^	21.71	1.32 × 10^–14^
Tube curvature	102.56	5.36 × 10^–23^	20.96	1.11 × 10^–16^	4.13	3.09 × 10^–4^	6.62	4.59 × 10^–9^	45.52	1.19 × 10^–22^
Lobe area ratio	10.83	4.50 × 10^–3^	3.71	1.38 × 10^–3^	0.55	8.60 × 10^–1^	1.34	4.08 × 10^–1^	2.92	1.60 × 10^–3^
Tube dilation	31.82	1.23 × 10^–7^	4.10	3.42 × 10^–4^	3.81	9.83 × 10^–4^	5.66	5.06 × 10^–7^	7.31	7.41 × 10^–11^
Lobe recurvation	23.42	8.20 × 10^–6^	3.60	2.02 × 10^–3^	2.92	1.57 × 10^–2^	0.95	6.34 × 10^–1^	3.91	1.80 × 10^–4^

### Phylogenetic signals of centroid size and morphological traits

**Figure 10:**
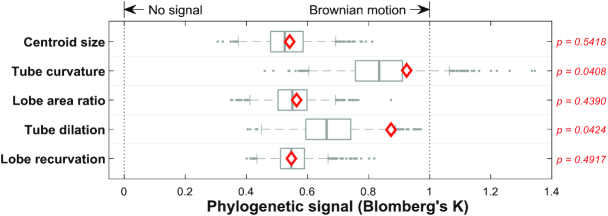
Phylogenetic signals of the centroid size and 4 morphological traits in clade Corytholoma. The distributions of the Blomberg K values were obtained using the phylogenetic trees of 1,000 replicates in the maximum likelihood analysis. Diamonds indicate the Blomberg K values calculated using the 50% majority-rule consensus tree. The gray circles in the boxplot denote the data point of outliers. The *P*-values are provided at the right of each boxplot.

The phylogenetic signals of the centroid size and 4 morphological traits were estimated (Fig. 10). The centroid size and 4 morphological traits of 5 specimens in each species were used (see Data Description for details). Blomberg K values of the tube curvature and tube dilation calculated using the 50% majority-rule consensus tree were 0.9250 and 0.8739, respectively. The permutation test for Blomberg K values rejected the null hypothesis that the 2 traits had no phylogenetic signal (*P* = 0.0408 for tube curvature and *P* = 0.0424 for tube dilation). These observations indicated that the change in the 2 traits approximated the Brownian motion model, and the 2 traits evolved gradually through time.

### 3D corolla shapes and forms at the ancestral states

**Figure 11:**
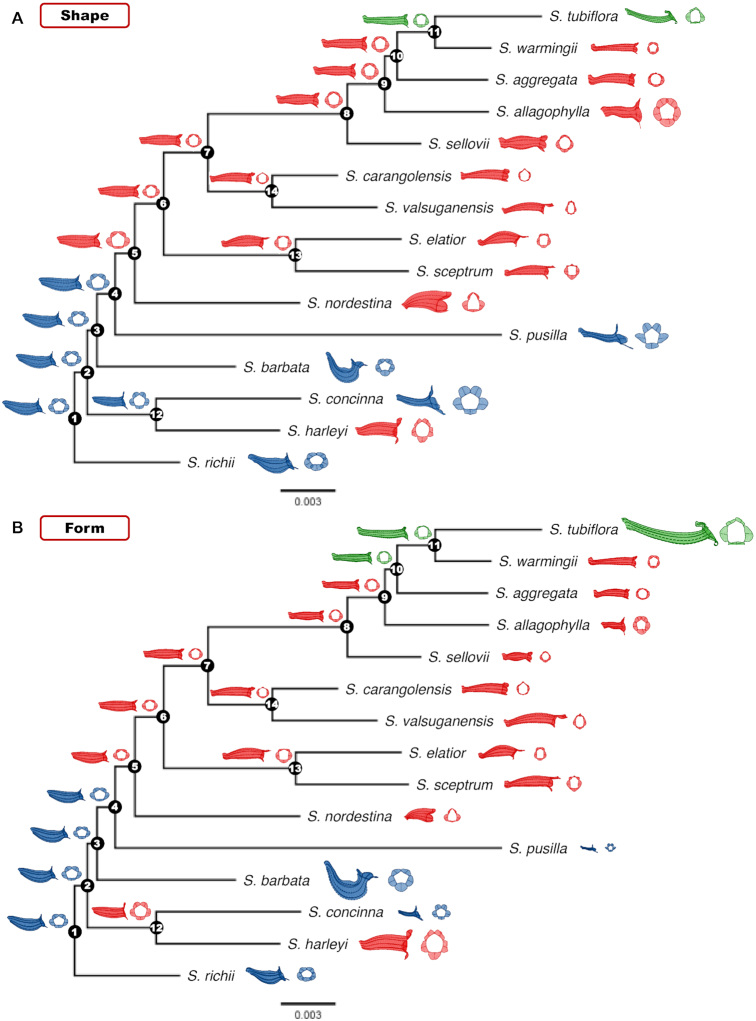
Reconstructed 3D corolla (A) shapes and (B) forms at the ancestral state for the Corytholoma species. The branch length indicates the number of substitutions per site, and the scale bar denotes 0.003 substitutions per site. The corolla colors of the extant species are assigned on the basis of the pollination type. Red, blue, and green denote hummingbird-, bee-, and moth-pollinated species, respectively. The corolla colors of the species at the ancestral states were estimated using the *k*-nearest neighbor algorithm with a *k* value of 5.

The 3D corolla shapes and forms at the ancestral states were reconstructed using the 50% majority-rule consensus tree (Fig. 11). For both the reconstructed shapes and forms, the corollas at nodes 1–4 and 12 bent upward, those at nodes 5–11 became straight and narrow, and those at nodes 13 and 14 bent downward in the tube. The measurement of centroid sizes and evaluation of morphological traits revealed more details on these transitions (Table S4). Centroid sizes of the corollas fluctuated from nodes 1 to 7 and increased gradually from nodes 8 to 11. Tube curvatures of the corollas decreased from nodes 1 to 11. Negative tube curvatures were observed on the corollas at nodes 13 and 14. Lobe area ratios of the corollas fluctuated from nodes 1 to 7 and decreased gradually from nodes 8 to 11. Tube dilations and lobe recurvations of the corollas also decreased from nodes 1 to 11. The decreasing trend extended to the nested nodes 12 to 14. The results obtained from the reconstructed corolla also indicated that the transitions in the traits were gradual.

The pollinator types of the 3D corolla shapes and forms at the ancestral states were estimated using sPC1–sPC4 and fPC1–fPC4, respectively, and the *k*-nearest neighbor algorithm with a *k* value of 5. The shifts in pollinator types were mostly consistent in both shape and form. We observed that the corolla shapes and forms at nodes 1–4 were estimated to be bee-pollinated, and the corolla shapes and forms at nodes 5–9 and 13–14 were estimated to be hummingbird-pollinated (Fig. 11). The corolla shape at node 11 was estimated to be moth-pollinated (Fig. 11A). The corolla shapes at nodes 10 and 12 were estimated to be hummingbird-pollinated and bee-pollinated, respectively. By contrast to the shape analysis, the corolla forms at nodes 10 and 12 were estimated to be moth-pollinated and hummingbird-pollinated, respectively (Fig. 11B). The size information (i.e., the difference between shape and form) altered the pollinator types at node 10 and 12.

**Figure 12:**
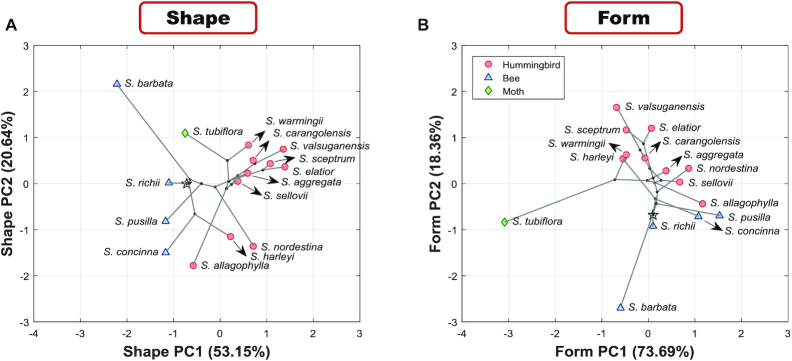
Distribution of the extant species and ancestral states in the morphospaces of the 3D corolla (A) shape and (B) form. The PC scores are standardized to zero mean and unit variance. White star and black points represent the ancestral state of node 1 (root) and ancestral states of nodes 2–14, respectively.

Fig. 12 presents the distributions of the extant species and ancestral states in the corolla shape and form morphospaces. In both morphospaces, *S. barbata* was ∼3 SDs away from the neighboring ancestral state. In the morphospace of the corolla form, *S. tubiflora* was ∼3 SDs away from the neighboring ancestral state. Both morphospaces were sparse in the neighborhoods of *S. barbata* and *S. tubiflora* compared with those of other species.

## Discussion

In this study, we acquired 3D corolla images of the Corytholoma species using µCT and identified the major 3D shape and 3D form variations of the corollas using landmark-based GM. We first revealed that the evolutionary allometry of corolla shapes was weak in Corytholoma species. According to the identified major shape variations, we defined and quantified 4 morphological traits—tube curvature, lobe area ratio, tube dilation, and lobe recurvation. We revealed that tube curvature and tube dilation were significantly associated with pollination type. The centroid size was also strongly associated with the pollination type. Taking together the trait values and phylogenetic information, we revealed strong phylogenetic signals in tube curvature and tube dilation. By reconstructing the corolla shapes, measuring the morphological traits at the ancestral states, and testing the phylogenetic signals of the traits, we discovered that the evolutionary changes in corolla shape were gradual in Corytholoma species.

### Resemblance of virtual flowers to corollas of clades other than clade Corytholoma

**Figure 13:**
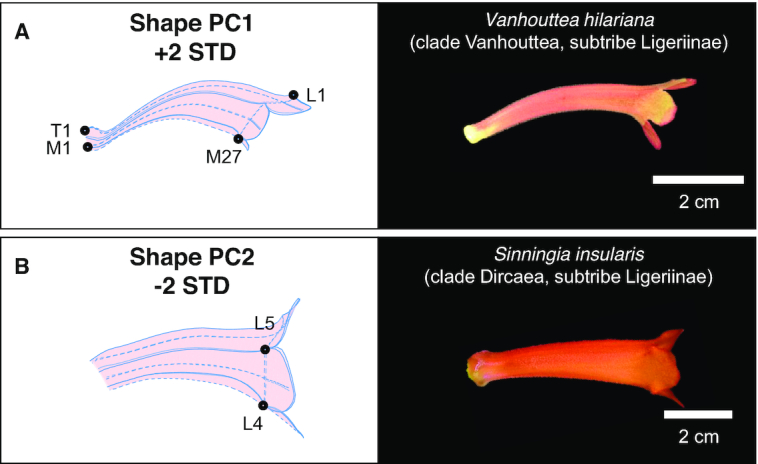
(A) Virtual flower of mean + 2D in sPC1 and the corolla image of *Vanhouttea hilariana* and (B) virtual flower of mean − 2D in sPC2 and the corolla image of *Sinningia insularis*.

The corolla shape variations identified in Corytholoma species resembled the corolla shapes of some species from other clades. The virtual flowers of mean + 2 SD in sPC1 and mean − 2 SD in sPC2 resembled the corollas of *Vanhouttea hilariana* (clade Vanhouttea, Fig. 13A) and *Sinningia insularis* (clade Dircaea, Fig. 13B), respectively. Surprisingly, some virtual flowers also resembled the corolla shapes of species of subtribes other than subtribe Ligeriinae. The virtual flower of mean + 2 SD in sPC1 (Fig. 5A) resembled the corolla of *Columnea microphylla* (subtribe Columneinae) [33]. The virtual flower of mean − 2 SD in sPC3 (Fig. 5A) resembled the corolla of *Drymonia urceolata* (subtribe Columneinae) [34].

### Evolutionary allometry and morphological integration of 3D corolla shape

The association between evolutionary allometry and morphological integration was supported by the evidence found in Corytholoma. After the emergence of GM in the last century, allometry has been believed to be associated with morphological integration [23, 35]. In Corytholoma, we revealed that the size only accounted for 5.46% of shape variation, suggesting that the allometry of corolla shape was weak. The mean of squared pairwise correlation coefficients between the 4 morphological traits was 0.0410, which suggested that the morphological integration in corolla shape was weak. Similar to the findings in a study of bird skulls [36], the aforementioned evidence indicated that evolutionary allometry and morphological integration are associated at a certain level. The present study suggests that the corolla shape in Corytholoma can serve as an example for the association between evolutionary allometry and morphological integration.

### Divergent corolla dominated the analysis of 3D shape and 3D form variation

Virtual flowers should be cautiously used to interpret the major shape and form variations of the corollas when species with extreme shapes or forms are included or when the species are sparse in the morphospace. The corolla shape and form of the Corytholoma species varied widely. In some studies on closely related species, the ranges of the PC scores were usually <3 SDs [11, 29]. By contrast, the PC scores in the present study spanned ≤5 SDs (Fig. 12). In the morphospace, *S. tubiflora* and *S. barbata* were distant from the clusters of the other species. Moreover, the space among *S. tubiflora, S. barbata*, and the cluster of the other species was large. The GM analysis reveals the major shape and form variations by applying linear interpolation or extrapolation to the landmarks of the species being studied (reviewed in [23]). Thus, the identified variation in the analysis could be considerably influenced by *S. tubiflora* and *S. barbata*. In other words, the divergent corolla shape of *S. tubiflora* and *S. barbata* would deviate the interpolated or extrapolated virtual flowers from reality. The most obvious example was the virtual flower of mean + 2 SD in fPC1 (Fig. 6A). The proximal part of the corolla protruded from the throat of the tube such that the corolla was inside out. Mean + 2D in fPC1 was beyond the cluster of the species in Fig. 12B. No corolla physically existing in nature can resemble such a virtual flower.

### Use of GM-derived morphological traits for phenotyping

Defining appropriate traits that correspond to shape variations is crucial for phenotyping. Conventionally, traits were defined on the basis of researchers’ observations and were quantified manually using calipers. After the emergence of GM and imaging techniques, some studies [16, 25, 37] have used PC scores from the GM analysis as the traits for phenotyping. Although comprehensive, PC scores were so complex that they could not specify the key changes in shape. We defined morphological traits by observing the major shape variations identified in the GM analysis. Subsequently, the traits were automatically measured from the 3D landmarks of the corollas. The proposed approach helped us objectively define and precisely quantify traits corresponding to major 3D shape variations.

### Limitations of 3D analysis of corolla shape evolution

Although we believe that this is one of the pioneering studies to integrate phylogenetic and 3D information to speculate regarding corolla shape evolution, the results may be affected by phylogenetic uncertainty [38]. Phylogenetic uncertainty is greatly increased owing to 2 issues: inconsistent tree topologies or branch lengths and incomplete sampling of extant species. We used the 1,000 phylogenetic trees obtained from each replicate for evaluating the phylogenetic signals. Therefore, the bias caused by relying on a single phylogenetic tree was avoided. The flower specimens of 2 species, *S. brasiliensis* and *S. aghensis*, with unique corolla shapes were not included in this study. In addition, the molecular sequences of newly added Corytholoma species [39], *S. helioana* and *S. muscicola*, were partially published. The topology of the phylogeny is data-dependent and could have been altered if these species had been included. However, inclusion of these specimens and sequences would have yielded more complete results of the analysis of corolla shape evolution.

## Potential Implications

The data preserved in the GigaScience database repository include 3D volumetric images, 3D surface images, and landmarks on the petal surface of the corollas. The 3D volumetric images can provide researchers the information regarding vascular bundles in the petal tissue. Experts on flower morphology, developmental biology, and plant taxonomy might focus on the anatomical information on petal tissue. The vascular bundle is crucial for morphology and development and plays an important role in the formation of petal tissue. Also, the 3D volumetric images can be used in education for vividly exhibiting the 3D structure of corollas. The 3D surface images can provide botanists the information on the surface, edge, and contour of petal tissue. Botanists might also be interested in the geometric information on petal tissue. The surface, edge, and contour are the fundamental features for understanding the geometric properties of the petal tissue. The landmarks can provide scientists the opportunities to practice the identification of corolla shape and form variations and the quantification of morphological traits. The results can also be used in studies comparing the corolla shape variations of clade Corytholoma with those of other taxa.

## Methods

### Major 3D shape and 3D form variations of the corollas

The major 3D shape and 3D form variations of corollas were identified from the landmarks obtained using GM. The major shape variations were determined using full-GPA [8]. Full-GPA removed the geometric information of the corollas related to translation, rotation, and scaling. The major form variations, defined as the combination of shape and size variations, were determined using partial-GPA. Partial-GPA removed the information of the corollas related to translation and rotation only. Following full- or partial-GPA, PCA was applied to the resulting landmarks. The obtained PCs were referred to as sPCs and fPCs. The first 4 sPCs and fPCs accounted for the majority of the variance and were used for representing the major 3D shape and 3D form variations between the corollas, respectively. Virtual flowers were created to visualize the major 3D shape and 3D form variations. The virtual flowers were obtained by performing an inverse PCA transform on a PC score [9].

### Evolutionary allometry of the 3D corolla shapes

The evolutionary allometry of the 3D corolla shapes in Corytholoma was evaluated using a multivariate regression analysis [40], correlation analysis, and permutation test [41]. In the multivariate regression analysis, regression coefficients were estimated using the full-GPA landmarks as the response variables and the centroid size as the predictor variable [40]. A shape score of the specimen was then obtained as the inner product of the full-GPA landmarks of the specimen and the vector of the regression coefficients. Subsequently, the correlation between the shape scores and centroid sizes was calculated. The square of the correlation coefficient indicated the degree of the size variation that accounted for the shape variation. Subsequently, the permutation test was performed to evaluate the dependency of the shape score on the centroid size. In the permutation test, the pairs of full-GPA landmarks and their associated centroid size were reshuffled among all the specimens 10,000 times. In each shuffle, the aforementioned multivariate regression analysis and correlation analysis were performed using the shuffled data to obtain a correlation coefficient. The accumulated squares of the correlation coefficients obtained from the 10,000 shuffles formed the null distribution of the permutation test. The *P*-value of the test was calculated as the proportion of the null distribution larger than the square of the correlation coefficient calculated using the unshuffled data. The *P*-value presented the level of the dependence of the shape score on the centroid size.

### Quantification of the morphological traits

**Figure 14:**
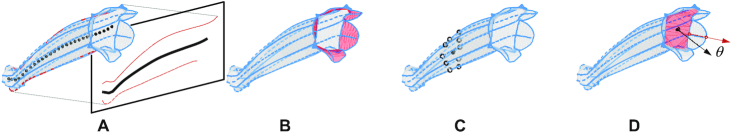
Illustration of corolla shape traits: (A) tube curvature, (B) lobe area ratio, (C) tube dilation, and (D) lobe recurvation. In (A), the dotted line indicates the tube axis, the parallelogram indicates the sagittal plane of the corolla, and the solid line indicates the mapping of the tube axis to the sagittal plane. In (B), the red area indicates the lobe of the corolla. In (C), the hollow dots indicate landmarks on the center tube transection, and the solid dot indicates the centroid of the landmarks. In (D), the red area indicates the tube-opening plane.

Morphological traits were defined by observing the variations of the first 4 sPCs and were directly quantified using the 3D corolla image. The traits included tube curvature, lobe area ratio, tube dilation, and lobe recurvation (Fig. 14). Tube curvature was defined as the second-order coefficient of the quadratic equation fitted to tube axis (dotted line in Fig. 14A). Tube axis was formed as the collection of the centroid points of the landmarks on the tube-tube rims and tube midribs that have the same order from the proximal part of the corolla. The centroid points were mapped to the sagittal plane of the tube (solid line in the parallelogram in Fig. 14A) before they were used for curve fitting. Lobe area ratio was defined as the ratio of lobe area (red area in Fig. 14B) to corolla surface area. The areas were calculated as the sizes of the triangle meshes connecting the landmarks surrounding the object. Tube dilation was defined as the ratio of the centroid size of center tube transection (the 14th landmarks from the proximal part of the corolla; hollow dots in Fig. 14C) to the length of the tube axis. The centroid size [42] of the center tube transection was defined as the root sum squared distance between the landmarks on the tube-tube rims or tube midribs and their centroid (solid dot in Fig. 14C). Lobe recurvation was defined as the mean of lobe bending angles of the 5 petals. The lobe bending angle for a petal (*θ* in Fig. 14D) was defined as the angle between the normal vector of the tube-opening plane (red area in Fig. 14D) and lobe-bending line (red line in Fig. 14D) of the petal. Tube-opening plane was defined as the plane optimally fitting the landmarks on the lobe-tube rim. The lobe-bending line of a petal was defined as the line connecting the proximal and distal landmarks on the lobe midrib.

### Association between morphological traits and pollination type

The association between morphological traits and pollination type and the level of the association were evaluated using LOD scores [43] and permutation tests, respectively. To calculate the LOD score for a trait, the ratio of the squared deviation of the trait to the sum of the within-group squared deviations of the trait was first calculated. The groups referred to pollinator types. The LOD score of the trait was then obtained as the logarithm of the ratio. A large LOD score indicates a strong association. Next, permutation tests were conducted to evaluate the levels of the association between the traits and pollination type. In a permutation test, pairs comprising a morphological trait and its pollination type were reshuffled among all the specimens 10,000 times. In each shuffle, an LOD score was calculated using the aforementioned procedure and shuffled data. The cumulative LOD scores of the 10,000 shuffles formed the null distribution of the permutation test. The *P-*value for the test was subsequently calculated as the frequency of the null distribution higher than the LOD score calculated using the unshuffled data. The *P-*value presented the level of association between the morphological traits and pollination type.

### Phylogenetic analysis

The phylogeny of the Corytholoma species was obtained using a maximum likelihood (ML) analysis. In the procedure, the sequences of 6 molecular markers of the Corytholoma species were gathered from published data ([4]; Table S5). The sequences were aligned using MAFFT [44], without manual adjustment. The alignments of the markers were then concatenated to obtain 4,414 sites. Subsequently, the combination of the HKY85 model, the estimated proportion of invariant sites (+I), and the variable site following a γdistribution (+gamma) was suggested by Modeltest 3.7 software [45], which is the best-fit nucleotide substitution model for the aligned sequence. The ML analysis was then performed using the aligned sequence, the aforementioned model, and parameters suggested by Modeltest 3.7, and GARLI 2.0 software [46] for 1,000 replicates. The 50% majority-rule consensus tree of the 1,000 replicates was then used as the representative phylogeny of the Corytholoma species for the following analyses.

### Tests of phylogenetic signals

The phylogenetic signals of the centroid size and morphological traits were evaluated using Blomberg K values [47] and permutation tests. Blomberg K value for a trait was calculated using the mean trait values of all the species, the phylogenetic trees of 1,000 replicates from the ML analysis, and the “phylosig” function in the R package phytools [48]. A K value of zero indicates no phylogenetic signal in the trait, whereas a K value of 1 indicates a strong phylogenetic signal; the trait evolution follows the Brownian motion model. The permutation tests were then performed to evaluate whether the K values significantly differed from zero. In a permutation test, the pairs of the species positions on the phylogenetic tree and the mean trait value were reshuffled 10,000 times. In each shuffle, the K value was calculated using the 50% majority-rule consensus tree. The cumulative K values of the 10,000 shuffles formed the null distribution of the permutation test. The *P-*value for the test was then calculated as the proportion of the null distribution larger than the K value calculated using the unshuffled data. The *P-*value presented the level that the K value differed from zero.

### Reconstruction of the 3D corolla shapes and forms at the ancestral states

3D corolla shapes and forms were reconstructed at the ancestral states in the phylogeny using weighted squared-change parsimony [49], the 3D landmarks of mean corolla shapes and forms of all the extant species, and the 50% majority-rule consensus tree. After reconstruction, the corolla forms were used for quantifying the centroid sizes and morphological traits at the ancestral states. To further assess the variation in the corolla shape and form at each ancestral state, the reconstruction was repeated 100 times. In each repetition, the mean corolla shape or form was calculated from 3 specimens that were randomly selected from the 5 specimens of each species. The variation of the 100 reconstructed 3D corolla shapes and forms was presented (Table S4).

## Availability of Supporting Data and Materials

The presented dataset and other data supporting this work are deposited in the *GigaScience* GigaDB repository [50]. We provided (i) the 2D slice images of the specimen obtained from the µCT scanner, (ii) the 3D volumetric images composed of the 2D slice images, (iii) the 3D surface images converted from the volumetric images, and (iv) the 3D landmarks of the corollas identified from the surface images. Representative 3D scans and 3D printer (PLY) files are also available to download and view from GigaDB and associated SketchFab and Thingiverse repositories.

## Additional Files

Figure S1. Within-species variance of the extant species in (A) sPCs and (B) fPCs. Red circles, blue triangles, and green diamonds represent hummingbird-pollinated, bee-pollinated, and moth-pollinated species, respectively.

Figure S2. Scatter plot of centroid size versus each sPC.

Table S1. List of studies using landmark-based GM.

Table S2. The specimen information and the scan parameters.

Table S3. The tests of normality and equal variance and the ANOVA of centroid size, shape PCs, and form PCs.

Table S4. Centroid size and 4 morphological traits of extant species and ancestral states.

Table S5. Species list and GenBank numbers.

giz155_GIGA-D-19-00247_Original_Submission

giz155_GIGA-D-19-00247_Revision_1

giz155_GIGA-D-19-00247_Revision_2

giz155_Response_to_Reviewer_Comments_Original_Submission

giz155_Response_to_Reviewer_Comments_Revision_1

giz155_Reviewer_1_Report_Original_Submission Daniel H Chitwood -- 7/21/2019 Reviewed

giz155_Reviewer_2_Report_Original_Submission Mao Li -- 8/7/2019 Reviewed

giz155_Reviewer_2_Report_Revision_1 Mao Li -- 9/24/2019 Reviewed

giz155_Supplemental_Figures_and_Tables

## Abbreviations

ANOVA: analysis of variance; GM: geometric morphometrics; GPA: generalized Procrustes analysis; KBCC: Dr. Cecilia Koo Botanic Conservation Center; LDA: linear discriminant analysis; LOD: logarithm of the odds; µCT: micro-computed tomography; ML: maximum likelihood; PCA: principal component analysis; SD: standard deviation.

## Competing Interests

The authors declare that they have no competing interests.

## Funding

This research was supported by NSC-101-2313-B-002-050-MY3 from National Science Council (Ministry of Science and Technology) of Taiwan.

## Authors' Contributions

H.C.H. and Y.F.K. conceived the project; H.C.H. and W.C.C. maintained the plant materials, collected the flower specimens, performed the landmark identification, conducted the 3D GM analyses, quantified the morphological traits, and reconstructed the 3D corolla shapes and forms at the ancestral states; H.C.H. performed the analysis of evolutionary allometry, the phylogenetic analysis, the association between the morphological traits and pollination type, and the test of phylogenetic signals; H.C.H. and Y.F.K. prepared the manuscript; Y.F.K. managed and supervised the work.
