# Supplementary material for: 3D revelation of phenotypic variation, evolutionary allometry, and ancestral states of corolla shape: a case study of clade Corytholoma (subtribe Ligeriinae, family Gesneriaceae)
Source: Gigascience. 2020 Jan 22;9(1):giz155. doi: 10.1093/gigascience/giz155 (PMC6974915; doi:10.1093/gigascience/giz155)
Supplement: giz155_GIGA-D-19-00247_Original_Submission [file giz155_giga-d-19-00247_original_submission.pdf]

// GigaScience

# 3D revelation of phenotypic variation, evolutionary allometry, and ancestral states of corolla shape: a case study of clade Corytholoma (subtribe Ligeriinae, family Gesneriaceae)

--Manuscript Draft--

| | |
|---|---|
| **Manuscript Number:** | GIGA-D-19-00247 |
| **Full Title:** | 3D revelation of phenotypic variation, evolutionary allometry, and ancestral states of corolla shape: a case study of clade Corytholoma (subtribe Ligeriinae, family Gesneriaceae) |
| **Article Type:** | Research |
| **Funding Information:** | Ministry of Science and Technology, Taiwan (TW) (NSC-101-2313-B-002-050-MY3) — Dr. Yan-Fu Kuo |
| **Abstract:** | Background:<br>Quantification of corolla shape variations helps biologists to investigate the diversity and evolution in plants. This study applied X-ray microcomputed tomography (μCT) to acquire three-dimensional (3D) structures of the corollas of clade Corytholoma. After acquiring volumetric images of the corollas and extracting a set of 415 3D landmarks from each volumetric image, the major shape and form variations of the corollas were identified from the landmarks by using geometric morphometrics (GM). Evolutionary allometry of the corolla shape was assessed. Morphological traits corresponding to the major shape variations were also defined and quantified and were subsequently used to examine their association with pollination type and to evaluate the phylogenetic signals. The landmarks were further used to reconstruct corolla shapes at the ancestral states.<br><br>Results:<br>GM results revealed that the first four principal components (PCs) in the shape and form analyses, respectively, accounted for 87.45% and 98.36% of the total variance. The centroid sizes of the corollas only accounted for 4.65% of the corolla shape variation, suggesting that the evolutionary allometry was weak. The four morphological traits corresponding to the four shape PCs were defined as tube curvature, lobe area, tube dilation, and lobe recurvation. Tube curvature and tube dilation were strongly associated with the pollination type and contained phylogenetic signals in clade Corytholoma.<br><br>Conclusions:<br>With the integration of μCT imaging into GM, the proposed approach boosted the precision in quantifying corolla traits and improved the understanding of the morphological traits corresponding to the pollination type, impact of size on shape variation, and evolution of corolla shape in clade Corytholoma. |
| **Corresponding Author:** | Hao-Chun Hsu<br><br>TAIWAN |
| **Corresponding Author Secondary Information:** | |
| **Corresponding Author's Institution:** | |
| **Corresponding Author's Secondary Institution:** | |
| **First Author:** | Hao-Chun Hsu |
| **First Author Secondary Information:** | |
| **Order of Authors:** | Hao-Chun Hsu<br>Wen-Chieh Chou |

Powered by Editorial Manager® and ProduXion Manager® from Aries Systems Corporation

| | |
|---|---|
| | Yan-Fu Kuo |
| **Order of Authors Secondary Information:** | |
| **Additional Information:** | |
| **Question** | **Response** |
| Are you submitting this manuscript to a special series or article collection? | No |
| **Experimental design and statistics**<br><br>Full details of the experimental design and statistical methods used should be given in the Methods section, as detailed in our Minimum Standards Reporting Checklist. Information essential to interpreting the data presented should be made available in the figure legends.<br><br>Have you included all the information requested in your manuscript? | Yes |
| **Resources**<br><br>A description of all resources used, including antibodies, cell lines, animals and software tools, with enough information to allow them to be uniquely identified, should be included in the Methods section. Authors are strongly encouraged to cite Research Resource Identifiers (RRIDs) for antibodies, model organisms and tools, where possible.<br><br>Have you included the information requested as detailed in our Minimum Standards Reporting Checklist? | Yes |
| **Availability of data and materials**<br><br>All datasets and code on which the conclusions of the paper rely must be either included in your submission or deposited in publicly available repositories (where available and ethically appropriate), referencing such data using a unique identifier in the references and in | Yes |

| | |
|---|---|
| the "Availability of Data and Materials" section of your manuscript.<br><br>Have you have met the above requirement as detailed in our Minimum Standards Reporting Checklist? | |

Powered by Editorial Manager® and ProduXion Manager® from Aries Systems Corporation

Manuscript

Click here to view linked References

Click here to access/download;Manuscript;17Genus Sinningia 3D GM_v5.4_submitted.docx

# 3D revelation of phenotypic variation, evolutionary allometry, and ancestral states of corolla shape: a case study of clade Corytholoma (subtribe Ligeriinae, family Gesneriaceae)

**Hao-Chun Hsu, Wen-Chieh Chou, Yan-Fu Kuo***

Department of Biomechatronics Engineering, National Taiwan University, Taipei, Taiwan

*** Correspondence:** Dr. Yan-Fu Kuo, Department of Biomechatronics Engineering, National Taiwan University, No. 1, Sec. 4, Roosevelt Rd. Taipei, 106, Taiwan. Phone: +886-2-33665329; Fax: +886-2-23627620; E-mail: ykuo@ntu.edu.tw.

## Abstract

**Background:**

Quantification of corolla shape variations helps biologists to investigate the diversity and evolution in plants. This study applied X-ray microcomputed tomography (μCT) to acquire three-dimensional (3D) structures of the corollas of clade Corytholoma. After acquiring volumetric images of the corollas and extracting a set of 415 3D landmarks from each volumetric image, the major shape and form variations of the corollas were identified from the landmarks by using geometric morphometrics (GM). Evolutionary allometry of the corolla shape was assessed. Morphological traits corresponding to the major shape variations were also defined and quantified and were subsequently used to examine their association with pollination type and to evaluate the phylogenetic signals. The landmarks were further used to reconstruct corolla shapes at the ancestral states.

**Results:**

GM results revealed that the first four principal components (PCs) in the shape and form analyses, respectively, accounted for 87.45% and 98.36% of the total variance. The centroid sizes of the corollas only accounted for 4.65% of the corolla shape variation, suggesting that the evolutionary allometry was weak. The four morphological traits corresponding to the four shape PCs were defined as tube curvature, lobe area, tube dilation, and lobe recurvation. Tube curvature and tube dilation were strongly associated with the pollination type and contained phylogenetic signals in clade Corytholoma.

**Conclusions:**

With the integration of μCT imaging into GM, the proposed approach boosted the precision in quantifying corolla traits and improved the understanding of the morphological traits corresponding to the pollination type, impact of size on shape variation, and evolution of corolla shape in clade Corytholoma.

**Keywords:** Corytholoma, Corolla shape variations, Evolutionary allometry, Geometric morphometrics (GM), generalized Procrustes analysis (GPA), Ligeriinae, X-ray micro-computed tomography (μCT)

## Background

The variation in corolla shapes in angiosperms has received considerable research attention [1, 2]. This variation was believed to be principally attributed to the specialization in animal-mediated pollination. Particularly, the species in clade Corytholoma of subtribe Ligeriinae (family Gesneriaceae) yield flowers with assorted shapes (tubular, funnel, and bell-shaped; Fig. 1) and various sizes (1–9 cm in length) and are associated with different pollinators [3]. Because of the rapid change in optimized corolla morphologies in a monophyletic group, the corollas serve as excellent materials for studying pollinator association and identifying the shape transition of the corollas. As corollas are complex three-dimensional (3D) objects, an approach should be developed for appropriately assessing their shape and size. This study applied X-ray micro-computed tomography (µCT) and 3D geometric morphometrics (GM) [4, 5] for identifying the major shape variations of the corollas, revealing the association between the corolla shape and pollination type, and elucidating the evolution of corolla shape in clade Corytholoma.

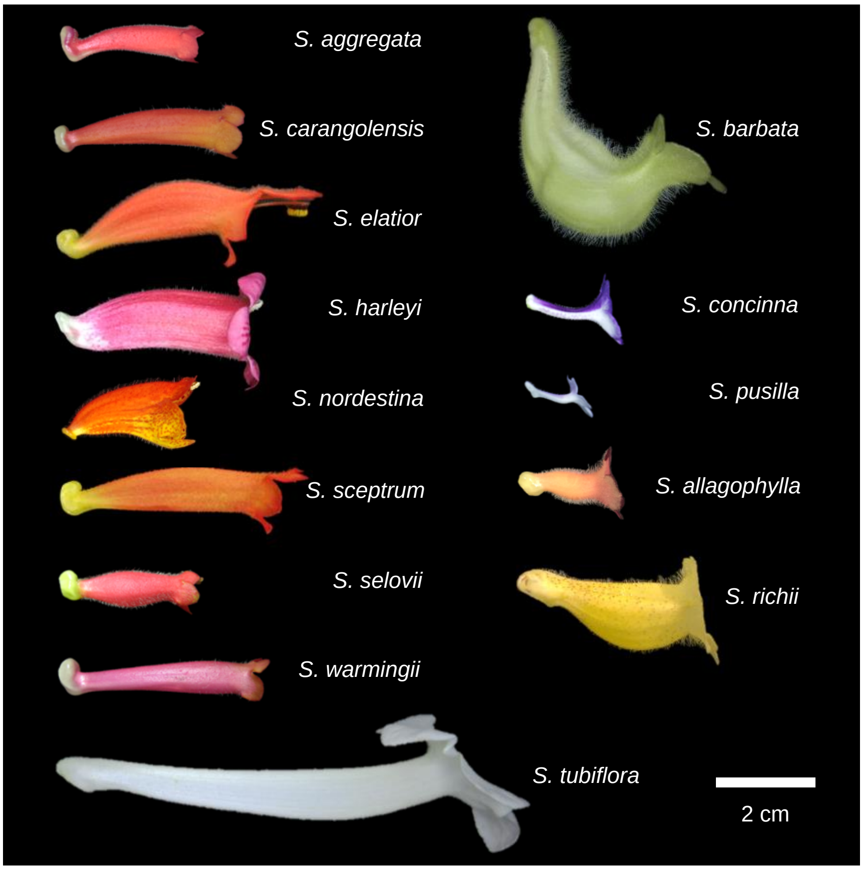


Figure 1. Side-view of the corolla of the species in clade Corytholoma.

In the last decade, landmark-based GM has been frequently applied to quantify shape variations of corollas (Table 1). Through landmark-based GM, the spatial variations of corolla landmarks (i.e., characteristic points of interest) can be extracted using generalized Procrustes analysis (GPA) [6, 7] and the major variations between the landmarks can be summarized using dimensionality reduction techniques (e.g., principal component analysis [PCA] or linear discriminant analysis [LDA]). Landmark-based GM can be two-dimensional (2D) or 3D. In studies using 2D GM, the identification of major shape variations was restricted by the imaging views (e.g., side, face, or dissected) of the corollas. However, corollas are objects with a complex 3D geometry. None of the three views provides complete information of the corolla structures [8]. This shortcoming can be overcome by combining 3D imaging techniques with GM [9]. In studies using 3D GM [8, 10, 11], the structural information of the whole corollas was comprehensively captured and retained.

Table 1. List of studies using landmark-based GM.

| Approach | Imaging object | Keywords (selected) | Family | Landmark* | | GM | | References |
|---|---|---|---|---|---|---|---|---|
| | | | | Primary | Secondary | GPA | PCA/LDA | |
| 2D | Side-view | Petal shape; Floral morphology | Gesneriaceae | 5 | 10 | Full | PCA | [12] |
| | | Pollination syndrome; Phylogenetic comparative methods | Gesneriaceae | 6 | 26 | Full | PCA | [13, 14] |
| | | Pollinator selection; Flower shape | Loasaceae | 5 | 0 | Full | PCA, LDA | [15, 16] |
| | | Flower shape; Pollination niches | Plantaginaceae | 4 | 12 | Full | PCA | [17] |
| | | Trait-dependent diversification; Pollination; Flower tube | Plantaginaceae | 9 | 1 | Full | LDA | [18] |
| | | Ancillary traits | Rubiaceae | 9 | 8 | Full | PCA, LDA | [19] |
| | Face-view | Selfing syndrome | Brassicaceae | 30 | 0 | Full | PCA | [20] |
| | | Floral shape evolution; pollination; corolla shape; plant-pollinator interactions; floral morphospace; allometry | Brassicaceae | 32 | 0 | Full | PCA | [21–26] |
| | | Petal shape; Floral morphology | Gesneriaceae | 5 | 25 | Full | PCA | [12] |
| | | Floral shape** | Goodeniaceae | 5 | 0 | Full | PCA | [27] |
| | | Fluctuating asymmetry | Orchidaceae | 4 | 5 | Full | PCA | [28] |
| | | Flower shape | Plantaginaceae | 28 | 0 | Full | PCA | [29] |
| | | Corolla shape; pollination | Solanaceae | 5 | 35 | Full | PCA | [30] |
| | | Floral symmetry | Valerianaceae | 10 | 0 | Full | LDA | [31] |
| | Dissected-view | Actinomorphy; Zygomorphy | Leguminosae | 2 | 18 | Full | PCA | [32] |
| | | Floral shape** | Leguminosae | 37 | 0 | Full | PCA | [33] |
| | | Directional asymmetry; floral organ shape; fluctuating asymmetry | Iridaceae | 39 | 16 | Full | PCA | [34, 35] |
| | | Floral shape** | Orchidaceae | 15 | 0 | Full | PCA | [36] |
| | | Floral shape, allometry** | Plantaginaceae | 4 | 16 | Partial | PCA | [37] |
| | | Floral shape** | Plantaginaceae | 8 | 47 | Partial | PCA | [38] |
| 3D | Whole corolla | Petal shape; dorsoventral asymmetry | Gesneriaceae | 10 | 65 | Full | PCA | [8] |
| | | Dorsoventral asymmetry; petal form variation | Gesneriaceae | 25 | 100 | Full | PCA | [11] |
| | | Floral shape, pollination** | Orchidaceae | 26 | 14 | Full | PCA | [10] |
| | | Corolla shape variations | Gesneriaceae | 25 | 390 | Full/ Partial | PCA | Present study |

* The primary and secondary landmarks follow the definitions in the Methods section.
** Keywords were not provided in these studies. The keywords were assigned by authors of the present study.

The 3D corolla information can promote the specificity for studying the allometry of corolla shape. Allometry refers to the change in target traits in response to the change in size [39]. Conventionally, allometry studies are limited to investigating the relationship between two distance-based measurements, such as lengths and widths, of objects [40]. After the emergence of GM techniques, some studies have investigated the allometry of corollas in the geometry aspect using 2D images [26, 37]. However, these studies still faced the

aforementioned shortcoming that 2D images inadequately capture the structural information of 3D objects. Additionally, 2D images are usually acquired manually, which may introduce error or artifacts due to inconsistent adjustments during the operation. Therefore, the allometry of corolla shape can be assessed more accurately and comprehensively by utilizing the precision and integrity of 3D imaging.

The major shape variations of the corollas identified through GM can also help in examining the association between corolla shape and pollinators. Corolla shape is one of the most prominent indicators associated with pollinator type [41, 42]. Conventionally, distance-based traits (e.g., diameter of corolla orifices and length of corolla tube) were used to evaluate pollination association [43–45]. However, distance-based traits are typically proposed based on manual observation and can be subjective. Additionally, these traits could be oversimplified and may not adequately describe the geometric properties of the corollas [46–48]. By contrast, the shape variations obtained using GM were identified through a serious of statistical procedures; thus, they could adequately represent the principal shape differences among the corollas. Gómez et al. [22] and Kaczorowski et al. [30] used the corolla shape variations quantified using 2D GM to examine the association between plant species and pollinators in *Erysimum* and *Nicotiana*, respectively. Traits identified using 3D GM precisely describe the leading variations in the geometric properties of corollas; thus, they can serve as excellent candidates in the tests of pollinator association.

Corolla shapes at the ancestral states is another intriguing research topic for biologists. To infer history and interpret the evolution of species, the characteristics of the species and their transitions along phylogeny are reconstructed and evaluated [49, 50]. The corolla shapes at the ancestral states can be reconstructed using a given phylogeny and corolla landmarks of the extant species [51]. Gómez et al. [25] reconstructed the corolla shapes in *Erysimum* and visualized the changes in shape at the ancestral states using GM and 2D landmarks in the face

view. Joly et al. [14] identified the evolutionary constraints on corolla shape in Gesneriaceae using GM and 2D landmarks in the side view. The corollas reconstructed using face or side views only provide a part of structural information of the corollas. By contrast, the corolla reconstructed using 3D landmarks shows complete structural information. Thus, 3D images may reveal more information regarding the transition of the corolla shapes at the ancestral states.

This study scrutinized the corolla shapes and forms of the species in clade Corytholoma. We used the 3D approach to acquire the images of the corollas; thus, the complete structural information of the corollas was retained. We selected 415 landmarks for each corolla; thus, the structures of the corollas were genuinely represented. We performed GM analyses on the landmarks to identify both the shape and form variations of the corollas; thus, the impact of corolla size on corolla shape could also be examined. We defined morphological traits of the corollas based on the GM results and quantified the traits directly using the 3D corolla images; thus, the traits were proposed statistically rather than manually. The proposed traits were subsequently used for investigating the association between pollination type and corolla shapes; thus, the leading shape variations could be used and interpreted in the association tests. We further evaluated the phylogenetic signals of corolla size and morphological traits; thus, the tempo and mode of corolla evolution could be assessed. Last, we reconstructed corolla shapes at the ancestral states using 3D landmarks; thus, more information regarding the shape transition of the corolla could be revealed.

## Data Description

Flower materials

One to two plant individuals of 15 species (Table 2) in clade Corytholoma were obtained from Dr. Cecilia Koo Botanic Conservation Center (Pingtung, Taiwan; Appendix 1). Two plant individuals were received for three species (*S. elatior*, *S. pusilla*, and *S. tubiflora*), the individuals of which were bred from the same single seed set of a $F_2$ inbred line. The plants were cultivated under natural lighting, 70%–80% humidity, and at 22–28°C in a greenhouse (Technology Commons X, College of Life Science, National Taiwan University, Taiwan). Five flowers were collected from each species between August 2015 and August 2016, resulting in 75 specimens. The Corytholoma species are protandrous. To minimize the developmental variations between species, collection was conducted at the developmental stage between anther and stigma anthesis. The specimens of the same species were collected in the same season to alleviate the shape variations caused by different flowering seasons. The specimens were prepared in fresh or were fixed in 70% ethanol solution (Table 2).

Table 2. Species list and dimension of the slice images.

| Species | Pollination Type* | Specimen type** | Dimension of slice image (pixels) | Number of slice image | KBCC accession |
|---|---|---|---|---|---|
| *S. aggregata* | H | F | 1000×1000 | 540-610 | K039092 |
| *S. allagophylla* | H | F | 1000×1000 | 540-610 | K039099 |
| *S. barbata* | B | E/F | 1968×1968*** | 1150-1390 | K039105 |
| *S. carangolensis* | H | F | 1000×1000 | 1120-1220 | K039112 |
| *S. concinna* | B | F | 1000×1000 | 580-680 | K039118 |
| *S. elatior* | H | E | 1000×1000 | 1045-1370 | K039127, K039129 |
| *S. harleyi* | H | F | 1000×1000 | 1280-1400 | K039135 |
| *S. nordestina* | H | F | 1000×1000 | 780-800 | K039168 |
| *S. pusilla* | B | F | 1000×1000 | 609-666 | K039169, K039170 |
| *S. richii* | B | E/F | 1000×1000 | 1060-1160 | K039174 |
| *S. sceptrum* | H | E | 1000×1000 | 1260-1298 | K039178 |
| *S. sellovii* | H | E | 1000×1000 | 930-1000 | K039184 |
| *S. tubiflora* | M | F | 1968×1968*** | 2230-2470 | K039197, K039200 |
| *S. valsuganensis* | H | F | 1000×1000 | 1540-1520 | K039203 |
| *S. warmingii* | H | E | 1000×1000 | 1160-1320 | K039205 |

* H: hummingbird pollination (ornithophily), B: bee pollination (melittophily), and M: moth pollination (phalaenophily).
** The letter E denotes the 70% ethanol-fixed specimen, and the letter F denotes the fresh specimen.
***The 3D images with a slice size of 1968 × 1968 were downsized to 984 × 984 before the reconstruction of volumetric and surface images. The identified landmarks were then magnified back to the original scale for the subsequent GM analysis.

The information on pollination types was obtained from Perret et al. [3]. The species were associated with three pollination types: hummingbird, bee, and moth (Table 2). The hummingbird-pollinated species have tubular corollas with red, orange, or yellow colors (Fig. 1). The bee-pollinated species have campanulate or salverform corollas with purple or white colors. The moth-pollinated species have narrow and long tubular corollas with pale color.

3D flower image data

The 3D images of the flowers were acquired using an X-ray µCT scanner (SkyScan 1076, Bruker; Kontich, Belgium). A 3D image was composed of hundreds or thousands of 2D slice images along the longitudinal axis. Image processing algorithms were applied to the slice images for segmenting the corollas from the background and for reducing the noise of the images. The processed 2D slice images of a corolla were then integrated into a 3D volumetric image (Fig. 2B). The volumetric image was then converted to a surface image (Fig. 2C) in a polygon file format (i.e., a PLY file) for the landmark identification. The details of the µCT setups, the graphical user interface program including all image processing procedures, and the demonstration video for the program can be found in the report by Wang et al. [8]. The 2D slice images, volumetric images, and the surface images of the specimens in this study are available in the GigaScience Database repository (GigaDB number).

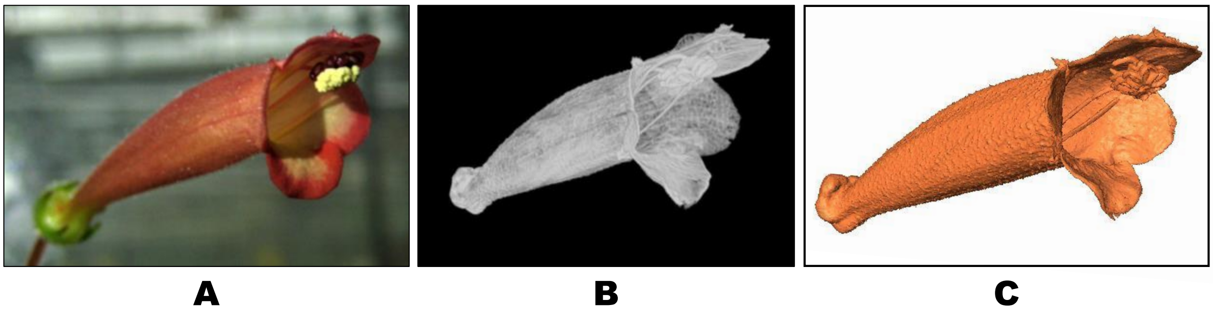


Figure 2. (A) Photograph, (B) volumetric image, and (C) surface image of a corolla of *S. sceptrum*.

**Analyses**

Corolla centroid size

The centroid sizes of the corollas of the Corytholoma species are illustrated in Figure 3A. The figure shows that the corolla sizes of *S. barbata* and *S. tubiflora* were greater than the average. The within-species variance of the corolla size of the two species was also larger than that of other species.

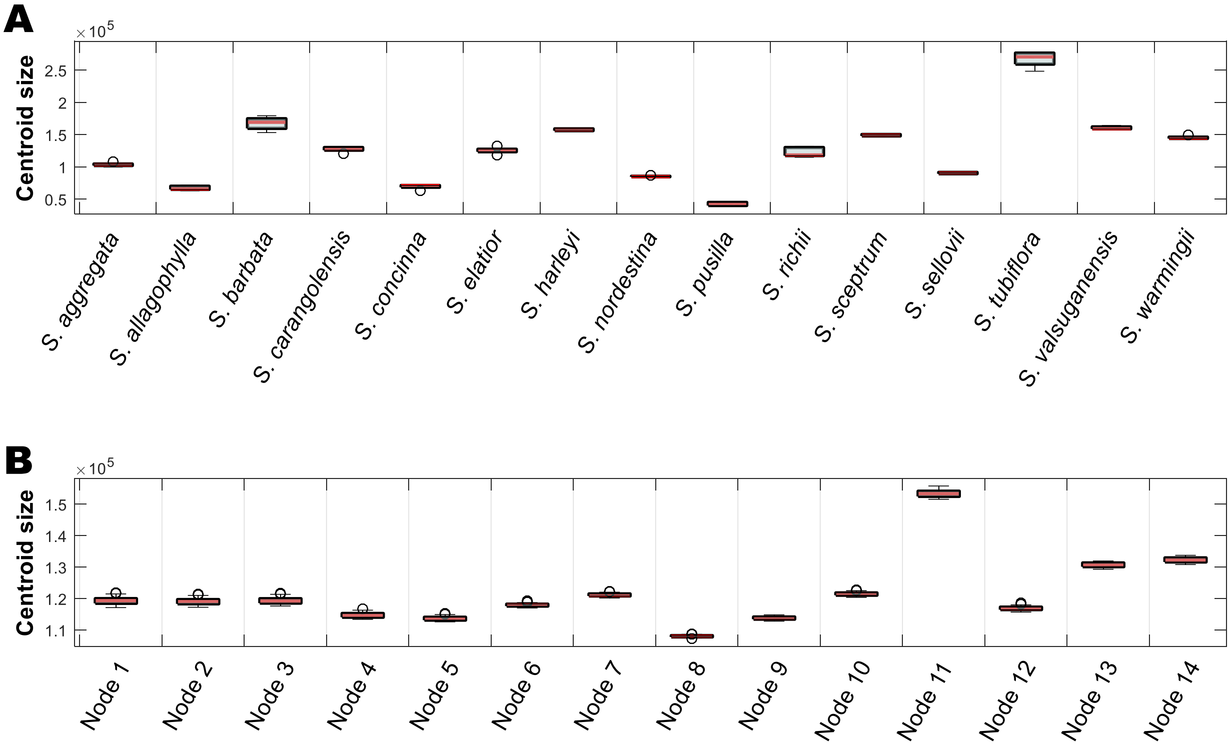


Figure 3. Centroid sizes of the corollas of the (A) extant species and (B) at the ancestral states.

## Major shape and form variations of the corollas

Major shape variations among the flowers were identified using the full-GPA GM procedure described in the Methods section. The first four shape principal components (PCs), referred to as shape PC1 (sPC1) to sPC4, accounted for 53.15%, 20.64%, 8.19%, and 5.47%, respectively, of the total variance. Figure 4A illustrates the major shape variations using virtual flowers. The virtual flower of the mean sPC values is illustrated in grey, and the virtual flowers with sPC values of mean ± 2 standard deviation (SD) are illustrated in pink.

The four sPCs were linked to four specific shape transitions. sPC1 primarily corresponded to tube curvature. The tube of the corolla with a small sPC1 value was bent upward at a considerable degree (Fig. 4A). By contrast, the tube of the corolla with a large sPC1 value was bent downward. sPC2 principally corresponded to the lobe area size. The line connecting landmarks L4–L5 separates the lobe (right) and tube (left). The corolla with a small sPC2 value had a larger lobe area than that with a large sPC2 value. Particularly, the lobe area of the corolla with a sPC2 value of mean + 2 SD was nearly absent. sPC3 particularly corresponded to tube dilation (the distance between landmarks T14 and M14). The corolla with a small sPC3 value dilated in the tube, whereas the corolla with a large sPC3 value shrank in the tube. sPC4 principally corresponded to lobe recurvation. The lobe midrib (the line connecting landmarks M27–M35) of the corolla with a small sPC4 value was bent outward. By contrast, the lobe midrib of the corolla with a large sPC4 value was almost parallel to the tube midrib (the line connecting landmarks M1–M27).

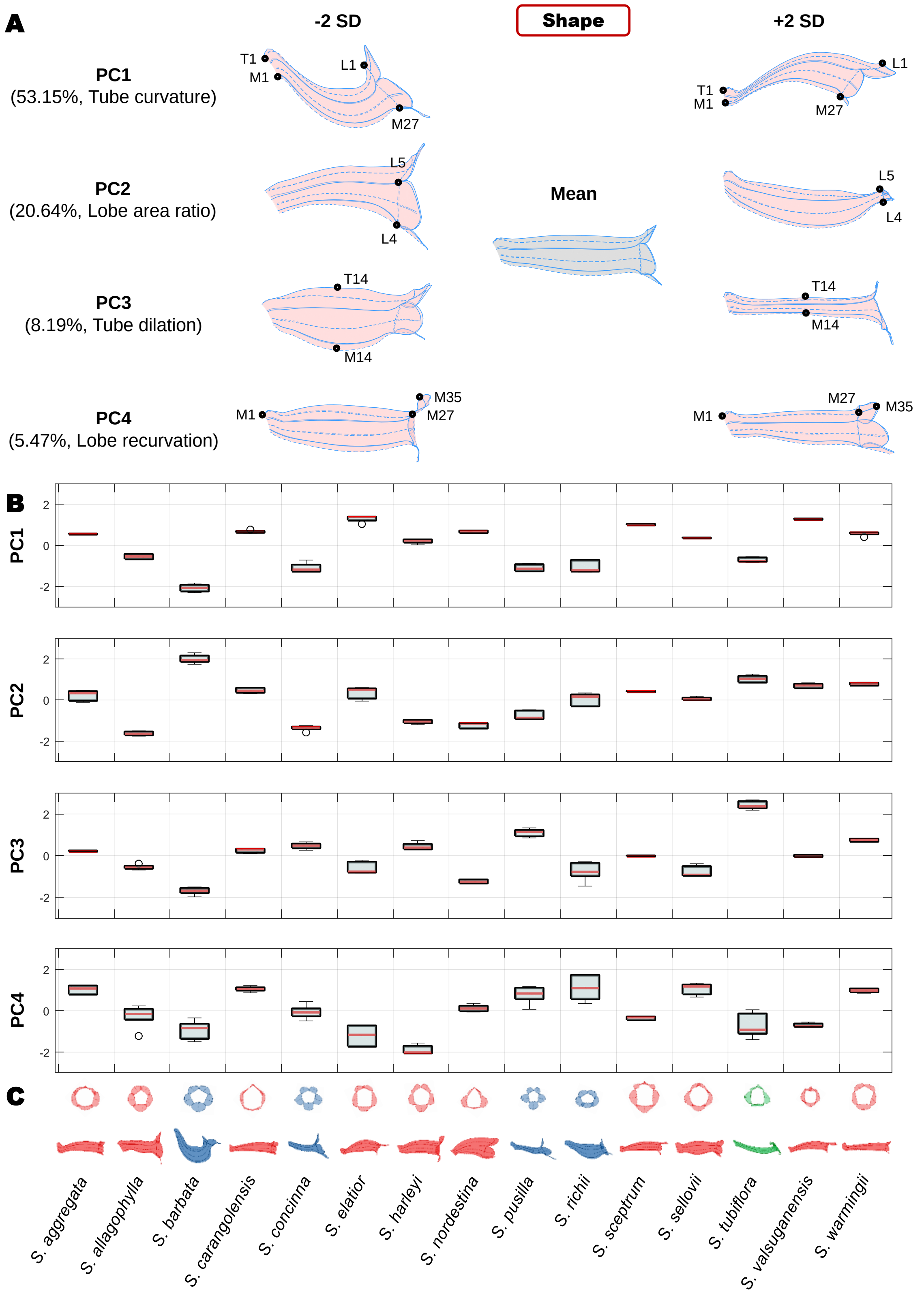


Figure 4. Major shape variations of the Corytholoma flowers: (A) virtual flowers with an sPC value of mean ± 2 SD, (B) distributions of the sPC scores, and (C) mean corolla shapes. In (A), black dots represent labeled landmarks. L, M, and T represent landmarks on lobe contour, midrib, and tube–tube rim, respectively. In (B), the sPC scores are standardized to zero mean and unit variance. In (C), the corollas are colored by pollination type.

Red, blue, and green represent hummingbird-pollinated, bee-pollinated, and moth-pollinated species, respectively.

Figures 4B and 4C illustrate the distributions of the sPC scores and the face and side views of the mean corolla shape for each species. The sPC scores were standardized to zero mean and unit variance. Note that the within-species variance of sPC scores in most species increased from sPC1 to sPC4 (Fig. S1A). Particularly, the within-species variances of species *S. allagophylla, S. elatior, S. richii,* and *S. tubiflora* in sPC4 were larger than those of the other species.

Major form variations among the flowers were identified using the partial-GPA GM procedure. The first four form PCs, referred to as form PC1–form PC4 (fPC1–fPC4), accounted for 73.69%, 18.36%, 4.03%, and 2.28%, respectively, of the total variance. Figure 5A illustrates the major form variations using virtual flowers. fPC1 primarily corresponded to the corolla size. The corolla with a small fPC1 value had a large corolla size, whereas the corolla with a large fPC1 value had a small corolla size. In fact, fPC1 was negatively correlated with centroid size ($r = -0.9952$; Fig. 6A) and accounted for 73.69% of the total form variation. Notably, fPC2, fPC3, and fPC4, respectively, correlated with sPC1, sPC2, and sPC3 ($r = 0.9576$; $r = 0.6866$; $r = 0.5027$; Fig. 6B–6D). However, *S. tubiflora* and *S. harleyi* did not follow the sPC2–fPC3 and sPC3–fPC4 correlations, respectively. The correlation coefficients increased considerably when these two species were excluded from the analyses ($r = 0.9267$ and $r = 0.8267$; Fig. 6C and 6D).

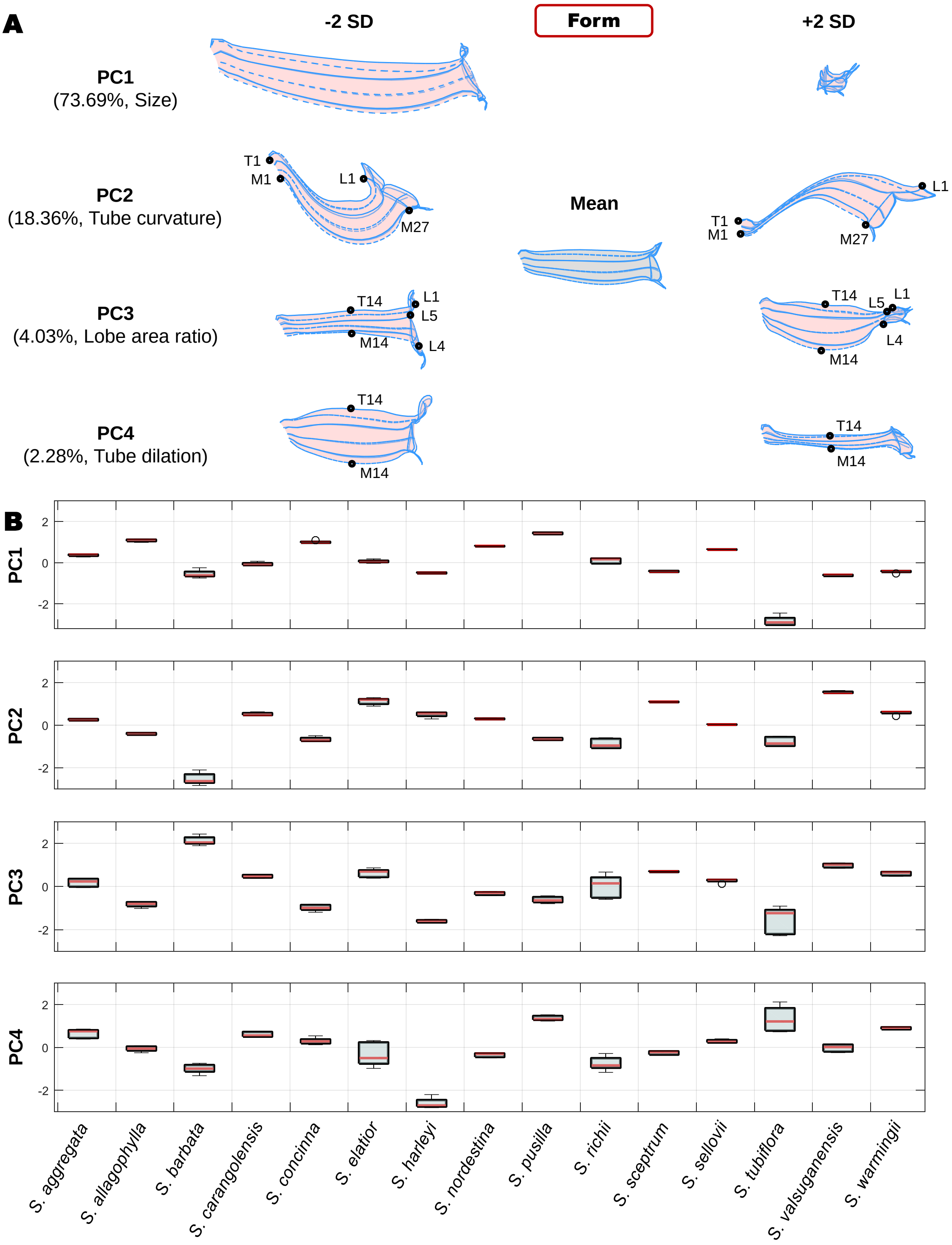


Figure 5. Form shape variations of the Corytholoma flowers: (A) virtual flowers with an fPC value of mean ± 2 SD and (B) distributions of the fPC scores. In (A), black dots represent labeled landmarks. L, M, and T represent landmarks on lobe contour, midrib, and tube–tube rim, respectively. In (B), the fPC scores are standardized to zero mean and unit variance.

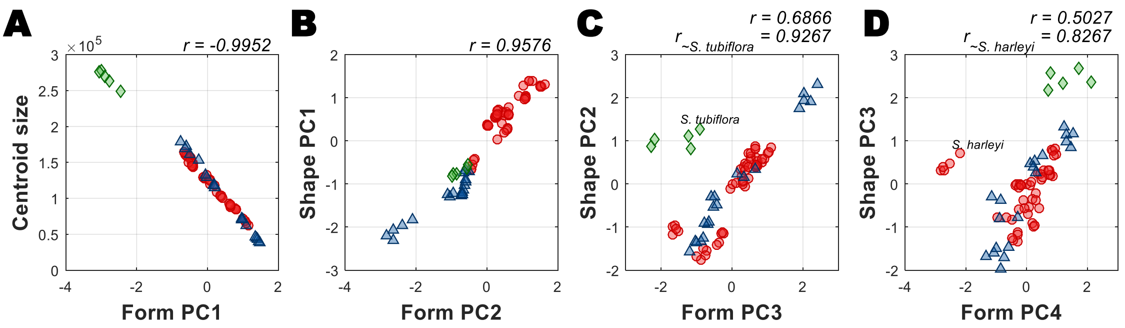


Figure 6. Analysis of correlation between the sPC and fPC scores. The PC scores are standardized to zero mean and unit variance. The correlation coefficients are provided at the upper right corners of scatter plots. Red circle, blue triangle, and green diamond represent hummingbird-pollinated, bee-pollinated, and moth-pollinated species, respectively.

## Evolutionary allometry of the corolla shape

The evolutionary allometry of the corolla shape was assessed. The correlation coefficient of 0.2157 between the shape scores and centroid sizes revealed that the centroid size of the corollas accounted for only 4.65% of the shape variation (Fig. 7A). In addition, the permutation test indicated that the correlation was weak but marginally significant (Fig. 7B, $p$ = 0.0625). Moreover, the low to medium levels of the correlations between sPCs and centroid size (Fig. S2) also supported that the allometry between shapes and size in Corytholoma was weak but significant.

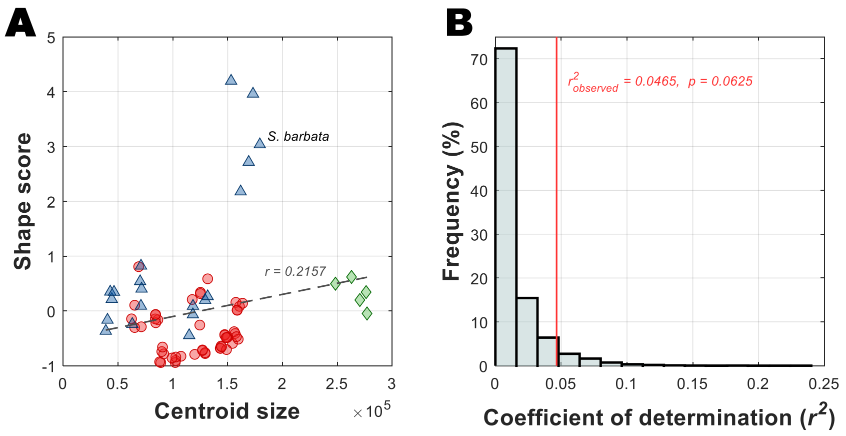

Figure 7. Evolutionary allometry of the corolla shapes in clade Corytholoma. Red circle, blue triangle, and green diamond represent hummingbird-pollinated, bee-pollinated, and moth-pollinated species, respectively.

## Morphological traits and their association with pollination type

Four morphological traits—tube curvature, lobe area ratio, tube dilation, and lobe recurvation—were defined based on the variations of the first four sPCs. The traits were subsequently quantified from the 3D images of the corollas (see the Methods section for details). Correlation analyses indicated that the defined traits adequately describe the major shape variations ($r \geq 0.7213$; Fig. 8). The pairwise correlations between the morphological traits indicated that the morphological traits were weakly correlated with each other ($r \leq 0.3501$).

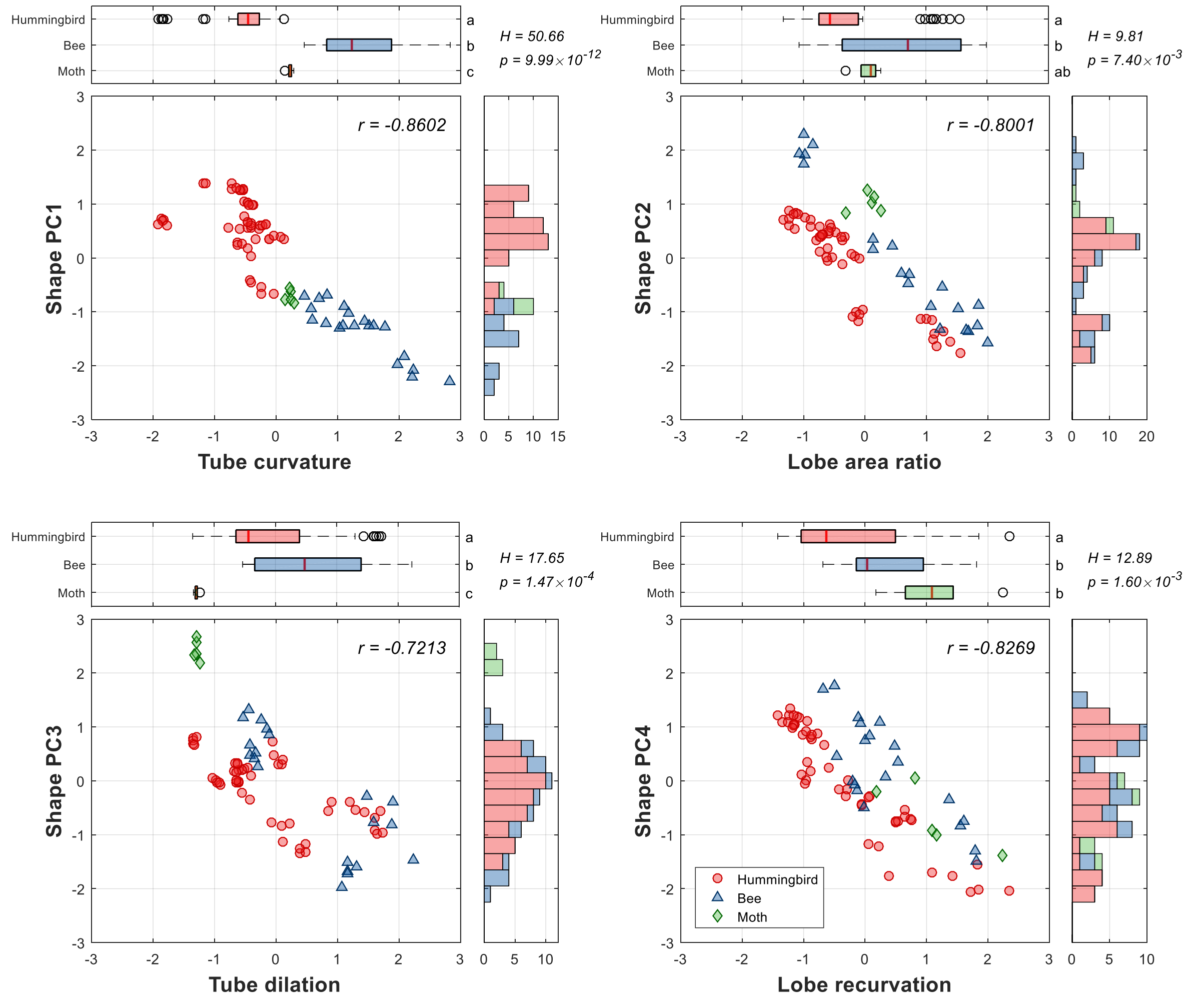


Figure 8. Scatter plots of the morphological traits and sPC scores. The trait and sPC scores are standardized to zero mean and unit variance. The correlation coefficients (*r*) are provided at the upper right corners of the scatter plots. The right panel of the boxplots presents the results of Kruskal–Wallis tests (*H* values). The lowercase alphabets at the right of the box plots denote groups of Scheffé's multiple comparison tests performed with a confidence level of 0.99.

The association between the traits and pollination types was examined. The centroid size was also included in the analyses. Kruskal–Wallis test results indicated that the four traits and centroid size significantly differed between the pollination types ($p < 7.40 \times 10^{-3}$, Table 3). Scheffé's multiple comparison test results suggested that tube curvature and tube dilation formed three clusters corresponding to the three pollination types ($p < 8.21 \times 10^{-3}$, Fig. 8, Table 3). The permutation test for logarithm of the odds (LOD) scores indicated that the

centroid size and the tube curvature was significantly associated with the three pollination types (LOD = 21.16, $p$ = 0).

Table 3. Kruskal–Wallis test results, Scheffé's multiple comparison test results, and LOD scores of the morphological traits by pollination type.

| Morphological traits | Kruskal-Wallis test | | Scheffé's multiple comparison test | | | | | | LOD score | $p$-[illegible] |
|---|---|---|---|---|---|---|---|---|---|---|
| | | | Hummingbird vs Bee | | Hummingbird vs Moth | | Bee vs Moth | | | |
| | *H*-value | *p*-value | *T*-value | *p*-value | *T*-value | *p*-value | *T*-value | *p*-value | | |
| **Centroid size** | 16.16 | $3.10\times10^{-4}$ | 2.13 | $1.11\times10^{-1}$ | 9.09 | $1.14\times10^{-12}$ | 8.48 | $1.46\times10^{-11}$ | 12.65 | [illegible] |
| **Tube curvature** | 50.66 | $9.99\times10^{-12}$ | 13.82 | $1.11\times10^{-16}$ | 3.21 | $8.21\times10^{-3}$ | 4.31 | $2.60\times10^{-4}$ | 21.16 | [illegible] |
| **Lobe area ratio** | 9.81 | $7.40\times10^{-3}$ | 3.34 | $5.64\times10^{-3}$ | 0.65 | $8.08\times10^{-1}$ | 1.15 | $5.81\times10^{-1}$ | 2.35 | 6.2[illegible] |
| **Tube dilation** | 17.65 | $1.47\times10^{-4}$ | 2.88 | $1.99\times10^{-2}$ | 2.81 | $2.36\times10^{-2}$ | 4.16 | $4.31\times10^{-4}$ | 3.86 | 1.9[illegible] |
| **Lobe recurvation** | 12.89 | $1.60\times10^{-3}$ | 2.57 | $4.20\times10^{-2}$ | 3.12 | $1.05\times10^{-2}$ | 1.56 | $3.01\times10^{-1}$ | 2.92 | 1.6[illegible] |

Phylogenetic signals of centroid size and morphological traits

The phylogenetic signals of the centroid size and four morphological traits were estimated (Fig. 9). Blomberg's K values of the tube curvature and tube dilation calculated using the 50% majority-rule consensus tree were 0.9250 and 0.8739, respectively. The permutation test for Blomberg's K values rejected the null hypothesis that the two traits had no phylogenetic signal ($p$ = 0.0427 for tube curvature and $p$ = 0.0584 for tube dilation). These observations indicated that the change in the two traits approximated the Brownian motion model, and the two traits evolved gradually through time.

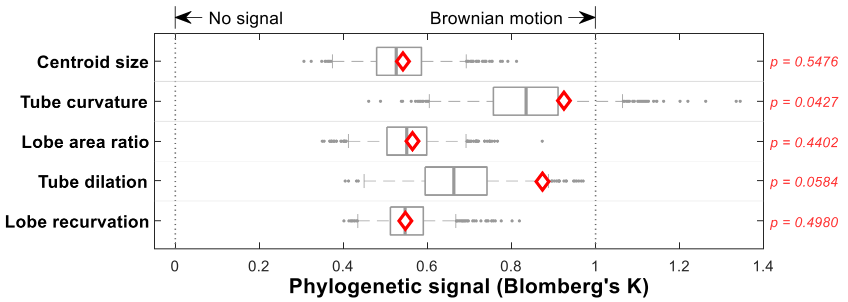


Figure 9. Phylogenetic signals of the centroid size and four morphological traits in clade Corytholoma. The distributions of the Blomberg's K values were obtained using the phylogenetic trees of 1,000 replicates in the maximum likelihood analysis. Diamonds indicate the Blomberg's K values calculated using the 50% majority-rule consensus tree. The *p* values are provided at the right of the boxplot.

## Corolla shapes and forms at the ancestral states

The corolla shapes and forms at the ancestral states were reconstructed using the 50% majority-rule consensus tree (Fig. 10). For both the reconstructed shapes and forms, the corollas at nodes 1–4 and 12 bent upward, those at nodes 5–11 became straight and narrow, and those at nodes 13 and 14 bent downward in the tube. The measurement of centroid sizes and evaluation of morphological traits revealed more details on these transitions (Table S2). Centroid sizes of the corollas fluctuated from nodes 1–7 and increased gradually from nodes 8–11. Tube curvatures of the corollas decreased from nodes 1–11. Negative tube curvatures were observed on the corollas at nodes 13 and 14. Lobe area ratios of the corollas fluctuated from nodes 1–7 and decreased gradually from nodes 8–11. Tube dilations and lobe recurvations of the corollas also decreased from nodes 1–11. The decreasing trend extended to the nested nodes 12–14. The results obtained from the reconstructed corolla also indicated that the transitions in the traits were gradual.

The pollinator types of the corolla shapes and forms at the ancestral states were estimated using sPC1–sPC4 and fPC1–fPC4, respectively, and the *k*-nearest neighbor algorithm with a *k* value of 5. The shifts in pollinator types were mostly consistent in both

shape and form. We observed that the corolla shapes and forms at nodes 1–4 were estimated to be bee-pollinated, and the corolla shapes and forms at nodes 5–9 and 13–14 were estimated to be hummingbird-pollinated (Fig. 10). The corolla shape at node 11 was estimated to be moth-pollinated (Fig. 10A). The corolla shapes at nodes 10 and 12 were estimated to be hummingbird-pollinated and bee-pollinated, respectively. The corolla forms at nodes 10 and 12 were, however, were estimated to be moth-pollinated and hummingbird-pollinated, respectively (Fig. 10B).

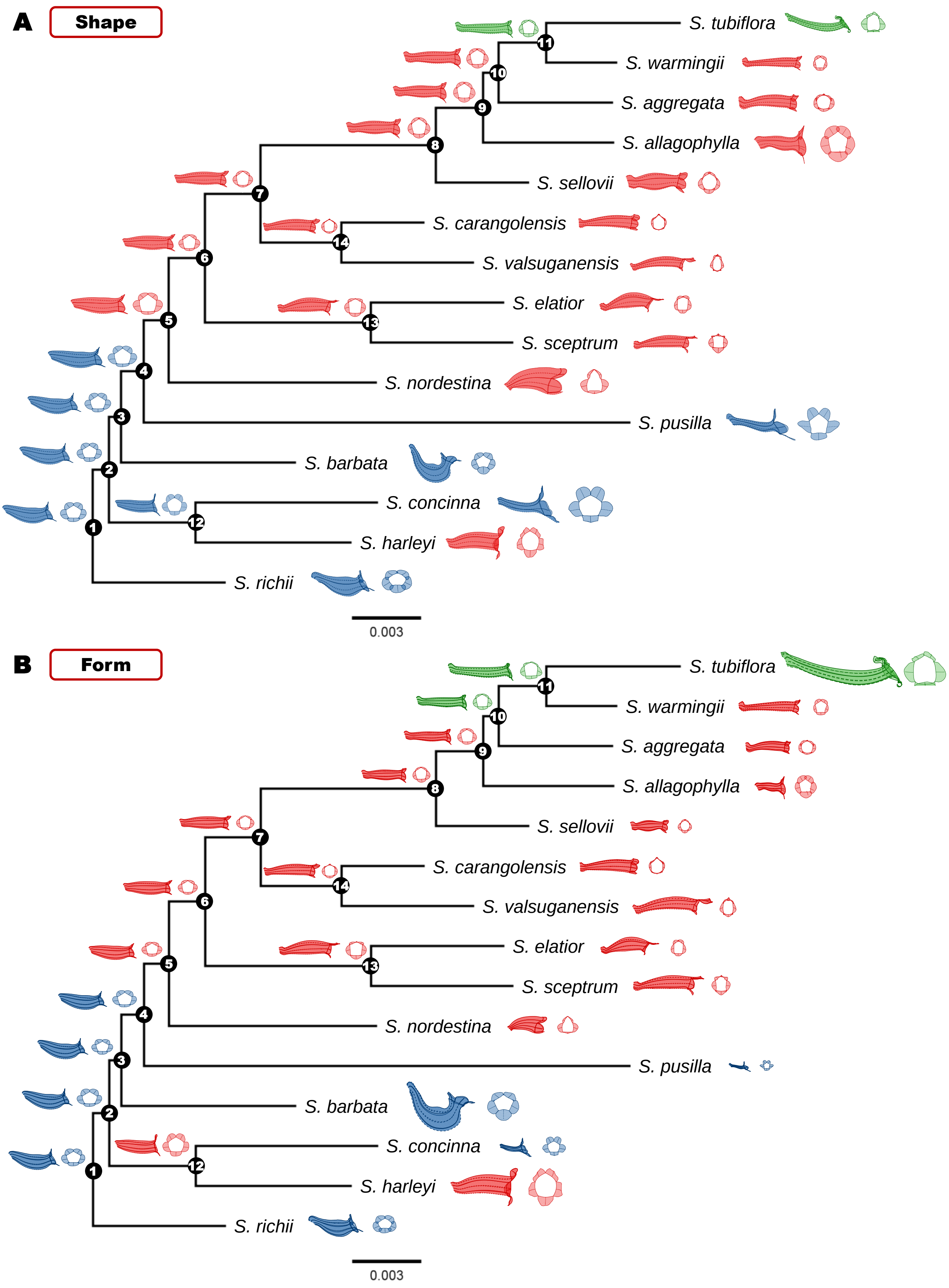


Figure 10. Reconstructed corolla (A) shapes and (B) forms at the ancestral state for the Corytholoma species. The branch length indicates the number of substitutions per site, and the scale bar denotes 0.003 substitutions

per site. The corolla colors of the extent species are assigned based on the pollination type. Red, blue, and green denotes hummingbird-, bee-, and moth-pollinated species, respectively. The corolla colors of the species at the ancestral states were estimated using the *k*-nearest neighbor algorithm with a *k* value of 5.

Figure 11 presents the distributions of the extant species and ancestral states in the corolla shape and form morphospaces. In both morphospaces, *S. barbata* was approximately 3 SDs away from the neighboring ancestral state. In the morphospace of the corolla form, *S. tubiflora* was approximately 3 SDs away from the neighboring ancestral state. Both the morphospaces were sparse in the neighborhoods of *S. barbata* and *S. tubiflora* compared with those of other species.

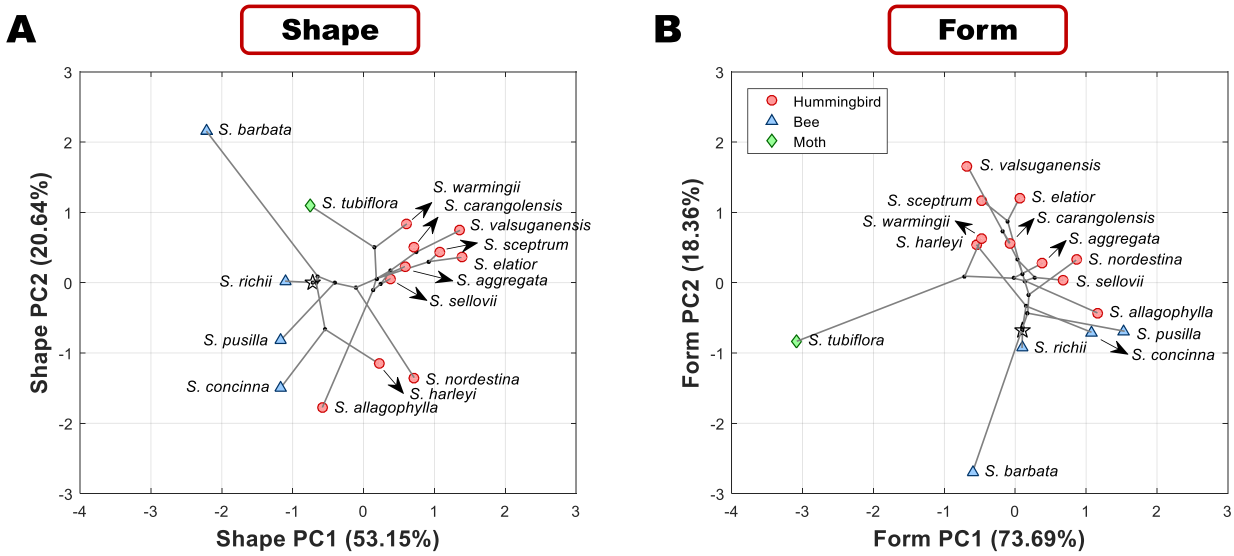


Figure 11. Distribution of the extant species and ancestral states in the morphospaces of the corolla (A) shape and (B) form. The PC scores are standardized to zero mean and unit variance. White star and black points represent the ancestral state of node 1 (root) and ancestral states of nodes 2–14, respectively.

## Discussion

In this study, we acquired the 3D corolla images of the Corytholoma species using µCT and identified the major shape and form variations of the corollas using landmark-based GM. We first revealed that the evolutionary allometry of corolla shapes was weak in Corytholoma species. According to the identified major shape variations, we defined and quantified four morphological traits—tube curvature, lobe area ratio, tube dilation, and lobe recurvation. We revealed that tube curvature and tube dilation were significantly associated with pollination type. The centroid size was also strongly associated with the pollination type. Taking together the trait values and phylogenetic information, we revealed strong phylogenetic signals in tube curvature and tube dilation. By reconstructing the corolla shapes, measuring the morphological traits at the ancestral states, and testing the phylogenetic signals of the traits, we discovered that the evolutionary changes in corolla shape were gradual in Corytholoma species.

### Resemblance of virtual flowers to corollas of clades other than clade Corytholoma

The corolla shape variations identified in Corytholoma species resembled the corolla shapes of some species from other clades. The virtual flowers of mean + 2 SD in sPC1 and mean − 2 SD in sPC2 resembled the corollas of *Vanhouttea hilariana* (clade Vanhouttea, Fig. 12A) and *Sinningia insularis* (clade Dircaea, Fig. 12B), respectively. Surprisingly, some virtual flowers also resembled the corolla shapes of species of subtribes other than subtribe Ligeriinae. The virtual flower of mean + 2 SD in sPC1 (Fig. 4A) resembled the corolla of *Columnea microphylla* (subtribe Columneinae) [52]. The virtual flower of mean − 2 SD in sPC3 (Fig. 4A) resembled the corolla of *Drymonia urceolata* (subtribe Columneinae) [53].

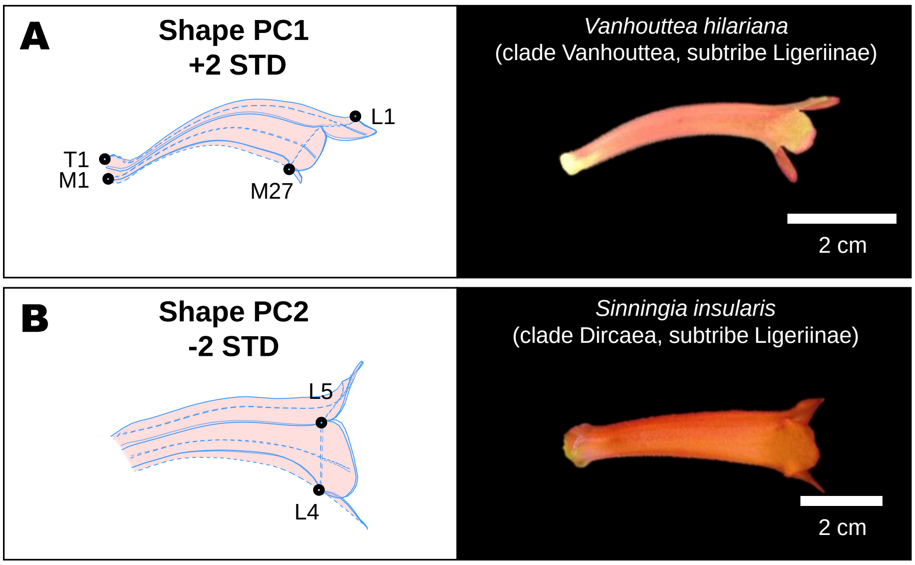


Figure 12. (A) Virtual flower of mean + 2D in sPC1 and the corolla image of *Vanhouttea hilariana* and (B) virtual flower of mean − 2D in sPC2 and the corolla image of *Sinningia insularis*.

Evolutionary allometry and morphological integration of corolla shape

The association between evolutionary allometry and morphological integration was supported by the evidence found in Corytholoma. After the emergence of GM in the last century, allometry has been believed to be associated with morphological integration [47, 54]. In Corytholoma, we revealed that the size only accounted for 4.65% of shape variation, suggesting that the allometry of corolla shape was weak. The mean of squared pairwise correlation coefficients between the four morphological traits was 0.0521, which suggested that the morphological integration in corolla shape was weak. Similar to the findings in a study of skulls of birds [55], the aforementioned evidence indicated that evolutionary allometry and morphological integration are associated at a certain level. The present study suggests that the corolla shape in Corytholoma can serve as an example for the association between evolutionary allometry and morphological integration.

### Divergent corolla dominated the analysis of shape and form variation

Virtual flowers should be cautiously used to interpret the major shape and form variations of the corollas when species with extreme shapes or forms are included or when the species are sparse in the morphospace. The corolla shape and form of the Corytholoma species varied largely. In some studies on closely related species, the ranges of the PC scores were usually less than 3 SDs [10, 25]. By contrast, the PC scores in the present study spanned up to 5 SDs (Fig. 10). In the morphospace, *S. tubiflora* and *S. barbata* were distant from the clusters of the other species. Moreover, the space among *S. tubiflora*, *S. barbata*, and the cluster of the other species was large. The GM analysis reveals the major shape and form variations by applying linear interpolation or extrapolation to the landmarks of the species being studied (reviewed in [47]). Thus, the identified variation in the analysis could be considerably influenced by *S. tubiflora* and *S. barbata*. In other words, the divergent corolla shape of *S. tubiflora* and *S. barbata* would deviate the interpolated or extrapolated virtual flowers from reality. The most obvious example was the virtual flower of mean + 2 SD in fPC1 (Fig. 5A). The proximal part of the corolla protruded from the throat of the tube such that the corolla was inside out. Mean + 2D in fPC1 was beyond the cluster of the species in Figure 10B. No corolla physically existing in nature can resemble such a virtual flower.

### Use of GM-derived morphological traits for phenotyping

Defining appropriate traits that correspond to shape variations is crucial for phenotyping. Conventionally, traits were defined based on researchers’ observations and were quantified manually using calipers. After the emergence of GM and imaging techniques, some studies [23, 30, 37] have used PC scores from the GM analysis as the traits for phenotyping. Although comprehensive, PC scores were so complex that they could not specify the key changes in shape. We defined morphological traits by observing the major shape variations

identified in the GM analysis. Subsequently, the traits were automatically measured from the 3D landmarks of the corollas. The proposed approach helped us objectively define and precisely quantify traits corresponding to major shape variations.

Limitations of 3D analysis of corolla shape evolution

Although we believe that this is one of the pioneer studies to integrate the phylogenetic and 3D information to speculate corolla shape evolution, the results may be affected by phylogenetic uncertainty [56]. Phylogenetic uncertainty is majorly increased due to two issues: inconsistent tree topologies or branch lengths and incomplete sampling of extant species. We used the 1,000 phylogenetic trees obtained from each replicates for evaluating the phylogenetic signals. Therefore, the bias caused by relying on a single phylogenetic tree was avoided. The flower specimens of two species, *S. brasiliensis* and *S. aghensis*, with unique corolla shapes were not included in this study. In addition, the molecular sequences of newly added Corytholoma species [57], *S. helioana* and *S. muscicola*, were partly published. The topology of the phylogeny is data-dependent and could have been altered if these species were included. However, inclusion of these specimens and sequences would have made yielded more complete results of the analysis of corolla shape evolution.

## Methods

Landmark identification

Landmarks were selected semi-automatically from the 3D surface images to describe the corolla shapes. See Wang et al. (2015) [8] and Hsu et al. (2017) [11] for the details of the landmark selection procedure. In this study, the landmarks along the lobe–tube rims (line connecting landmarks I, IV, and II in Fig. 13) were added to identify the lobe (yellow) and tube (pink) areas of the petal. The primary landmarks (roman letters in Fig. 13) and identification of lobe contours (line connecting landmarks I, III and II), lobe midribs (line connecting landmarks III and IV), tube midribs (line connecting landmarks IV and VI), lobe–tube rims, and tube–tube rims (line connecting landmarks I and V, and line connecting landmarks II and VII) were selected using Landmark software [58]. The secondary landmarks, equally distributed points between two primary landmarks, were obtained using a program developed in MATLAB (The MathWorks, Natick, MA, USA) [8]. On each petal, 17, 9, 9, 27, and 27 landmarks were observed on the lobe contour, lobe–lobe rim, lobe midrib, tube midrib, and tube–tube rim, respectively. A total of 415 landmarks, including 25 primary and 390 secondary, were collected for each specimen. The landmark data of the specimens in this study are available in the GigaScience Database repository (GigaDB number).

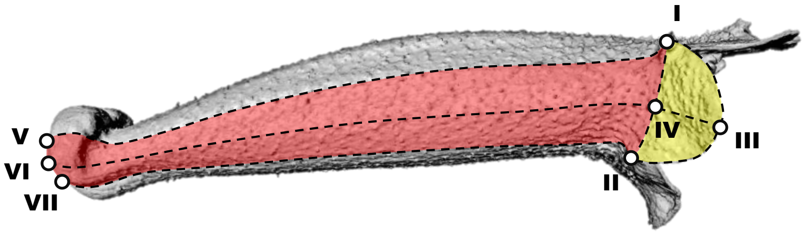


Figure 13. Illustration of primary landmarks (roman letters), lobe contour, lobe–tube rim, lobe midrib, tube midrib, and tube–tube rims. The primary landmarks were defined as the intersections of adjacent lobes (I and II), the distal and proximal points of petal midribs (III and VI), the intersection of the lobe–tube rims and midribs (IV), and the proximal points of the tube–tube rims (V and VII). The secondary landmarks are equally

distributed points along the lobe contour (line connecting I, III, and II), lobe–tube rim (line connecting I, IV, and II), lobe midrib (line connecting III and IV), tube midrib (line connecting IV and VI), and tube–tube rim (line connecting I and V, and line connecting II and VII), respectively, between two primary landmarks.

Major shape and form variations of the corollas

The major shape and form variations of corollas were identified from the landmarks obtained using GM. The major shape variations were determined using full-GPA [59]. Full-GPA removed the geometric information of the corollas related to translation, rotation, and scaling. The major form variations, defined as the combination of shape and size variations, were determined using partial-GPA. Partial-GPA removed the information of the corollas related to translation and rotation only. Following full- or partial- GPA, PCA was applied to the resulting landmarks. The obtained PCs were referred to as sPCs and fPCs. The first four sPCs and fPCs accounted for the majority of the variance and were used for representing the major shape and form variations between the corollas, respectively. Virtual flowers were created to visualize the major shape and form variations. The virtual flowers were generated by manipulating landmarks and by applying inverse PCA to the manipulated landmarks. See Wang et al. (2015) [8] for the details of the visualization of the shape and form variations.

Evolutionary allometry of the corolla shapes

The evolutionary allometry of the corolla shapes in Corytholoma was evaluated using a multivariate regression analysis [60], correlation analysis, and permutation test [63]. In the multivariate regression analysis, regression coefficients were estimated using the full-GPA landmarks as the response variables and the centroid size as the predictor variable [60]. A shape score of the specimen was then obtained as the inner product of the full-GPA landmarks of the specimen and the vector of the regression coefficients. Subsequently, the correlation between the shape scores and centroid sizes was calculated. The square of the

correlation coefficient indicated the degree of the size variation that accounted for the shape variation. Subsequently, the permutation test was performed to evaluate the dependency of the shape score on the centroid size. In the permutation test, the pairs of full-GPA landmarks and their associated centroid size were reshuffled among all the specimens 10,000 times. In each shuffle, the aforementioned multivariate regression analysis and correlation analysis were performed using the shuffled data to obtain a correlation coefficient. The accumulated squares of the correlation coefficients obtained from the 10,000 shuffles formed the null distribution of the permutation test. The $p$ value of the test was calculated as the proportion of the null distribution larger than the square of the correlation coefficient calculated using the unshuffled data. The $p$ value presented the level of the dependence of the shape score on the centroid size.

Quantification of the morphological traits

Morphological traits were defined by observing the variations of the first four sPCs and were directly quantified using the 3D corolla image. The traits included tube curvature, lobe area ratio, tube dilation, and lobe recurvation (Fig. 14). Tube curvature was defined as the second-order coefficient of the quadratic equation fitted to tube axis (dotted line in Fig. 14A). Tube axis was formed as the collection of the centroid points of the landmarks on the tube–tube rims and tube midribs that have the same order from the proximal part of the corolla. The centroid points were mapped to the sagittal plane of the tube (solid line in the parallelogram in Fig. 14A) before they were used for curve fitting. Lobe area ratio was defined as the ratio of lobe area (red area in Fig. 14B) to corolla surface area. The areas were calculated as the sizes of the triangle meshes connecting the landmarks surrounding the object. Tube dilation was defined as the ratio of the centroid size of center tube transection (the 14th landmarks from the proximal part of the corolla; hollow dots in Fig. 14C) to the

length of tube axis. The centroid size [61] of center tube transection was defined as the root sum squared distance between the landmarks on the tube–tube rims or tube midribs to their centroid (solid dot in Fig. 14C). Lobe recurvation was defined as the mean of lobe bending angles of the five petals. The lobe bending angle for a petal ($\theta$ in Fig. 14D) was defined as the angle between the normal vector of the tube-opening plane (red area in Fig. 14D) and lobe-bending line (red line in Fig. 14D) of the petal. Tube-opening plane was defined as the plane optimally fitting the landmarks on the lobe–tube rim. The lobe-bending line of a petal was defined as the line connecting the proximal and distal landmarks on the lobe midrib.

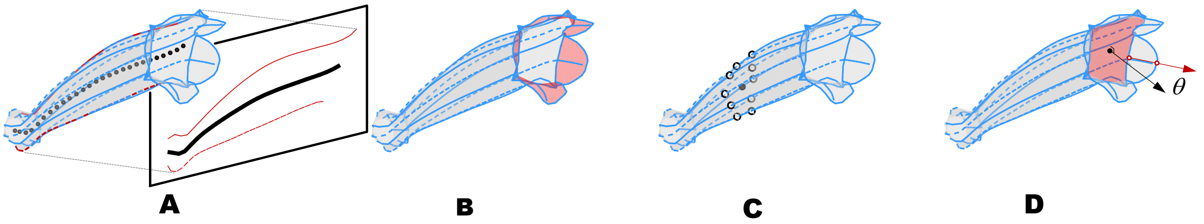


Figure 14. Illustration of the corolla shape traits: (A) tube curvature, (B) lobe area ratio, (C) tube dilation, and (D) lobe recurvation. In (A), the dotted line indicates the tube axis, the parallelogram indicates the sagittal plane of the corolla, and the solid line indicates the mapping of the tube axis to the sagittal plane. In (B), the red area indicates the lobe of the corolla. In (C), the hollow dots indicate landmarks on center tube transection, and the solid dot indicates the centroid of the landmarks. In (D), the red area indicates the tube-opening plane.

Association between the morphological traits and pollination type

The association between the morphological traits and pollination type and the level of the association were evaluated using LOD scores [62] and permutation tests, respectively. To calculate the LOD score for a trait, the ratio of the squared deviation of the trait to the sum of the within-group squared deviations of the trait was first calculated. The groups referred to pollinator types. The LOD score of the trait was then obtained as the logarithm of the ratio. A large LOD score indicates a strong association. Next, permutation tests were conducted to evaluate the levels of the association between the traits and pollination type. In a permutation

test, pairs comprising a morphological trait and its pollination type were reshuffled among all the specimens 50,000 times. In each shuffle, an LOD score was calculated using the aforementioned procedure and shuffled data. The cumulative LOD scores of the 50,000 shuffles formed the null distribution of the permutation test. The *p* value for the test was subsequently calculated as the frequency of the null distribution higher than the LOD score calculated using the unshuffled data. The *p* value presented the level of association between the morphological traits and pollination type.

Phylogenetic analysis

The phylogeny of the Corytholoma species was obtained using a maximum likelihood (ML) analysis. In the procedure, the sequences of six molecular markers of the Corytholoma species were gathered from published data ([3]; Table S1). The sequences were aligned using a program, MAFFT [64], without manual adjustment. The alignments of the markers were then concatenated to obtain 4,414 sites. Subsequently, the combination of the HKY85 model, the estimated proportion of invariant sites (+I) and the variable site following a gamma distribution (+gamma) was suggested by Modeltest 3.7 software [66], which is the best-fit nucleotide substitution model for the aligned sequence. The ML analysis was then performed using the aligned sequence, the aforementioned model and parameters suggested by Modeltest 3.7, and GARLI 2.0 software [65] for 1,000 replicates. The 50% majority-rule consensus tree of the 1,000 replicates was then used as the representative phylogeny of the Corytholoma species for the following analyses.

Tests of phylogenetic signals

The phylogenetic signals of the centroid size and morphological traits were evaluated using Blomberg’s K values [67] and permutation tests. Blomberg’s K value for a trait was

calculated using the mean trait values of all the species, the phylogenetic trees of 1,000 replicates from the ML analysis, and the "phylosig" function in the R package phytools [68]. A K value of zero indicates no phylogenetic signal in the trait, whereas a K value of one indicates a strong phylogenetic signal; the trait evolution follows the Brownian motion model. The permutation tests were then performed to evaluate if the K values significantly differed from zero. In a permutation test, the pairs of the species positions on the phylogenetic tree and the mean trait value were reshuffled 1,000 times. In each shuffle, the K value was calculated using the 50% majority-rule consensus tree. The cumulative K values of the 1,000 shuffles formed the null distribution of the permutation test. The *p* value for the test was then calculated as the proportion of the null distribution larger than the K value calculated using the unshuffled data. The *p* value presented the level that the K value differed from zero.

Reconstruction of the corolla shapes and forms at the ancestral states

Corolla shapes and forms were reconstructed at the ancestral states in the phylogeny using weighted squared-change parsimony [69], the 3D landmarks of mean corolla shapes and forms of all the extant species, and the 50% majority-rule consensus tree. After reconstruction, the corolla forms were used for quantifying the centroid sizes and morphological traits at the ancestral states. To further assess the variation in the corolla shape and form at each ancestral state, the reconstruction was repeated 100 times. In each repetition, the mean corolla shape or form was calculated from three specimens that were randomly selected from the five specimens of each species. The variation of the 100 reconstructed corolla shapes and forms was presented.

**Funding**

This research was supported by NSC-101-2313-B-002-050-MY3 from National Science Council (Ministry of Science and Technology) of Taiwan.

**Acknowledgements**

We thank the National Laboratory Animal Center for the technical support on the μCT scanning and Mr. Chun-Ming Chen at the Dr. Cecilia Koo Botanic Conservation and Environmental Protection Center for providing and maintaining the plant materials.

**References**

1. Walcher-Chevillet CL, Kramer EM. Breaking the mold: understanding the evolution and development of lateral organs in diverse plant models. Curr Opin Genet Dev. 2016;39:79-84. doi:10.1016/j.gde.2016.06.005

2. Moyroud E, Glover BJ. The evolution of diverse floral morphologies. Curr Biol. 2017;27(17):R941-R951. doi:10.1016/j.cub.2017.06.053

3. Perret M, Chautems A, Spichiger R, Kite G, Savolainen V. Systematics and evolution of tribe Sinningieae (Gesneriaceae): evidence from phylogenetic analyses of six plastid DNA regions and nuclear ncpGS. Am J Bot. 2003;90(3):445-460. doi:10.3732/ajb.90.3.445

4. Lawing AM, Polly PD. Geometric morphometrics: recent applications to the study of evolution and development. J Zool. 2010;280(1):1-7. doi:10.1111/j.1469-7998.2009.00620.x

5. Zelditch ML, Swiderski DL, Sheets HD. Geometric morphometrics for biologists: a

primer. 2nd ed. Cambridge, MA: Academic Press; 2012.

6. Gower JC. Generalized procrustes analysis. Psychometrika. 1975;40(1):33-51.

7. Rohlf FJ, Slice D. Extensions of the Procrustes method for the optimal superimposition of landmarks. Syst Biol. 1990;39(1):40-59. doi:10.2307/2992207

8. Wang CN, Hsu HC, Wang CC, Lee TK, Kuo YF. Quantifying floral shape variation in 3D using microcomputed tomography: a case study of a hybrid line between actinomorphic and zygomorphic flowers. Front Plant Sci. 2015;6:724. doi:10.3389/fpls.2015.00724

9. Adams DC, Rohlf FJ, Slice DE. A field comes of age: geometric morphometrics in the 21st century. Hystrix. 2013;24(1):7. doi:10.4404/hystrix-24.1-6283

10. van der Niet T, Zollikofer CP, de León MSP, Johnson SD, Linder HP. Three-dimensional geometric morphometrics for studying floral shape variation. Trends Plant Sci. 2010;15(8):423-426. doi:10.1016/j.tplants.2010.05.005

11. Hsu HC, Wang CN, Liang CH, Wang CC, Kuo YF. Association between petal form variation and CYC2-like genotype in a hybrid line of Sinningia speciosa. Front Plant Sci. 2017;8:558. doi:10.3389/fpls.2017.00558

12. Hsu HC, Chen CY, Lee TK, Weng LK, Yeh DM, Lin TT., et al. Quantitative analysis of floral symmetry and tube dilation in an F2 cross of Sinningia speciosa. Sci Hort. 2015;188:71-77. doi:10.1016/j.scienta.2015.03.0

13. Alexandre H, Vrignaud J, Mangin B, Joly S. Genetic architecture of pollination syndrome

transition between hummingbird-specialist and generalist species in the genus Rhytidophyllum (Gesneriaceae). PeerJ; 2015;3:e1028. doi:10.7717/peerj.1028

14. Joly S, Lambert F, Alexandre H, Clavel J, Léveillé- Bourret É, Clark JL. Greater pollination generalization is not associated with reduced constraints on corolla shape in Antillean plants. Evolution. 2018;72(2):244-260. doi:10.1111/evo.13410

15. Strelin MM, Benitez-Vieyra S, Ackermann M, Cocucci AA. Flower reshaping in the transition to hummingbird pollination in Loasaceae subfam. Loasoideae despite absence of corolla tubes or spurs. Evol Ecol. 2016;30(3):401-417.

16. Strelin MM, Benitez- Vieyra S, Fornoni J, Klingenberg CP, Cocucci A. The evolution of floral ontogenetic allometry in the Andean genus Caiophora (Loasaceae, subfam. Loasoideae). Evol Dev. 2018;20(1):29-39. doi:10.1111/ede.12246

17. Blanco- Pastor JL, Ornosa C, Romero D, Liberal IM, Gómez JM, Vargas P. Bees explain floral variation in a recent radiation of Linaria. J Evolution Biol. 2015;28(4):851-863. doi:10.1111/jeb.12609

18. Fernández-Mazuecos M, Blanco-Pastor JL, Gómez JM, Vargas P. Corolla morphology influences diversification rates in bifid toadflaxes (Linaria sect. Versicolores). Ann Bot. 2013;112(9):1705-1722. doi:10.1093/aob/mct214

19. Hernández-Ramírez AM, Aké-Castillo JA. A geometric morphometrics study of stigma-anther polymorphism in the tropical distylous Palicourea padifolia (Rubiaceae). Am J

Plant Sci. 2014;5(10):1449. doi:10.4236/ajps.2014.510160
20. Carleial S, Van Kleunen M, Stift M. Small reductions in corolla size and pollen: ovule ratio, but no changes in flower shape in selfing populations of the North American Arabidopsis lyrata. Oecologia. 2017;183(2):401-413. doi: 10.1007/s00442-016-3773-4
21. Gómez JM, Perfectti F, Camacho JPM. Natural selection on Erysimum mediohispanicum flower shape: insights into the evolution of zygomorphy. Am Nat. 2006;168(4):531-545. doi:10.1086/507048
22. Gómez JM, Bosch J, Perfectti F, Fernández JD, Abdelaziz M, Camacho JPM. Spatial variation in selection on corolla shape in a generalist plant is promoted by the preference patterns of its local pollinators. Proc R Soc B. 2008;275(1648):2241-2249. doi:10.1098/rspb.2008.0512
23. Gómez JM, Abdelaziz M, Muñoz-  Pajares J, Perfectti F. Heritability and genetic correlation of corolla shape and size in Erysimum mediohispanicum. Evolution. 2009;63(7):1820-1831. doi:10.1111/j.1558-5646.2009.00667.x
24. Savriama Y, Gómez JM, Perfectti F, Klingenberg CP. Geometric morphometrics of corolla shape: dissecting components of symmetric and asymmetric variation in Erysimum mediohispanicum (Brassicaceae). New Phytol. 2012;196(3):945-954. doi:10.1111/j.1469-8137.2012.04312.x
25. Gómez JM, Perfectti F, Lorite J. The role of pollinators in floral diversification in a clade

of generalist flowers. Evolution. 2015;69(4):863-878.

26. Gómez JM, Torices R, Lorite J, Klingenberg CP, Perfectti F. The role of pollinators in the evolution of corolla shape variation, disparity and integration in a highly diversified plant family with a conserved floral bauplan. Ann Bot. 2016;117(5):889-904. doi:10.1093/aob/mcv194

27. Gardner AG, Gerald JNF, Menz J, Shepherd KA, Howarth DG, Jabaily RS. Characterizing floral symmetry in the Core Goodeniaceae with geometric morphometrics. PLoS One. 2016;11(5):e0154736. doi:10.1371/journal.pone.0154736

28. Shipunov AB, Bateman RM. Geometric morphometrics as a tool for understanding Dactylorhiza (Orchidaceae) diversity in European Russia. Biol J Linn Soc. 2005;85(1):1-12. doi:10.1111/j.1095-8312.2005.00468.x

29. Baranzelli MC, Johnson LA, Cosacov A, Sérsic AN. Historical and ecological divergence among populations of Monttea chilensis (Plantaginaceae), an endemic endangered shrub bordering the Atacama Desert, Chile. Evol Ecol. 2014;28(4):751-774. doi:10.1007/s10682-014-9694-y

30. Kaczorowski RL, Seliger AR, Gaskett AC, Wigsten SK, Raguso RA. Corolla shape vs. size in flower choice by a nocturnal hawkmoth pollinator. Funct Ecol. 2012;26(3):577-587. doi:10.1111/j.1365-2435.2012.01982.x

31. Berger BA, Ricigliano VA, Savriama Y, Lim A, Thompson V, Howarth DG. Geometric

morphometrics reveals shifts in flower shape symmetry and size following gene knockdown of CYCLOIDEA and ANTHOCYANIDIN SYNTHASE. BMC plant biol. 2017;17(1):205. doi:10.1186/s12870-017-1152-x

32. Sinjushin AA, Bagheri A, Maassoumi AA, Rahiminejad MR. Terata of two legume species with radialized corolla: some correlations in floral symmetry. Plant Syst. Evol. 2015;301(10):2387-2397. doi:10.1007/s00606-015-1235-9

33. Püschel TA, Espejo J, Sanzana MJ, Benítez HA. Analysing the floral elements of the lost tree of Easter Island: a morphometric comparison between the remaining ex-situ lines of the endemic extinct species Sophora toromiro. PloS One. 2014;9(12):e115548. doi:10.1371/journal.pone.0115548

34. Radović S, Urošević A, Hočevar K, Vuleta A, Manitašević Jovanović S, Tucić B. Geometric morphometrics of functionally distinct floral organs in Iris pumila: Analyzing patterns of symmetric and asymmetric shape variations. Arch Biol Sci. 2017;69(2):223-231. doi:10.2298/ABS160912086R

35. Tucić B, Budečević S, Manitašević Jovanović S, Vuleta A, Klingenberg CP. Phenotypic plasticity in response to environmental heterogeneity contributes to fluctuating asymmetry in plants: first empirical evidence. J Evolution Biol. 2018;31(2):197-210. doi:10.1111/jeb.13207

36. Dalayap RM, Torres MAJ, Demayo CG. Landmark and outline methods in describing

petal, sepal and labellum shapes of the flower of Mokara orchid varieties. Int J Agric Biol. 2011;13:652-658. doi:11-106/AWB/2011/13-5-652-658

37. Feng X, Wilson Y, Bowers J, Kennaway R, Bangham A, Hannah A, et al. Evolution of allometry in Antirrhinum. Plant Cell. 2009;21(10):2999-3007. doi:10.1105/tpc.109.069054

38. Cui ML, Copsey L, Green AA, Bangham JA, Coen E. Quantitative control of organ shape by combinatorial gene activity. PLoS Biol. 2010;8(11):e1000538. doi:10.1371/journal.pbio.1000538

39. Gould SJ. Allometry and size in ontogeny and phylogeny. Biol Rev. 1966;41(4):587-638.

40. Niklas KJ. Plant allometry: the scaling of form and process. Chicago and London: University of Chicago Press; 1994.

41. Stebbins GL. Adaptive radiation of reproductive characteristics in angiosperms, I: pollination mechanisms. Annu Rev Ecol Syst. 1970;1(1):307-326.

42. Fenster CB, Armbruster WS, Wilson P, Dudash MR, Thomson JD. Pollination syndromes and floral specialization. Annu Rev Ecol Evol Syst. 2004;35:375-403. doi:10.1146/annurev.ecolsys.34.011802.132347

43. Schulte LJ, Clark JL, Novak SJ, Jeffries SK, Smith JF. Speciation within Columnea section angustiflora (Gesneriaceae): islands, pollinators and climate. Mol Phylogenet Evol. 2015;84:125-144. doi:10.1016/j.ympev.2014.12.008

44. Ramírez-Aguirre E, Martén-Rodríguez S, Ornelas JF. Floral variation, nectar production,

and reproductive success of two Drymonia (Gesneriaceae) species with mixed pollination syndromes. Int J Plant Sci. 2016;177(6):469-480. doi:10.1086/686584

45. Ling SJ, Meng QW, Tang L, Ren MX. Pollination syndromes of Chinese Gesneriaceae: a comparative study between Hainan Island and neighboring regions. Bot Rev. 2017;83(1):59-73. doi:10.1007/s12229-017-9181-6

46. Claude J. Morphometrics with R. New York: Springer Science & Business Media; 2008.

47. Klingenberg CP. Evolution and development of shape: integrating quantitative approaches. Nat Rev Genet. 2010;11(9):623. doi:10.1038/nrg2829

48. Klingenberg CP. Size, shape, and form: concepts of allometry in geometric morphometrics. Dev Genes Evol. 2016;226(3):113-137. doi:10.1007/s00427-016-0539-2

49. Klingenberg CP, Gidaszewski NA. Testing and quantifying phylogenetic signals and homoplasy in morphometric data. Syst Biol. 2010;59(3):245-261. doi:10.1093/sysbio/syp106

50. Chartier M, Jabbour F, Gerber S, Mitteroecker P, Sauquet H, von Balthazar M, et al. The floral morphospace–a modern comparative approach to study angiosperm evolution. New Phytol. 2014;204(4):841-853. doi:10.1111/nph.12969

51. Palci A, Lee MS. Geometric morphometrics, homology and cladistics: review and recommendations. Cladistics. 2018;1-13. doi:10.1111/cla.12340

52. Smith JF, Ooi MT, Schulte L, Amaya-Márquez M, Pritchard R, Clark JL. Searching for

monophyly in the subgeneric classification systems of Columnea (Gesneriaceae). Selbyana. 2013;126-142.

53. Clark JL, Clavijo L, Muchhala N. Convergence of anti-bee pollination mechanisms in the Neotropical plant genus Drymonia (Gesneriaceae). Evol Ecol 2015;29(3):355-377.

54. Klingenberg CP. Morphological integration and developmental modularity. Annu Rev Ecol Evol Syst 2008;39:115-132. doi: 0.1146/annurev.ecolsys.37.091305.110054

55. Klingenberg CP, Marugán-Lobón J. Evolutionary covariation in geometric morphometric data: analyzing integration, modularity, and allometry in a phylogenetic context. Syst Biol 2013;62(4):591-610.

56. Rangel TF, Colwell RK, Graves GR, Fučíková K, Rahbek C, Diniz- Filho JAF. Phylogenetic uncertainty revisited: Implications for ecological analyses. Evolution. 2015;69(5):1301-1312. doi:10.1111/evo.12644

57. Chautems A, Lopes TCC, Peixoto M, Rossini J. Taxonomic revision of Sinningia Nees (Gesneriaceae) IV: six new species from Brazil and a long overlooked taxon. Candollea. 2010;65(2):241-267.

58. Wiley DF, Amenta N, Alcantara DA, Ghosh D, Kil YJ, Delson E, et al. Evolutionary morphing. In VIS 05. IEEE Visualization, 2005. (pp. 431-438). IEEE.

59. Rohlf FJ, Slice D. Extensions of the Procrustes method for the optimal superimposition of landmarks. Syst Biol. 1990;39(1):40-59.

60. Monteiro LR. Multivariate regression models and geometric morphometrics: the search for causal factors in the analysis of shape. Syst Biol. 1999;48(1):192-199.

61. Mitteroecker P, Gunz P. Advances in geometric morphometrics. Evol Biol. 2009;36(2):235-247.

62. Morton NE. Sequential tests for the detection of linkage. Am J Hum Genet. 1955;7(3):277.

63. Churchill GA, Doerge RW. Empirical threshold values for quantitative trait mapping. Genetics. 1994;138(3):963-971.

64. Katoh K, Standley DM. MAFFT multiple sequence alignment software version 7: improvements in performance and usability. Mol biol evol. 2013;30(4):772-780. doi:10.1093/molbev/mst010

65. Bazinet AL, Zwickl DJ, Cummings MP. A gateway for phylogenetic analysis powered by grid computing featuring GARLI 2.0. Syst biol. 2014;63(5):812-818. doi:10.1093/sysbio/syu031

66. Posada D, Crandall KA. Modeltest: testing the model of DNA substitution. Bioinformatics. 1998;14:817-818.

67. Blomberg SP, Garland T, Ives AR. Testing for phylogenetic signal in comparative data: behavioral traits are more labile. Evolution. 2003;57(4):717-745. doi:10.1111/j.0014-3820.2003.tb00285.x

68. Revell LJ. Phytools: an R package for phylogenetic comparative biology (and other things). Methods Ecol Evol. 2012;3(2):217-223.

69. Maddison WP. Squared-change parsimony reconstructions of ancestral states for continuous-valued characters on a phylogenetic tree. Syst Biol. 1991;40(3):304-314.

# Supplementary

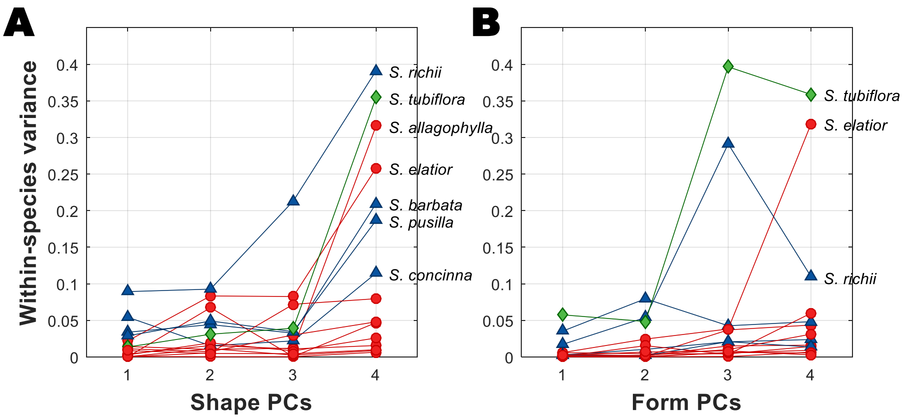


Figure S1. Within-species variance of the extant species in (A) sPCs and (B) fPCs. Red circle, blue triangle, and green diamond represent hummingbird-pollinated, bee-pollinated, and moth-pollinated species, respectively.

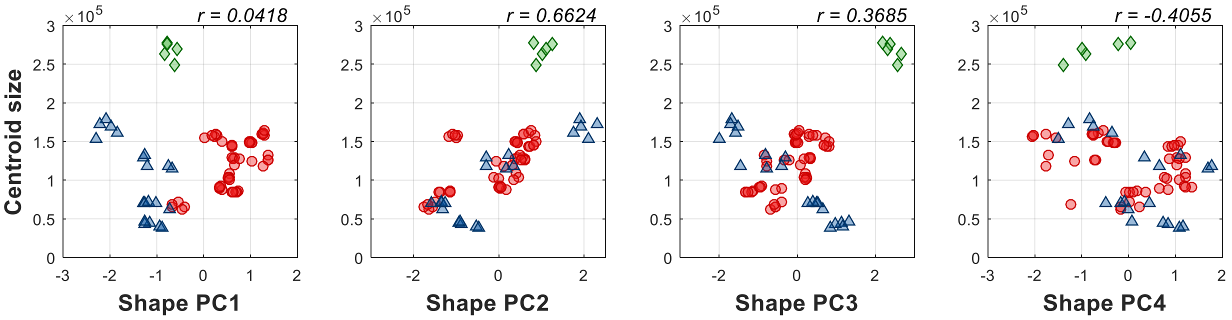


Figure S2. Scatter plot of centroid size versus each sPC. Red circle, blue triangle, and green diamond represent hummingbird-pollinated, bee-pollinated, and moth-pollinated species, respectively.

Table S1. Species list and GenBank numbers.

| Species | *trnS-trnG* | *ncpGS* | *rpl16* | *atpB-rbcL* | *trnL-trnF* | *trnT-trnL* |
|---|---|---|---|---|---|---|
| *S. aggregata* | AJ438364 | AJ459619 | AJ487715 | AJ439913 | AJ439757 | AJ439262 |
| *S. allagophylla* | AJ438407 | AJ459663 | AJ487758 | AJ439956 | AJ439801 | AJ439306 |
| *S. barbata* | AJ438386 | AJ459642 | AJ487738 | AJ439936 | AJ439780 | AJ439285 |
| *S. carangolensis* | AJ438391 | AJ459647 | AJ487743 | AJ439940 | AJ439785 | AJ439290 |
| *S. concinna* | AJ438393 | AJ459649 | AJ487745 | AJ439942 | AJ439787 | AJ439292 |
| *S. elatior* | AJ438398 | AJ459654 | AJ487749 | AJ439947 | AJ439792 | AJ439297 |
| *S. harleyi* | AJ438392 | AJ459648 | AJ487744 | AJ439941 | AJ439786 | AJ439291 |
| *S. nordestina* | AJ438387 | AJ459643 | AJ487739 | AJ439937 | AJ439781 | AJ439286 |
| *S. pusilla* | AJ438410 | AJ459666 | AJ487761 | AJ439959 | AJ439804 | AJ439309 |
| *S. richii* | AJ438403 | AJ459659 | AJ487754 | AJ439952 | AJ439797 | AJ439302 |
| *S. sceptrum* | AJ438399 | AJ459655 | AJ487750 | AJ439948 | AJ439793 | AJ439298 |
| *S. sellovii* | AJ438383 | AJ459639 | AJ487735 | AJ439933 | AJ439777 | AJ439282 |
| *S. tubiflora* | AJ438380 | AJ459636 | AJ487732 | AJ439930 | AJ439774 | AJ439279 |
| *S. valsuganensis* | AJ438401 | AJ459657 | AJ487752 | AJ439950 | AJ439795 | AJ439300 |
| *S. warmingii* | AJ438372 | AJ459627 | AJ487723 | AJ439921 | AJ439765 | AJ439270 |

Table S2. Centroid size and four morphological traits of extant species and ancestral states.

| | Centroid size ($10^4$) | Traits (mean ± standard deviation) | | | |
|---|---|---|---|---|---|
| | | Tube curvature ($10^{-4}$) | Lobe area ratio ($10^{-2}$) | Tube dilation ($10^{-1}$) | Lobe recurvation ($10^2$) |
| **Extant species** | | | | | |
| *S. aggregata* | 10.34 ± 0.29 | −1.77 ± 0.99 | 7.59 ± 0.56 | 8.99 ± 0.32 | 1.70 ± 0.29 |
| *S. allagophylla* | 6.71 ± 0.35 | −0.42 ± 0.82 | 14.93 ± 2.56 | 17.12 ± 0.73 | 2.93 ± 0.41 |
| *S. barbata* | 16.73 ± 1.01 | 14.26 ± 1.73 | 3.97 ± 0.24 | 16.01 ± 0.31 | 5.80 ± 0.25 |
| *S. carangolensis* | 12.71 ± 0.39 | −1.48 ± 0.21 | 7.59 ± 0.32 | 8.87 ± 0.40 | 1.61 ± 0.32 |
| *S. concinna* | 6.93 ± 0.35 | 8.51 ± 2.60 | 14.90 ± 0.86 | 9.79 ± 0.21 | 3.06 ± 0.22 |
| *S. elatior* | 12.54 ± 0.50 | −3.88 ± 1.51 | 6.33 ± 0.92 | 10.66 ± 1.20 | 4.26 ± 0.54 |
| *S. harleyi* | 15.75 ± 0.20 | −1.99 ± 0.50 | 8.87 ± 0.20 | 11.39 ± 0.28 | 6.11 ± 0.45 |
| *S. nordestina* | 8.52 ± 0.10 | −9.57 ± 0.28 | 12.89 ± 0.42 | 12.76 ± 0.53 | 1.90 ± 0.08 |
| *S. pusilla* | 4.26 ± 0.30 | 7.10 ± 2.29 | 13.56 ± 1.30 | 10.08 ± 0.67 | 3.41 ± 0.23 |
| *S. richii* | 12.25 ± 0.75 | 6.30 ± 0.84 | 10.61 ± 0.81 | 18.61 ± 1.05 | 3.37 ± 0.77 |
| *S. sceptrum* | 14.91 ± 0.13 | −1.37 ± 0.22 | 6.87 ± 0.05 | 8.76 ± 0.06 | 3.26 ± 0.21 |
| *S. sellovii* | 9.04 ± 0.14 | 0.70 ± 0.96 | 8.10 ± 0.76 | 16.71 ± 1.55 | 1.80 ± 0.29 |
| *S. tubiflora* | 26.70 ± 1.19 | 2.43 ± 0.27 | 9.42 ± 0.66 | 6.06 ± 0.14 | 5.00 ± 1.01 |
| *S. valsuganensis* | 16.03 ± 0.25 | −2.20 ± 0.25 | 6.14 ± 0.42 | 7.40 ± 0.20 | 4.20 ± 0.10 |
| *S. warmingii* | 14.53 ± 0.24 | 0.14 ± 0.45 | 5.17 ± 0.27 | 5.88 ± 0.07 | 1.80 ± 0.19 |
| **Ancestral states** | | | | | |
| Node 1 | 11.94 ± 0.11 | 6.18 ± 0.24 | 7.86 ± 0.18 | 15.45 ± 0.15 | 3.17 ± 0.21 |
| Node 2 | 11.91 ± 0.10 | 6.15 ± 0.26 | 7.60 ± 0.15 | 15.08 ± 0.12 | 3.16 ± 0.17 |
| Node 3 | 11.93 ± 0.10 | 5.64 ± 0.29 | 7.36 ± 0.13 | 15.05 ± 0.11 | 3.10 ± 0.15 |
| Node 4 | 11.47 ± 0.08 | 5.00 ± 0.23 | 7.51 ± 0.10 | 14.25 ± 0.10 | 2.72 ± 0.15 |
| Node 5 | 11.36 ± 0.06 | 2.97 ± 0.18 | 7.71 ± 0.08 | 13.32 ± 0.09 | 2.33 ± 0.12 |
| Node 6 | 11.80 ± 0.05 | 1.22 ± 0.13 | 7.28 ± 0.07 | 11.97 ± 0.08 | 2.15 ± 0.09 |
| Node 7 | 12.11 ± 0.05 | 1.15 ± 0.13 | 7.09 ± 0.08 | 11.75 ± 0.08 | 2.02 ± 0.05 |
| Node 8 | 10.80 ± 0.03 | 1.06 ± 0.11 | 7.21 ± 0.13 | 11.52 ± 0.22 | 1.78 ± 0.12 |
| Node 9 | 11.38 ± 0.05 | 1.39 ± 0.11 | 6.91 ± 0.12 | 9.88 ± 0.11 | 2.10 ± 0.11 |
| Node 10 | 12.15 ± 0.06 | 1.46 ± 0.12 | 6.38 ± .013 | 9.03 ± 0.08 | 2.12 ± 0.13 |
| Node 11 | 15.34 ± 0.11 | 1.96 ± 0.11 | 4.96 ± 0.14 | 7.16 ± 0.04 | 2.59 ± 0.23 |
| Node 12 | 11.69 ± 0.06 | 2.49 ± 0.19 | 8.10 ± 0.16 | 13.16 ± 0.08 | 3.90 ± 0.10 |
| Node 13 | 13.07 ± 0.07 | −1.71 ± 0.26 | 6.50 ± 0.14 | 10.18 ± 0.19 | 3.21 ± 0.10 |
| Node 14 | 13.23 ± 0.08 | −1.29 ± 0.06 | 6.80 ± 0.12 | 8.98 ± 0.09 | 2.39 ± 0.07 |

Table 1

Click here to access/download;Table;Table.1_5.3.docx

Table 1. List of studies using landmark-based GM.

| Approach | Imaging object | Keywords (selected) | Family | Landmark* | | GM | | References |
|---|---|---|---|---|---|---|---|---|
| | | | | Primary | Secondary | GPA | PCA/LDA | |
| 2D | Side-view | Petal shape; Floral morphology | Gesneriaceae | 5 | 10 | Full | PCA | [12] |
| | | Pollination syndrome; Phylogenetic comparative methods | Gesneriaceae | 6 | 26 | Full | PCA | [13, 14] |
| | | Pollinator selection; Flower shape | Loasaceae | 5 | 0 | Full | PCA, LDA | [15, 16] |
| | | Flower shape; Pollination niches | Plantaginaceae | 4 | 12 | Full | PCA | [17] |
| | | Trait-dependent diversification; Pollination; Flower tube | Plantaginaceae | 9 | 1 | Full | LDA | [18] |
| | | Ancillary traits | Rubiaceae | 9 | 8 | Full | PCA, LDA | [19] |
| | Face-view | Selfing syndrome | Brassicaceae | 30 | 0 | Full | PCA | [20] |
| | | Floral shape evolution; pollination; corolla shape; plant-pollinator interactions; floral morphospace; allometry | Brassicaceae | 32 | 0 | Full | PCA | [21–26] |
| | | Petal shape; Floral morphology | Gesneriaceae | 5 | 25 | Full | PCA | [12] |
| | | Floral shape** | Goodeniaceae | 5 | 0 | Full | PCA | [27] |
| | | Fluctuating asymmetry | Orchidaceae | 4 | 5 | Full | PCA | [28] |
| | | Flower shape | Plantaginaceae | 28 | 0 | Full | PCA | [29] |
| | | Corolla shape; pollination | Solanaceae | 5 | 35 | Full | PCA | [30] |
| | | Floral symmetry | Valerianaceae | 10 | 0 | Full | LDA | [31] |
| | Dissected-view | Actinomorphy; Zygomorphy | Leguminosae | 2 | 18 | Full | PCA | [32] |
| | | Floral shape** | Leguminosae | 37 | 0 | Full | PCA | [33] |
| | | Directional asymmetry; floral organ shape; fluctuating asymmetry | Iridaceae | 39 | 16 | Full | PCA | [34, 35] |
| | | Floral shape** | Orchidaceae | 15 | 0 | Full | PCA | [36] |
| | | Floral shape, allometry** | Plantaginaceae | 4 | 16 | Partial | PCA | [37] |
| | | Floral shape** | Plantaginaceae | 8 | 47 | Partial | PCA | [38] |
| 3D | Whole corolla | Petal shape; dorsoventral asymmetry | Gesneriaceae | 10 | 65 | Full | PCA | [8] |
| | | Dorsoventral asymmetry; petal form variation | Gesneriaceae | 25 | 100 | Full | PCA | [11] |
| | | Floral shape, pollination** | Orchidaceae | 26 | 14 | Full | PCA | [10] |
| | | Corolla shape variations | Gesneriaceae | 25 | 390 | Full/ Partial | PCA | Present study |

* The primary and secondary landmarks follow the definitions in the Methods section.

** Keywords were not provided in these studies. The keywords were assigned by authors of the present study.

Click here to access/download;Table;Table.2_5.3.docx

Table 2. Species list and dimension of the slice images.

| Species | Pollination Type* | Specimen type** | Dimension of slice image (pixels) | Number of slice image | KBCC accession |
|---|---|---|---|---|---|
| *S. aggregata* | H | F | 1000×1000 | 540-610 | K039092 |
| *S. allagophylla* | H | F | 1000×1000 | 540-610 | K039099 |
| *S. barbata* | B | E/F | 1968×1968*** | 1150-1390 | K039105 |
| *S. carangolensis* | H | F | 1000×1000 | 1120-1220 | K039112 |
| *S. concinna* | B | F | 1000×1000 | 580-680 | K039118 |
| *S. elatior* | H | E | 1000×1000 | 1045-1370 | K039127, K039129 |
| *S. harleyi* | H | F | 1000×1000 | 1280-1400 | K039135 |
| *S. nordestina* | H | F | 1000×1000 | 780-800 | K039168 |
| *S. pusilla* | B | F | 1000×1000 | 609-666 | K039169, K039170 |
| *S. richii* | B | E/F | 1000×1000 | 1060-1160 | K039174 |
| *S. sceptrum* | H | E | 1000×1000 | 1260-1298 | K039178 |
| *S. sellovii* | H | E | 1000×1000 | 930-1000 | K039184 |
| *S. tubiflora* | M | F | 1968×1968*** | 2230-2470 | K039197, K039200 |
| *S. valsuganensis* | H | F | 1000×1000 | 1540-1520 | K039203 |
| *S. warmingii* | H | E | 1000×1000 | 1160-1320 | K039205 |

* H: hummingbird pollination (ornithophily), B: bee pollination (melittophily), and M: moth pollination (phalaenophily).
** The letter E denotes the 70% ethanol-fixed specimen, and the letter F denotes the fresh specimen.
***The 3D images with a slice size of 1968 × 1968 were downsized to 984 × 984 before the reconstruction of volumetric and surface images. The identified landmarks were then magnified back to the original scale for the subsequent GM analysis.

Click here to access/download;Table;Table.3_5.3.docx

Table 3. Kruskal–Wallis test results, Scheffé's multiple comparison test results, and LOD scores of the morphological traits by pollination type.

| Morphological traits | Kruskal-Wallis test | | Scheffé's multiple comparison test | | | | | | LOD score | *p*-value |
|---|---|---|---|---|---|---|---|---|---|---|
| | | | Hummingbird vs Bee | | Hummingbird vs Moth | | Bee vs Moth | | | |
| | *H*-value | *p*-value | *T*-value | *p*-value | *T*-value | *p*-value | *T*-value | *p*-value | | |
| **Centroid size** | 16.16 | $3.10\times10^{-4}$ | 2.13 | $1.11\times10^{-1}$ | 9.09 | $1.14\times10^{-12}$ | 8.48 | $1.46\times10^{-11}$ | 12.65 | 0 |
| **Tube curvature** | 50.66 | $9.99\times10^{-12}$ | 13.82 | $1.11\times10^{-16}$ | 3.21 | $8.21\times10^{-3}$ | 4.31 | $2.60\times10^{-4}$ | 21.16 | 0 |
| **Lobe area ratio** | 9.81 | $7.40\times10^{-3}$ | 3.34 | $5.64\times10^{-3}$ | 0.65 | $8.08\times10^{-1}$ | 1.15 | $5.81\times10^{-1}$ | 2.35 | $6.25\times10^{-3}$ |
| **Tube dilation** | 17.65 | $1.47\times10^{-4}$ | 2.88 | $1.99\times10^{-2}$ | 2.81 | $2.36\times10^{-2}$ | 4.16 | $4.31\times10^{-4}$ | 3.86 | $1.90\times10^{-4}$ |
| **Lobe recurvation** | 12.89 | $1.60\times10^{-3}$ | 2.57 | $4.20\times10^{-2}$ | 3.12 | $1.05\times10^{-2}$ | 1.56 | $3.01\times10^{-1}$ | 2.92 | $1.69\times10^{-3}$ |

Click here to access/download;Figure;Fig.1_5.3.pdf

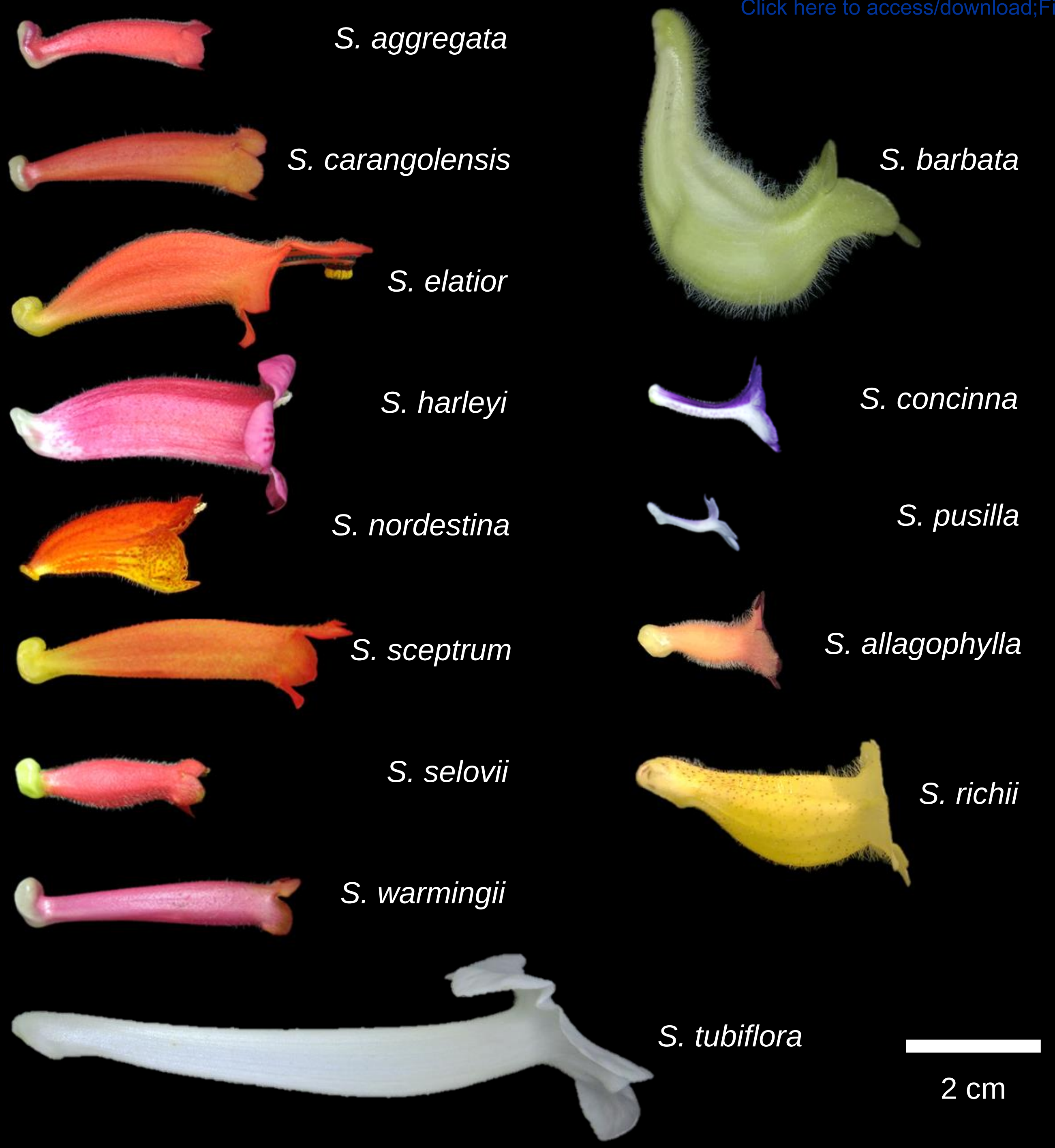

Figure 2

Click here to access/download;Figure;Fig.2_5.3.pdf

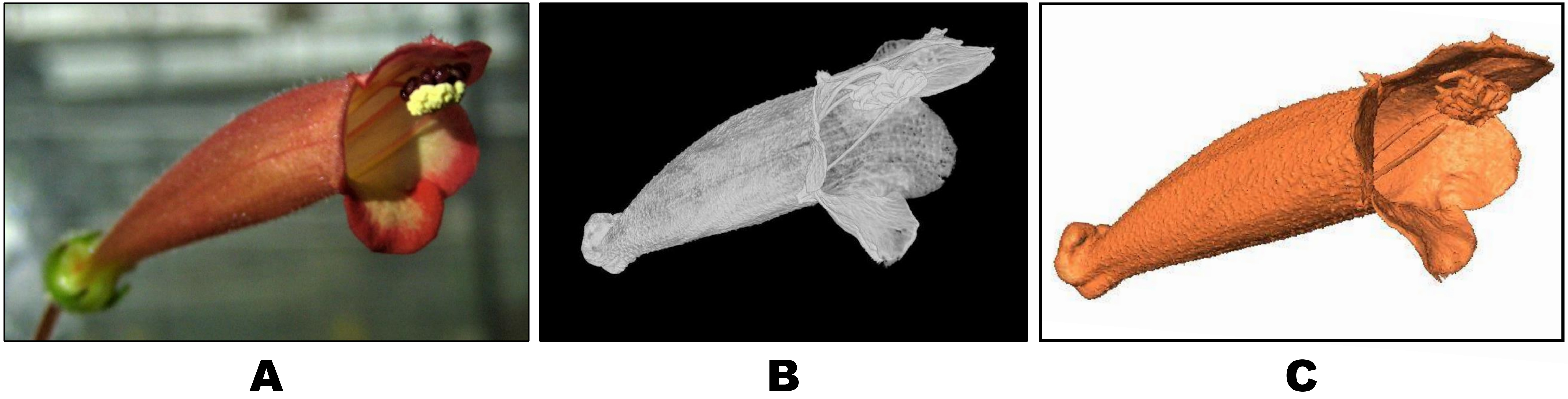

Figure 3

Click here to access/download;Figure;Fig.3_5.3.pdf

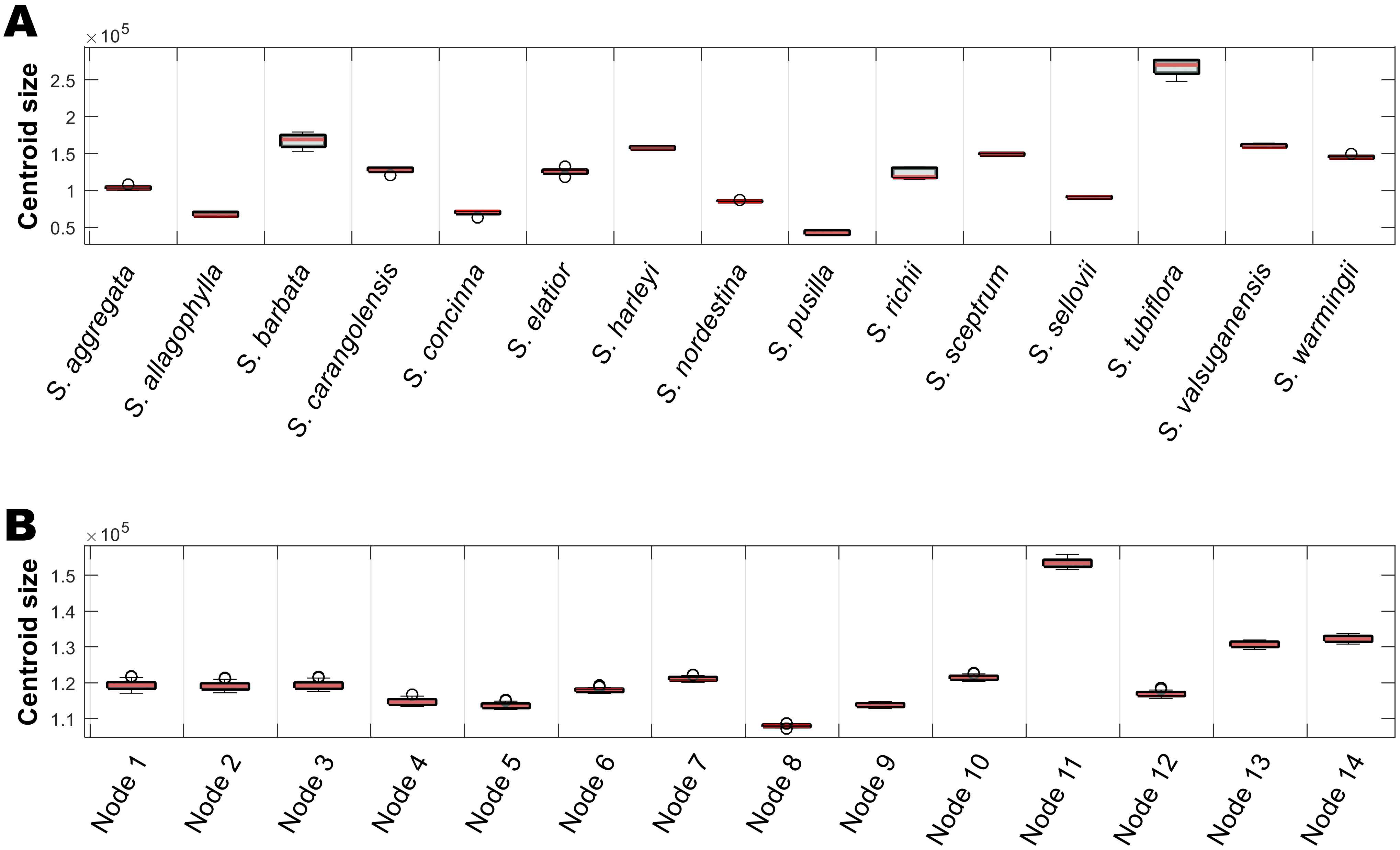

Click here to access/download;Figure;Fig.4_5.3.pdf

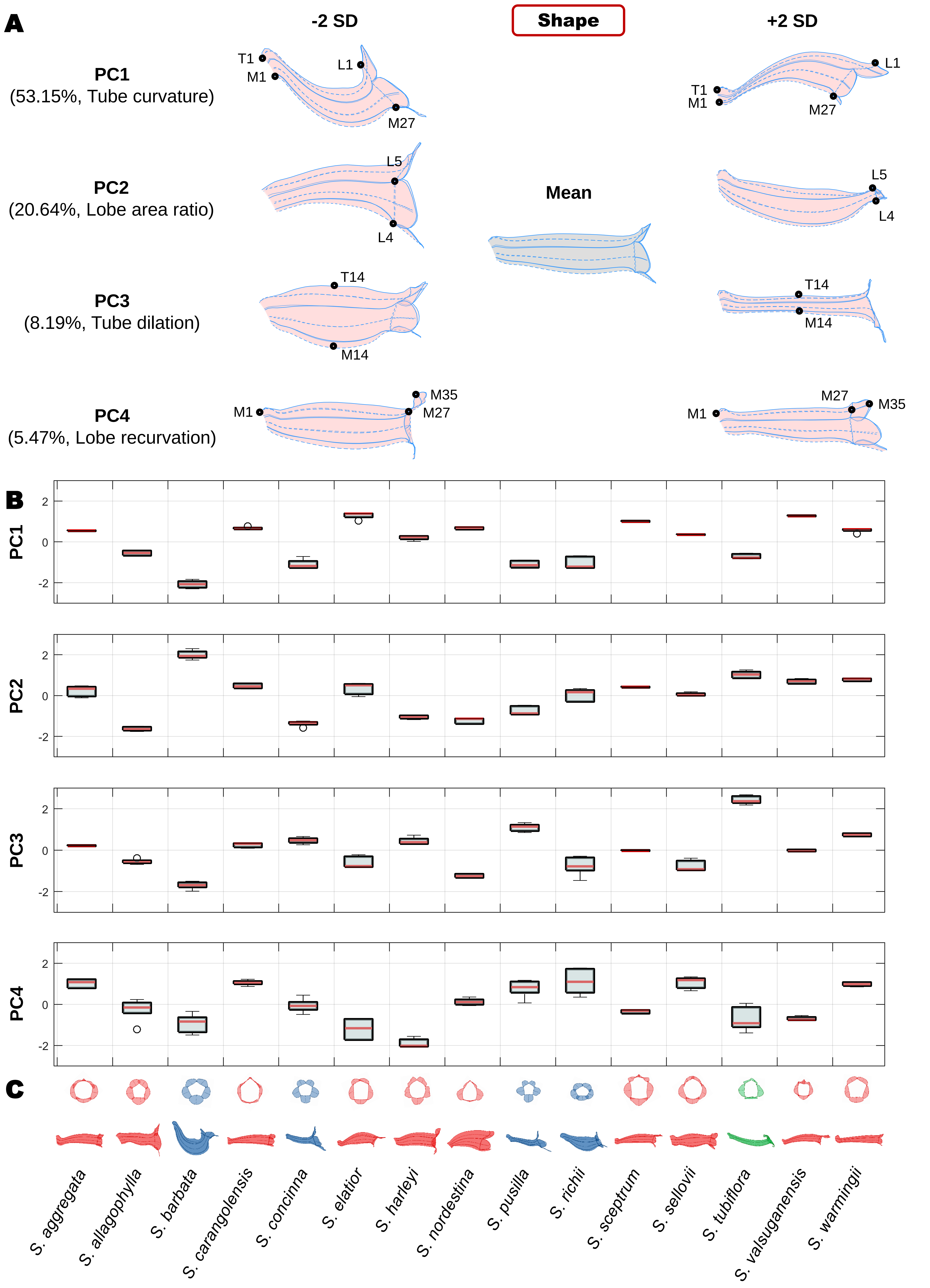

Click here to access/download;Figure;Fig.5_5.3.pdf

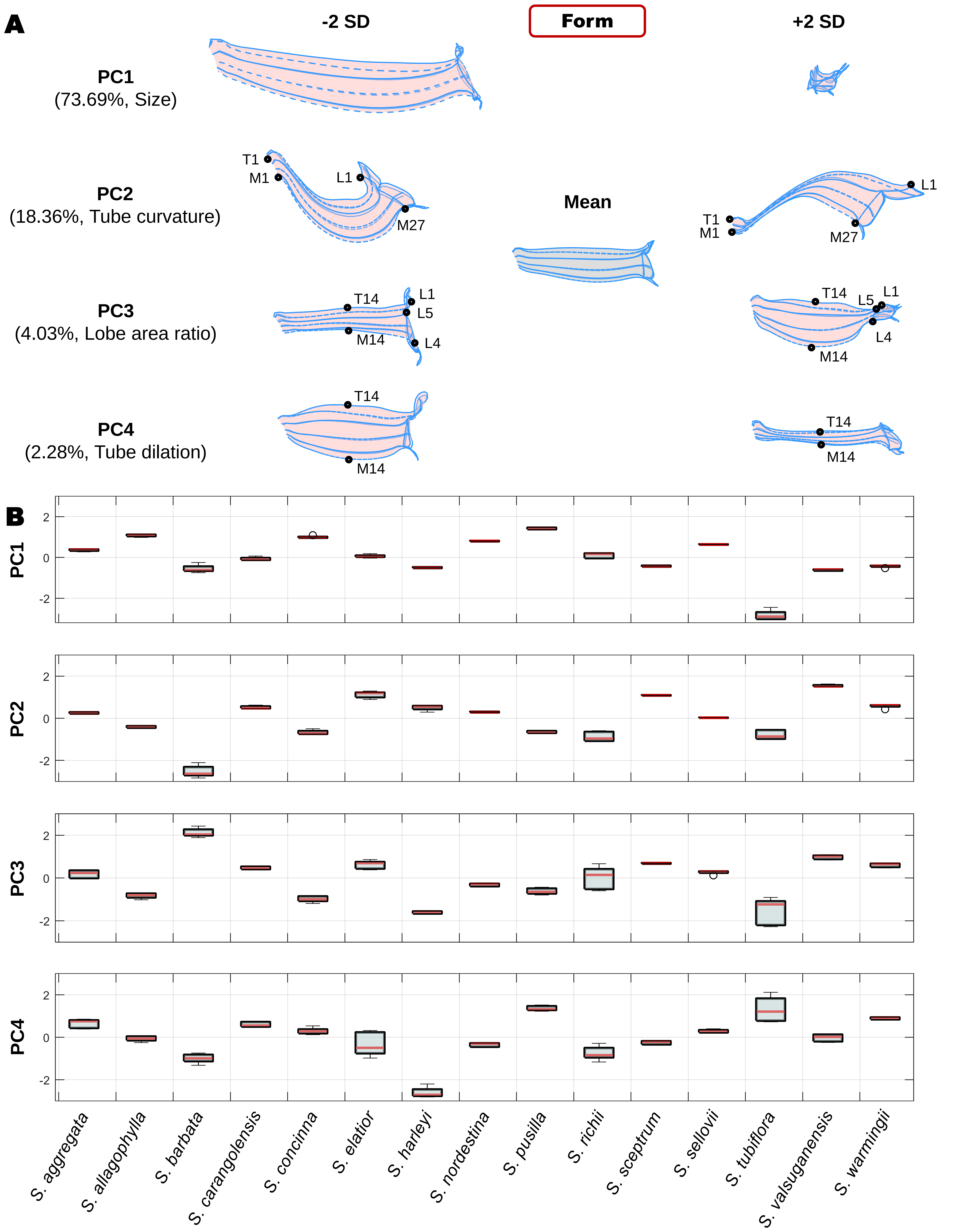

Figure 6

Click here to access/download;Figure;Fig.6_5.3.pdf

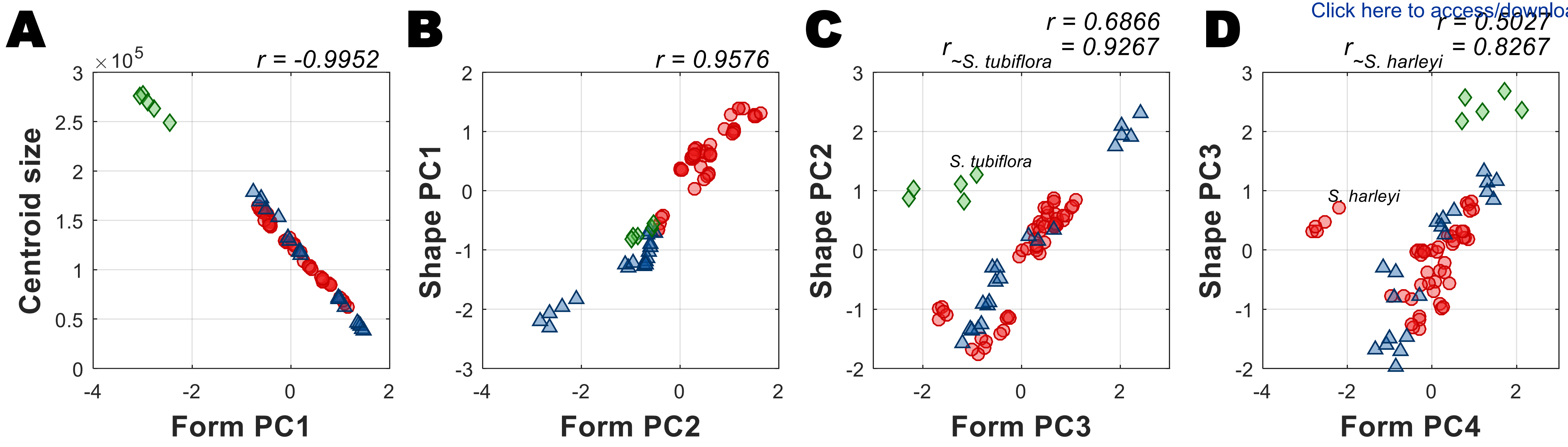

Figure 7

Click here to access/download;Figure;Fig.7_5.3.pdf

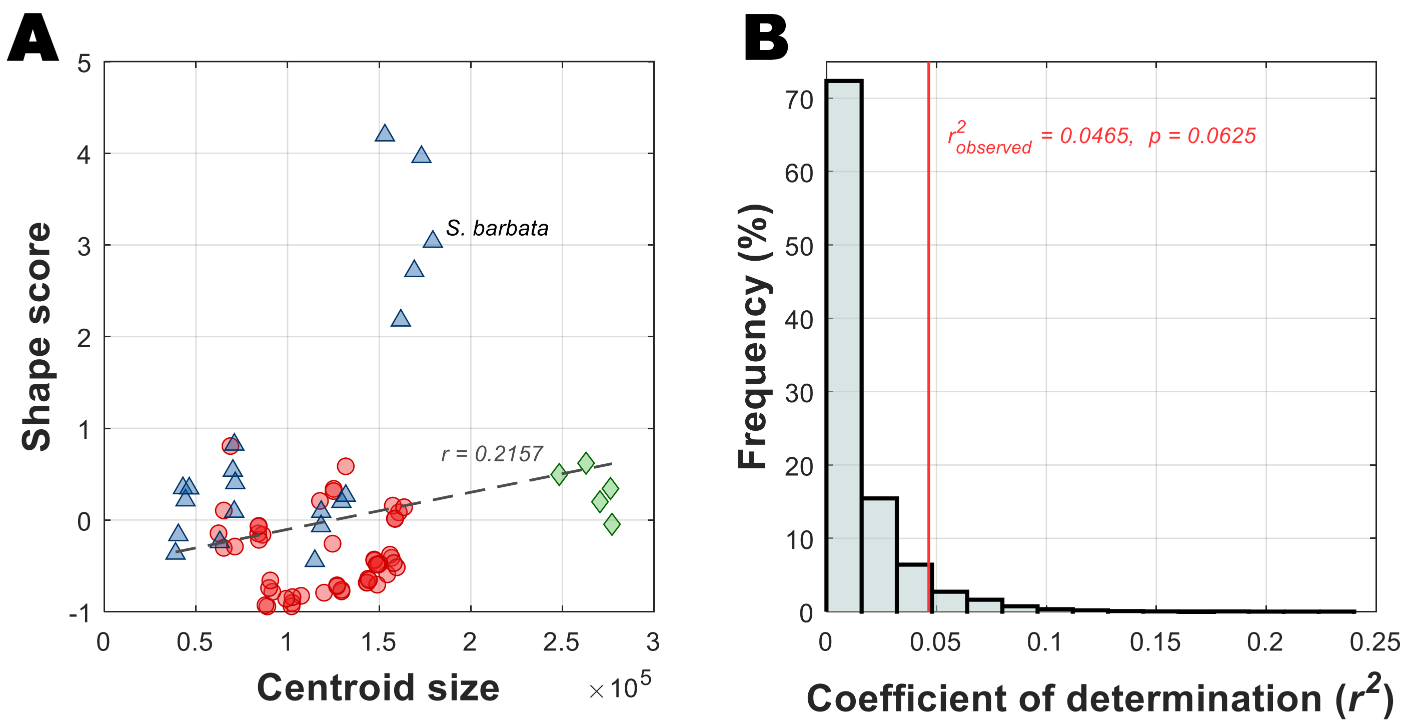

Figure 8

Click here to access/download;Figure;Fig.8_5.3.pdf

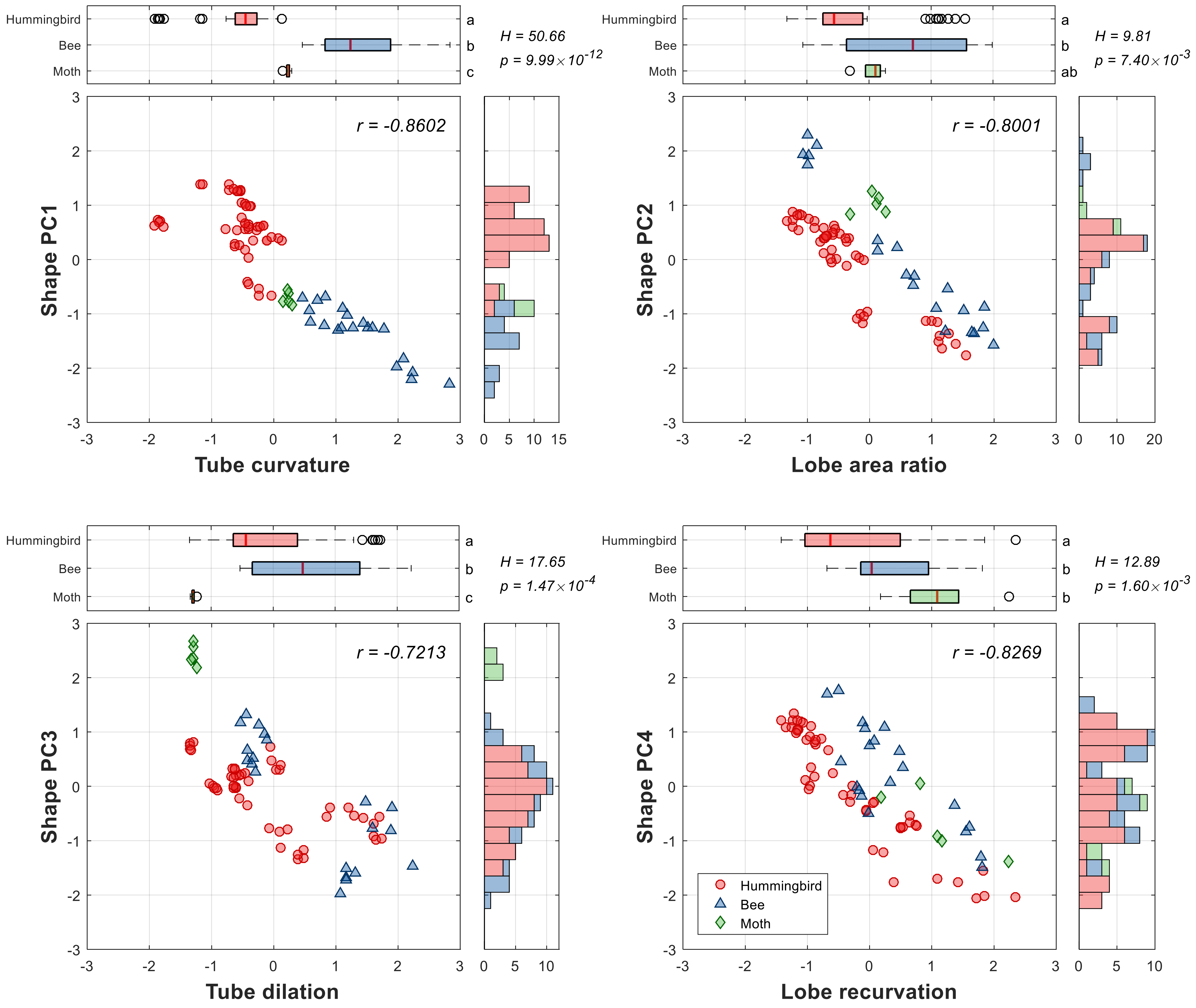

Figure 9

Click here to access/download;Figure;Fig.9_5.3.pdf

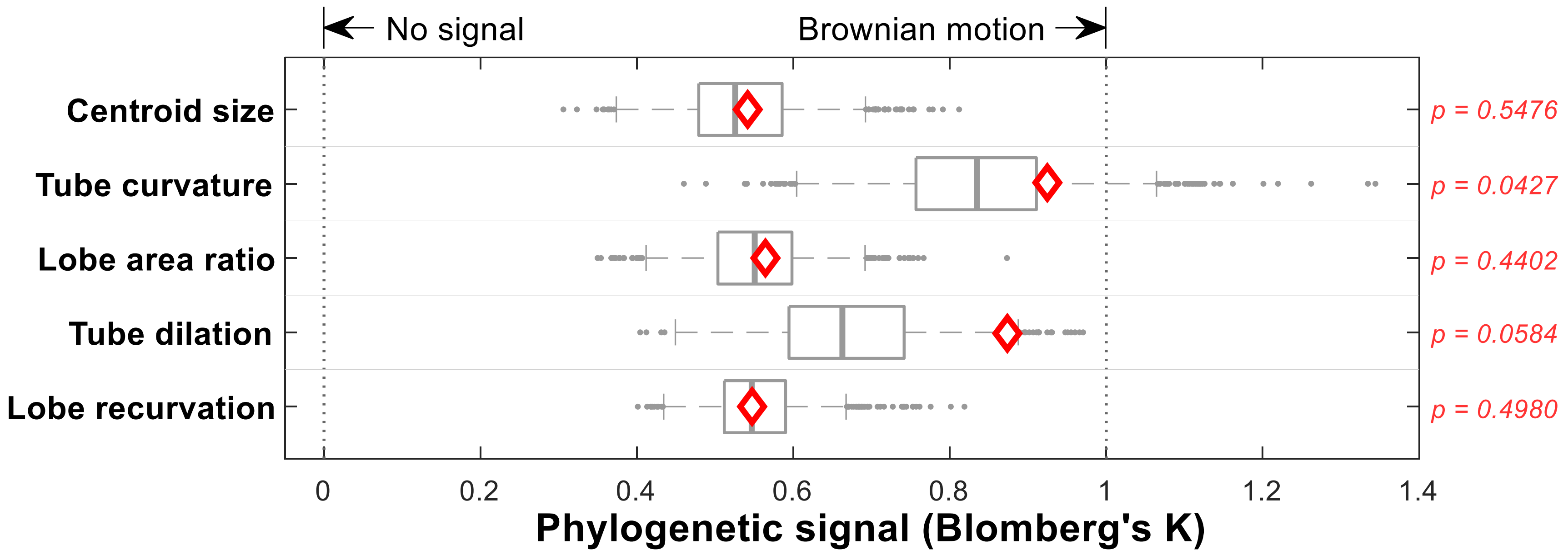

Click here to access/download;Figure;Fig.10_5.3.pdf

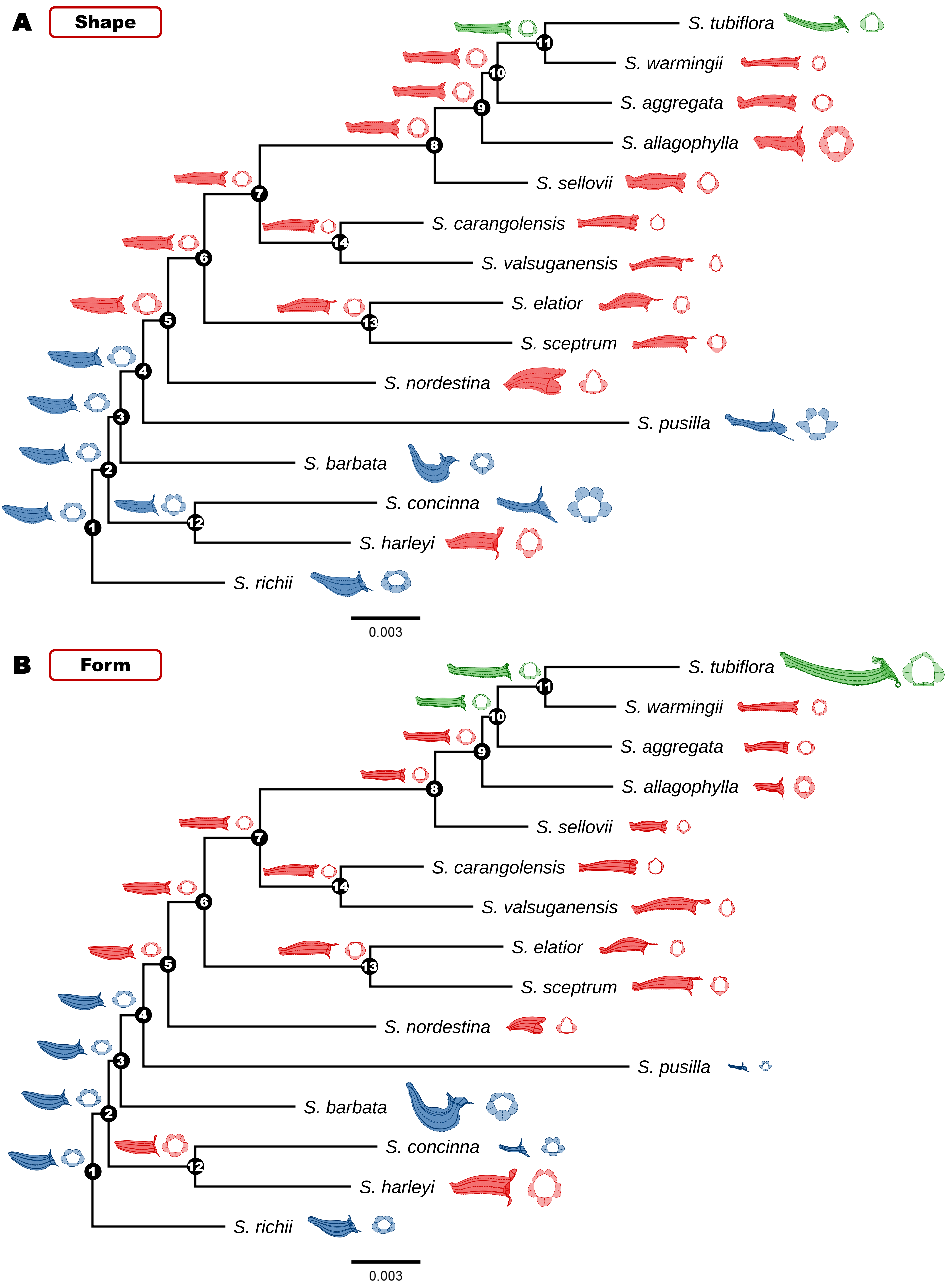

Figure 11

Click here to access/download;Figure;Fig.11_5.3.pdf

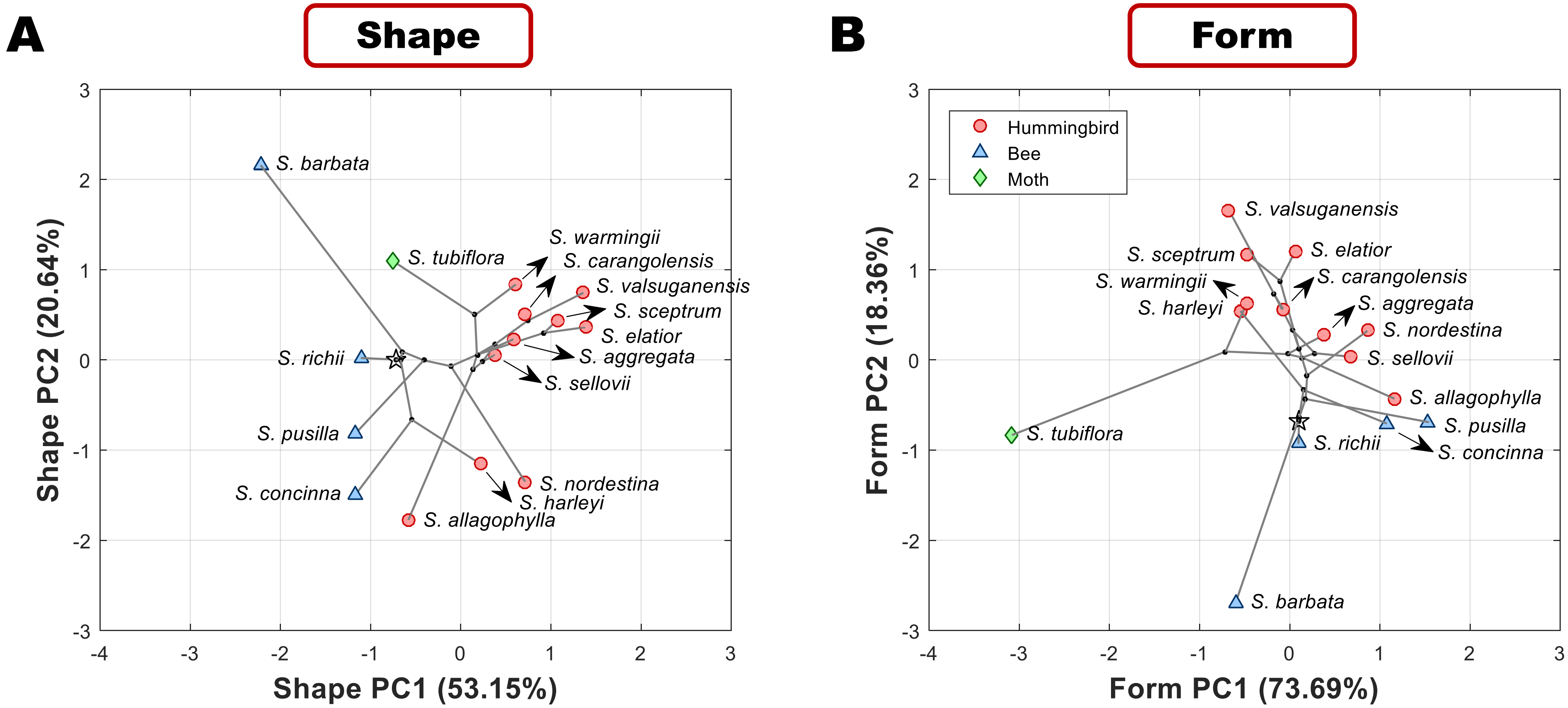

Figure 12

Click here to access/download;Figure;Fig.12_5.3.pdf

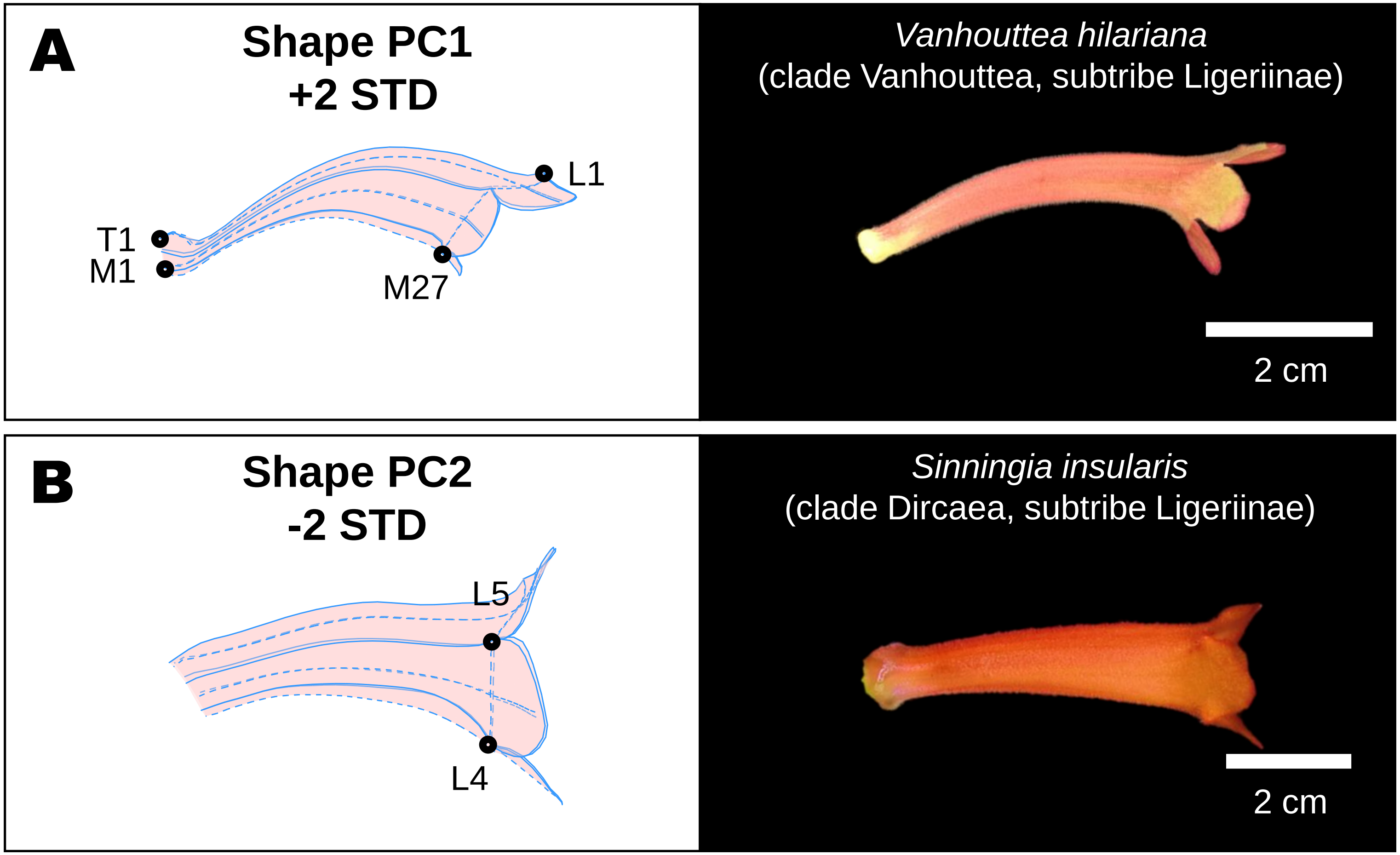

Figure 13

Click here to access/download;Figure;Fig.13_5.3.pdf

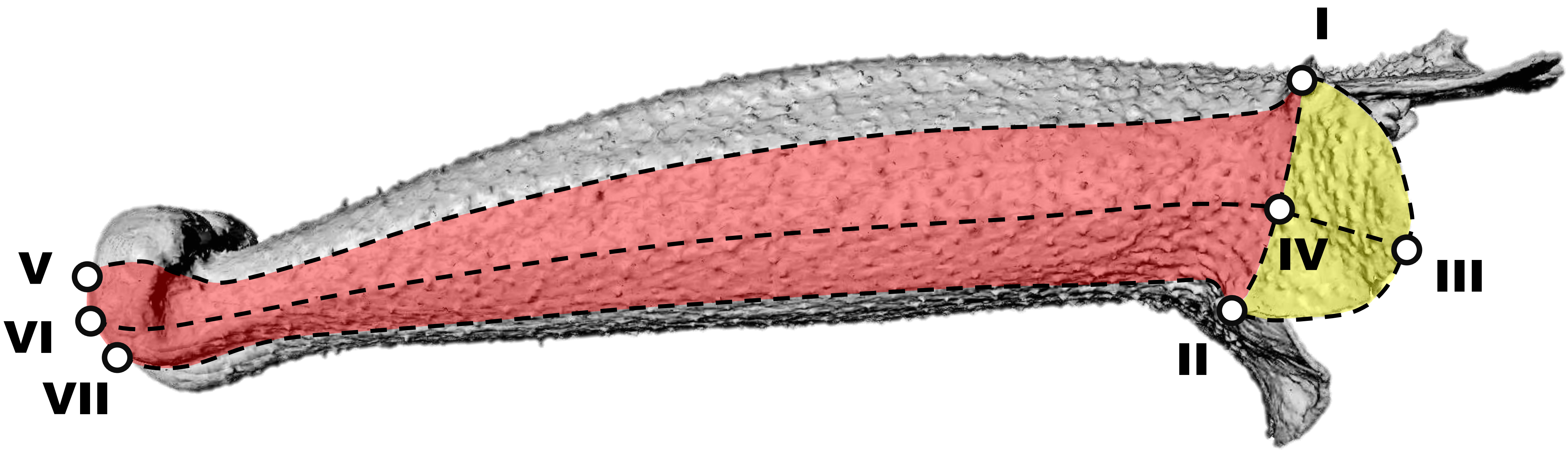

Figure 14

Click here to access/download;Figure;Fig.14_5.3.pdf

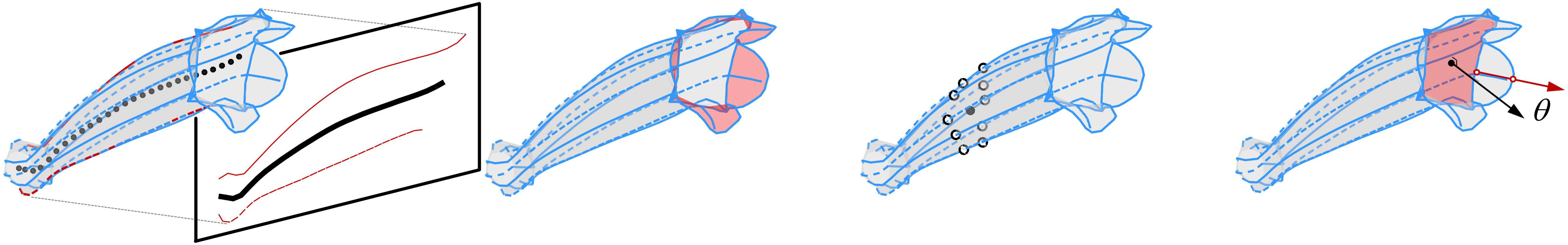


A

B

C

D

Supplementary Material, Table S1

Click here to access/download
**Supplementary Material**
Table.S1_5.3.docx

Supplementary Material, Table S2

Click here to access/download
**Supplementary Material**
Table.S2_5.3.docx

Supplementary Material, Figure S1

Click here to access/download
**Supplementary Material**
Fig.S1_5.3.pdf

Supplementary Material, Figure S2

Click here to access/download

**Supplementary Material**

Fig.S2_5.3.pdf

Personal Cover

Click here to access/download;Personal Cover;17Genus Sinningia 3D GM_Cover Letter.docx

Dr. Goodman
Editor-in-Chief
*GigaScience*

July 7, 2019

Dear Dr. Goodman,

Please find enclosed our manuscript entitled "3D revelation of phenotypic variation, evolutionary allometry, and ancestral states of corolla shape: a case study of clade Corytholoma (subtribe Ligeriinae, family Gesneriaceae)" by Hao-Chun Hsu, Wen-Chieh Chou, Yan-Fu Kuo.

In this manuscript, we applied X-ray microcomputed tomography (µCT) to acquire three-dimensional (3D) structures of the corollas of clade Corytholoma. We acquired volumetric images of the corollas and extracted a set of 415 3D landmarks from each volumetric image. We identified the major shape and form variations of the corollas from the landmarks by using geometric morphometrics (GM). We defined and quantified morphological traits corresponding to the major shape variations and used the trait values to examine their association with pollination type and to evaluate the phylogenetic signals. We also used landmarks to reconstruct corolla shapes at the ancestral states. The proposed approach revealed that the first four principal components (PCs) in the shape and form analyses, respectively, accounted for 87.45% and 98.36% of the total variance. The centroid sizes of the corollas only accounted for 4.65% of the corolla shape variation, suggesting that the evolutionary allometry was weak. The four morphological traits corresponding to the four shape PCs were defined as tube curvature, lobe area, tube dilation, and lobe recurvation. Tube curvature and tube dilation were strongly associated with the pollination type and contained phylogenetic signals in clade Corytholoma. We also plan to submit raw 2D slice images obtained from µCT to the *GigaDB* repository. We believe that our findings would appeal to the readership of *GigaScience* because revealing the evolution and diversity of corolla shape is a raising issue. We also believe that this study meets the goal of *GigaScience* because the data to be submitted to *GigaDB* can promoting the reproducibility of analyses and the findable and reusable of research data.

This manuscript has not been published and is not under consideration for publication elsewhere. We have no conflicts of interest to disclose. All authors have approved the manuscript and agree with its submission to *GigaScience*.

We look forward to hearing from you at your earliest convenience.

Sincerely yours,

Yan-Fu Kuo, PhD
Associate Professor
Department of Biomechatronics Engineering
National Taiwan University
