# Supplementary material for: 3D revelation of phenotypic variation, evolutionary allometry, and ancestral states of corolla shape: a case study of clade Corytholoma (subtribe Ligeriinae, family Gesneriaceae)
Source: Gigascience. 2020 Jan 22;9(1):giz155. doi: 10.1093/gigascience/giz155 (PMC6974915; doi:10.1093/gigascience/giz155)
Supplement: giz155_GIGA-D-19-00247_Revision_1 [file giz155_giga-d-19-00247_revision_1.pdf]

GigaScience

3D revelation of phenotypic variation, evolutionary allometry, and ancestral states of corolla shape: a case study of clade Corytholoma (subtribe Ligeriinae, family Gesneriaceae)

--Manuscript Draft--

**Manuscript Number:**	GIGA-D-19-00247R1
**Full Title:**	3D revelation of phenotypic variation, evolutionary allometry, and ancestral states of corolla shape: a case study of clade Corytholoma (subtribe Ligeriinae, family Gesneriaceae)
**Article Type:**	Research
**Funding Information:**	Ministry of Science and Technology, Taiwan (TW) (NSC-101-2313-B-002-050-MY3) — Dr. Yan-Fu Kuo
**Abstract:**	Background: Quantification of corolla shape variations helps biologists to investigate the diversity and evolution in plants. This study applied X-ray microcomputed tomography (μCT) to acquire three-dimensional (3D) structures of the corollas of clade Corytholoma. After acquiring volumetric images of the corollas and extracting a set of 415 3D landmarks from each volumetric image, the major 3D shape and 3D form variations of the corollas were identified from the landmarks by using geometric morphometrics (GM). Evolutionary allometry of the corolla shape was assessed. Morphological traits corresponding to the major shape variations were also defined and quantified and were subsequently used to examine their association with pollination type and to evaluate the phylogenetic signals. The landmarks were further used to reconstruct corolla shapes at the ancestral states. Results: GM results revealed that the first four principal components (PCs) in the 3D shape and 3D form analyses, respectively, accounted for 87.86% and 96.34% of the total variance. The centroid sizes of the corollas only accounted for 5.46% of the corolla shape variation, suggesting that the evolutionary allometry was weak. The four morphological traits corresponding to the four shape PCs were defined as tube curvature, lobe area, tube dilation, and lobe recurvation. Tube curvature and tube dilation were strongly associated with the pollination type and contained phylogenetic signals in clade Corytholoma. Conclusions: With the integration of μCT imaging into GM, the proposed approach boosted the precision in quantifying corolla traits and improved the understanding of the morphological traits corresponding to the pollination type, impact of size on shape variation, and evolution of corolla shape in clade Corytholoma.
**Corresponding Author:**	Hao-Chun Hsu<br><br>TAIWAN
**Corresponding Author Secondary Information:**	
**Corresponding Author's Institution:**	
**Corresponding Author's Secondary Institution:**	
**First Author:**	Hao-Chun Hsu
**First Author Secondary Information:**	
**Order of Authors:**	Hao-Chun Hsu
	Wen-Chieh Chou
	Yan-Fu Kuo
**Order of Authors Secondary Information:**	
**Response to Reviewers:**	###Please find the "Person cover (response to reviewer)" for the complete response (with figures)###

Powered by Editorial Manager® and ProduXion Manager® from Aries Systems Corporation

Reviewer #1
In their manuscript "3D revelation of phenotypic variation, evolutionary allometry, and ancestral states of corolla shape: a case study of clade Corytholoma (subtribe Ligeriinae, family Gesneriaceae)", Hsu et al. present a thorough morphometric analysis of corolla shape. I very much enjoyed reading this manuscript. It integrates 2D and 3D geometric morphometrics with evolutionary theory in an unprecedented way using plant organs as an example. The morphometric analyses and the specimens are beautiful. The availability of 2D slice images, volumetric images, and surface images in the GigaScience Database repository is extremely valuable for the reproducibility of morphometric studies.

I only have minor suggestions to improve readability.
... Author: Many thanks for this very positive review.

The reader is often referred to references [8] and [11] for critical methods information. This includes: image processing information (page 10, lines 181-185), landmark selection procedure (page 28, lines 469-470), and visualization (page 29, line 505). While of course all the information doesn't need to be repeated here, it should be summarized, so that the reader can understand the gist of what is being done without having to refer back to these publications.

... Author: Thanks for the comment. The manuscript was revised accordingly. The image processing information was updated in Lines 166-179 of the revised manuscript. The landmark selection procedure was updated in Lines 188-211 of the revised manuscript. The visualization was updated in Lines 508-509 of the revised manuscript.

Can a full explanation of the primary landmarks be provided? Figure 13 helps, but it would be good to see the full 25 primary landmarks. It would also help to see a visualization of all secondary landmarks too, just so the reader has a visual understanding of what the data is based upon. It might help to do this for two morphologically distinct species. For those not familiar with corolla morphology in these species, a botanical/morphological/anatomical description of what the primary landmarks correspond to would help understanding.

... Author: Thanks for the insightful comment. The Fig. 3 of the revised manuscript was updated accordingly (Line 212). In the Fig. 3, two species with distinct corolla morphology were provided. The details corresponding to the corolla morphology, petal position, homologous features were provided in the revised manuscript (Lines 188-203).

Scaling is used for shape but not for form. Why?

... Author: Thanks for the comment. Please let us clarify further. Form variation is defined as the variation of shape and size together, and the scale (i.e., distances) between landmarks present the size. To retain form information in the landmarks, scaling cannot apply to the landmarks. Thus, when size is considered as a critical factor (e.g., [1, 2]), partial-GPA is used to investigate the form variation.

... [1] Klingenberg CP. Evolution and development of shape: integrating quantitative approaches. Nat Rev Genet. 2010;11(9):623. doi:10.1038/nrg2829
... [2] Klingenberg CP. Size, shape, and form: concepts of allometry in geometric morphometrics. Dev Genes Evol. 2016;226(3):113-137. doi:10.1007/s00427-016-0539-2

Also, I know "shape" and "form" have strict meanings in the morphometric community. But upon first usage, it might be good to just add "2D" and "3D" for clarification, not only for those not familiar with morphometrics, but also in some non-English languages the use of "shape" and "form" is confusing.

... Author: Thanks for the comment. Please let us clarify further. In this study, the shape and form analyses were both carried out in 3D using 3D landmarks. We also added “3D” before shape and form throughout the revised manuscript (highlighted in green) and hoping that this will help to clarify the use of shape and form.

The methods come after the results section. But at least for the landmarks, an explanation up front might be helpful for readers to understand the analysis and interpret it.

... Author: Thanks for the insightful comment. The manuscript was updated accordingly (Lines 187-220 of the revised manuscript).

Reviewer #2
In the manuscript "3D revelation of phenotypic variation, evolutionary allometry, and ancestral states of corolla shape: a case study of clade Corytholoma (subtribe Ligeriinae, family Gesneriaceae)", Hsu et al. applied 3D X-ray microcomputed tomography and landmark-based geometric morphometrics to study the corolla shape variation, evolutionary allometry, and some phylogenetic analysis. The manuscript is well written. The details are explained very clear. For their data, the analysis has been performed thoroughly.

... Author: Many thanks for this very positive review.

The main concern I have is the sample size. In this work, the authors studied 15 species. However, each species only has one or two plant individuals and also were collected from different years. Although five samples from this single or two plants were scanned, some of the analysis and statements cannot convince me. In my opinion, if this is a method or breakthrough technology paper, using small sample size to test and valid the method might be fine. But this manuscript is not a method paper because all the methods are existing and commonly used. For the work focusing on the biology, I would evaluate the result which should be supported by solid analysis with enough sample size. In this work, I was wondering whether this single (or two) individual plant are representative. Assume so, then I think the analysis for the major variation using PCA and the phylogenetic analysis based on the mean shape might be fine. But the result related the within-species variation cannot convince me. Can the variance in this particular plant represent the variance for the entire species? I don't think so. The variation basically is the within-plant variation. Thus the analysis and result such as Fig. 3A, Fig. 4B, 5B are supported well. In addition, Fig 3 and Fig 4B don't have the statistic.

... Author: Thanks for the insightful comment.

... In short, the specimen number increases from 75 to 153 in the revised manuscript. Please allow us to explain the reason of including only 75 specimens in the original manuscript below.

... We agree that a large sample size is always a good practice for analyzing the shape variations. However, 3D CT scanning is costly and time-consuming. The specimen used for 3D scanning was preselected according to corolla shape variation analysis using 2D side views (Fig. R1 below). In the past years, we only have collected a total of 153 corolla specimens with uneven specimen numbers for each species. To assess the within-species variation, all the 153 specimens were included in the analyses of 3D shape and form variation and of evolutionary allometry. To avoid the results may be dominated by the species with larger sample size or the species with larger morphological variation, five specimens of each species selected based on shape score were used in the analyses of phylogenetic signal and of ancestral state reconstruction (Lines 147-152 of the revised manuscript). The Table 1, 2, Fig. 4, 5B, 6B, 7, 8, 9, S1, and S2 of the revised manuscript were updated.

... (Please find the "Person cover [response to reviewer]" for the figure)
... Figure R1. Illustration of 2D side-view corolla shape variation analysis. The left top was the 2D side-view image, the left bottom was the selected landmark in the image, and the right was the scatter plot of PC scores obtained from GM analysis using 2D side-view landmarks.

... Per the reviewer’s suggestion, we include all the 153 specimens in the revised manuscript. Each species has at least two plant individuals. The updates are reflected in Lines 135-137 and 139-141 of the revised manuscript.

Powered by Editorial Manager® and ProduXion Manager® from Aries Systems Corporation

... The specimen for the same species was collected in the same flowering season (Lines 141-142 of the revised manuscript). Each species has its own habits; the flowering time of most species was not synchronized.

... In Fig. 4 (Line 229), 5B (Line 256), and 6B (Line 285) of the revised manuscript, the multiple comparison tests for centroid size and PCs were provided. We hope that this will help to present the within-species variation.

Another point is about the landmark based method. First, in this work, 25 primary landmarks are selected manually first. Then the rest 390 secondary landmarks are derived computationally, but still highly rely on the manual landmarks. Thus this landmark dataset may also introduce error, and can be subjective and labor intensive. 3D X-ray images also has variance for each scan. This has the similar problem with other method such as the author mentioned for other work: (line 89) "2D images are usually acquired manually, which may introduce error or artifacts…"; (line 97) " distance-based traits are typically proposed based on manual observation and can be subjective"; (line 126) " the traits were proposed statistically rather than manually". Second, the landmark based method has been widely applied. However, it has a main limitation that you have to make sure the landmarks are homologous. I was wonder whether all the corollas have five petals. If not, how the authors made sure the landmark were homologous? Because of this limitation, similar work cannot be easily expanded to a wider range of species. It would be better to discuss a bit in the discussion.

... Author: Thanks for the insightful comment. We revised the paragraphs of 3D flower image data (Lines 166-179), landmark identification (Line 188-211) and updated the Fig. 2 (Line 181) and 3 (Fig. 212) of the revised manuscript.

... We agree that manual landmark selection may introduce error, is subjective, and is labor-intensive. CT images are at a very high resolution (36.547 micron). The details of the corollas are clearly visible. Although all the primary landmarks and the tube-tube rims were selected manually using a software Landmark, these anatomical features are obvious and can easily be identified (Fig. 3 of the revised manuscript). Thus, the error introduced by manual selection can be minimized. The secondary landmarks were automatically selected using a software developed by our team [1]. Thus, the selection of the secondary landmarks can be objective.

... The 3D imaging of each specimen was calibrated using a bar phantom (for calibrating the spatial resolution and the X-ray quality, Lines 167-168 of the revised manuscript). The scan parameters like source voltage, source current, and scan resolution were than determined (Table S2 of the revised manuscript).

... In our humble opinion, the 3D approach can overcome the "subjective" issues using 2D imaging. For example, the angle of capturing the image of the object is largely controlled by the operator. This is no doubt that the process is subjective and varies from one operator to another. The error and the so-called artifacts are introduced to the analysis.

... We also agree that it is critical to use homologous landmarks in landmark-based GM. Gesneriaceae is a family in the order Lamiales. In plant systematics, the Lamiales flower is gamopetalous (has lobe part and tube part) and consist of two dorsal petals, two lateral patels, and one ventral petal. The petal position (or the petal identity) can be identified according to the dorsal staminoid (the Lamiales flowers are alternipetalous and the dorsal stamen is underdeveloped and infertile). Thus, homologous anatomical features can be easily identified. The selected landmarks are also homologous.

... [1] Wang YH, Hsu HC, Chou WC, Kuo YF. Automatically Identifying floral contours and vascular bundles in 3D images. In 2018 ASABE Annual International Meeting, 2018. (p. 1). American Society of Agricultural and Biological Engineers.

Some minor points:
a)At the beginning, I was confused about the "shape" and "form". It got clear after reading several pages. It would be nice to clarify a bit at the beginning.

Powered by Editorial Manager® and ProduXion Manager® from Aries Systems Corporation

... Author: Thanks for the comment. We annotated the form refers to shape and size together at the beginning of the Background (Lines 51 of the revised manuscript).

b)(Line 78) Although this table is a very nice literature summary, I feel it is not directly related to this study. The authors could consider to move it to supplement.

... Author: Thanks for the comment. The table was moved to supplement in the revised manuscript (Line 770).

c)(Line 141) I cannot find Appendix 1.

... Author: The irrelevant text was remove in the revised manuscript.

d)(Line 167) From this summary, it looks like the color has strong correlation with the pollination types. If the authors have the 2D images as well, additional analysis to associate color and pollination types can be done.

... Author: Thanks for the insightful comment. Indeed, the previous study, Perret et al. (2003), indicated that, apart from the corolla shape, the corolla color would be one of the important factor corresponding to the pollination type. In this study, we focus on the variation of corolla shape and aim to identify its shape trait in 3D. We removed the color information in the revised manuscript.

... We have other in-progress research related to flower color and color pattern on these species (Fig. R2). The preliminary results showed that the corolla color and color pattern are also complex and their association with pollination type is not straightforward and not intuitive (Fig. R3). We would like to publish the finding on the corolla color as an independent research article.

... (Please find the "Person cover [response to reviewer]" for the figure)
... Figure R2. Foreground-background separation for ventral petals. The upper panel is the original image. The bottom panel is the foreground-background separated mask, the white region is foreground, and the black region is background.

... (Please find the "Person cover [response to reviewer]" for the figure)
... Figure R3. (a) Foreground color, (b) background color, and (c) contrast of the variegated species. Red: hummingbird-pollinated species; Blue: Bee-pollinated species.

e)(Line 200) No indication of "the average" in the figure.

... Author: The Fig. 4 were updated in the revised manuscript (Lines 225 and 229).

f)(Line 204) Fig 3B was not mentioned anywhere in the manuscript.

... Author: The figure was removed accordingly (Fig. 4 of the revised manuscript, Line 229).

g)(Line 274) I know "shape scores" will be explained in the method section later, but it appeared too suddenly here. Please either explain a bit here or guide readers to the method section.

... Author: Thanks for the comment. The manuscript was revised accordingly (Line 315-316 of the revised manuscript).

h)(Line 276), I know $p=0.0625$ is closed to 0.05, but instead of "significant", people normally say it is not statistical significant

... Author: Thanks for the insightful comment. Per your comment on the sample size, we increased the number of specimen from 75 to 153 and re-analyzed the evolutionary allometry. The $p = 0.0031$ in the revised manuscript (Lines 304-306).

i)(Line 305) the p value is only for the tube curvature.

	... Author: Thanks for the comment. The manuscript was revised accordingly (Line 335-336 of the revised manuscript).<br><br>j)(Line 310) Cannot see the last column of the Table 3.<br><br>... Author: The column size was adjusted (Line 339 of the revised manuscript). The full table 2 was also submitted separately of the file inventory for this manuscript.<br><br>k)(Line 350) How to explain the difference of pollination types at nodes 10 and 12 between shape and form?<br><br>... Author: Thanks for the insightful comment. Due to the analyses of shape and form were based on the sPCs and fPCs, the difference between two PCs was expected to be the size variation. Thus, the intuitive explanation for the difference of pollination types at nodes 10 and 12 between shape and form would be the size difference (Line 380-381 of the revised manuscript).<br><br>l)In the method section, authors used different number of permutations (e.g. 10,000 times, 1000 times, 100 times) for different methods. I just wonder how authors picked these numbers.<br><br>... Author: The reshuffle times are increased to 10,000 to make p-value having four decimal places of precision for the three permutation tests in the revised manuscript. The relevant information was updated in the revised manuscript (Line 522, 569, and 599).
**Additional Information:**	
**Question**	**Response**
Are you submitting this manuscript to a special series or article collection?	No
**Experimental design and statistics**<br><br>Full details of the experimental design and statistical methods used should be given in the Methods section, as detailed in our Minimum Standards Reporting Checklist. Information essential to interpreting the data presented should be made available in the figure legends.<br><br>Have you included all the information requested in your manuscript?	Yes
**Resources**<br><br>A description of all resources used, including antibodies, cell lines, animals and software tools, with enough information to allow them to be uniquely identified, should be included in the Methods section. Authors are strongly encouraged to cite Research Resource	Yes

Identifiers (RRIDs) for antibodies, model organisms and tools, where possible.<br><br>Have you included the information requested as detailed in our Minimum Standards Reporting Checklist?	
**Availability of data and materials**<br><br>All datasets and code on which the conclusions of the paper rely must be either included in your submission or deposited in publicly available repositories (where available and ethically appropriate), referencing such data using a unique identifier in the references and in the “Availability of Data and Materials” section of your manuscript.<br><br>Have you have met the above requirement as detailed in our Minimum Standards Reporting Checklist?	Yes

Manuscript

Click here to view linked References

Click here to access/download;Manuscript;17Genus Sinningia 3D GM_v5.9_submitted.docx

3D revelation of phenotypic variation, evolutionary allometry, and ancestral states of corolla shape: a case study of clade Corytholoma (subtribe Ligeriinae, family Gesneriaceae)

Hao-Chun Hsu, Wen-Chieh Chou, Yan-Fu Kuo*

Department of Biomechatronics Engineering, National Taiwan University, Taipei, Taiwan

*** Correspondence:** Dr. Yan-Fu Kuo, Department of Biomechatronics Engineering, National Taiwan University, No. 1, Sec. 4, Roosevelt Rd. Taipei, 106, Taiwan. Phone: +886-2-33665329; Fax: +886-2-23627620; E-mail: ykuo@ntu.edu.tw.

Abstract

Background:

Quantification of corolla shape variations helps biologists to investigate the diversity and evolution in plants. This study applied X-ray microcomputed tomography (μCT) to acquire three-dimensional (3D) structures of the corollas of clade Corytholoma. After acquiring volumetric images of the corollas and extracting a set of 415 3D landmarks from each volumetric image, the major 3D shape and 3D form variations of the corollas were identified from the landmarks by using geometric morphometrics (GM). Evolutionary allometry of the corolla shape was assessed. Morphological traits corresponding to the major shape variations were also defined and quantified and were subsequently used to examine their association with pollination type and to evaluate the phylogenetic signals. The landmarks were further used to reconstruct corolla shapes at the ancestral states.

Results:

GM results revealed that the first four principal components (PCs) in the 3D shape and 3D form analyses, respectively, accounted for 87.86% and 96.34% of the total variance. The centroid sizes of the corollas only accounted for 5.46% of the corolla shape variation, suggesting that the evolutionary allometry was weak. The four morphological traits corresponding to the four shape PCs were defined as tube curvature, lobe area, tube dilation, and lobe recurvation. Tube curvature and tube dilation were strongly associated with the pollination type and contained phylogenetic signals in clade Corytholoma.

Conclusions:

With the integration of µCT imaging into GM, the proposed approach boosted the precision in quantifying corolla traits and improved the understanding of the morphological traits corresponding to the pollination type, impact of size on shape variation, and evolution of corolla shape in clade Corytholoma.

Keywords: Corytholoma, Corolla shape variations, Evolutionary allometry, Geometric morphometrics (GM), generalized Procrustes analysis (GPA), Ligeriinae, X-ray micro-computed tomography (µCT)

Background

The variation in corolla shapes and forms (i.e., shape and size together [1]) in angiosperms has received considerable research attention [2, 3]. This variation was believed to be principally attributed to the specialization in animal-mediated pollination. Particularly, the species in clade Corytholoma of subtribe Ligeriinae (family Gesneriaceae) yield flowers with assorted shapes (tubular, funnel, and bell-shaped; Fig. 1) and various sizes (1–9 cm in length) and are associated with different pollinators [4]. Because of the rapid change in optimized corolla morphologies in a monophyletic group, the corollas serve as excellent materials for studying pollinator association and identifying the shape transition of the corollas. As corollas are complex three-dimensional (3D) objects, an approach should be developed for appropriately assessing their shape and size. This study applied X-ray micro-computed tomography (µCT) and 3D geometric morphometrics (GM) [5, 6] for identifying the major shape and form variations of the corollas, revealing the association between the corolla shape and pollination type, and elucidating the evolution of corolla shape in clade Corytholoma.

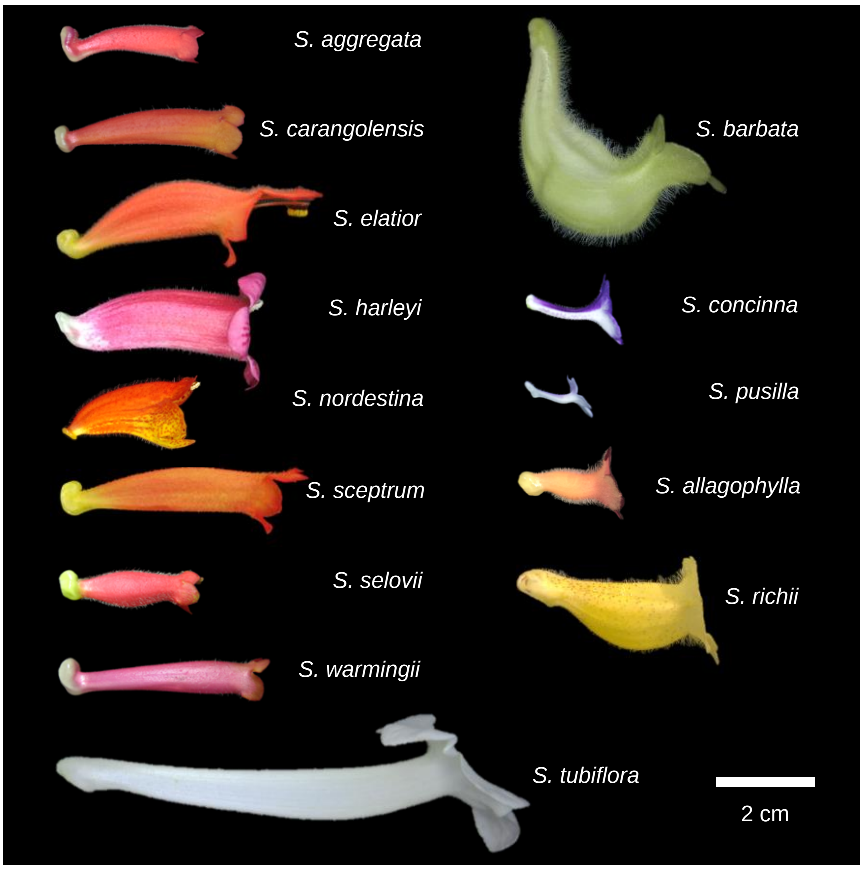

Figure 1. Side-view of the corolla of the species in clade Corytholoma.

In the last decade, landmark-based GM has been frequently applied to quantify shape and form variations of corollas (Table S1). Through landmark-based GM, the spatial variations of corolla landmarks (i.e., characteristic points of interest) can be extracted using generalized Procrustes analysis (GPA) [7, 8] and the major variations between the landmarks can be summarized using dimensionality reduction techniques (e.g., principal component analysis [PCA] or linear discriminant analysis [LDA]). Landmark-based GM can be two-dimensional (2D) or 3D. In studies using 2D GM, the identification of major shape and form variations was restricted by the imaging views (e.g., side, face, or dissected) of the corollas. However, corollas are objects with a complex 3D geometry. None of the three views provides complete information of the corolla structures [9]. This shortcoming can be overcome by combining 3D imaging techniques with GM [10]. In studies using 3D GM [9, 11, 12], the structural information of the whole corollas was comprehensively captured and retained.

The 3D corolla information can promote the specificity for studying the allometry of corolla shape. Allometry refers to the change in target traits in response to the change in size [13]. Conventionally, allometry studies are limited to investigating the relationship between two distance-based measurements, such as lengths and widths, of objects [14]. After the emergence of GM techniques, some studies have investigated the allometry of corollas in the geometry aspect using 2D images [15, 16]. However, these studies still faced the aforementioned shortcoming that 2D images inadequately capture the structural information of 3D objects. Additionally, 2D images are usually acquired manually, which may introduce error or artifacts due to inconsistent adjustments during the operation. Therefore, the allometry of corolla shape can be assessed more accurately and comprehensively by utilizing the precision and integrity of 3D imaging.

The major shape variations of the corollas identified through GM can also help in examining the association between corolla shape and pollinators. Corolla shape is one of the most prominent indicators associated with pollinator type [17, 18]. Conventionally, distance-based traits (e.g., diameter of corolla orifices and length of corolla tube) were used to evaluate pollination association [19-21]. However, distance-based traits are typically proposed based on manual observation and can be subjective. Additionally, these traits could be oversimplified and may not adequately describe the geometric properties of the corollas [1, 22, 23]. By contrast, the shape variations obtained using GM were identified through a serious of statistical procedures; thus, they could adequately represent the principal shape differences among the corollas. Gómez et al. [24] and Kaczorowski et al. [25] used the corolla shape variations quantified using 2D GM to examine the association between plant species and pollinators in *Erysimum* and *Nicotiana*, respectively. Traits identified using 3D

GM precisely describe the leading variations in the geometric properties of corollas; thus, they can serve as excellent candidates in the tests of pollinator association.

Corolla shapes at the ancestral states is another intriguing research topic for biologists. To infer history and interpret the evolution of species, the characteristics of the species and their transitions along phylogeny are reconstructed and evaluated [26, 27]. The corolla shapes at the ancestral states can be reconstructed using a given phylogeny and corolla landmarks of the extant species [28]. Gómez et al. [29] reconstructed the corolla shapes in *Erysimum* and visualized the changes in shape at the ancestral states using GM and 2D landmarks in the face view. Joly et al. [30] identified the evolutionary constraints on corolla shape in Gesneriaceae using GM and 2D landmarks in the side view. The corollas reconstructed using face or side views only provide a part of structural information of the corollas. By contrast, the corolla reconstructed using 3D landmarks shows complete structural information. Thus, 3D images may reveal more information regarding the transition of the corolla shapes at the ancestral states.

This study scrutinized the 3D corolla shapes and forms of the species in clade Corytholoma. We used the 3D approach to acquire the images of the corollas; thus, the complete structural information of the corollas was retained. We selected 415 landmarks for each corolla; thus, the structures of the corollas were genuinely represented. We performed GM analyses on the landmarks to identify both the 3D shape and 3D form variations of the corollas; thus, the impact of corolla size on corolla shape could also be examined. We defined morphological traits of the corollas based on the GM results and quantified the traits directly using the 3D corolla images; thus, the traits were proposed statistically rather than manually. The proposed traits were subsequently used for investigating the association between pollination type and corolla shapes; thus, the leading shape variations could be used and interpreted in the association tests. We further evaluated the phylogenetic signals of corolla

size and morphological traits; thus, the tempo and mode of corolla evolution could be assessed. Last, we reconstructed corolla shapes at the ancestral states using 3D landmarks; thus, more information regarding the shape transition of the corolla could be revealed.

Data Description

Flower materials

The germplasms of 15 species (Table 1) in clade Corytholoma were obtained from Dr. Cecilia Koo Botanic Conservation Center (KBCC), Pingtung, Taiwan, and were maintained by establishing inbred lines. The plant individuals were cultivated under natural lighting, 70%–80% humidity, and at 22–28°C in a greenhouse (Technology Commons X, College of Life Science, National Taiwan University, Taiwan). Six to sixteen flowers were collected from two to five plant individuals of each species between August 2015 and August 2016, resulting in a total of 153 specimens (Table S2). The specimens of the same species were collected in the same flowering season to alleviate the shape variations caused by different flowering seasons. The Corytholoma species are protandrous, which means the anther matures before the stigma. To minimize the developmental variations, the collection was conducted at the developmental stage between anther and stigma anthesis. The specimens were prepared in fresh or were fixed in 70% ethanol solution (Table 1).

All the 153 specimens were used in the analyses of 3D shape and form variations and of evolutionary allometry. To avoid being dominated by the species with larger sample size, five specimens with shape scores nearby the median of each species were selected. These specimens (a total of 75) were used in the analyses of phylogenetic signals and of ancestral state reconstruction (see Methods for the calculation of shape score and see Table S2 for the specimen information).

Table 1. Species list and dimension of the slice images.

Species	Pollination type [a]	Specimen type [b]	KBCC and inbred line accession
S. aggregata	H	F	K039091, K039092, K039093
S. allagophylla	H	F	K039099, L039110, HC0909-d
S. barbata	B	E/F	K039104, K039105, HC1206-a, HC1206-d
S. carangolensis	H	F	K039112, HC1912-2, HC1912-b
S. concinna	B	F	K039117, K039118, HC2202-t
S. elatior	H	E	K039126, K039127, K039129, K039131
S. harleyi	H	F	K039135, HC3403-3, HC3403-8
S. nordestina	H	F	K039168, HC5504-1, HC5504-3
S. pusilla	B	F	K039169, K039170, K039171, K039172, HC5803-2
S. richii	B	E/F	K039174, K039175, K039176, K039177
S. sceptrum	H	E	K039178, K039179, K039181
S. sellovii	H	E	K039184, K039185, K039186
S. tubiflora	M	F	K039197, K039198, K039199, K039200, K039201
S. valsuganensis	H	F	K039203, K039204
S. warmingii	H	E	K039205, K039209, K039216

[a] H: hummingbird pollination (ornithophily), B: bee pollination (melittophily), and M: moth pollination (phalaenophily).
[b] The letter E denotes the 70% ethanol-fixed specimen, and the letter F denotes the fresh specimen.
[c] The 3D images with a slice size of 1968 × 1968 were downsized to 984 × 984 before the reconstruction of volumetric and surface images. The identified landmarks were then magnified back to the original scale for the subsequent GM analysis.

The information on pollination types was obtained from Perret et al. [4]. The species were associated with three pollination types: hummingbird, bee, and moth (Table 1). The hummingbird-pollinated species have tubular corollas (Fig. 1). The bee-pollinated species have campanulate or salverform corollas. The moth-pollinated species have narrow and long tubular corollas.

3D flower image data

The 3D images of the flowers were acquired using an X-ray µCT scanner (SkyScan 1076, Bruker; Kontich, Belgium). The spatial resolution of the scanner was 36.547 µm in each dimension (Table S2). A 3D image was composed of hundreds or thousands of 2D slice images along the longitudinal axis. In each 2D slice image, image thresholding, morphological operation, and connected component labeling were performed to reduce the noise of the images and to separate the region of corolla from the background [9]. The processed 2D slice images of the same specimen were then integrated into a 3D volumetric image [9] (Fig. 2B). The volumetric images were next converted into surface images [9] (Fig. 2C), in which the surfaces of the corollas were covered by triangular meshes. The surface images were saved in polygon (PLY) file format for the subsequent landmark identification [9]. The 2D slice images, volumetric images, and the surface images of the specimens are available in the GigaScience database repository [GigaDB number]. The demonstration video of the program for generating a 3D volumetric image from 2D slice images can be found in [9].

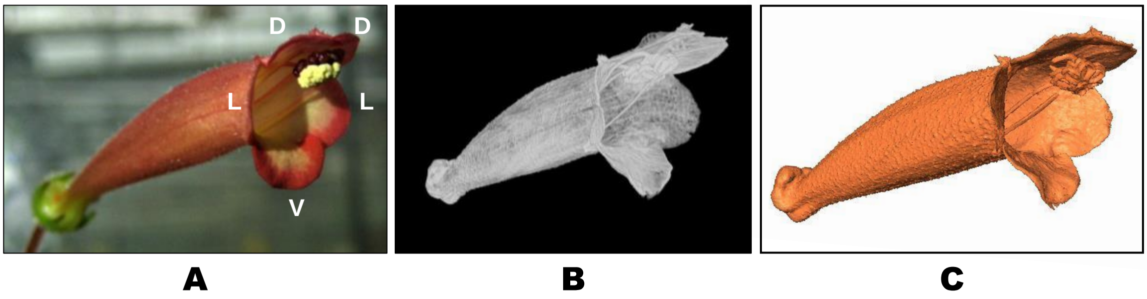

Figure 2. (A) Photograph, (B) volumetric image, and (C) surface image of a corolla of *S. sceptrum*. D denotes dorsal position, L denotes lateral position, and V denotes ventral position.

Landmark identification

Landmarks were defined based on the homologous and anatomically recognizable features of the corollas. The corollas of the Corytholoma species consist of two dorsal, two lateral, and one ventral petals (Fig. 2A). A petal has a lobe region (hanging part) and a tube region (part connecting to other petals). A petal also has a three-nerved venation extending from the proximal end of the tube region to the distal end of the lobe region. The homologous features of a petal (Fig. 3) include the intersections of adjacent lobes (white point with roman letter I), the contour of lobe (solid line), the petal midrib (main vein; round dot line), the lobe–tube connected rims (square dot line), and the tube–tube connected rim (dash line). Twenty five primary landmarks (roman letters in Fig. 3) were defined based on the features, including five intersections of adjacent lobes (I), five proximal points of petal midribs (II), five distal points of petal midribs (III), five intersections of the lobe–tube rims and petal midribs (IV), and five proximal points of the tube–tube rims (V). Three hundred and ninety secondary landmarks were defined as 15 equally distributed points on each lobe contour, 7 equally distributed points on each lobe–lobe rim, 7 equally distributed points on each lobe midrib, 25 equally distributed points on each tube midrib, and 25 equally distributed points on each tube–tube rim.

The landmarks were selected semi-automatically from the 3D surface images. The selection of the intersections of adjacent lobes, the contours of lobe–tube connected rims, and the contour of tube-tube connected rim were performed manually using a software Landmark [9, 12, 31]. The contours and midribs were pre-identified automatically using a software developed by authors' team [32]. The secondary landmarks were then automatic determined based on the selected contours, midribs, and rims using a program developed in MATLAB (The MathWorks, Natick, MA, USA) [9]. The landmarks of the specimens in this study are available in the GigaScience database repository [GigaDB number].

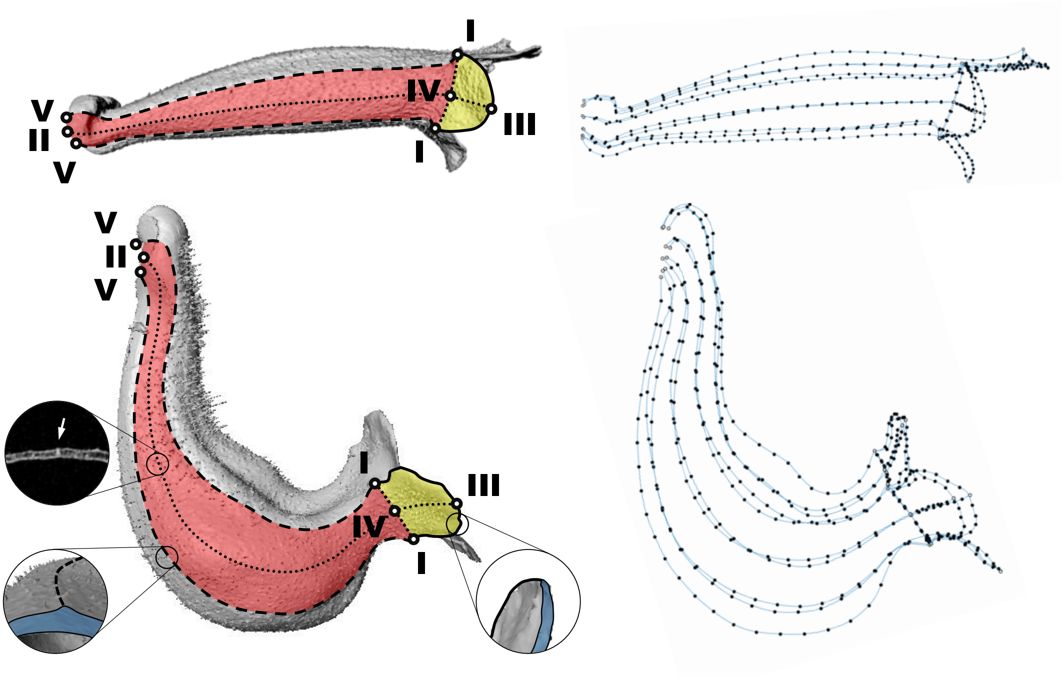

Figure 3. Landmarks of *S. sceptrum* (top panel) and *S. barbata* (bottom panel). A total of 415 landmarks, including 25 primary (Roman numbers) and 390 secondary, were identified on each corolla based on the homologous features of the petals. White point with roman letters: the intersections of adjacent lobes, solid line: the contour of lobe, round dot line: the contour of petal midrib, square dot line: the contours of lobe–tube connected rims, dash line: the contour of tube-tube connected rim, I: the intersections of adjacent lobes, II and III: the proximal and distal points of petal midribs, IV: the intersection of the lobe–tube rims and midribs, and V: the proximal points of the tube–tube rims. Yellow shade denotes lobe region; red shade denotes tube region. White arrow indicates the location of midrib in the 2D slice image.

Analyses

Corolla centroid size

The centroid sizes of the corollas of the Corytholoma species are illustrated in Figure 4. The figure shows that the corolla sizes of *S. tubiflora* were much greater than the average. The within-species variance of the corolla size of the *S. barbata* and *S. tubiflora* was larger than that of other species.

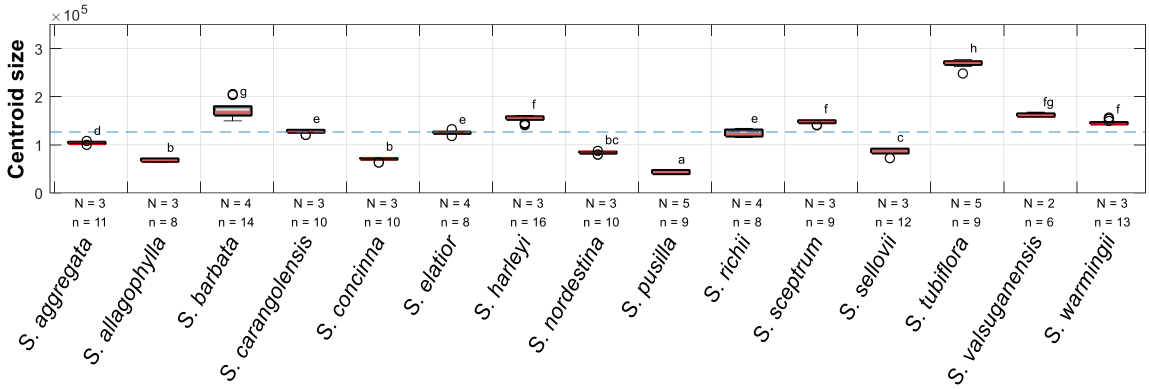

Figure 4. Centroid sizes of the corollas of the extant species. The blue dot line denotes the average centroid size. The lowercase alphabets at the right up of the box plots denote groups of Scheffé's multiple comparison tests performed with a confidence level of 0.99. The letter N denotes the number of plant individual; the letter n denotes the number of specimen.

Major 3D shape and form variations of the corollas

Major 3D shape variations among the flowers were identified using the full-GPA GM procedure described in the Methods section. The first four shape principal components (PCs), referred to as shape PC1 (sPC1) to sPC4, accounted for 52.57%, 21.39%, 7.99%, and 5.91%, respectively, of the total variance. Figure 4A illustrates the major shape variations using virtual flowers. The virtual flower of the mean sPC values is illustrated in grey, and the virtual flowers with sPC values of mean ± 2 standard deviation (SD) are illustrated in pink.

The four sPCs were linked to four specific shape transitions. sPC1 primarily corresponded to tube curvature. The tube of the corolla with a small sPC1 value was bent upward at a considerable degree (Fig. 5A). By contrast, the tube of the corolla with a large sPC1 value was bent downward. sPC2 principally corresponded to the lobe area size. The line connecting landmarks L4–L5 separates the lobe (right) and tube (left). The corolla with a small sPC2 value had a larger lobe area than that with a large sPC2 value. Particularly, the lobe area of the corolla with a sPC2 value of mean + 2 SD was nearly absent. sPC3 particularly corresponded to tube dilation (the distance between landmarks T14 and M14).

The corolla with a small sPC3 value dilated in the tube, whereas the corolla with a large sPC3 value shrank in the tube. sPC4 principally corresponded to lobe recurvation. The lobe midrib (the line connecting landmarks M27–M35) of the corolla with a small sPC4 value was bent outward. By contrast, the lobe midrib of the corolla with a large sPC4 value was almost parallel to the tube midrib (the line connecting landmarks M1–M27).

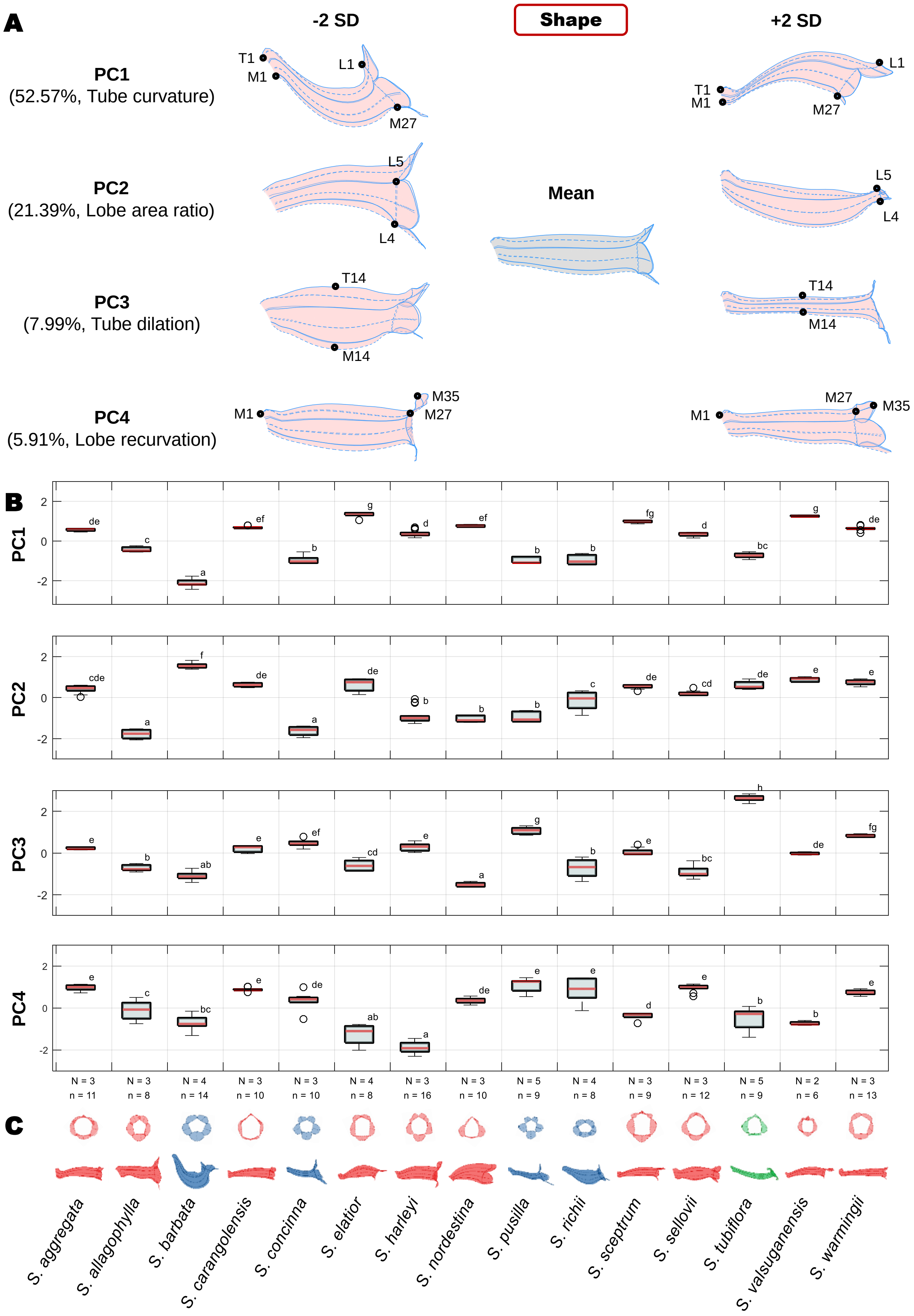

Figure 5. Major 3D shape variations of the Corytholoma flowers: (A) virtual flowers with an sPC value of mean ± 2 SD, (B) distributions of the sPC scores, and (C) mean corolla shapes. In (A), black dots represent labeled

landmarks. L, M, and T represent landmarks on lobe contour, midrib, and tube–tube rim, respectively. In (B), the sPC scores are standardized to zero mean and unit variance. In (C), the corollas are colored by pollination type. Red, blue, and green represent hummingbird-pollinated, bee-pollinated, and moth-pollinated species, respectively. The lowercase alphabets at the right up of the box plots denote groups of Scheffé's multiple comparison tests performed at a confidence level of 0.99. The letter N denotes the number of plant individual, and the letter n denotes the number of specimen.

Figures 5B and 5C illustrate the distributions of the sPC scores and the face and side views of the mean corolla shape for each species. The sPC scores were standardized to zero mean and unit variance. Note that the within-species variance of sPC scores in most species increased from sPC1 to sPC4 (Fig. S1A). Particularly, the within-species variances of species *S. allagophylla*, *S. elatior*, *S. richii*, and *S. tubiflora* in sPC4 were larger than those of the other species.

Major 3D form variations among the flowers were identified using the partial-GPA GM procedure. The first four form PCs, referred to as form PC1–form PC4 (fPC1–fPC4), accounted for 69.38%, 19.90%, 4.43%, and 2.63%, respectively, of the total variance. Figure 6A illustrates the major form variations using virtual flowers. fPC1 primarily corresponded to the corolla size. The corolla with a small fPC1 value had a large corolla size, whereas the corolla with a large fPC1 value had a small corolla size. In fact, fPC1 was negatively correlated with centroid size ($r = -0.9952$; Fig. 7A) and accounted for 73.69% of the total form variation. Notably, fPC2, fPC3, and fPC4, respectively, correlated with sPC1, sPC2, and sPC3 ($r = 0.9571$; $r = 0.7858$; $r = 0.5470$; Fig. 7B–7D). However, *S. tubiflora* and *S. harleyi* did not follow the sPC2–fPC3 and sPC3–fPC4 correlations, respectively. The correlation coefficients increased considerably when these two species were excluded from the analyses ($r = 0.9222$ and $r = 0.8274$; Fig. 7C and 7D).

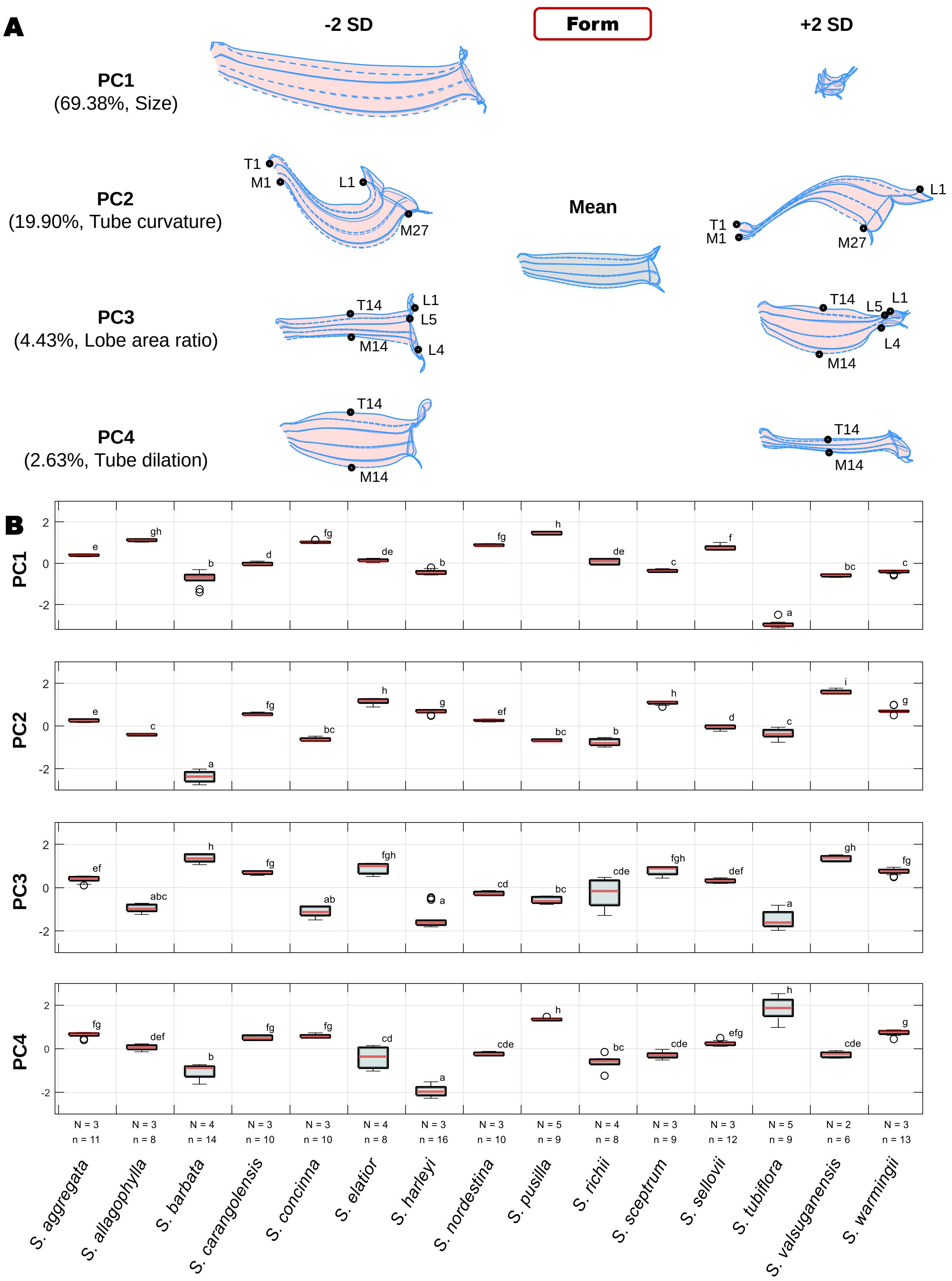

Figure 6. Major 3D form variations of the Corytholoma flowers: (A) virtual flowers with an fPC value of mean ± 2 SD and (B) distributions of the fPC scores. In (A), black dots represent labeled landmarks. L, M, and T represent landmarks on lobe contour, midrib, and tube–tube rim, respectively. In (B), the fPC scores are

standardized to zero mean and unit variance. The lowercase alphabets at the right up of the box plots denote groups of Scheffé’s multiple comparison tests performed at a confidence level of 0.99. The letter N denotes the number of plant individual, and the letter n denotes the number of specimen.

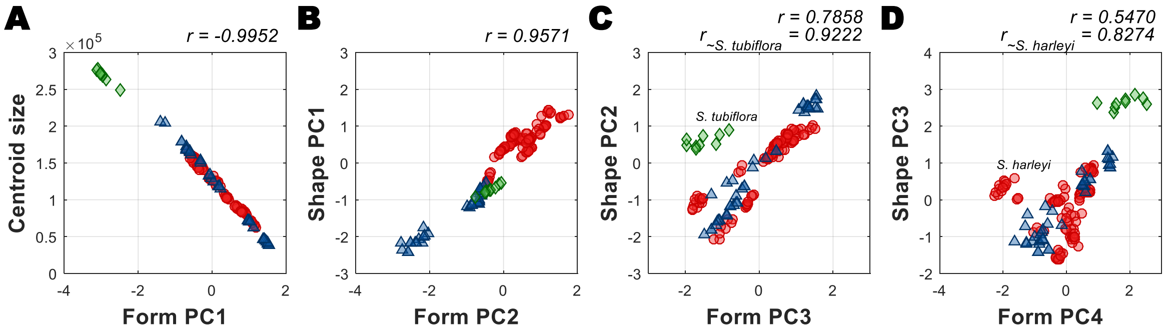

Figure 7. Analysis of correlation between the sPC and fPC scores. The PC scores are standardized to zero mean and unit variance. The correlation coefficients are provided at the upper right corners of scatter plots. Red circle, blue triangle, and green diamond represent hummingbird-pollinated, bee-pollinated, and moth-pollinated species, respectively.

Evolutionary allometry of the 3D corolla shape

The evolutionary allometry of the corolla shape was assessed. To summarize the overall shape variation, the shape scores were calculated using full-GPA landmarks and multivariate regression (see Methods for the details). The correlation coefficient of 0.2336 between the shape scores and centroid sizes revealed that the centroid size of the corollas accounted for only 5.46% of the shape variation (Fig. 8A). In addition, the permutation test indicated that the correlation was weak but statistically significant (Fig. 8B, p = 0.0031). Moreover, the low to medium levels of the correlations between sPCs and centroid size (Fig. S2) also supported that the allometry between shapes and size in Corytholoma was weak but significant.

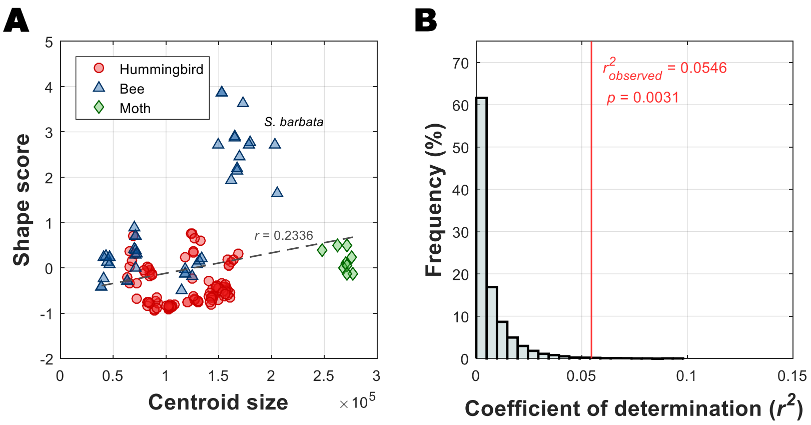

Figure 8. Evolutionary allometry of the corolla shapes in clade Corytholoma. Red circle, blue triangle, and green diamond represent hummingbird-pollinated, bee-pollinated, and moth-pollinated species, respectively.

Morphological traits and their association with pollination type

Four morphological traits—tube curvature, lobe area ratio, tube dilation, and lobe recurvation—were defined based on the variations of the first four sPCs. The traits were subsequently quantified from the 3D images of the corollas (see the Methods section for the calculation of shape scores). Correlation analyses indicated that the defined traits adequately describe the major shape variations ($r \geq 0.7376$; Fig. 9). The pairwise correlations between the morphological traits indicated that the morphological traits were weakly correlated with each other ($r \leq 0.3109$).

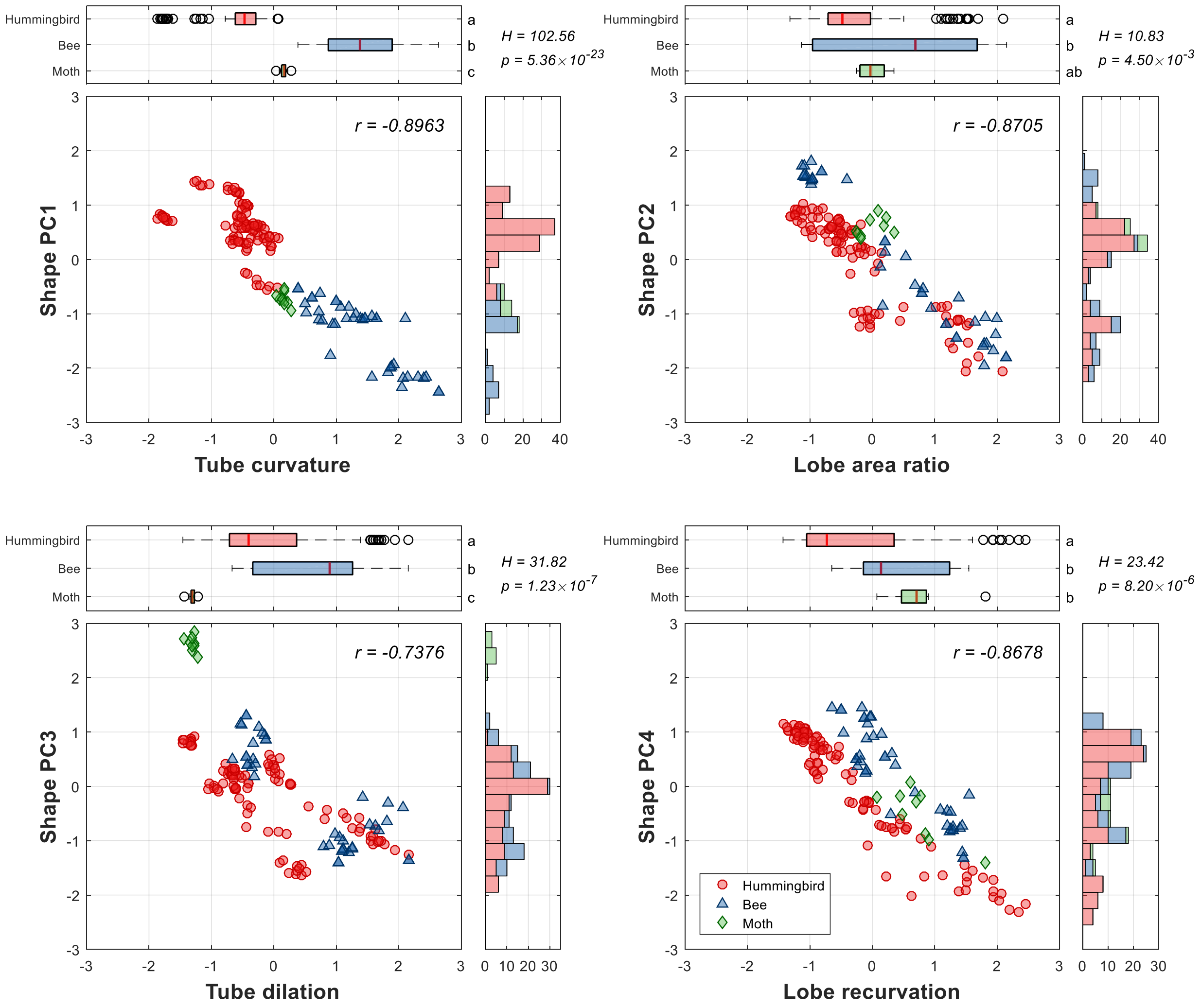

Figure 9. Scatter plots of the morphological traits and sPC scores. The trait and sPC scores are standardized to zero mean and unit variance. The correlation coefficients (*r*) are provided at the upper right corners of the scatter plots. The right panel of the boxplots presents the results of Kruskal–Wallis tests (*H* values). The lowercase alphabets at the right of the box plots denote groups of Scheffé's multiple comparison tests performed with a confidence level of 0.99.

The association between the traits and pollination types was examined. The centroid size was also included in the analyses. Kruskal–Wallis test results indicated that the four traits and centroid size significantly differed between the pollination types ($p < 4.50 \times 10^{-3}$, Table 2). Scheffé's multiple comparison test results suggested that tube curvature and tube dilation formed three clusters corresponding to the three pollination types ($p < 3.09 \times 10^{-4}$ and $p < 3.42 \times 10^{-4}$, respectively; Fig. 9; Table 2). The permutation test for logarithm of the odds

(LOD) scores indicated that the centroid size and the tube curvature was significantly associated with the three pollination types (LOD = 21.71 and LOD = 45.52, $p = 0$ and $p = 0$, respectively).

Table 2. Kruskal–Wallis test results, Scheffé's multiple comparison test results, and LOD scores of the morphological traits by pollination type.

Morphological traits	Kruskal-Wallis test		Scheffé's multiple comparison test						LOD score	*p*-value
			Hummingbird vs Bee		Hummingbird vs Moth		Bee vs Moth			
	H-value	*p*-value	*T*-value	*p*-value	*T*-value	*p*-value	*T*-value	*p*-value		
**Centroid size**	26.81	1.51×10^{-6}	1.76	2.15×10^{-1}	11.23	1.11×10^{-16}	11.49	1.11×10^{-16}	21.71	0
**Tube curvature**	102.56	5.36×10^{-23}	20.96	1.11×10^{-16}	4.13	3.09×10^{-4}	6.62	4.59×10^{-9}	45.52	0
**Lobe area ratio**	10.83	4.50×10^{-3}	3.71	1.38×10^{-3}	0.55	8.60×10^{-1}	1.34	4.08×10^{-1}	2.92	1.60×10^{-3}
**Tube dilation**	31.82	1.23×10^{-7}	4.10	3.42×10^{-4}	3.81	9.83×10^{-4}	5.66	5.06×10^{-7}	7.31	0
**Lobe recurvation**	23.42	8.20×10^{-6}	3.60	2.02×10^{-3}	2.92	1.57×10^{-2}	0.95	6.34×10^{-1}	3.91	1.80×10^{-4}

Phylogenetic signals of centroid size and morphological traits

The phylogenetic signals of the centroid size and four morphological traits were estimated (Fig. 10). The centroid size and four morphological traits of five specimens in each species were used (See Data Description for the details). Blomberg's K values of the tube curvature and tube dilation calculated using the 50% majority-rule consensus tree were 0.9250 and 0.8739, respectively. The permutation test for Blomberg's K values rejected the null hypothesis that the two traits had no phylogenetic signal ($p = 0.0408$ for tube curvature and $p = 0.0424$ for tube dilation). These observations indicated that the change in the two traits approximated the Brownian motion model, and the two traits evolved gradually through time.

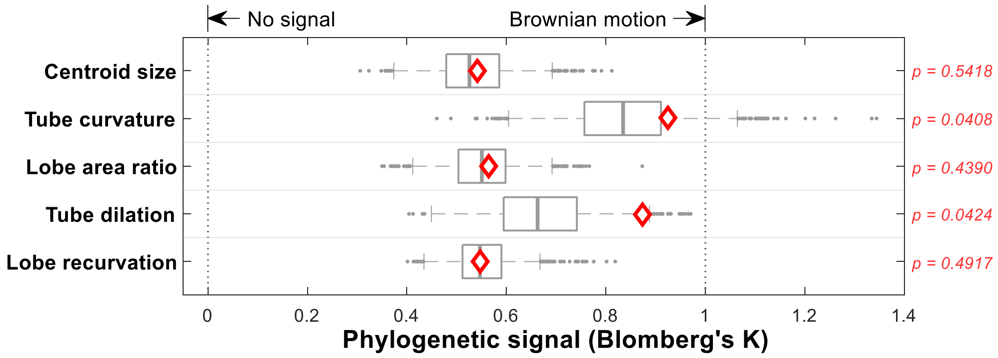

Figure 10. Phylogenetic signals of the centroid size and four morphological traits in clade Corytholoma. The distributions of the Blomberg's K values were obtained using the phylogenetic trees of 1,000 replicates in the maximum likelihood analysis. Diamonds indicate the Blomberg's K values calculated using the 50% majority-rule consensus tree. The *p* values are provided at the right of the boxplot.

3D corolla shapes and forms at the ancestral states

The 3D corolla shapes and forms at the ancestral states were reconstructed using the 50% majority-rule consensus tree (Fig. 11). For both the reconstructed shapes and forms, the corollas at nodes 1–4 and 12 bent upward, those at nodes 5–11 became straight and narrow, and those at nodes 13 and 14 bent downward in the tube. The measurement of centroid sizes and evaluation of morphological traits revealed more details on these transitions (Table S3). Centroid sizes of the corollas fluctuated from nodes 1–7 and increased gradually from nodes 8–11. Tube curvatures of the corollas decreased from nodes 1–11. Negative tube curvatures were observed on the corollas at nodes 13 and 14. Lobe area ratios of the corollas fluctuated from nodes 1–7 and decreased gradually from nodes 8–11. Tube dilations and lobe recurvations of the corollas also decreased from nodes 1–11. The decreasing trend extended to the nested nodes 12–14. The results obtained from the reconstructed corolla also indicated that the transitions in the traits were gradual.

The pollinator types of the 3D corolla shapes and forms at the ancestral states were estimated using sPC1–sPC4 and fPC1–fPC4, respectively, and the *k*-nearest neighbor

algorithm with a *k* value of 5. The shifts in pollinator types were mostly consistent in both shape and form. We observed that the corolla shapes and forms at nodes 1–4 were estimated to be bee-pollinated, and the corolla shapes and forms at nodes 5–9 and 13–14 were estimated to be hummingbird-pollinated (Fig. 11). The corolla shape at node 11 was estimated to be moth-pollinated (Fig. 11A). The corolla shapes at nodes 10 and 12 were estimated to be hummingbird-pollinated and bee-pollinated, respectively. By contrast to the shape analysis, the corolla forms at nodes 10 and 12 were estimated to be moth-pollinated and hummingbird-pollinated, respectively (Fig. 11B). The size information (i.e., the difference between shape and form) altered the pollinator types at node 10 and 12.

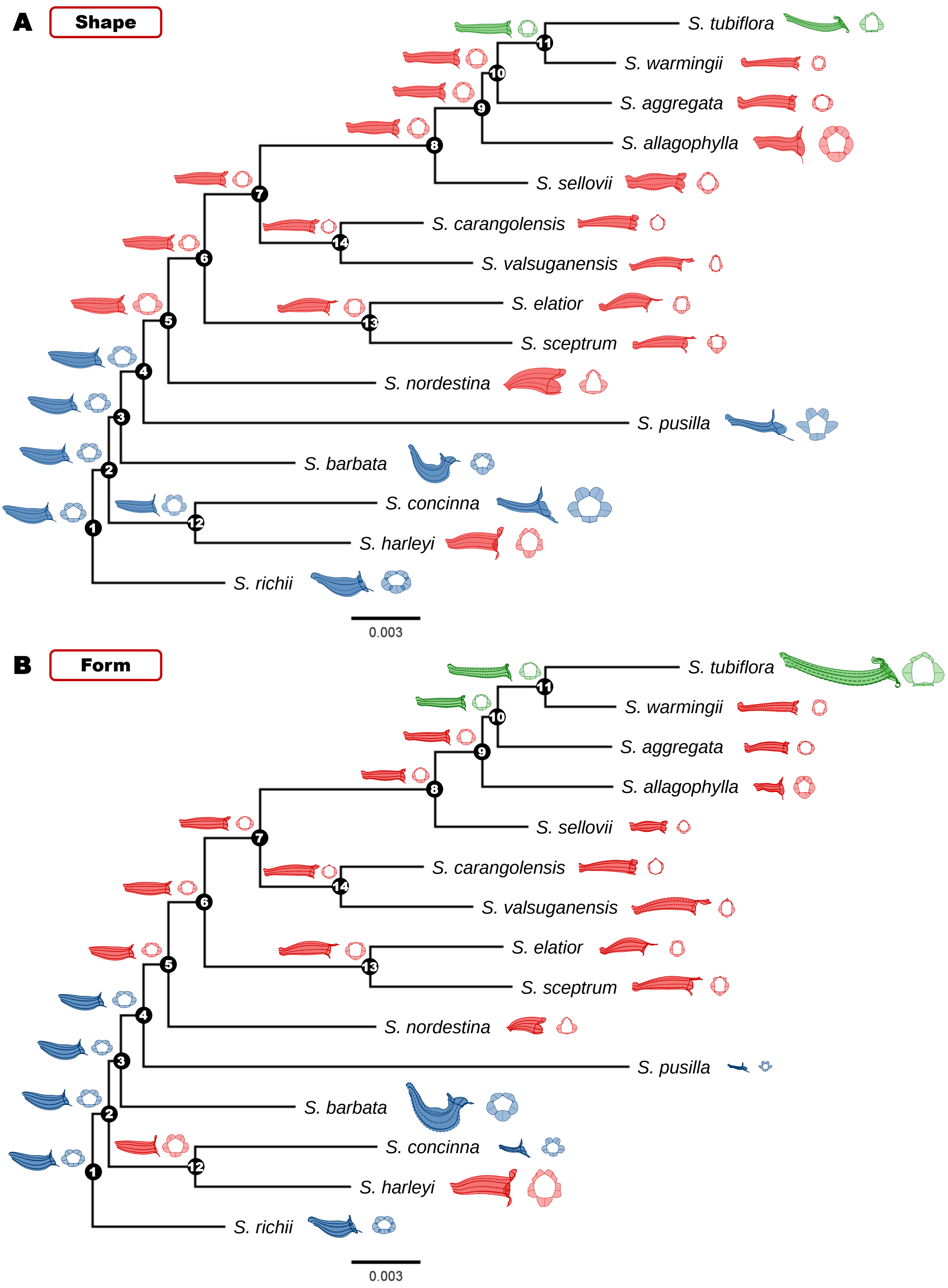

Figure 11. Reconstructed 3D corolla (A) shapes and (B) forms at the ancestral state for the Corytholoma species. The branch length indicates the number of substitutions per site, and the scale bar denotes 0.003 substitutions

per site. The corolla colors of the extent species are assigned based on the pollination type. Red, blue, and green denotes hummingbird-, bee-, and moth-pollinated species, respectively. The corolla colors of the species at the ancestral states were estimated using the *k*-nearest neighbor algorithm with a *k* value of 5.

Figure 12 presents the distributions of the extant species and ancestral states in the corolla shape and form morphospaces. In both morphospaces, *S. barbata* was approximately 3 SDs away from the neighboring ancestral state. In the morphospace of the corolla form, *S. tubiflora* was approximately 3 SDs away from the neighboring ancestral state. Both the morphospaces were sparse in the neighborhoods of *S. barbata* and *S. tubiflora* compared with those of other species.

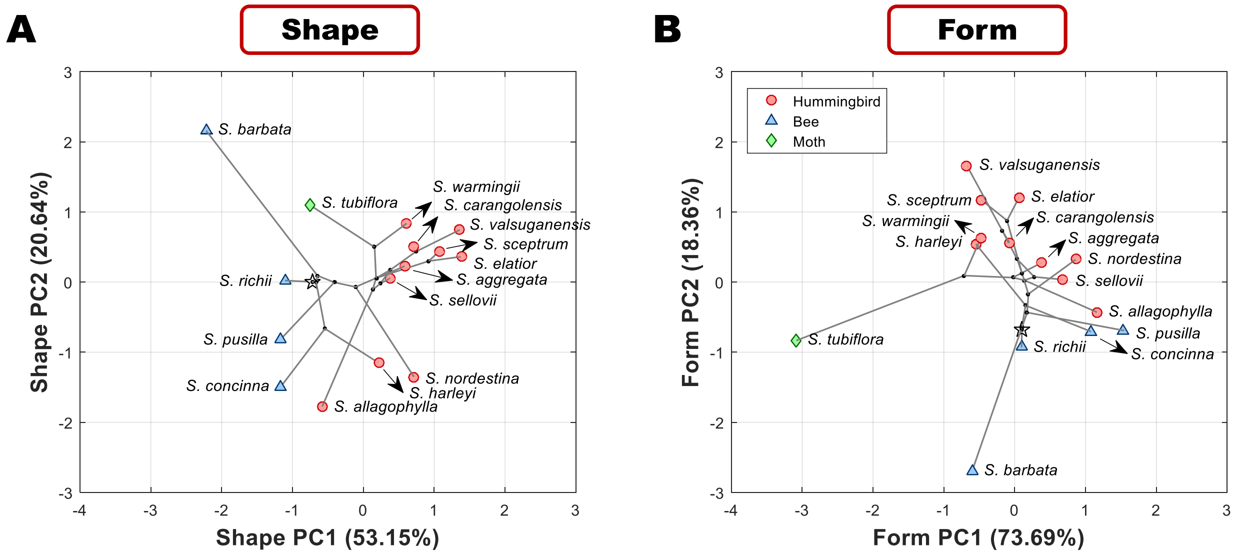

Figure 12. Distribution of the extant species and ancestral states in the morphospaces of the 3D corolla (A) shape and (B) form. The PC scores are standardized to zero mean and unit variance. White star and black points represent the ancestral state of node 1 (root) and ancestral states of nodes 2–14, respectively.

Discussion

In this study, we acquired the 3D corolla images of the Corytholoma species using µCT and identified the major 3D shape and 3D form variations of the corollas using landmark-based GM. We first revealed that the evolutionary allometry of corolla shapes was weak in Corytholoma species. According to the identified major shape variations, we defined and quantified four morphological traits—tube curvature, lobe area ratio, tube dilation, and lobe recurvation. We revealed that tube curvature and tube dilation were significantly associated with pollination type. The centroid size was also strongly associated with the pollination type. Taking together the trait values and phylogenetic information, we revealed strong phylogenetic signals in tube curvature and tube dilation. By reconstructing the corolla shapes, measuring the morphological traits at the ancestral states, and testing the phylogenetic signals of the traits, we discovered that the evolutionary changes in corolla shape were gradual in Corytholoma species.

Resemblance of virtual flowers to corollas of clades other than clade Corytholoma

The corolla shape variations identified in Corytholoma species resembled the corolla shapes of some species from other clades. The virtual flowers of mean + 2 SD in sPC1 and mean − 2 SD in sPC2 resembled the corollas of *Vanhouttea hilariana* (clade Vanhouttea, Fig. 13A) and *Sinningia insularis* (clade Dircaea, Fig. 13B), respectively. Surprisingly, some virtual flowers also resembled the corolla shapes of species of subtribes other than subtribe Ligeriinae. The virtual flower of mean + 2 SD in sPC1 (Fig. 5A) resembled the corolla of *Columnea microphylla* (subtribe Columneinae) [33]. The virtual flower of mean − 2 SD in sPC3 (Fig. 5A) resembled the corolla of *Drymonia urceolata* (subtribe Columneinae) [34].

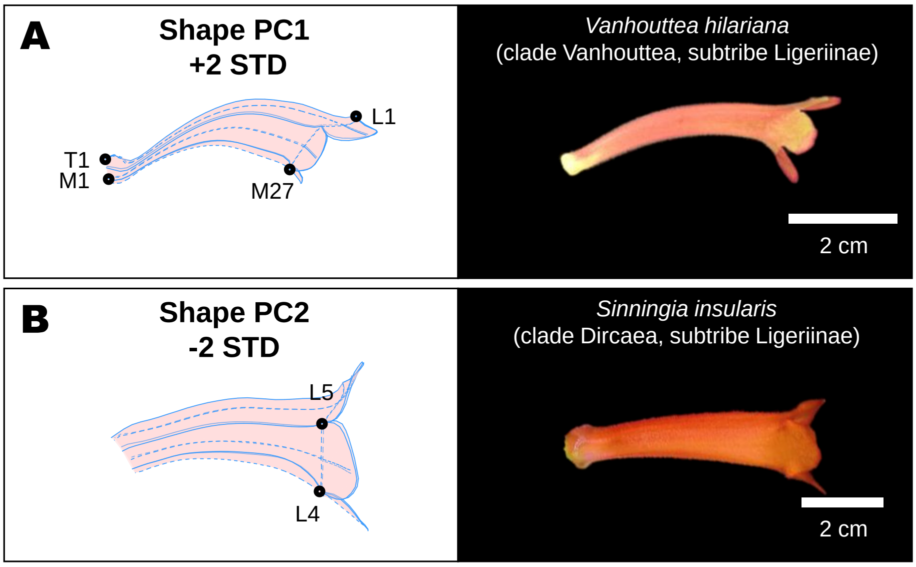

Figure 13. (A) Virtual flower of mean + 2D in sPC1 and the corolla image of *Vanhouttea hilariana* and (B) virtual flower of mean − 2D in sPC2 and the corolla image of *Sinningia insularis*.

Evolutionary allometry and morphological integration of 3D corolla shape

The association between evolutionary allometry and morphological integration was supported by the evidence found in Corytholoma. After the emergence of GM in the last century, allometry has been believed to be associated with morphological integration [23, 35]. In Corytholoma, we revealed that the size only accounted for 5.46% of shape variation, suggesting that the allometry of corolla shape was weak. The mean of squared pairwise correlation coefficients between the four morphological traits was 0.0410, which suggested that the morphological integration in corolla shape was weak. Similar to the findings in a study of skulls of birds [36], the aforementioned evidence indicated that evolutionary allometry and morphological integration are associated at a certain level. The present study suggests that the corolla shape in Corytholoma can serve as an example for the association between evolutionary allometry and morphological integration.

Divergent corolla dominated the analysis of 3D shape and 3D form variation

Virtual flowers should be cautiously used to interpret the major shape and form variations of the corollas when species with extreme shapes or forms are included or when the species are sparse in the morphospace. The corolla shape and form of the Corytholoma species varied largely. In some studies on closely related species, the ranges of the PC scores were usually less than three SDs [11, 29]. By contrast, the PC scores in the present study spanned up to five SDs (Fig. 12). In the morphospace, *S. tubiflora* and *S. barbata* were distant from the clusters of the other species. Moreover, the space among *S. tubiflora*, *S. barbata*, and the cluster of the other species was large. The GM analysis reveals the major shape and form variations by applying linear interpolation or extrapolation to the landmarks of the species being studied (reviewed in [23]). Thus, the identified variation in the analysis could be considerably influenced by *S. tubiflora* and *S. barbata*. In other words, the divergent corolla shape of *S. tubiflora* and *S. barbata* would deviate the interpolated or extrapolated virtual flowers from reality. The most obvious example was the virtual flower of mean + 2 SD in fPC1 (Fig. 6A). The proximal part of the corolla protruded from the throat of the tube such that the corolla was inside out. Mean + 2D in fPC1 was beyond the cluster of the species in Figure 12B. No corolla physically existing in nature can resemble such a virtual flower.

Use of GM-derived morphological traits for phenotyping

Defining appropriate traits that correspond to shape variations is crucial for phenotyping. Conventionally, traits were defined based on researchers’ observations and were quantified manually using calipers. After the emergence of GM and imaging techniques, some studies [16, 25, 37] have used PC scores from the GM analysis as the traits for phenotyping. Although comprehensive, PC scores were so complex that they could not specify the key changes in shape. We defined morphological traits by observing the major shape variations

identified in the GM analysis. Subsequently, the traits were automatically measured from the 3D landmarks of the corollas. The proposed approach helped us objectively define and precisely quantify traits corresponding to major 3D shape variations.

Limitations of 3D analysis of corolla shape evolution

Although we believe that this is one of the pioneer studies to integrate the phylogenetic and 3D information to speculate corolla shape evolution, the results may be affected by phylogenetic uncertainty [38]. Phylogenetic uncertainty is majorly increased due to two issues: inconsistent tree topologies or branch lengths and incomplete sampling of extant species. We used the 1,000 phylogenetic trees obtained from each replicates for evaluating the phylogenetic signals. Therefore, the bias caused by relying on a single phylogenetic tree was avoided. The flower specimens of two species, *S. brasiliensis* and *S. aghensis*, with unique corolla shapes were not included in this study. In addition, the molecular sequences of newly added Corytholoma species [39], *S. helioana* and *S. muscicola*, were partly published. The topology of the phylogeny is data-dependent and could have been altered if these species were included. However, inclusion of these specimens and sequences would have made yielded more complete results of the analysis of corolla shape evolution.

Methods

Major 3D shape and 3D form variations of the corollas

The major 3D shape and 3D form variations of corollas were identified from the landmarks obtained using GM. The major shape variations were determined using full-GPA [40]. Full-GPA removed the geometric information of the corollas related to translation, rotation, and scaling. The major form variations, defined as the combination of shape and size variations, were determined using partial-GPA. Partial-GPA removed the information of the corollas related to translation and rotation only. Following full- or partial-GPA, PCA was applied to the resulting landmarks. The obtained PCs were referred to as sPCs and fPCs. The first four sPCs and fPCs accounted for the majority of the variance and were used for representing the major 3D shape and 3D form variations between the corollas, respectively. Virtual flowers were created to visualize the major 3D shape and 3D form variations. The virtual flowers were obtained by performing an inverse PCA transform on a PC scores [9].

Evolutionary allometry of the 3D corolla shapes

The evolutionary allometry of the 3D corolla shapes in Corytholoma was evaluated using a multivariate regression analysis [41], correlation analysis, and permutation test [42]. In the multivariate regression analysis, regression coefficients were estimated using the full-GPA landmarks as the response variables and the centroid size as the predictor variable [41]. A shape score of the specimen was then obtained as the inner product of the full-GPA landmarks of the specimen and the vector of the regression coefficients. Subsequently, the correlation between the shape scores and centroid sizes was calculated. The square of the correlation coefficient indicated the degree of the size variation that accounted for the shape variation. Subsequently, the permutation test was performed to evaluate the dependency of the shape score on the centroid size. In the permutation test, the pairs of full-GPA landmarks

and their associated centroid size were reshuffled among all the specimens 10,000 times. In each shuffle, the aforementioned multivariate regression analysis and correlation analysis were performed using the shuffled data to obtain a correlation coefficient. The accumulated squares of the correlation coefficients obtained from the 10,000 shuffles formed the null distribution of the permutation test. The *p* value of the test was calculated as the proportion of the null distribution larger than the square of the correlation coefficient calculated using the unshuffled data. The *p* value presented the level of the dependence of the shape score on the centroid size.

Quantification of the morphological traits

Morphological traits were defined by observing the variations of the first four sPCs and were directly quantified using the 3D corolla image. The traits included tube curvature, lobe area ratio, tube dilation, and lobe recurvation (Fig. 14). Tube curvature was defined as the second-order coefficient of the quadratic equation fitted to tube axis (dotted line in Fig. 14A). Tube axis was formed as the collection of the centroid points of the landmarks on the tube–tube rims and tube midribs that have the same order from the proximal part of the corolla. The centroid points were mapped to the sagittal plane of the tube (solid line in the parallelogram in Fig. 14A) before they were used for curve fitting. Lobe area ratio was defined as the ratio of lobe area (red area in Fig. 14B) to corolla surface area. The areas were calculated as the sizes of the triangle meshes connecting the landmarks surrounding the object. Tube dilation was defined as the ratio of the centroid size of center tube transection (the 14th landmarks from the proximal part of the corolla; hollow dots in Fig. 14C) to the length of tube axis. The centroid size [43] of center tube transection was defined as the root sum squared distance between the landmarks on the tube–tube rims or tube midribs to their centroid (solid dot in Fig. 14C). Lobe recurvation was defined as the mean of lobe bending

angles of the five petals. The lobe bending angle for a petal (θ in Fig. 14D) was defined as the angle between the normal vector of the tube-opening plane (red area in Fig. 14D) and lobe-bending line (red line in Fig. 14D) of the petal. Tube-opening plane was defined as the plane optimally fitting the landmarks on the lobe–tube rim. The lobe-bending line of a petal was defined as the line connecting the proximal and distal landmarks on the lobe midrib.

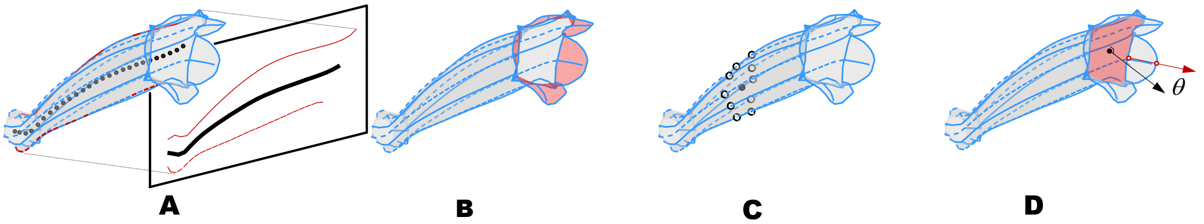

Figure 14. Illustration of the corolla shape traits: (A) tube curvature, (B) lobe area ratio, (C) tube dilation, and (D) lobe recurvation. In (A), the dotted line indicates the tube axis, the parallelogram indicates the sagittal plane of the corolla, and the solid line indicates the mapping of the tube axis to the sagittal plane. In (B), the red area indicates the lobe of the corolla. In (C), the hollow dots indicate landmarks on center tube transection, and the solid dot indicates the centroid of the landmarks. In (D), the red area indicates the tube-opening plane.

Association between the morphological traits and pollination type

The association between the morphological traits and pollination type and the level of the association were evaluated using LOD scores [44] and permutation tests, respectively. To calculate the LOD score for a trait, the ratio of the squared deviation of the trait to the sum of the within-group squared deviations of the trait was first calculated. The groups referred to pollinator types. The LOD score of the trait was then obtained as the logarithm of the ratio. A large LOD score indicates a strong association. Next, permutation tests were conducted to evaluate the levels of the association between the traits and pollination type. In a permutation test, pairs comprising a morphological trait and its pollination type were reshuffled among all the specimens 10,000 times. In each shuffle, an LOD score was calculated using the

aforementioned procedure and shuffled data. The cumulative LOD scores of the 10,000 shuffles formed the null distribution of the permutation test. The *p* value for the test was subsequently calculated as the frequency of the null distribution higher than the LOD score calculated using the unshuffled data. The *p* value presented the level of association between the morphological traits and pollination type.

Phylogenetic analysis

The phylogeny of the Corytholoma species was obtained using a maximum likelihood (ML) analysis. In the procedure, the sequences of six molecular markers of the Corytholoma species were gathered from published data ([4]; Table S4). The sequences were aligned using a program, MAFFT [45], without manual adjustment. The alignments of the markers were then concatenated to obtain 4,414 sites. Subsequently, the combination of the HKY85 model, the estimated proportion of invariant sites (+I) and the variable site following a gamma distribution (+gamma) was suggested by Modeltest 3.7 software [46], which is the best-fit nucleotide substitution model for the aligned sequence. The ML analysis was then performed using the aligned sequence, the aforementioned model and parameters suggested by Modeltest 3.7, and GARLI 2.0 software [47] for 1,000 replicates. The 50% majority-rule consensus tree of the 1,000 replicates was then used as the representative phylogeny of the Corytholoma species for the following analyses.

Tests of phylogenetic signals

The phylogenetic signals of the centroid size and morphological traits were evaluated using Blomberg’s K values [48] and permutation tests. Blomberg’s K value for a trait was calculated using the mean trait values of all the species, the phylogenetic trees of 1,000 replicates from the ML analysis, and the “phylosig” function in the R package phytools [49].

A K value of zero indicates no phylogenetic signal in the trait, whereas a K value of one indicates a strong phylogenetic signal; the trait evolution follows the Brownian motion model. The permutation tests were then performed to evaluate if the K values significantly differed from zero. In a permutation test, the pairs of the species positions on the phylogenetic tree and the mean trait value were reshuffled 10,000 times. In each shuffle, the K value was calculated using the 50% majority-rule consensus tree. The cumulative K values of the 10,000 shuffles formed the null distribution of the permutation test. The *p* value for the test was then calculated as the proportion of the null distribution larger than the K value calculated using the unshuffled data. The *p* value presented the level that the K value differed from zero.

Reconstruction of the 3D corolla shapes and forms at the ancestral states

3D corolla shapes and forms were reconstructed at the ancestral states in the phylogeny using weighted squared-change parsimony [50], the 3D landmarks of mean corolla shapes and forms of all the extant species, and the 50% majority-rule consensus tree. After reconstruction, the corolla forms were used for quantifying the centroid sizes and morphological traits at the ancestral states. To further assess the variation in the corolla shape and form at each ancestral state, the reconstruction was repeated 100 times. In each repetition, the mean corolla shape or form was calculated from three specimens that were randomly selected from the five specimens of each species. The variation of the 100 reconstructed 3D corolla shapes and forms was presented (Table S3).

Funding

This research was supported by NSC-101-2313-B-002-050-MY3 from National Science Council (Ministry of Science and Technology) of Taiwan.

Acknowledgements

We thank the National Laboratory Animal Center for the technical support on the μCT scanning and Mr. Chun-Ming Chen at the Dr. Cecilia Koo Botanic Conservation and Environmental Protection Center for providing and maintaining the plant materials.

References

1. Klingenberg CP. Size, shape, and form: concepts of allometry in geometric morphometrics. Dev Genes Evol. 2016;226(3):113-137. doi:10.1007/s00427-016-0539-2

2. Walcher-Chevillet CL, Kramer EM. Breaking the mold: understanding the evolution and development of lateral organs in diverse plant models. Curr Opin Genet Dev. 2016;39:79-84. doi:10.1016/j.gde.2016.06.005

3. Moyroud E, Glover BJ. The evolution of diverse floral morphologies. Curr Biol. 2017;27(17):R941-R951. doi:10.1016/j.cub.2017.06.053

4. Perret M, Chautems A, Spichiger R, Kite G, Savolainen V. Systematics and evolution of tribe Sinningieae (Gesneriaceae): evidence from phylogenetic analyses of six plastid DNA regions and nuclear ncpGS. Am J Bot. 2003;90(3):445-460. doi:10.3732/ajb.90.3.445

5. Lawing AM, Polly PD. Geometric morphometrics: recent applications to the study of

evolution and development. J Zool. 2010;280(1):1-7. doi:10.1111/j.1469-7998.2009.00620.x

6. Zelditch ML, Swiderski DL, Sheets HD. Geometric morphometrics for biologists: a primer. 2nd ed. Cambridge, MA: Academic Press; 2012.

7. Gower JC. Generalized procrustes analysis. Psychometrika. 1975;40(1):33-51.

8. Rohlf FJ, Slice D. Extensions of the Procrustes method for the optimal superimposition of landmarks. Syst Biol. 1990;39(1):40-59. doi:10.2307/2992207

9. Wang CN, Hsu HC, Wang CC, Lee TK, Kuo YF. Quantifying floral shape variation in 3D using microcomputed tomography: a case study of a hybrid line between actinomorphic and zygomorphic flowers. Front Plant Sci. 2015;6:724. doi:10.3389/fpls.2015.00724

10. Adams DC, Rohlf FJ, Slice DE. A field comes of age: geometric morphometrics in the 21st century. Hystrix. 2013;24(1):7. doi:10.4404/hystrix-24.1-6283

11. van der Niet T, Zollikofer CP, de León MSP, Johnson SD, Linder HP. Three-dimensional geometric morphometrics for studying floral shape variation. Trends Plant Sci. 2010;15(8):423-426. doi:10.1016/j.tplants.2010.05.005

12. Hsu HC, Wang CN, Liang CH, Wang CC, Kuo YF. Association between petal form variation and CYC2-like genotype in a hybrid line of Sinningia speciosa. Front Plant Sci. 2017;8:558. doi:10.3389/fpls.2017.00558

13. Gould SJ. Allometry and size in ontogeny and phylogeny. Biol Rev. 1966;41(4):587-638.

14. Niklas KJ. Plant allometry: the scaling of form and process. Chicago and London: University of Chicago Press; 1994.

15. Gómez JM, Torices R, Lorite J, Klingenberg CP, Perfectti F. The role of pollinators in the evolution of corolla shape variation, disparity and integration in a highly diversified plant family with a conserved floral bauplan. Ann Bot. 2016;117(5):889-904. doi:10.1093/aob/mcv194

16. Feng X, Wilson Y, Bowers J, Kennaway R, Bangham A, Hannah A, et al. Evolution of allometry in Antirrhinum. Plant Cell. 2009;21(10):2999-3007. doi:10.1105/tpc.109.069054

17. Stebbins GL. Adaptive radiation of reproductive characteristics in angiosperms, I: pollination mechanisms. Annu Rev Ecol Syst. 1970;1(1):307-326.

18. Fenster CB, Armbruster WS, Wilson P, Dudash MR, Thomson JD. Pollination syndromes and floral specialization. Annu Rev Ecol Evol Syst. 2004;35:375-403. doi:10.1146/annurev.ecolsys.34.011802.132347

19. Schulte LJ, Clark JL, Novak SJ, Jeffries SK, Smith JF. Speciation within Columnea section angustiflora (Gesneriaceae): islands, pollinators and climate. Mol Phylogenet Evol. 2015;84:125-144. doi:10.1016/j.ympev.2014.12.008

20. Ramírez-Aguirre E, Martén-Rodríguez S, Ornelas JF. Floral variation, nectar production, and reproductive success of two Drymonia (Gesneriaceae) species with mixed pollination syndromes. Int J Plant Sci. 2016;177(6):469-480. doi:10.1086/686584

21. Ling SJ, Meng QW, Tang L, Ren MX. Pollination syndromes of Chinese Gesneriaceae: a comparative study between Hainan Island and neighboring regions. Bot Rev. 2017;83(1):59-73. doi:10.1007/s12229-017-9181-6

22. Claude J. Morphometrics with R. New York: Springer Science & Business Media; 2008.

23. Klingenberg CP. Evolution and development of shape: integrating quantitative approaches. Nat Rev Genet. 2010;11(9):623. doi:10.1038/nrg2829

24. Gómez JM, Bosch J, Perfectti F, Fernández JD, Abdelaziz M, Camacho JPM. Spatial variation in selection on corolla shape in a generalist plant is promoted by the preference patterns of its local pollinators. Proc R Soc B. 2008;275(1648):2241-2249. doi:10.1098/rspb.2008.0512

25. Kaczorowski RL, Seliger AR, Gaskett AC, Wigsten SK, Raguso RA. Corolla shape vs. size in flower choice by a nocturnal hawkmoth pollinator. Funct Ecol. 2012;26(3):577-587. doi:10.1111/j.1365-2435.2012.01982.x

26. Klingenberg CP, Gidaszewski NA. Testing and quantifying phylogenetic signals and homoplasy in morphometric data. Syst Biol. 2010;59(3):245-261. doi:10.1093/sysbio/syp106

27. Chartier M, Jabbour F, Gerber S, Mitteroecker P, Sauquet H, von Balthazar M, et al. The floral morphospace–a modern comparative approach to study angiosperm evolution. New Phytol. 2014;204(4):841-853. doi:10.1111/nph.12969

28. Palci A, Lee MS. Geometric morphometrics, homology and cladistics: review and recommendations. Cladistics. 2018;1-13. doi:10.1111/cla.12340

29. Gómez JM, Perfectti F, Lorite J. The role of pollinators in floral diversification in a clade of generalist flowers. Evolution. 2015;69(4):863-878.

30. Joly S, Lambert F, Alexandre H, Clavel J, Léveillé- Bourret É, Clark JL. Greater pollination generalization is not associated with reduced constraints on corolla shape in Antillean plants. Evolution. 2018;72(2):244-260. doi:10.1111/evo.13410

31. Wiley DF, Amenta N, Alcantara DA, Ghosh D, Kil YJ, Delson E, et al. Evolutionary morphing. In VIS 05. IEEE Visualization, 2005. (pp. 431-438). IEEE.

32. Wang YH, Hsu HC, Chou WC, Kuo YF. Automatically Identifying floral contours and vascular bundles in 3D images. In 2018 ASABE Annual International Meeting, 2018. (p. 1). American Society of Agricultural and Biological Engineers.

33. Smith JF, Ooi MT, Schulte L, Amaya-Márquez M, Pritchard R, Clark JL. Searching for monophyly in the subgeneric classification systems of Columnea (Gesneriaceae). Selbyana. 2013;126-142.

34. Clark JL, Clavijo L, Muchhala N. Convergence of anti-bee pollination mechanisms in the Neotropical plant genus Drymonia (Gesneriaceae). Evol Ecol 2015;29(3):355-377.

35. Klingenberg CP. Morphological integration and developmental modularity. Annu Rev Ecol Evol Syst 2008;39:115-132. doi: 0.1146/annurev.ecolsys.37.091305.110054

36. Klingenberg CP, Marugán-Lobón J. Evolutionary covariation in geometric morphometric data: analyzing integration, modularity, and allometry in a phylogenetic context. Syst Biol 2013;62(4):591-610.

37. Gómez JM, Abdelaziz M, Muñoz- Pajares J, Perfectti F. Heritability and genetic correlation of corolla shape and size in Erysimum mediohispanicum. Evolution. 2009;63(7):1820-1831. doi:10.1111/j.1558-5646.2009.00667.x

38. Rangel TF, Colwell RK, Graves GR, Fučíková K, Rahbek C, Diniz- Filho JAF. Phylogenetic uncertainty revisited: Implications for ecological analyses. Evolution. 2015;69(5):1301-1312. doi:10.1111/evo.12644

39. Chautems A, Lopes TCC, Peixoto M, Rossini J. Taxonomic revision of Sinningia Nees (Gesneriaceae) IV: six new species from Brazil and a long overlooked taxon. Candollea. 2010;65(2):241-267.

40. Rohlf FJ, Slice D. Extensions of the Procrustes method for the optimal superimposition of landmarks. Syst Biol. 1990;39(1):40-59.

41. Monteiro LR. Multivariate regression models and geometric morphometrics: the search for causal factors in the analysis of shape. Syst Biol. 1999;48(1):192-199.

42. Churchill GA, Doerge RW. Empirical threshold values for quantitative trait mapping. Genetics. 1994;138(3):963-971.

43. Mitteroecker P, Gunz P. Advances in geometric morphometrics. Evol Biol.

2009;36(2):235-247.

44. Morton NE. Sequential tests for the detection of linkage. Am J Hum Genet. 1955;7(3):277.

45. Katoh K, Standley DM. MAFFT multiple sequence alignment software version 7: improvements in performance and usability. Mol biol evol. 2013;30(4):772-780. doi:10.1093/molbev/mst010

46. Posada D, Crandall KA. Modeltest: testing the model of DNA substitution. Bioinformatics. 1998;14:817-818.

47. Bazinet AL, Zwickl DJ, Cummings MP. A gateway for phylogenetic analysis powered by grid computing featuring GARLI 2.0. Syst biol. 2014;63(5):812-818. doi:10.1093/sysbio/syu031

48. Blomberg SP, Garland T, Ives AR. Testing for phylogenetic signal in comparative data: behavioral traits are more labile. Evolution. 2003;57(4):717-745. doi:10.1111/j.0014-3820.2003.tb00285.x

49. Revell LJ. Phytools: an R package for phylogenetic comparative biology (and other things). Methods Ecol Evol. 2012;3(2):217-223.

50. Maddison WP. Squared-change parsimony reconstructions of ancestral states for continuous-valued characters on a phylogenetic tree. Syst Biol. 1991;40(3):304-314.

Supplement

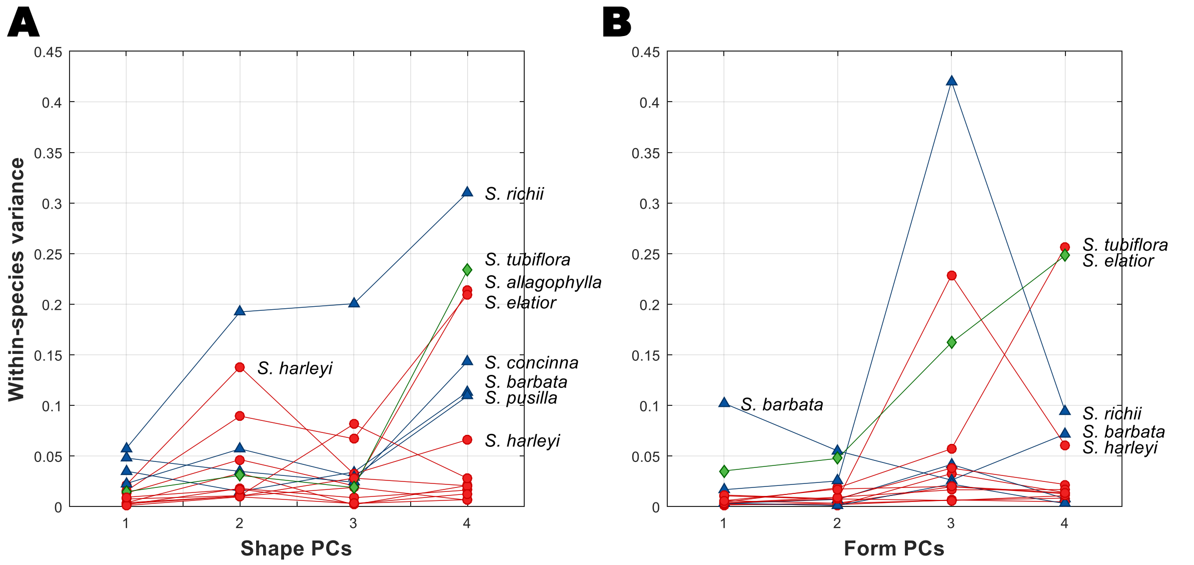

Figure S1. Within-species variance of the extant species in (A) sPCs and (B) fPCs. Red circle, blue triangle, and green diamond represent hummingbird-pollinated, bee-pollinated, and moth-pollinated species, respectively.

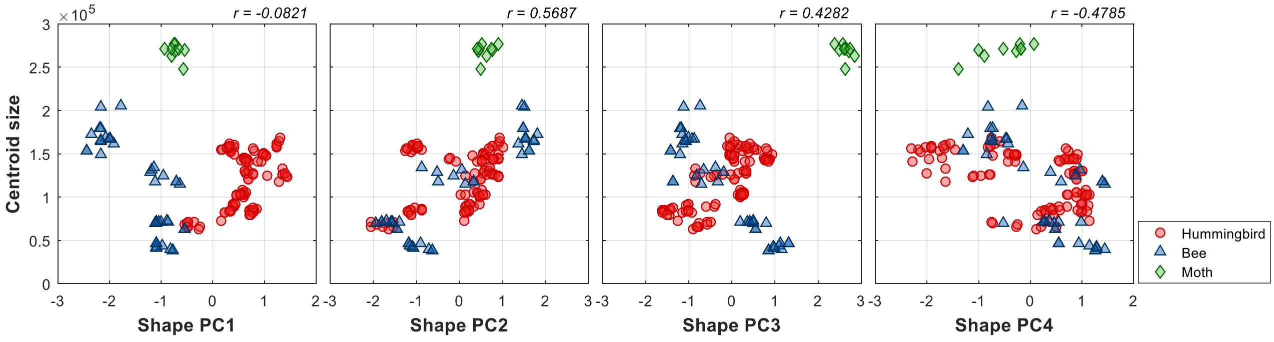

Figure S2. Scatter plot of centroid size versus each sPC.

Table S1. List of studies using landmark-based GM.

Approach	Imaging object	Keywords (selected)	Family	Landmark*		GM		References
				Primary	Secondary	GPA	PCA/LDA	
2D	Side-view	Petal shape; Floral morphology	Gesneriaceae	5	10	Full	PCA	[1]
		Pollination syndrome; Phylogenetic comparative methods	Gesneriaceae	6	26	Full	PCA	[2, 3]
		Pollinator selection; Flower shape	Loasaceae	5	0	Full	PCA, LDA	[4, 5]
		Flower shape; Pollination niches	Plantaginaceae	4	12	Full	PCA	[6]
		Trait-dependent diversification; Pollination; Flower tube	Plantaginaceae	9	1	Full	LDA	[7]
		Ancillary traits	Rubiaceae	9	8	Full	PCA, LDA	[8]
	Face-view	Selfing syndrome	Brassicaceae	30	0	Full	PCA	[9]
		Floral shape evolution; pollination; corolla shape; plant-pollinator interactions; floral morphospace; allometry	Brassicaceae	32	0	Full	PCA	[10-15]
		Petal shape; Floral morphology	Gesneriaceae	5	25	Full	PCA	[1]
		Floral shape**	Goodeniaceae	5	0	Full	PCA	[16]
		Fluctuating asymmetry	Orchidaceae	4	5	Full	PCA	[17]
		Flower shape	Plantaginaceae	28	0	Full	PCA	[18]
		Corolla shape; pollination	Solanaceae	5	35	Full	PCA	[19}
		Floral symmetry	Valerianaceae	10	0	Full	LDA	[20]
	Dissected-view	Actinomorphy; Zygomorphy	Leguminosae	2	18	Full	PCA	[21]
		Floral shape**	Leguminosae	37	0	Full	PCA	[22]
		Directional asymmetry; floral organ shape; fluctuating asymmetry	Iridaceae	39	16	Full	PCA	[23, 24]
		Floral shape**	Orchidaceae	15	0	Full	PCA	[25]
		Floral shape, allometry**	Plantaginaceae	4	16	Partial	PCA	[26]
		Floral shape**	Plantaginaceae	8	47	Partial	PCA	[27]
3D	Whole corolla	Petal shape; dorsoventral asymmetry	Gesneriaceae	10	65	Full	PCA	[28]
		Dorsoventral asymmetry; petal form variation	Gesneriaceae	25	100	Full	PCA	[29]
		Floral shape, pollination**	Orchidaceae	26	14	Full	PCA	[30]
		Corolla shape variations	Gesneriaceae	25	390	Full/ Partial	PCA	Present study

* The primary and secondary landmarks follow the definitions in the Methods section.
** Keywords were not provided in these studies. The keywords were assigned by authors of the present study.

Table S2. The specimen information and the scan parameters

Species	Isolate	Specimen ID	Scan parameters			Specimen use*
			Voltage (kV)	Current (μA)	Resolution (μm)	
Sinningia aggregata	K039091	K039091_07	49	200	36.54704	G, E, P, A
		K039091_08	49	200	36.54704	G, E
		K039091_09	49	200	36.54704	G, E, P, A
	K039092	K039092_01	40	250	36.54703	G, E
		K039092_02	40	250	36.54703	G, E
		K039092_03	40	250	36.54703	G, E
		K039092_04	49	200	36.54704	G, E
		K039092_05	49	200	36.54704	G, E, P, A
	K039093	K039093_01	49	200	36.54704	G, E
		K039093_02	49	200	36.54704	G, E, P, A
		K039093_03	49	200	36.54704	G, E, P, A
Sinningia allagophylla	HC0909-d	HC0909-d_09	40	250	36.54703	G, E, P, A
	K039099	K039099_01	40	250	36.54703	G, E
		K039099_02	40	250	36.54703	G, E
		K039099_03	40	250	36.54703	G, E, P, A
		K039099_04	40	250	36.54703	G, E, P, A
		K039099_05	40	250	36.54703	G, E
	K039100	K039100_07	40	250	36.54704	G, E, P, A
		K039100_08	40	250	36.54704	G, E, P, A
Sinningia barbata	HC1206-a	HC1206-a_02	49	200	36.54703	G, E, P, A
		HC1206-a_03	49	200	36.54703	G, E, P, A
		HC1206-a_09	49	200	36.54703	G, E
	HC1206-d	HC1206-d_06	40	250	36.54704	G, E
		HC1206-d_07	40	250	36.54704	G, E
		HC1206-d_08	40	250	36.54704	G, E
		HC1206-d_09	40	250	36.54703	G, E, P, A
		HC1206-d_10	40	250	36.54703	G, E, P, A
	K039104	K039104_02	49	200	36.54703	G, E
	K039105	K039105_01	49	200	36.54703	G, E
		K039105_02	40	250	36.54704	G, E
		K039105_03	40	250	36.54704	G, E, P, A
		K039105_04	40	250	36.54704	G, E
		K039105_05	40	250	36.54704	G, E
Sinningia carangolensis	HC1912-2	HC1912-2_02	40	250	36.54703	G, E, P, A
		HC1912-2_03	40	250	36.54703	G, E, P, A
		HC1912-2_04	40	250	36.54703	G, E, P, A
	HC1912-b	HC1912-b_01	40	250	36.54703	G, E
		HC1912-b_08	40	250	36.54704	G, E
	K039112	K039112_01	40	250	36.54704	G, E, P, A
		K039112_02	40	250	36.54704	G, E
		K039112_03	40	250	36.54704	G, E, P, A
		K039112_04	40	250	36.54704	G, E
		K039112_05	40	250	36.54704	G, E
Sinningia concinna	HC2202-t	HC2202-t_04	40	250	36.54703	G, E, P, A
		HC2202-t_05	40	250	36.54703	G, E

	K039117	K039117_01	40	250	36.54703	G, E
		K039117_03	40	250	36.54703	G, E
		K039117_06	40	250	36.54703	G, E, P, A
	K039118	K039118_01	40	250	36.54703	G, E
		K039118_02	40	250	36.54703	G, E
		K039118_03	40	250	36.54703	G, E, P, A
		K039118_04	40	250	36.54703	G, E, P, A
		K039118_05	40	250	36.54703	G, E, P, A
Sinningia elatior	K039126	K039126_01	49	200	36.54703	G, E
		K039126_02	49	200	36.54703	G, E
	K039127	K039127_01	49	200	36.54703	G, E, P, A
		K039127_02	49	200	36.54703	G, E, P, A
	K039129	K039129_01	49	200	36.54703	G, E, P, A
		K039129_02	49	200	36.54703	G, E
		K039129_03	49	200	36.54703	G, E, P, A
	K039131	K039131_01	49	200	36.54703	G, E, P, A
Sinningia harleyi	HC3403-3	HC3403-3_10	49	200	36.54703	G, E, P, A
		HC3403-3_16	49	200	36.54703	G, E
		HC3403-3_17	49	200	36.54703	G, E
	HC3403-8	HC3403-8_01	40	250	36.54703	G, E
		HC3403-8_08	40	250	36.54703	G, E
		HC3403-8_09	40	250	36.54703	G, E
		HC3403-8_10	40	250	36.54703	G, E, P, A
		HC3403-8_11	40	250	36.54703	G, E
		HC3403-8_12	40	250	36.54703	G, E, P, A
		HC3403-8_13	40	250	36.54703	G, E
		HC3403-8_14	40	250	36.54703	G, E
	K039135	K039135_01	40	250	36.54703	G, E
		K039135_02	40	250	36.54703	G, E, P, A
		K039135_03	40	250	36.54703	G, E
		K039135_04	40	250	36.54703	G, E
		K039135_05	40	250	36.54703	G, E, P, A
Sinningia nordestina	HC5504-1	HC5504-1_02	40	250	36.54703	G, E
		HC5504-1_03	40	250	36.54703	G, E, P, A
		HC5504-1_04	40	250	36.54703	G, E
	HC5504-3	HC5504-3_03	40	250	36.54704	G, E, P, A
		HC5504-3_04	40	250	36.54704	G, E
	K039168	K039168_01	40	250	36.54703	G, E
		K039168_02	40	250	36.54703	G, E, P, A
		K039168_03	40	250	36.54703	G, E
		K039168_04	40	250	36.54703	G, E, P, A
		K039168_05	40	250	36.54703	G, E, P, A
Sinningia pusilla	HC5803-2	HC5803-2_01	40	250	36.54703	G, E
	HC5803-7	HC5803-7_09	40	250	36.54703	G, E, P, A
	K039170	K039170_01	40	250	36.54703	G, E, P, A
		K039170_02	40	250	36.54703	G, E
		K039170_03	40	250	36.54703	G, E
		K039170_04	40	250	36.54703	G, E
		K039170_05	40	250	36.54703	G, E, P, A
	K039171	K039171_01	40	250	36.54703	G, E, P, A
	K039172	K039172_01	40	250	36.54703	G, E, P, A
Sinningia richii	K039174	K039174_01	49	200	36.54703	G, E
		K039174_02	40	250	36.54703	G, E, P, A
		K039174_03	40	250	36.54704	G, E
		K039174_04	40	250	36.54704	G, E, P, A
		K039174_05	40	250	36.54704	G, E, P, A
	K039175	K039175_01	40	250	36.54704	G, E, P, A

	K039176	K039176_01	40	250	36.54704	G, E
	K039177	K039177_01	40	250	36.54704	G, E, P, A
Sinningia sceptrum	K039178	K039178_01	49	200	36.54703	G, E
		K039178_02	49	200	36.54703	G, E, P, A
		K039178_03	49	200	36.54703	G, E
		K039178_04	49	200	36.54703	G, E, P, A
		K039178_05	49	200	36.54703	G, E, P, A
	K039179	K039179_01	49	200	36.54703	G, E, P, A
		K039179_06	49	200	36.54703	G, E, P, A
	K039181	K039181_01	49	200	36.54703	G, E
		K039181_02	49	200	36.54703	G, E
Sinningia sellovii	K039184	K039184_01	49	200	36.54703	G, E
		K039184_02	49	200	36.54703	G, E
		K039184_03	49	200	36.54703	G, E, P, A
		K039184_04	49	200	36.54703	G, E
		K039184_05	49	200	36.54703	G, E
	K039185	K039185_02	49	200	36.54703	G, E
	K039186	K039186_11	49	200	36.54703	G, E
		K039186_12	49	200	36.54703	G, E, P, A
		K039186_13	49	200	36.54703	G, E, P, A
		K039186_14	49	200	36.54703	G, E
		K039186_15	49	200	36.54703	G, E, P, A
		K039186_16	49	200	36.54703	G, E, P, A
Sinningia tubiflora	K039197	K039197_01	49	200	36.54703	G, E
		K039197_02	49	200	36.54703	G, E, P, A
	K039198	K039198_01	40	250	36.54704	G, E, P, A
		K039198_02	40	250	36.54704	G, E, P, A
	K039199	K039199_02	40	250	36.54704	G, E
	K039200	K039200_01	49	200	36.54703	G, E, P, A
		K039200_02	49	200	36.54703	G, E
		K039200_03	49	200	36.54703	G, E, P, A
	K039201	K039201_04	40	250	36.54704	G, E
Sinningia valsuganensis	K039203	K039203_01	40	250	36.54704	G, E, P, A
		K039203_02	40	250	36.54704	G, E, P, A
		K039203_03	40	250	36.54704	G, E, P, A
		K039203_04	40	250	36.54704	G, E, P, A
		K039203_05	40	250	36.54704	G, E, P, A
	K039204	K039204_06	40	250	36.54704	G, E
Sinningia warmingii	K039205	K039205_01	49	200	36.54703	G, E, P, A
		K039205_02	49	200	36.54703	G, E
		K039205_03	49	200	36.54703	G, E, P, A
		K039205_04	49	200	36.54704	G, E
		K039205_05	40	250	36.54704	G, E
	K039209	K039209_03	40	250	36.54704	G, E, P, A
		K039209_09	40	250	36.54704	G, E, P, A
		K039209_10	40	250	36.54704	G, E
		K039209_11	40	250	36.54704	G, E
	K039216	K039216_01	49	200	36.54704	G, E
		K039216_02	49	200	36.54704	G, E, P, A
		K039216_04	49	200	36.54704	G, E
		K039216_05	49	200	36.54704	G, E

* The letter G denotes the analysis of 3D shape and form variation, the letter E denotes the analysis of evolutionary allometry, the letter P denotes the analysis of phylogenetic signal, and the letter A denotes the analysis of ancestral state reconstruction.

Table S3. Centroid size and four morphological traits of extant species and ancestral states.

	Centroid size (10^4)	Traits (mean ± standard deviation)			
		Tube curvature (10^{-4})	Lobe area ratio (10^{-2})	Tube dilation (10^{-1})	Lobe recurvation (10^2)
**Extant species**					
S. aggregata	10.34 ± 0.29	−1.77 ± 0.99	7.59 ± 0.56	8.99 ± 0.32	1.70 ± 0.29
S. allagophylla	6.71 ± 0.35	−0.42 ± 0.82	14.93 ± 2.56	17.12 ± 0.73	2.93 ± 0.41
S. barbata	16.73 ± 1.01	14.26 ± 1.73	3.97 ± 0.24	16.01 ± 0.31	5.80 ± 0.25
S. carangolensis	12.71 ± 0.39	−1.48 ± 0.21	7.59 ± 0.32	8.87 ± 0.40	1.61 ± 0.32
S. concinna	6.93 ± 0.35	8.51 ± 2.60	14.90 ± 0.86	9.79 ± 0.21	3.06 ± 0.22
S. elatior	12.54 ± 0.50	−3.88 ± 1.51	6.33 ± 0.92	10.66 ± 1.20	4.26 ± 0.54
S. harleyi	15.75 ± 0.20	−1.99 ± 0.50	8.87 ± 0.20	11.39 ± 0.28	6.11 ± 0.45
S. nordestina	8.52 ± 0.10	−9.57 ± 0.28	12.89 ± 0.42	12.76 ± 0.53	1.90 ± 0.08
S. pusilla	4.26 ± 0.30	7.10 ± 2.29	13.56 ± 1.30	10.08 ± 0.67	3.41 ± 0.23
S. richii	12.25 ± 0.75	6.30 ± 0.84	10.61 ± 0.81	18.61 ± 1.05	3.37 ± 0.77
S. sceptrum	14.91 ± 0.13	−1.37 ± 0.22	6.87 ± 0.05	8.76 ± 0.06	3.26 ± 0.21
S. sellovii	9.04 ± 0.14	0.70 ± 0.96	8.10 ± 0.76	16.71 ± 1.55	1.80 ± 0.29
S. tubiflora	26.70 ± 1.19	2.43 ± 0.27	9.42 ± 0.66	6.06 ± 0.14	5.00 ± 1.01
S. valsuganensis	16.03 ± 0.25	−2.20 ± 0.25	6.14 ± 0.42	7.40 ± 0.20	4.20 ± 0.10
S. warmingii	14.53 ± 0.24	0.14 ± 0.45	5.17 ± 0.27	5.88 ± 0.07	1.80 ± 0.19
**Ancestral states**					
Node 1	11.94 ± 0.11	6.18 ± 0.24	7.86 ± 0.18	15.45 ± 0.15	3.17 ± 0.21
Node 2	11.91 ± 0.10	6.15 ± 0.26	7.60 ± 0.15	15.08 ± 0.12	3.16 ± 0.17
Node 3	11.93 ± 0.10	5.64 ± 0.29	7.36 ± 0.13	15.05 ± 0.11	3.10 ± 0.15
Node 4	11.47 ± 0.08	5.00 ± 0.23	7.51 ± 0.10	14.25 ± 0.10	2.72 ± 0.15
Node 5	11.36 ± 0.06	2.97 ± 0.18	7.71 ± 0.08	13.32 ± 0.09	2.33 ± 0.12
Node 6	11.80 ± 0.05	1.22 ± 0.13	7.28 ± 0.07	11.97 ± 0.08	2.15 ± 0.09
Node 7	12.11 ± 0.05	1.15 ± 0.13	7.09 ± 0.08	11.75 ± 0.08	2.02 ± 0.05
Node 8	10.80 ± 0.03	1.06 ± 0.11	7.21 ± 0.13	11.52 ± 0.22	1.78 ± 0.12
Node 9	11.38 ± 0.05	1.39 ± 0.11	6.91 ± 0.12	9.88 ± 0.11	2.10 ± 0.11
Node 10	12.15 ± 0.06	1.46 ± 0.12	6.38 ± .013	9.03 ± 0.08	2.12 ± 0.13
Node 11	15.34 ± 0.11	1.96 ± 0.11	4.96 ± 0.14	7.16 ± 0.04	2.59 ± 0.23
Node 12	11.69 ± 0.06	2.49 ± 0.19	8.10 ± 0.16	13.16 ± 0.08	3.90 ± 0.10

Node 13	13.07 ± 0.07	−1.71 ± 0.26	6.50 ± 0.14	10.18 ± 0.19	3.21 ± 0.10
Node 14	13.23 ± 0.08	−1.29 ± 0.06	6.80 ± 0.12	8.98 ± 0.09	2.39 ± 0.07

Table S4. Species list and GenBank numbers.

**Species**	***trnS-trnG***	***ncpGS***	***rpl16***	***atpB-rbcL***	***trnL-trnF***	***trnT-trnL***
S. aggregata	AJ438364	AJ459619	AJ487715	AJ439913	AJ439757	AJ439262
S. allagophylla	AJ438407	AJ459663	AJ487758	AJ439956	AJ439801	AJ439306
S. barbata	AJ438386	AJ459642	AJ487738	AJ439936	AJ439780	AJ439285
S. carangolensis	AJ438391	AJ459647	AJ487743	AJ439940	AJ439785	AJ439290
S. concinna	AJ438393	AJ459649	AJ487745	AJ439942	AJ439787	AJ439292
S. elatior	AJ438398	AJ459654	AJ487749	AJ439947	AJ439792	AJ439297
S. harleyi	AJ438392	AJ459648	AJ487744	AJ439941	AJ439786	AJ439291
S. nordestina	AJ438387	AJ459643	AJ487739	AJ439937	AJ439781	AJ439286
S. pusilla	AJ438410	AJ459666	AJ487761	AJ439959	AJ439804	AJ439309
S. richii	AJ438403	AJ459659	AJ487754	AJ439952	AJ439797	AJ439302
S. sceptrum	AJ438399	AJ459655	AJ487750	AJ439948	AJ439793	AJ439298
S. sellovii	AJ438383	AJ459639	AJ487735	AJ439933	AJ439777	AJ439282
S. tubiflora	AJ438380	AJ459636	AJ487732	AJ439930	AJ439774	AJ439279
S. valsuganensis	AJ438401	AJ459657	AJ487752	AJ439950	AJ439795	AJ439300
S. warmingii	AJ438372	AJ459627	AJ487723	AJ439921	AJ439765	AJ439270

Reference (Table S1)

1. Hsu HC, Chen CY, Lee TK, Weng LK, Yeh DM, Lin TT., et al. Quantitative analysis of floral symmetry and tube dilation in an F2 cross of Sinningia speciosa. Sci Hort. 2015;188:71-77. doi:10.1016/j.scienta.2015.03.0

2. Alexandre H, Vrignaud J, Mangin B, Joly S. Genetic architecture of pollination syndrome transition between hummingbird-specialist and generalist species in the genus Rhytidophyllum (Gesneriaceae). PeerJ; 2015;3:e1028. doi:10.7717/peerj.1028

3. Joly S, Lambert F, Alexandre H, Clavel J, Léveillé- Bourret É, Clark JL. Greater pollination generalization is not associated with reduced constraints on corolla shape in Antillean plants. Evolution. 2018;72(2):244-260. doi:10.1111/evo.13410

4. Strelin MM, Benitez-Vieyra S, Ackermann M, Cocucci AA. Flower reshaping in the transition to hummingbird pollination in Loasaceae subfam. Loasoideae despite absence of corolla tubes or spurs. Evol Ecol. 2016;30(3):401-417.

5. Strelin MM, Benitez- Vieyra S, Fornoni J, Klingenberg CP, Cocucci A. The evolution of floral ontogenetic allometry in the Andean genus Caiophora (Loasaceae, subfam. Loasoideae). Evol Dev. 2018;20(1):29-39. doi:10.1111/ede.12246

6. Blanco- Pastor JL, Ornosa C, Romero D, Liberal IM, Gómez JM, Vargas P. Bees explain

floral variation in a recent radiation of Linaria. J Evolution Biol. 2015;28(4):851-863. doi:10.1111/jeb.12609

7. Fernández-Mazuecos M, Blanco-Pastor JL, Gómez JM, Vargas P. Corolla morphology influences diversification rates in bifid toadflaxes (Linaria sect. Versicolores). Ann Bot. 2013;112(9):1705-1722. doi:10.1093/aob/mct214

8. Hernández-Ramírez AM, Aké-Castillo JA. A geometric morphometrics study of stigma-anther polymorphism in the tropical distylous Palicourea padifolia (Rubiaceae). Am J Plant Sci. 2014;5(10):1449. doi:10.4236/ajps.2014.510160

9. Carleial S, Van Kleunen M, Stift M. Small reductions in corolla size and pollen: ovule ratio, but no changes in flower shape in selfing populations of the North American Arabidopsis lyrata. Oecologia. 2017;183(2):401-413. doi: 10.1007/s00442-016-3773-4

10. Gómez JM, Perfectti F, Camacho JPM. Natural selection on Erysimum mediohispanicum flower shape: insights into the evolution of zygomorphy. Am Nat. 2006;168(4):531-545. doi:10.1086/507048

11. Gómez JM, Bosch J, Perfectti F, Fernández JD, Abdelaziz M, Camacho JPM. Spatial variation in selection on corolla shape in a generalist plant is promoted by the preference patterns of its local pollinators. Proc R Soc B. 2008;275(1648):2241-2249. doi:10.1098/rspb.2008.0512

12. Gómez JM, Abdelaziz M, Muñoz- Pajares J, Perfectti F. Heritability and genetic

correlation of corolla shape and size in Erysimum mediohispanicum. Evolution. 2009;63(7):1820-1831. doi:10.1111/j.1558-5646.2009.00667.x

13. Savriama Y, Gómez JM, Perfectti F, Klingenberg CP. Geometric morphometrics of corolla shape: dissecting components of symmetric and asymmetric variation in Erysimum mediohispanicum (Brassicaceae). New Phytol. 2012;196(3):945-954. doi:10.1111/j.1469-8137.2012.04312.x

14. Gómez JM, Perfectti F, Lorite J. The role of pollinators in floral diversification in a clade of generalist flowers. Evolution. 2015;69(4):863-878.

15. Gómez JM, Torices R, Lorite J, Klingenberg CP, Perfectti F. The role of pollinators in the evolution of corolla shape variation, disparity and integration in a highly diversified plant family with a conserved floral bauplan. Ann Bot. 2016;117(5):889-904. doi:10.1093/aob/mcv194

16. Gardner AG, Gerald JNF, Menz J, Shepherd KA, Howarth DG, Jabaily RS. Characterizing floral symmetry in the Core Goodeniaceae with geometric morphometrics. PLoS One. 2016;11(5):e0154736. doi:10.1371/journal.pone.0154736

17. Shipunov AB, Bateman RM. Geometric morphometrics as a tool for understanding Dactylorhiza (Orchidaceae) diversity in European Russia. Biol J Linn Soc. 2005;85(1):1-12. doi:10.1111/j.1095-8312.2005.00468.x

18. Baranzelli MC, Johnson LA, Cosacov A, Sérsic AN. Historical and ecological divergence

among populations of Monttea chilensis (Plantaginaceae), an endemic endangered shrub bordering the Atacama Desert, Chile. Evol Ecol. 2014;28(4):751-774. doi:10.1007/s10682-014-9694-y

19. Kaczorowski RL, Seliger AR, Gaskett AC, Wigsten SK, Raguso RA. Corolla shape vs. size in flower choice by a nocturnal hawkmoth pollinator. Funct Ecol. 2012;26(3):577-587. doi:10.1111/j.1365-2435.2012.01982.x

20. Berger BA, Ricigliano VA, Savriama Y, Lim A, Thompson V, Howarth DG. Geometric morphometrics reveals shifts in flower shape symmetry and size following gene knockdown of CYCLOIDEA and ANTHOCYANIDIN SYNTHASE. BMC plant biol. 2017;17(1):205. doi:10.1186/s12870-017-1152-x

21. Sinjushin AA, Bagheri A, Maassoumi AA, Rahiminejad MR. Terata of two legume species with radialized corolla: some correlations in floral symmetry. Plant Syst. Evol. 2015;301(10):2387-2397. doi:10.1007/s00606-015-1235-9

22. Püschel TA, Espejo J, Sanzana MJ, Benítez HA. Analysing the floral elements of the lost tree of Easter Island: a morphometric comparison between the remaining ex-situ lines of the endemic extinct species Sophora toromiro. PloS One. 2014;9(12):e115548. doi:10.1371/journal.pone.0115548

23. Radović S, Urošević A, Hočevar K, Vuleta A, Manitašević Jovanović S, Tucić B. Geometric morphometrics of functionally distinct floral organs in Iris pumila: Analyzing

patterns of symmetric and asymmetric shape variations. Arch Biol Sci. 2017;69(2):223-231. doi:10.2298/ABS160912086R

24. Tucić B, Budečević S, Manitašević Jovanović S, Vuleta A, Klingenberg CP. Phenotypic plasticity in response to environmental heterogeneity contributes to fluctuating asymmetry in plants: first empirical evidence. J Evolution Biol. 2018;31(2):197-210. doi:10.1111/jeb.13207

25. Dalayap RM, Torres MAJ, Demayo CG. Landmark and outline methods in describing petal, sepal and labellum shapes of the flower of Mokara orchid varieties. Int J Agric Biol. 2011;13:652-658. doi:11-106/AWB/2011/13-5-652-658

26. Feng X, Wilson Y, Bowers J, Kennaway R, Bangham A, Hannah A, et al. Evolution of allometry in Antirrhinum. Plant Cell. 2009;21(10):2999-3007. doi:10.1105/tpc.109.069054

27. Cui ML, Copsey L, Green AA, Bangham JA, Coen E. Quantitative control of organ shape by combinatorial gene activity. PLoS Biol. 2010;8(11):e1000538. doi:10.1371/journal.pbio.1000538

28. Wang CN, Hsu HC, Wang CC, Lee TK, Kuo YF. Quantifying floral shape variation in 3D using microcomputed tomography: a case study of a hybrid line between actinomorphic and zygomorphic flowers. Front Plant Sci. 2015;6:724. doi:10.3389/fpls.2015.00724

29. Hsu HC, Wang CN, Liang CH, Wang CC, Kuo YF. Association between petal form variation and CYC2-like genotype in a hybrid line of Sinningia speciosa. Front Plant Sci.

2017;8:558. doi:10.3389/fpls.2017.00558

30. van der Niet T, Zollikofer CP, de León MSP, Johnson SD, Linder HP. Three-dimensional geometric morphometrics for studying floral shape variation. Trends Plant Sci. 2010;15(8):423-426. doi:10.1016/j.tplants.2010.05.005

 Click here to access/download;Table;Table 1_5.9.docx

Table 1. Species list and dimension of the slice images.

Species	Pollination type [a]	Specimen type [b]	KBCC and inbred line accession
S. aggregata	H	F	K039091, K039092, K039093
S. allagophylla	H	F	K039099, L039110, HC0909-d
S. barbata	B	E/F	K039104, K039105, HC1206-a, HC1206-d
S. carangolensis	H	F	K039112, HC1912-2, HC1912-b
S. concinna	B	F	K039117, K039118, HC2202-t
S. elatior	H	E	K039126, K039127, K039129, K039131
S. harleyi	H	F	K039135, HC3403-3, HC3403-8
S. nordestina	H	F	K039168, HC5504-1, HC5504-3
S. pusilla	B	F	K039169, K039170, K039171, K039172, HC5803-2
S. richii	B	E/F	K039174, K039175, K039176, K039177
S. sceptrum	H	E	K039178, K039179, K039181
S. sellovii	H	E	K039184, K039185, K039186
S. tubiflora	M	F	K039197, K039198, K039199, K039200, K039201
S. valsuganensis	H	F	K039203, K039204
S. warmingii	H	E	K039205, K039209, K039216

[a] H: hummingbird pollination (ornithophily), B: bee pollination (melittophily), and M: moth pollination (phalaenophily).
[b] The letter E denotes the 70% ethanol-fixed specimen, and the letter F denotes the fresh specimen.
[c] The 3D images with a slice size of 1968 × 1968 were downsized to 984 × 984 before the reconstruction of volumetric and surface images. The identified landmarks were then magnified back to the original scale for the subsequent GM analysis.

Click here to access/download;Table;Table 2_5.9.docx

Table 2. Kruskal–Wallis test results, Scheffé's multiple comparison test results, and LOD scores of the morphological traits by pollination type.

Morphological traits	Kruskal-Wallis test		Scheffé's multiple comparison test						LOD score	*p*-value
			Hummingbird vs Bee		Hummingbird vs Moth		Bee vs Moth			
	H-value	*p*-value	*T*-value	*p*-value	*T*-value	*p*-value	*T*-value	*p*-value		
**Centroid size**	26.81	$1.51{\times}10^{-6}$	1.76	$2.15{\times}10^{-1}$	11.23	$1.11{\times}10^{-16}$	11.49	$1.11{\times}10^{-16}$	21.71	0
**Tube curvature**	102.56	$5.36{\times}10^{-23}$	20.96	$1.11{\times}10^{-16}$	4.13	$3.09{\times}10^{-4}$	6.62	$4.59{\times}10^{-9}$	45.52	0
**Lobe area ratio**	10.83	$4.50{\times}10^{-3}$	3.71	$1.38{\times}10^{-3}$	0.55	$8.60{\times}10^{-1}$	1.34	$4.08{\times}10^{-1}$	2.92	$1.60{\times}10^{-3}$
**Tube dilation**	31.82	$1.23{\times}10^{-7}$	4.10	$3.42{\times}10^{-4}$	3.81	$9.83{\times}10^{-4}$	5.66	$5.06{\times}10^{-7}$	7.31	0
**Lobe recurvation**	23.42	$8.20{\times}10^{-6}$	3.60	$2.02{\times}10^{-3}$	2.92	$1.57{\times}10^{-2}$	0.95	$6.34{\times}10^{-1}$	3.91	$1.80{\times}10^{-4}$

Click here to access/download;Figure;Fig.1_5.9.pdf

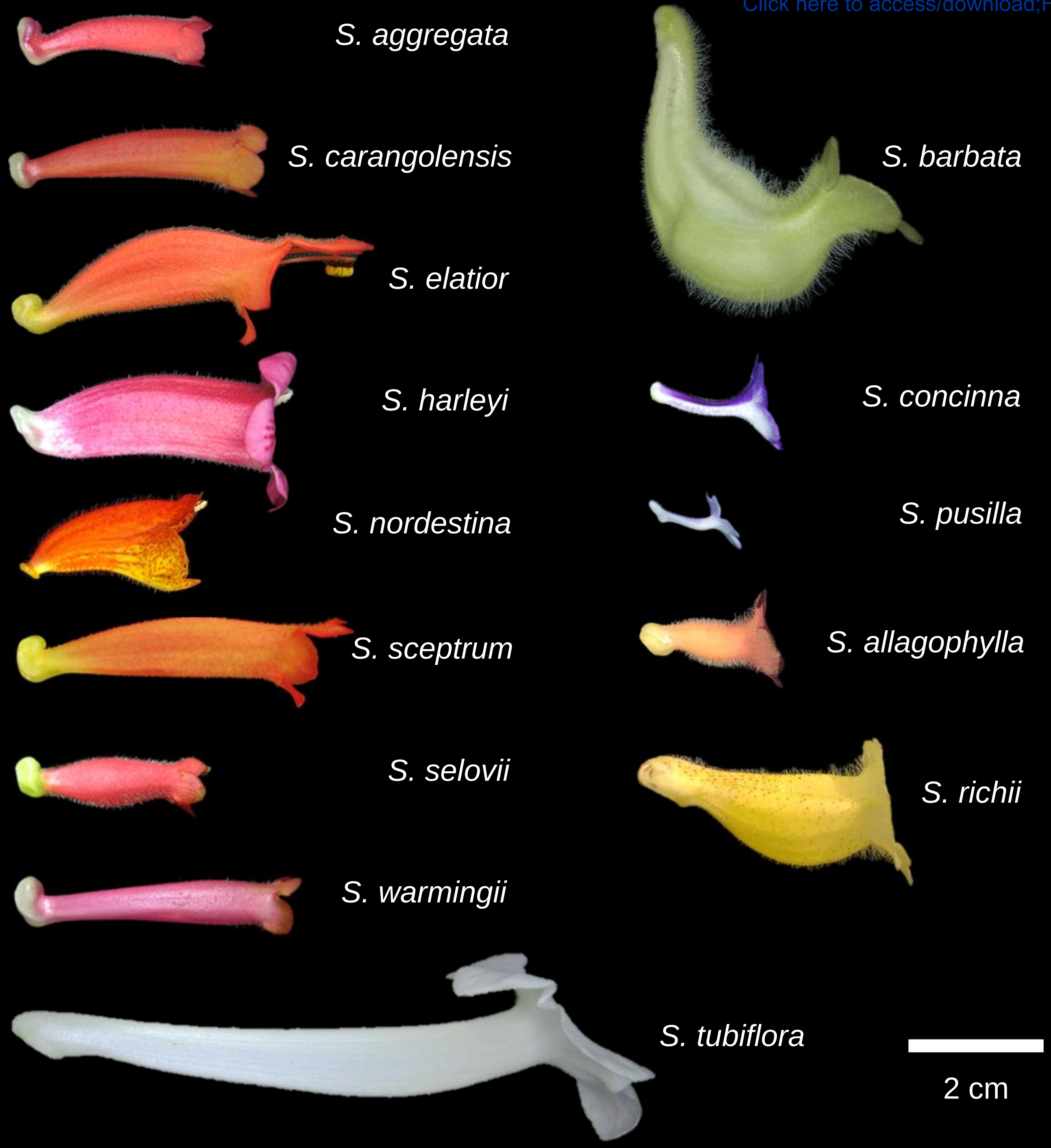

Figure 2

Click here to access/download;Figure;Fig.2_5.9.pdf

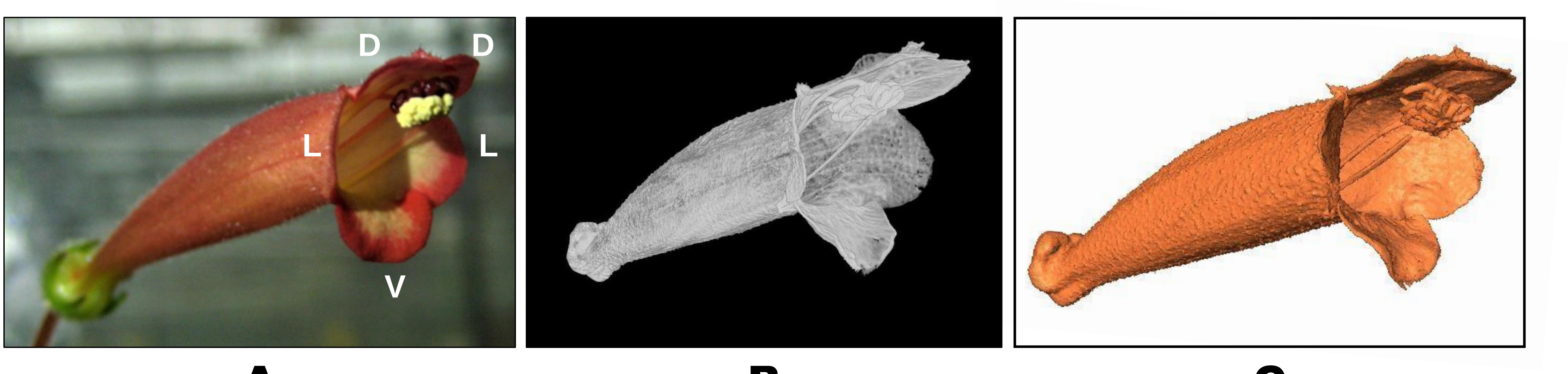

A B C

Figure 3

Click here to access/download;Figure;Fig.3_5.9.pdf

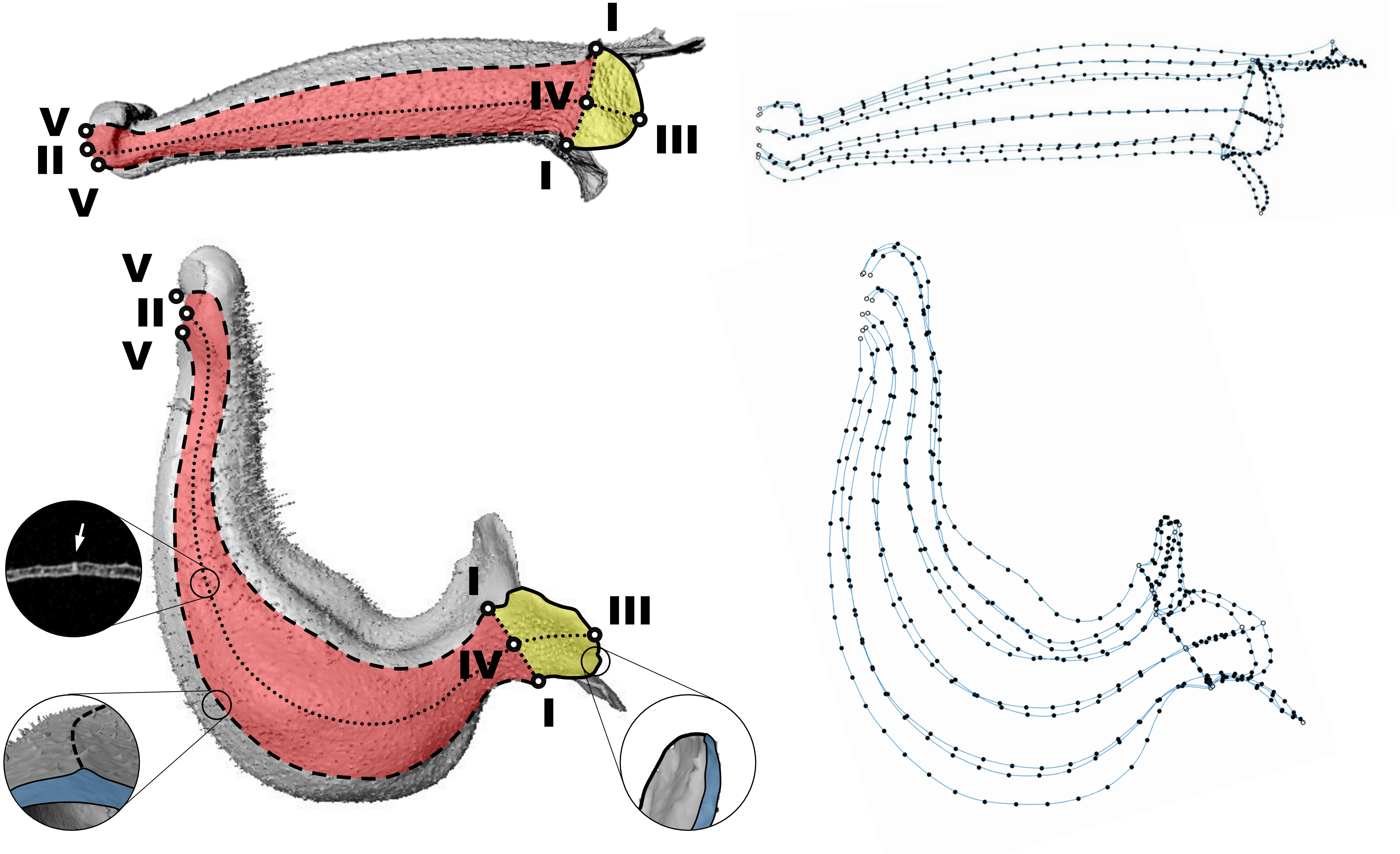

Figure 4

Click here to access/download;Figure;Fig.4_5.9.pdf

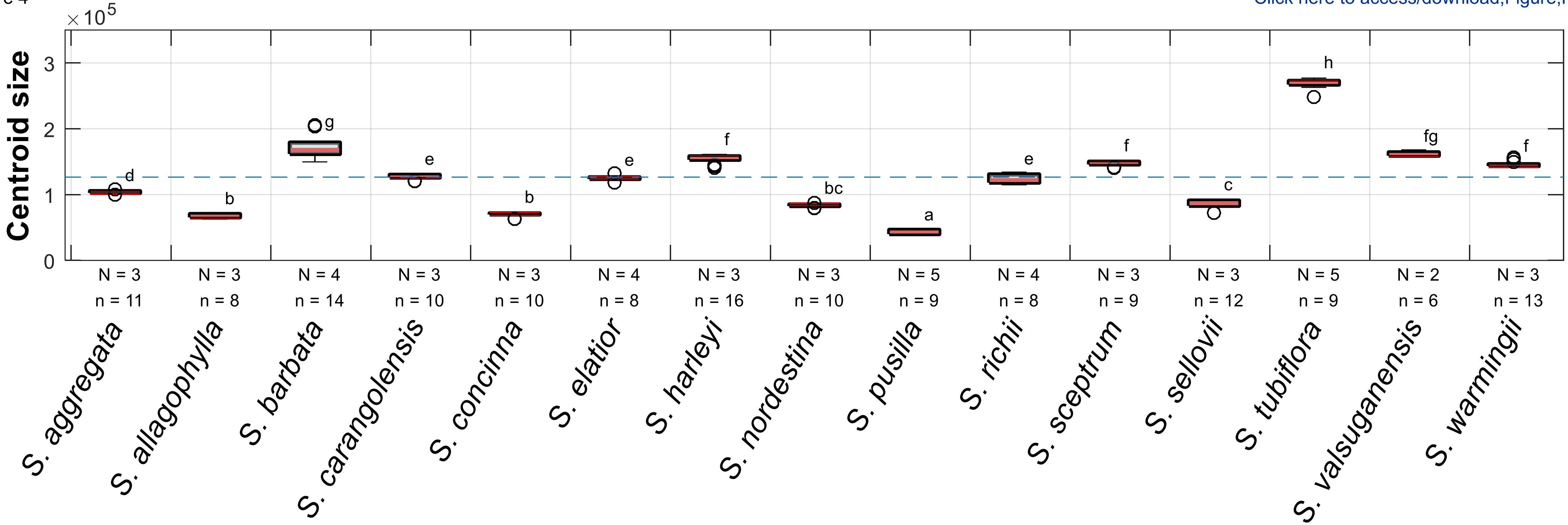

Figure 5

Click here to access/download;Figure;Fig.5_5.9.pdf

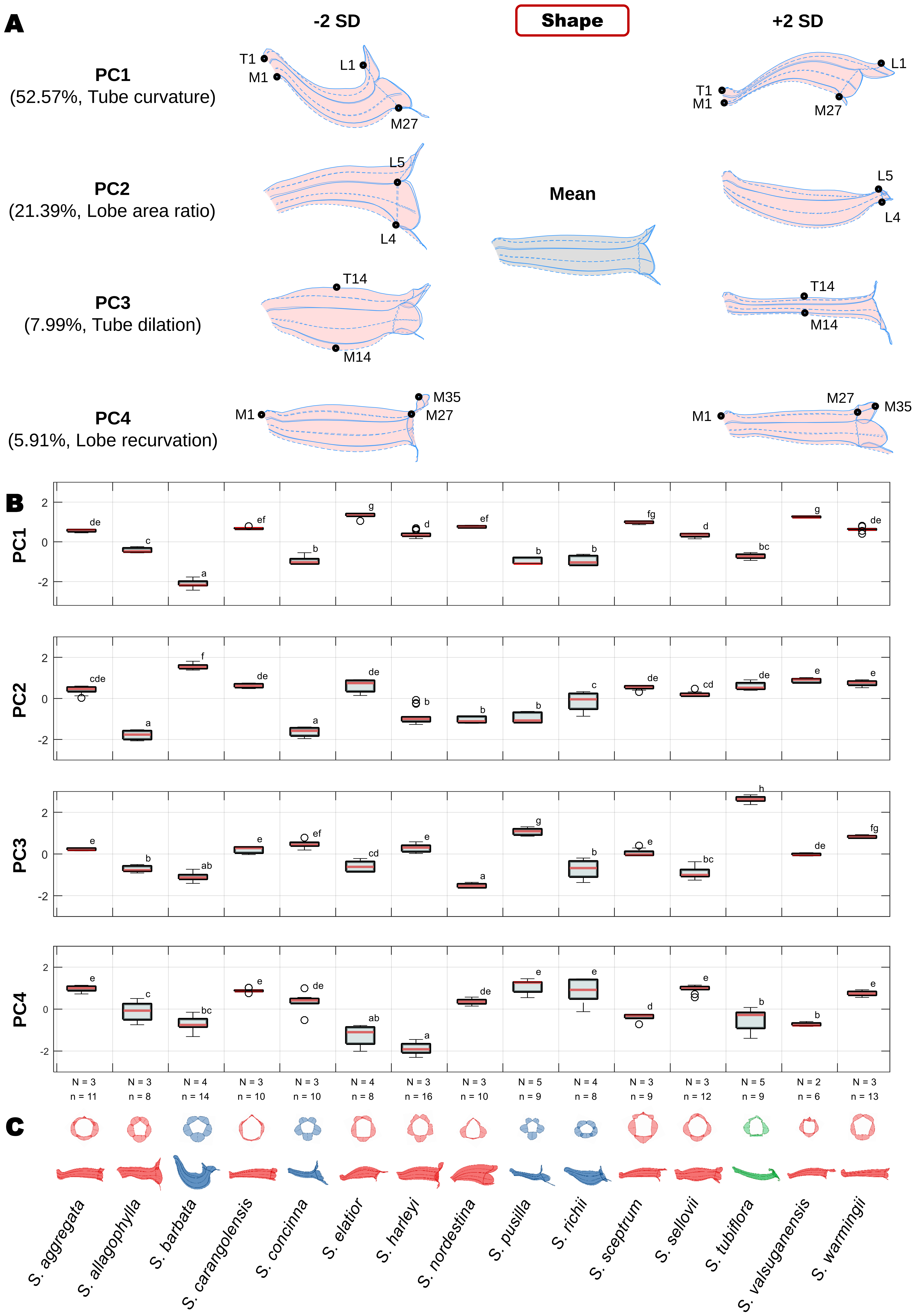

Figure 6

Click here to access/download;Figure;Fig.6_5.9.pdf

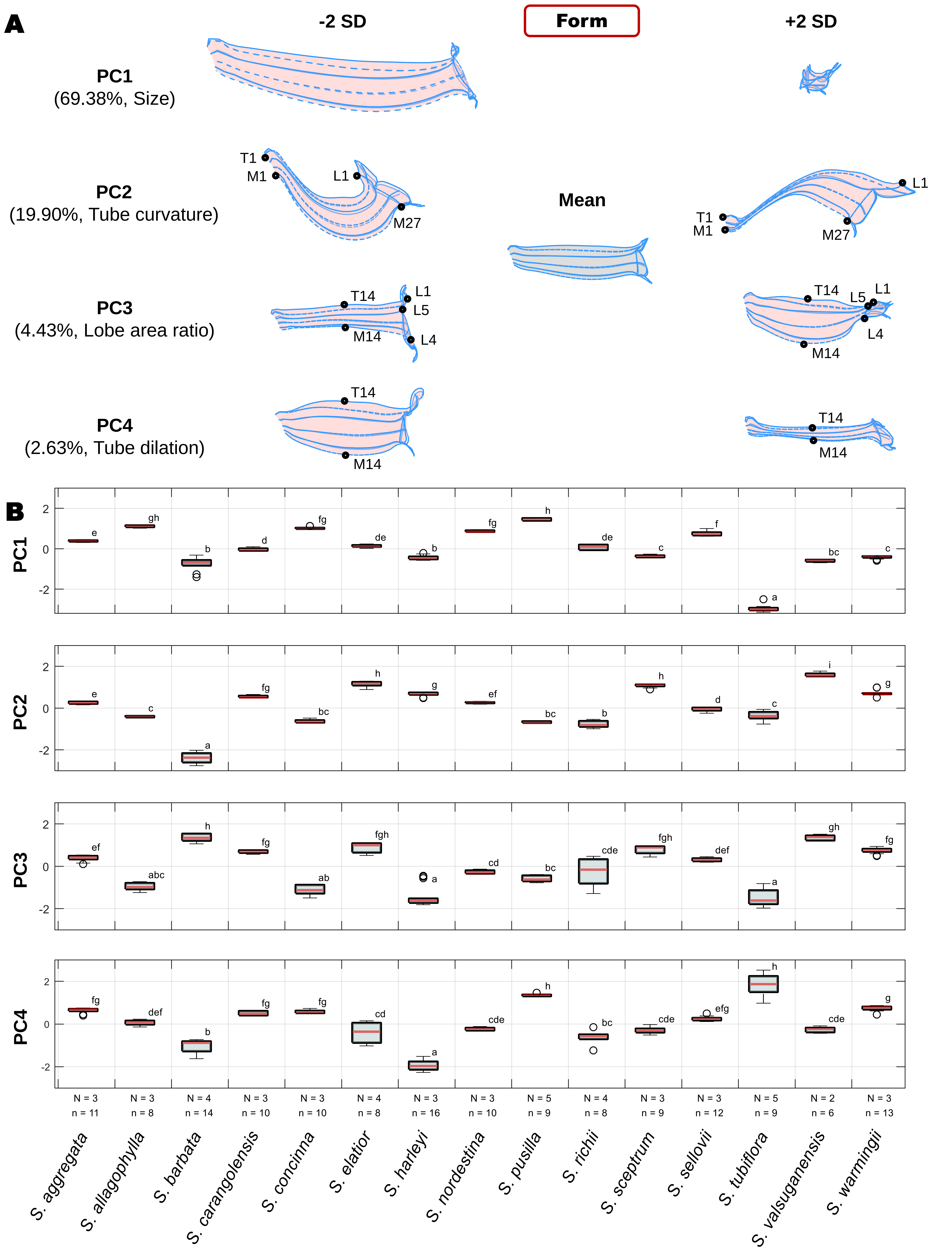

Figure 7

Click here to access/download;Figure;Fig.7_5.9.pdf

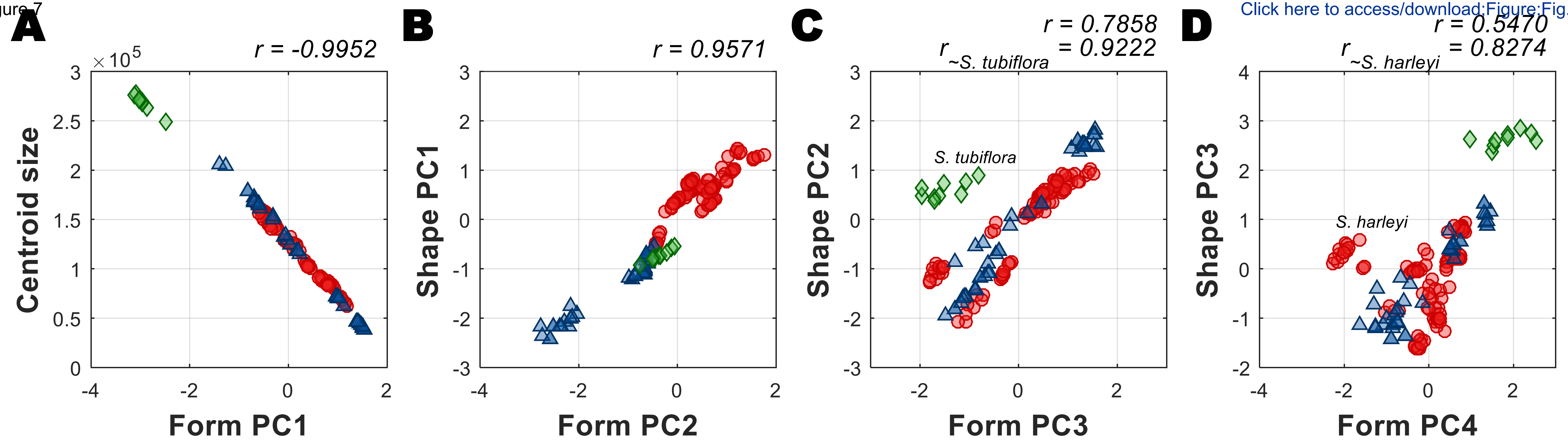

Figure 8

Click here to access/download;Figure;Fig.8_5.9.pdf

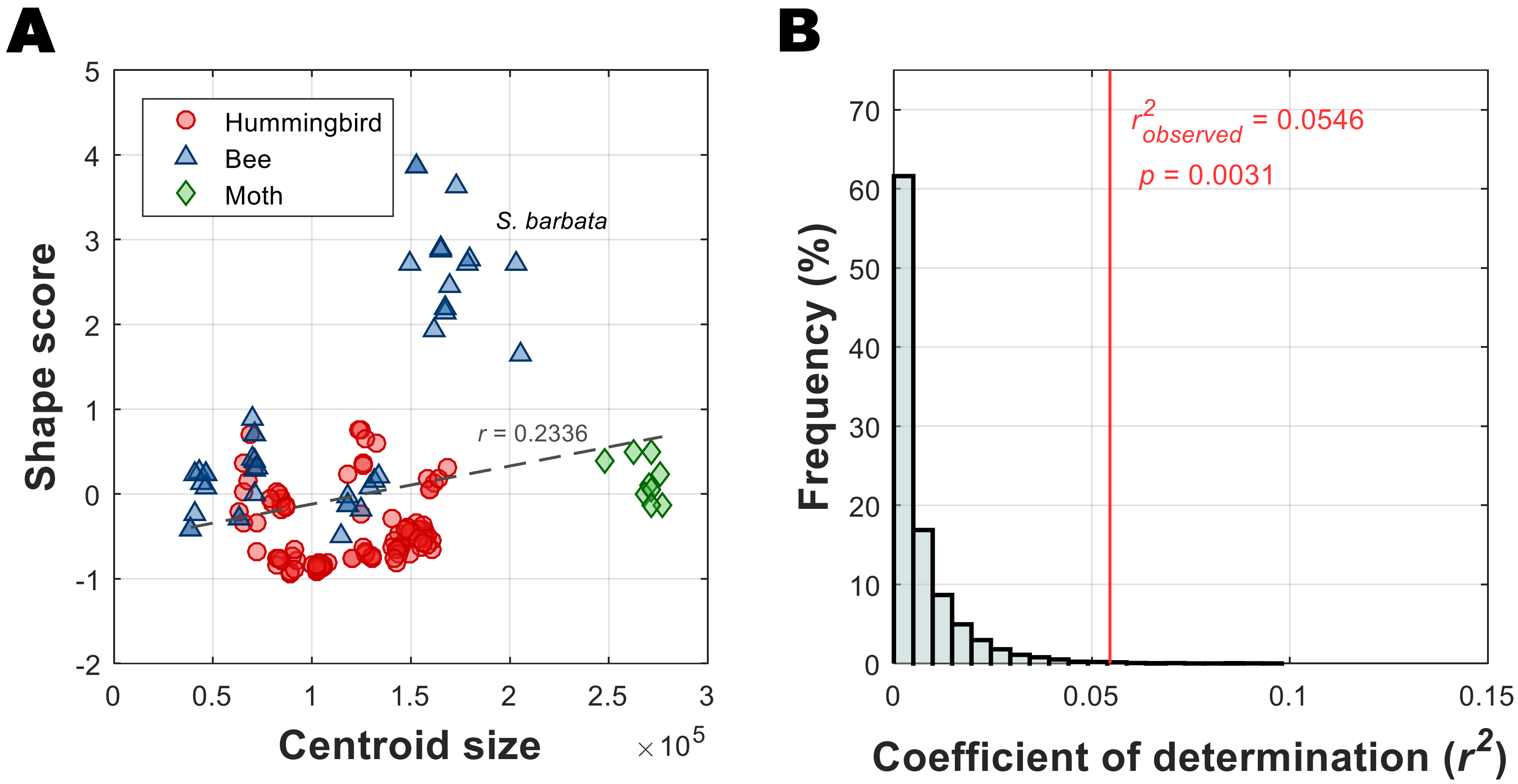

Figure 9

Click here to access/download;Figure;Fig.9_5.9.pdf

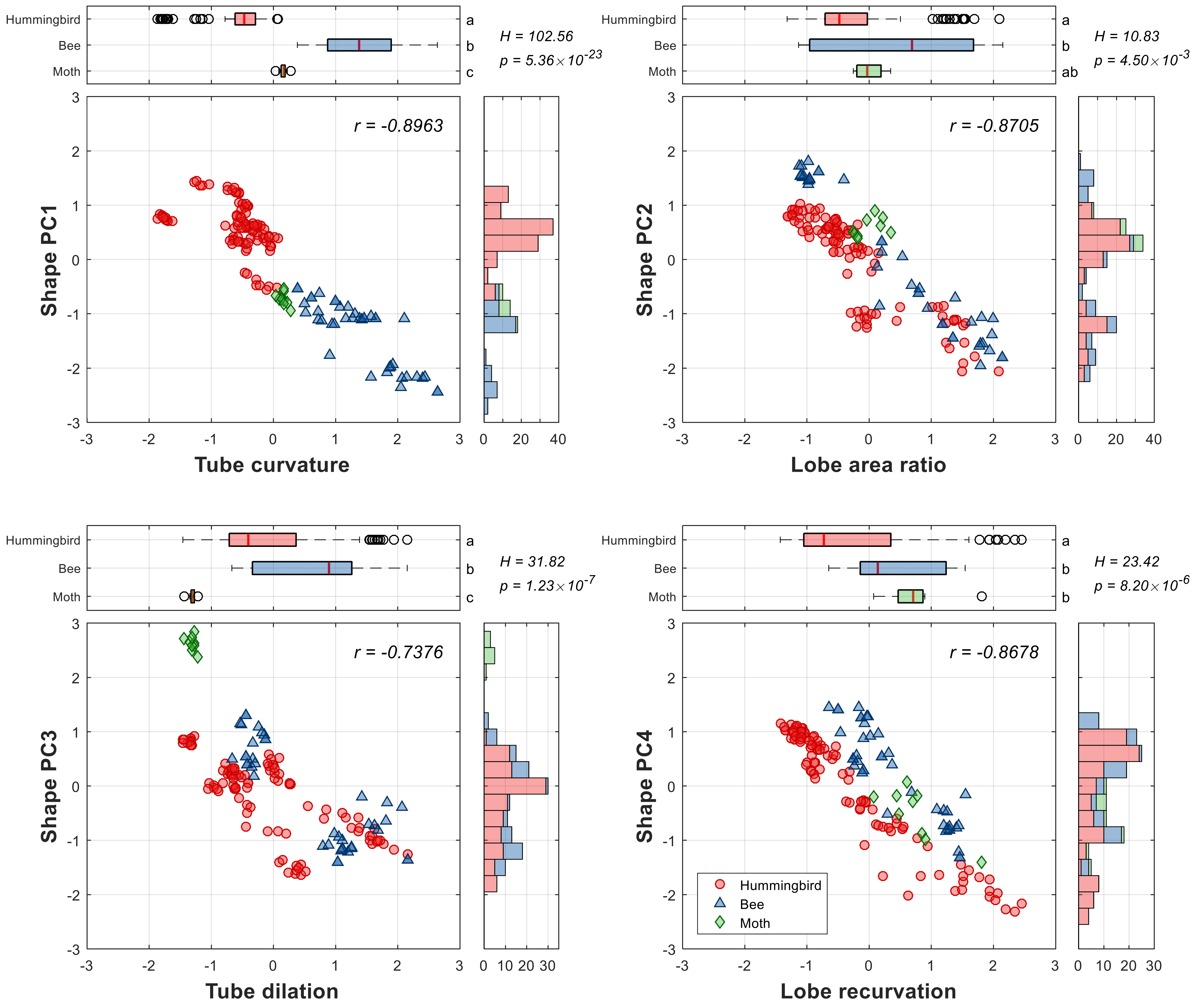

Figure 10

Click here to access/download;Figure;Fig.10_5.9.pdf

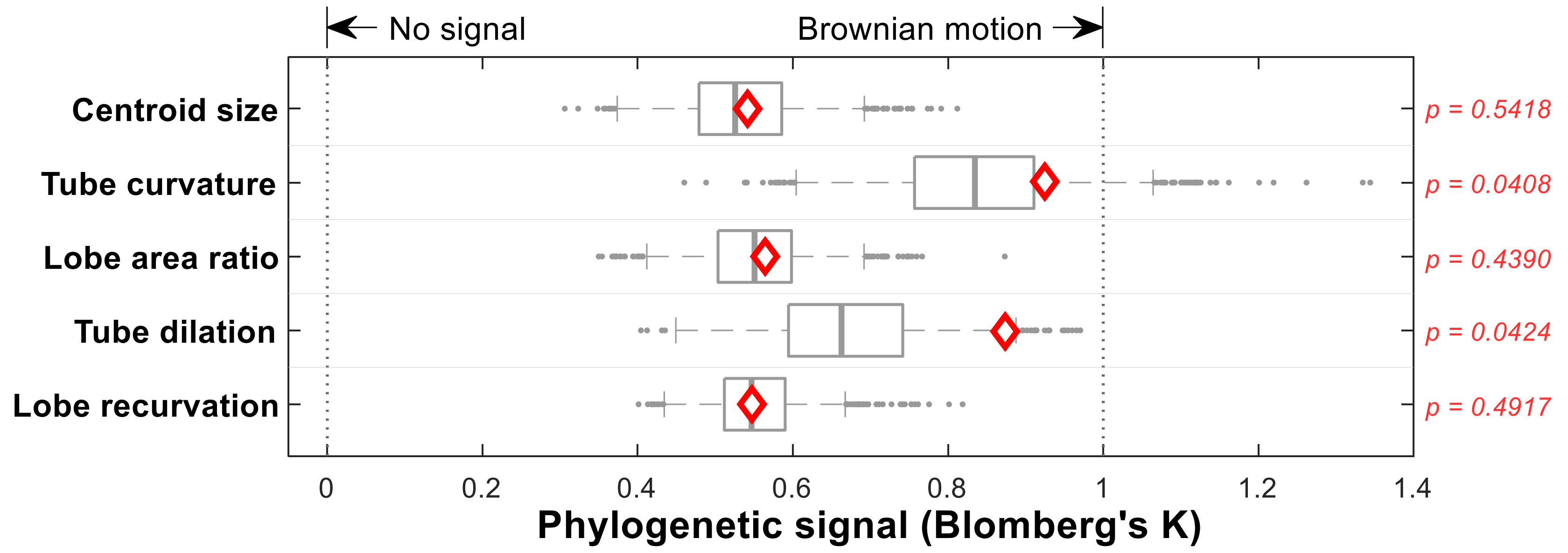

Click here to access/download;Figure;Fig.11_5.9.pdf

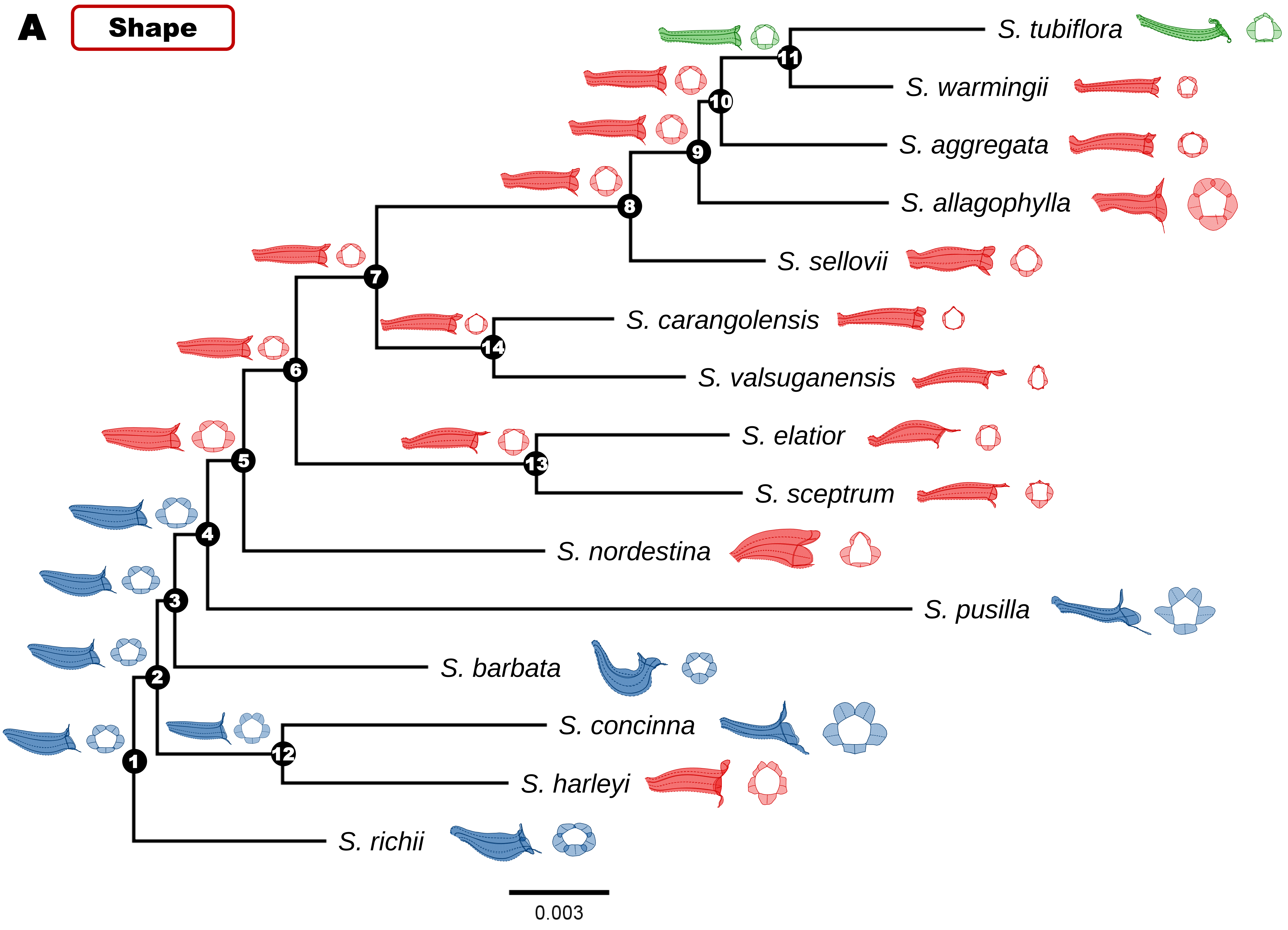

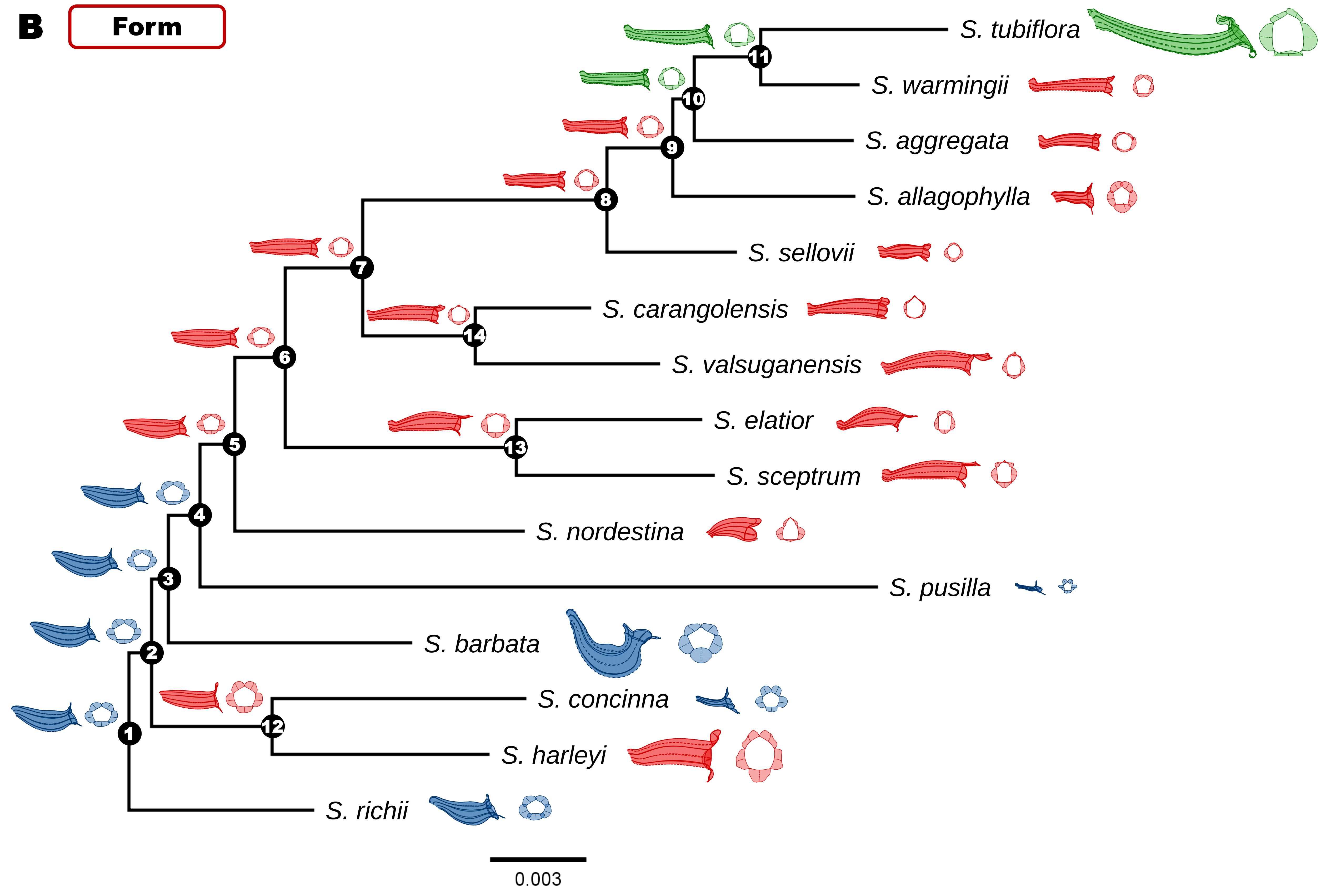

Click here to access/download;Figure;Fig.12_5.9.pdf

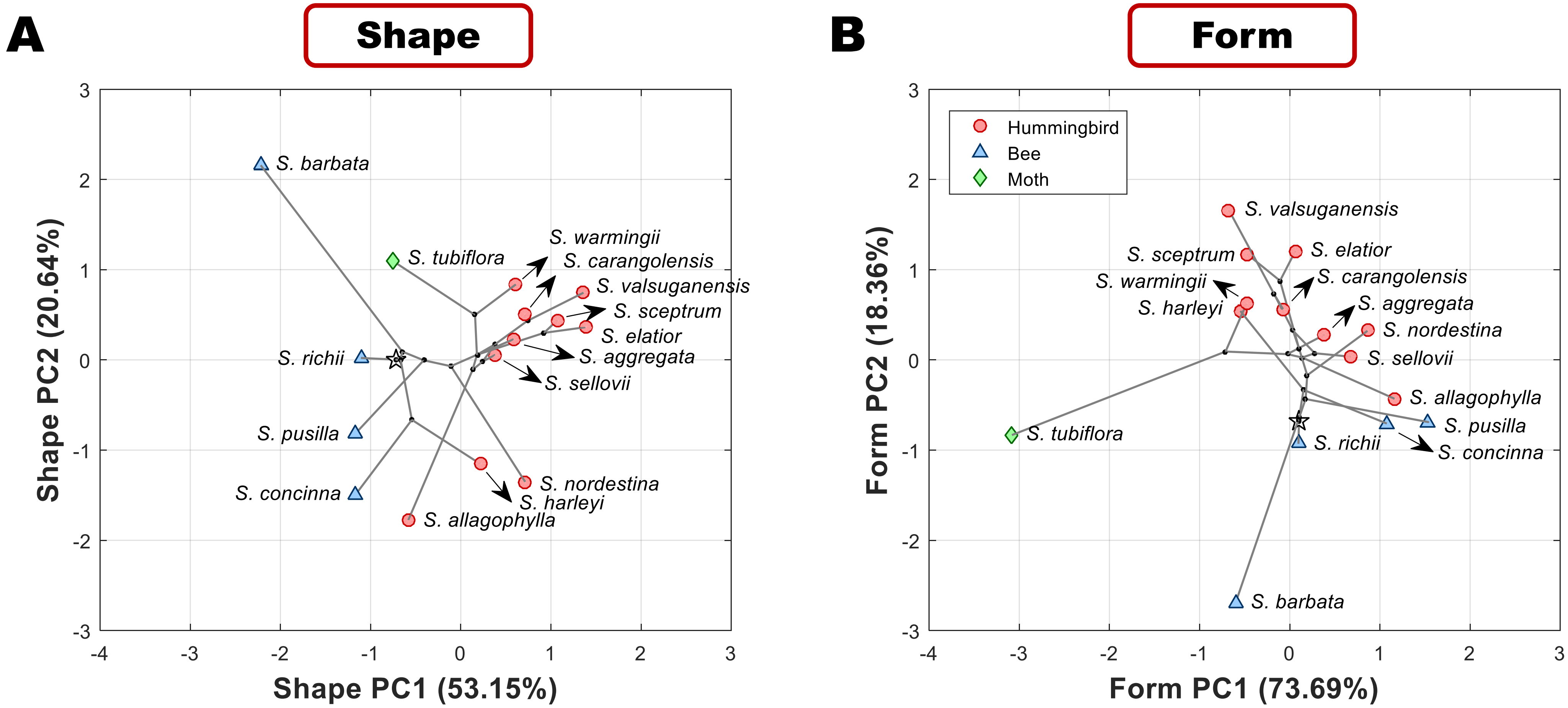

Click here to access/download;Figure;Fig.13_5.9.pdf

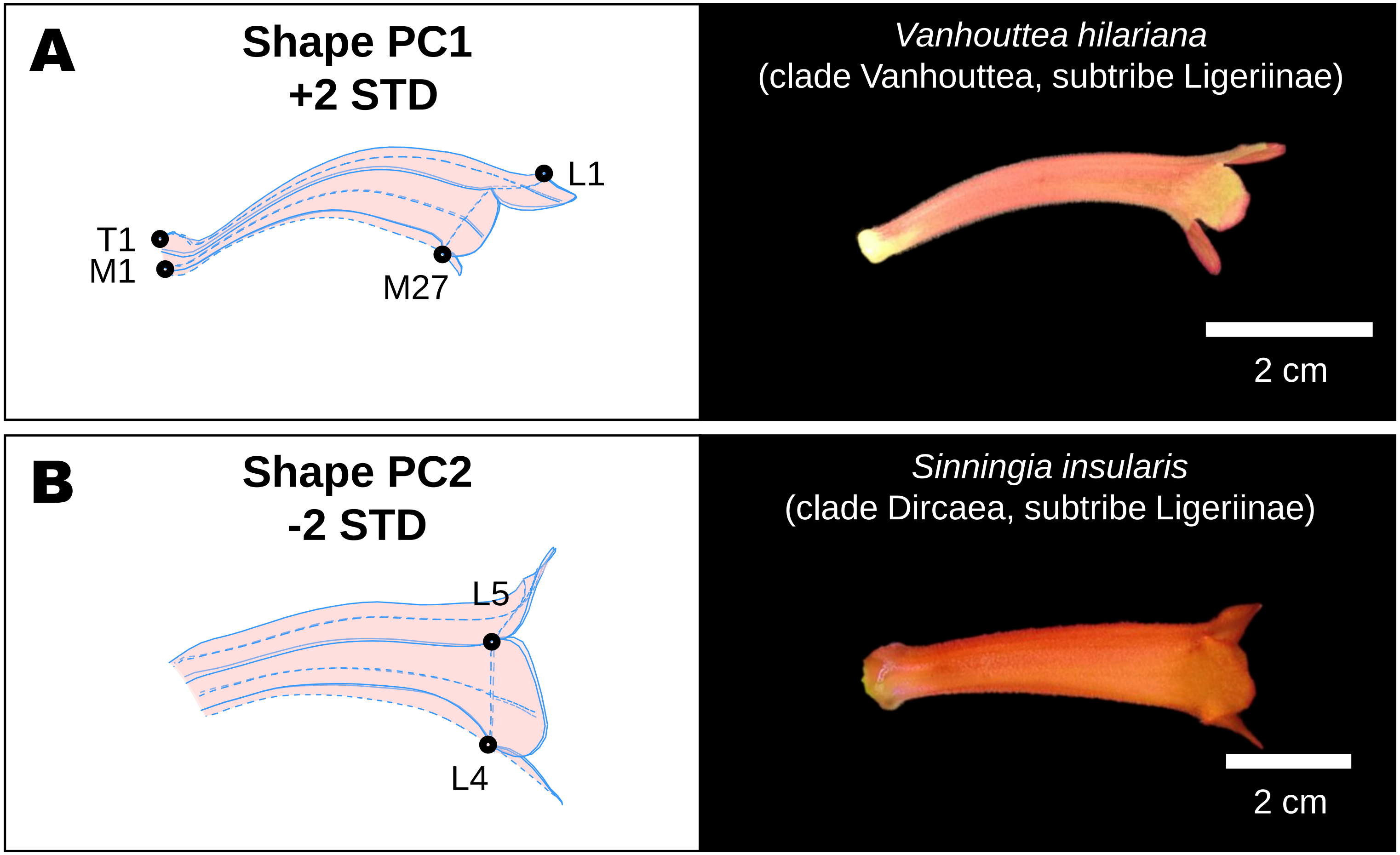

Figure 14

Click here to access/download;Figure;Fig.14_5.9.pdf

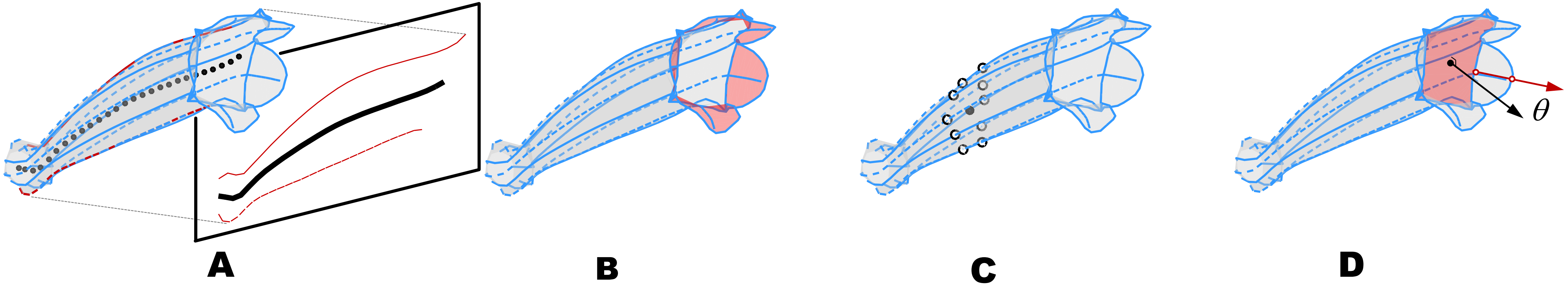

Click here to access/download
Supplementary Material
Table S1_5.9.docx

Click here to access/download
Supplementary Material
Table S2_5.9.docx

Supplementary Material, Table S3

Click here to access/download
Supplementary Material
Table S3_5.9.docx

Supplementary Material, Table S4

Click here to access/download
Supplementary Material
Table S4_5.9.docx

Supplementary Material, Figure S1

Click here to access/download
Supplementary Material
Fig.S1_5.9.pdf

Supplementary Material, Figure S2

Click here to access/download
Supplementary Material
Fig.S2_5.9.pdf

Personal Cover Click here to access/download;Personal Cover;17Genus Sinningia 3D GM_Cover Letter_revision.docx

Dr. Goodman

Editor-in-Chief

GigaScience

Sep 22, 2019

Dear Dr. Goodman,

Thank you for your time and effort on collecting the reviewers' comments for manuscript GIGA-D-19-00247, entitled "3D revelation of phenotypic variation, evolutionary allometry, and ancestral states of corolla shape: a case study of clade Corytholoma (subtribe Ligeriinae, family Gesneriaceae). We are pleased to know that our manuscript was offered minor revision as a recommendation. All the reviewers provided very constructive comments to our work. We have updated the manuscript accordingly. Attached please also find our point-by-point response to the comments raised by the reviewers.

We look forward to hearing from you at your earliest convenience.

Sincerely yours,

Yan-Fu Kuo, PhD

Associate Professor

Department of Biomechatronics Engineering

National Taiwan University

Response to reviewer

Click here to access/download;Personal Cover;17Genus Sinningia 3D GM_Rebuttal_v2_submitted.docx

Daniel H Chitwood (Reviewer 1)

Reviewer Recommendation Term:	Minor Revision
Comments to Author:	
In their manuscript "3D revelation of phenotypic variation, evolutionary allometry, and ancestral states of corolla shape: a case study of clade Corytholoma (subtribe Ligeriinae, family Gesneriaceae)", Hsu et al. present a thorough morphometric analysis of corolla shape. I very much enjoyed reading this manuscript. It integrates 2D and 3D geometric morphometrics with evolutionary theory in an unprecedented way using plant organs as an example. The morphometric analyses and the specimens are beautiful. The availability of 2D slice images, volumetric images, and surface images in the GigaScience Database repository is extremely valuable for the reproducibility of morphometric studies.<br><br>I only have minor suggestions to improve readability.	Many thanks for this very positive review.
The reader is often referred to references [8] and [11] for critical methods information. This includes: image processing information (page 10, lines 181-185), landmark selection procedure (page 28, lines 469-470), and visualization (page 29, line 505). While of course all the information doesn't need to	Thanks for the comment. The manuscript was revised accordingly. The image processing information was updated in Lines 166-179 of the revised manuscript. The landmark selection procedure was updated in Lines 188-211 of the revised manuscript. The visualization was updated in Lines 508-509 of the revised manuscript.

be repeated here, it should be summarized, so that the reader can understand the gist of what is being done without having to refer back to these publications.	
Can a full explanation of the primary landmarks be provided? Figure 13 helps, but it would be good to see the full 25 primary landmarks. It would also help to see a visualization of all secondary landmarks too, just so the reader has a visual understanding of what the data is based upon. It might help to do this for two morphologically distinct species. For those not familiar with corolla morphology in these species, a botanical/morphological/anatomical description of what the primary landmarks correspond to would help understanding.	Thanks for the insightful comment.<br><br>The Fig. 3 of the revised manuscript was updated accordingly (Line 212). In the Fig. 3, two species with distinct corolla morphology were provided.<br><br>The details corresponding to the corolla morphology, petal position, homologous features were provided in the revised manuscript (Lines 188-203).
Scaling is used for shape but not for form. Why?	Thanks for the comment.<br><br>Please let us clarify further. Form variation is defined as the variation of shape and size together, and the scale (i.e., distances) between landmarks present the size. To retain form information in the landmarks, scaling cannot apply to the landmarks. Thus, when size is considered as a critical factor (e.g., [1, 2]), partial-GPA is used to investigate the form variation.<br><br>[1] Klingenberg CP. Evolution and development of shape: integrating quantitative approaches. Nat Rev Genet. 2010;11(9):623. doi:10.1038/nrg2829<br>[2] Klingenberg CP. Size, shape, and form: concepts of allometry in geometric morphometrics. Dev Genes Evol. 2016;226(3):113-137. doi:10.1007/s00427-016-0539-2

Also, I know "shape" and "form" have strict meanings in the morphometric community. But upon first usage, it might be good to just add "2D" and "3D" for clarification, not only for those not familiar with morphometrics, but also in some non-English languages the use of "shape" and "form" is confusing.	Thanks for the comment.<br><br>Please let us clarify further. In this study, the shape and form analyses were both carried out in 3D using 3D landmarks.<br><br>We also added “3D” before shape and form throughout the revised manuscript (highlighted in green) and hoping that this will help to clarify the use of shape and form.
The methods come after the results section. But at least for the landmarks, an explanation up front might be helpful for readers to understand the analysis and interpret it.	Thanks for the insightful comment. The manuscript was updated accordingly (Lines 187-220 of the revised manuscript).

Mao Li (Reviewer 2)

Reviewer Recommendation Term:	Minor Revision
Comments to Author:	
In the manuscript "3D revelation of phenotypic variation, evolutionary allometry, and ancestral states of corolla shape: a case study of clade Corytholoma (subtribe Ligeriinae, family Gesneriaceae)", Hsu et al. applied 3D X-ray microcomputed tomography and landmark-based geometric morphometrics to study the corolla shape variation, evolutionary allometry, and some phylogenetic analysis. The manuscript is well written. The details are explained very clear. For their data, the analysis has been performed thoroughly.	Many thanks for this very positive review.
The main concern I have is the sample size. In this work, the authors studied 15 species. However, each species only has one or two plant individuals and also were collected from different years. Although five samples from this single or two plants were scanned, some of the analysis and statements cannot convince me. In my opinion, if this is a method or breakthrough technology paper, using small sample size to test and valid the method might be fine. But this manuscript is not a method paper because all the methods are existing and commonly used. For the work focusing on the biology, I would evaluate the result which should be supported by solid analysis with enough sample size. In this work, I was wondering whether this single (or two) individual plant are representative. Assume so, then I think the analysis for the major variation using PCA and the phylogenetic analysis based on the mean shape might be fine. But the result	Thanks for the insightful comment.<br><br>In short, the specimen number increases from 75 to 153 in the revised manuscript. Please allow us to explain the reason of including only 75 specimens in the original manuscript below.<br><br>We agree that a large sample size is always a good practice for analyzing the shape variations. However, 3D μCT scanning is costly and time-consuming. The specimen used for 3D scanning was preselected according to corolla shape variation analysis using 2D side views (Fig. R1 below). In the past years, we only have collected a total of 153 corolla specimens with uneven specimen numbers for each species. To assess the within-species variation, all the 153 specimens were included in the analyses of 3D shape and form variation and of evolutionary allometry. To avoid the results may be dominated by the species with larger sample size or the species with larger morphological variation, five specimens of each species selected based on shape score were used in the analyses of phylogenetic signal and of ancestral state reconstruction (Lines 147-152 of the revised

related the within-species variation cannot convince me. Can the variance in this particular plant represent the variance for the entire species? I don't think so. The variation basically is the within-plant variation. Thus the analysis and result such as Fig. 3A, Fig. 4B, 5B are supported well. In addition, Fig 3 and Fig 4B don't have the statistic.

manuscript). The Table 1, 2, Fig. 4, 5B, 6B, 7, 8, 9, S1, and S2 of the revised manuscript were updated.

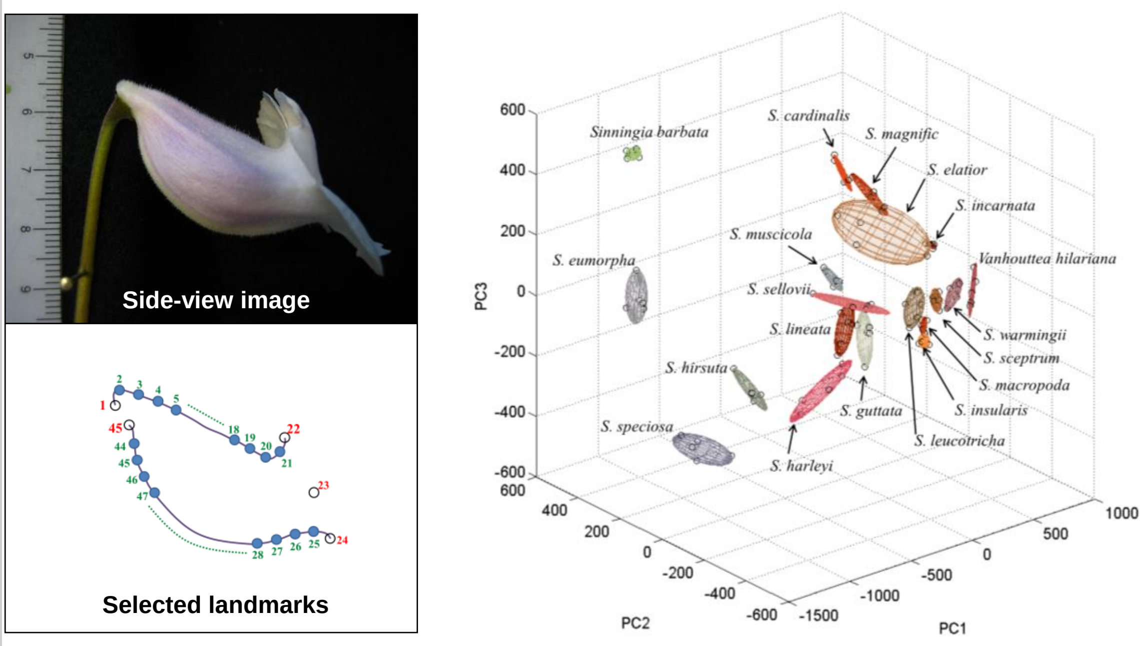

Figure R1. Illustration of 2D side-view corolla shape variation analysis. The left top was the 2D side-view image, the left bottom was the selected landmark in the image, and the right was the scatter plot of PC scores obtained from GM analysis using 2D side-view landmarks.

Per the reviewer’s suggestion, we include all the 153 specimens in the revised manuscript. Each species has at least two plant individuals. The updates are reflected in Lines 135-137 and 139-141 of the revised manuscript.

The specimen for the same species was collected in the same flowering season (Lines 141-142 of the revised manuscript). Each species has its own habits; the flowering time of most species was not synchronized.

	In Fig. 4 (Line 229), 5B (Line 256), and 6B (Line 285) of the revised manuscript, the multiple comparison tests for centroid size and PCs were provided. We hope that this will help to present the within-species variation.
Another point is about the landmark based method. First, in this work, 25 primary landmarks are selected manually first. Then the rest 390 secondary landmarks are derived computationally, but still highly rely on the manual landmarks. Thus this landmark dataset may also introduce error, and can be subjective and labor intensive. 3D X-ray images also has variance for each scan. This has the similar problem with other method such as the author mentioned for other work: (line 89) "2D images are usually acquired manually, which may introduce error or artifacts…"; (line 97) " distance-based traits are typically proposed based on manual observation and can be subjective"; (line 126) " the traits were proposed statistically rather than manually". Second, the landmark based method has been widely applied. However, it has a main limitation that you have to make sure the landmarks are homologous. I was wonder whether all the corollas have five petals. If not, how the authors made sure the landmark were homologous? Because of this limitation, similar work cannot be easily expanded to a wider range of species. It would be better to discuss a bit in the discussion.	Thanks for the insightful comment.<br><br>We revised the paragraphs of 3D flower image data (Lines 166-179), landmark identification (Line 188-211) and updated the Fig. 2 (Line 181) and 3 (Fig. 212) of the revised manuscript.<br><br>We agree that manual landmark selection may introduce error, is subjective, and is labor-intensive. μCT images are at a very high resolution (36.547 micron). The details of the corollas are clearly visible. Although all the primary landmarks and the tube-tube rims were selected manually using a software Landmark, these anatomical features are obvious and can easily be identified (Fig. 3 of the revised manuscript). Thus, the error introduced by manual selection can be minimized. The secondary landmarks were automatically selected using a software developed by our team [1]. Thus, the selection of the secondary landmarks can be objective.<br><br>The 3D imaging of each specimen was calibrated using a bar phantom (for calibrating the spatial resolution and the X-ray quality, Lines 167-168 of the revised manuscript). The scan parameters like source voltage, source current, and scan resolution were than determined (Table S2 of the revised manuscript).<br><br>In our humble opinion, the 3D approach can overcome the “subjective” issues using 2D imaging. For example, the angle of capturing the image of the object is largely controlled by the operator. This is no doubt that the process is subjective and varies from one operator to another. The error and the so-called artifacts are introduced to the analysis.<br><br>We also agree that it is critical to use homologous landmarks in landmark-based GM.

	Gesneriaceae is a family in the order Lamiales. In plant systematics, the Lamiales flower is gamopetalous (has lobe part and tube part) and consist of two dorsal petals, two lateral patels, and one ventral petal. The petal position (or the petal identity) can be identified according to the dorsal staminoid (the Lamiales flowers are alternipetalous and the dorsal stamen is underdeveloped and infertile). Thus, homologous anatomical features can be easily identified. The selected landmarks are also homologous.<br><br>[1] Wang YH, Hsu HC, Chou WC, Kuo YF. Automatically Identifying floral contours and vascular bundles in 3D images. In 2018 ASABE Annual International Meeting, 2018. (p. 1). American Society of Agricultural and Biological Engineers.
Some minor points:<br>a) At the beginning, I was confused about the "shape" and "form". It got clear after reading several pages. It would be nice to clarify a bit at the beginning.<br><br>b) (Line 78) Although this table is a very nice literature summary, I feel it is not directly related to this study. The authors could consider to move it to supplement.<br><br>c) (Line 141) I cannot find Appendix 1.<br><br>d) (Line 167) From this summary, it looks like the color has strong correlation with the pollination types. If the authors have the 2D images as well, additional analysis to associate color and pollination types can be done.	a) Thanks for the comment. We annotated the form refers to shape and size together at the beginning of the Background (Lines 51 of the revised manuscript).<br><br>b) Thanks for the comment. The table was moved to supplement in the revised manuscript (Line 770).<br><br>c) The irrelevant text was remove in the revised manuscript.<br><br>d) Thanks for the insightful comment. Indeed, the previous study, Perret et al. (2003), indicated that, apart from the corolla shape, the corolla color would be one of the important factor corresponding to the pollination type. In this study, we focus on the variation of corolla shape and aim to identify its shape trait in 3D. We removed the color information in the revised manuscript.<br><br>We have other in-progress research related to flower color and color pattern on these

species (Fig. R2). The preliminary results showed that the corolla color and color pattern are also complex and their association with pollination type is not straightforward and not intuitive (Fig. R3). We would like to publish the finding on the corolla color as an independent research article.

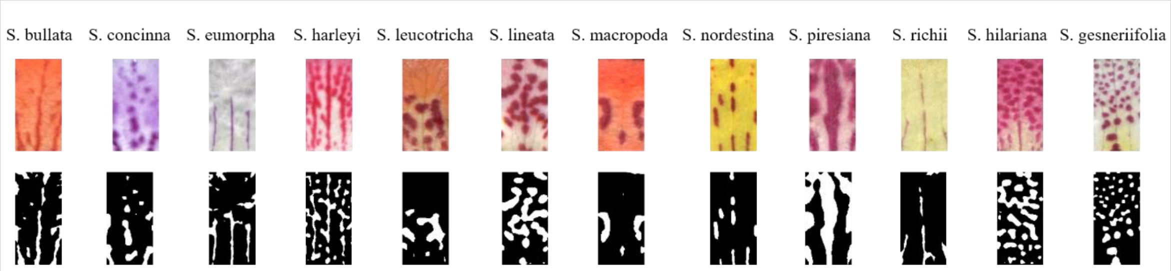

Figure R2. Foreground-background separation for ventral petals. The upper panel is the original image. The bottom panel is the foreground-background separated mask, the white region is foreground, and the black region is background.

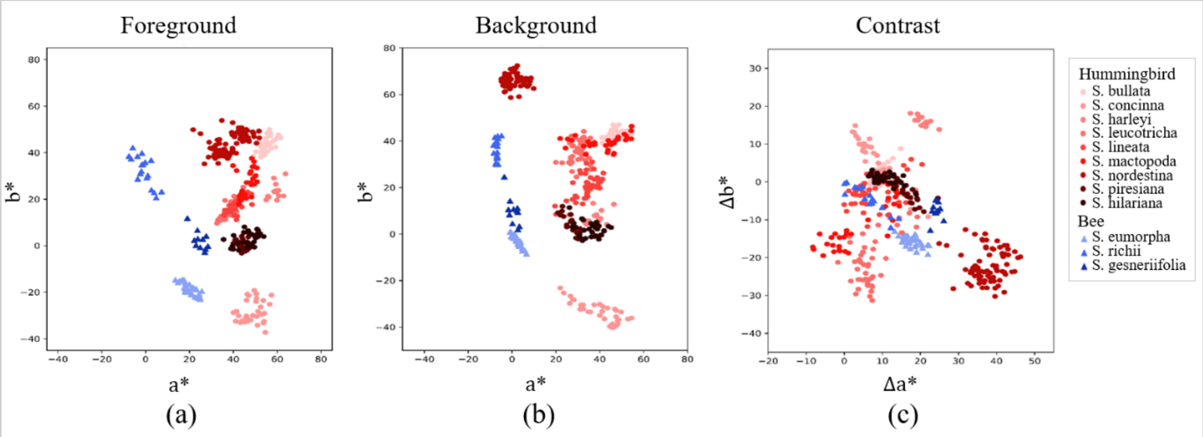

Figure R3. (a) Foreground color, (b) background color, and (c) contrast of the variegated species. Red: hummingbird-pollinated species; Blue: Bee-pollinated species.

e) (Line 200) No indication of "the average" in the figure.

e) The Fig. 4 were updated in the revised manuscript (Lines 225 and 229).

f) (Line 204) Fig 3B was not mentioned anywhere in the manuscript.	f) The figure was removed accordingly (Fig. 4 of the revised manuscript, Line 229).
g) (Line 274) I know "shape scores" will be explained in the method section later, but it appeared too suddenly here. Please either explain a bit here or guide readers to the method section.	g) Thanks for the comment. The manuscript was revised accordingly (Line 315-316 of the revised manuscript).
h) (Line 276), I know p=0.0625 is closed to 0.05, but instead of "significant", people normally say it is not statistical significant	h) Thanks for the insightful comment. Per your comment on the sample size, we increased the number of specimen from 75 to 153 and re-analyzed the evolutionary allometry. The p = 0.0031 in the revised manuscript (Lines 304-306).
i) (Line 305) the p value is only for the tube curvature.	i) Thanks for the comment. The manuscript was revised accordingly (Line 335-336 of the revised manuscript).
j) (Line 310) Cannot see the last column of the Table 3.	j) The column size was adjusted (Line 339 of the revised manuscript). The full table 2 was also submitted separately of the file inventory for this manuscript.
k) (Line 350) How to explain the difference of pollination types at nodes 10 and 12 between shape and form?	k) Thanks for the insightful comment. Due to the analyses of shape and form were based on the sPCs and fPCs, the difference between two PCs was expected to be the size variation. Thus, the intuitive explanation for the difference of pollination types at nodes 10 and 12 between shape and form would be the size difference (Line 380-381 of the revised manuscript).
l) In the method section, authors used different number of permutations (e.g. 10,000 times, 1000 times, 100 times) for different methods. I just wonder how authors picked these numbers.	l) The reshuffle times are increased to 10,000 for the three permutation tests in the revised manuscript. The relevant information was updated in the revised manuscript (Line 522, 569, and 599).
