# Supplementary material for: 3D revelation of phenotypic variation, evolutionary allometry, and ancestral states of corolla shape: a case study of clade Corytholoma (subtribe Ligeriinae, family Gesneriaceae)
Source: Gigascience. 2020 Jan 22;9(1):giz155. doi: 10.1093/gigascience/giz155 (PMC6974915; doi:10.1093/gigascience/giz155)
Supplement: giz155_Supplemental_Figures_and_Tables [file giz155_supplemental_figures_and_tables.zip › Table S1_6.2.docx]

Table S1. List of studies using landmark-based GM.

| Approach | Imaging object | Keywords (selected) | Family | Landmark* | | GM | | References |
| --- | --- | --- | --- | --- | --- | --- | --- | --- |
|  |  |  |  | Primary | Secondary | GPA | PCA/LDA |  |
| 2D | Side-view | Petal shape; Floral morphology | Gesneriaceae | 5 | 10 | Full | PCA | [1] |
|  |  | Pollination syndrome; Phylogenetic comparative methods | Gesneriaceae | 6 | 26 | Full | PCA | [2, 3] |
|  |  | Pollinator selection; Flower shape | Loasaceae | 5 | 0 | Full | PCA, LDA | [4, 5] |
|  |  | Flower shape; Pollination niches | Plantaginaceae | 4 | 12 | Full | PCA | [6] |
|  |  | Trait-dependent diversification; Pollination; Flower tube | Plantaginaceae | 9 | 1 | Full | LDA | [7] |
|  |  | Ancillary traits | Rubiaceae | 9 | 8 | Full | PCA, LDA | [8] |
|  | Face-view | Selfing syndrome | Brassicaceae | 30 | 0 | Full | PCA | [9] |
|  |  | Floral shape evolution; pollination; corolla shape; plant-pollinator interactions; floral morphospace; allometry | Brassicaceae | 32 | 0 | Full | PCA | [10-15] |
|  |  | Petal shape; Floral morphology | Gesneriaceae | 5 | 25 | Full | PCA | [1] |
|  |  | Floral shape** | Goodeniaceae | 5 | 0 | Full | PCA | [16] |
|  |  | Fluctuating asymmetry | Orchidaceae | 4 | 5 | Full | PCA | [17] |
|  |  | Flower shape | Plantaginaceae | 28 | 0 | Full | PCA | [18] |
|  |  | Corolla shape; pollination | Solanaceae | 5 | 35 | Full | PCA | [19} |
|  |  | Floral symmetry | Valerianaceae | 10 | 0 | Full | LDA | [20] |
|  | Dissected-view | Actinomorphy; Zygomorphy | Leguminosae | 2 | 18 | Full | PCA | [21] |
|  |  | Floral shape** | Leguminosae | 37 | 0 | Full | PCA | [22] |
|  |  | Directional asymmetry; floral organ shape; fluctuating asymmetry | Iridaceae | 39 | 16 | Full | PCA | [23, 24] |
|  |  | Floral shape** | Orchidaceae | 15 | 0 | Full | PCA | [25] |
|  |  | Floral shape, allometry** | Plantaginaceae | 4 | 16 | Partial | PCA | [26] |
|  |  | Floral shape** | Plantaginaceae | 8 | 47 | Partial | PCA | [27] |
| 3D | Whole corolla | Petal shape; dorsoventral asymmetry | Gesneriaceae | 10 | 65 | Full | PCA | [28] |
|  |  | Dorsoventral asymmetry; petal form variation | Gesneriaceae | 25 | 100 | Full | PCA | [29] |
|  |  | Floral shape, pollination** | Orchidaceae | 26 | 14 | Full | PCA | [30] |
|  |  | Corolla shape variations | Gesneriaceae | 25 | 390 | Full/ Partial | PCA | Present study |

* The primary and secondary landmarks follow the definitions in the Methods section.

** Keywords were not provided in these studies. The keywords were assigned by authors of the present study.

1. Hsu HC, Chen CY, Lee TK, Weng LK, Yeh DM, Lin TT., et al. Quantitative analysis of floral symmetry and tube dilation in an F2 cross of Sinningia speciosa. Sci Hort. 2015;188:71-77. doi:10.1016/j.scienta.2015.03.0

2. Alexandre H, Vrignaud J, Mangin B, Joly S. Genetic architecture of pollination syndrome transition between hummingbird-specialist and generalist species in the genus Rhytidophyllum (Gesneriaceae). PeerJ; 2015;3:e1028. doi:10.7717/peerj.1028

3. Joly S, Lambert F, Alexandre H, Clavel J, Léveillé‐Bourret É, Clark JL. Greater pollination generalization is not associated with reduced constraints on corolla shape in Antillean plants. Evolution. 2018;72(2):244-260. doi:10.1111/evo.13410

4. Strelin MM, Benitez-Vieyra S, Ackermann M, Cocucci AA. Flower reshaping in the transition to hummingbird pollination in Loasaceae subfam. Loasoideae despite absence of corolla tubes or spurs. Evol Ecol. 2016;30(3):401-417.

5. Strelin MM, Benitez‐Vieyra S, Fornoni J, Klingenberg CP, Cocucci A. The evolution of floral ontogenetic allometry in the Andean genus Caiophora (Loasaceae, subfam. Loasoideae). Evol Dev. 2018;20(1):29-39. doi:10.1111/ede.12246

6. Blanco‐Pastor JL, Ornosa C, Romero D, Liberal IM, Gómez JM, Vargas P. Bees explain floral variation in a recent radiation of Linaria. J Evolution Biol. 2015;28(4):851-863. doi:10.1111/jeb.12609

7. Fernández-Mazuecos M, Blanco-Pastor JL, Gómez JM, Vargas P. Corolla morphology influences diversification rates in bifid toadflaxes (Linaria sect. Versicolores). Ann Bot. 2013;112(9):1705-1722. doi:10.1093/aob/mct214

8. Hernández-Ramírez AM, Aké-Castillo JA. A geometric morphometrics study of stigma-anther polymorphism in the tropical distylous Palicourea padifolia (Rubiaceae). Am J Plant Sci. 2014;5(10):1449. doi:10.4236/ajps.2014.510160

9. Carleial S, Van Kleunen M, Stift M. Small reductions in corolla size and pollen: ovule ratio, but no changes in flower shape in selfing populations of the North American Arabidopsis lyrata. Oecologia. 2017;183(2):401-413. doi: 10.1007/s00442-016-3773-4

10. Gómez JM, Perfectti F, Camacho JPM. Natural selection on Erysimum mediohispanicum flower shape: insights into the evolution of zygomorphy. Am Nat. 2006;168(4):531-545. doi:10.1086/507048

11. Gómez JM, Bosch J, Perfectti F, Fernández JD, Abdelaziz M, Camacho JPM. Spatial variation in selection on corolla shape in a generalist plant is promoted by the preference patterns of its local pollinators. Proc R Soc B. 2008;275(1648):2241-2249. doi:10.1098/rspb.2008.0512

12. Gómez JM, Abdelaziz M, Muñoz‐Pajares J, Perfectti F. Heritability and genetic correlation of corolla shape and size in Erysimum mediohispanicum. Evolution. 2009;63(7):1820-1831. doi:10.1111/j.1558-5646.2009.00667.x

13. Savriama Y, Gómez JM, Perfectti F, Klingenberg CP. Geometric morphometrics of corolla shape: dissecting components of symmetric and asymmetric variation in Erysimum mediohispanicum (Brassicaceae). New Phytol. 2012;196(3):945-954. doi:10.1111/j.1469-8137.2012.04312.x

14. Gómez JM, Perfectti F, Lorite J. The role of pollinators in floral diversification in a clade of generalist flowers. Evolution. 2015;69(4):863-878.

15. Gómez JM, Torices R, Lorite J, Klingenberg CP, Perfectti F. The role of pollinators in the evolution of corolla shape variation, disparity and integration in a highly diversified plant family with a conserved floral bauplan. Ann Bot. 2016;117(5):889-904. doi:10.1093/aob/mcv194

16. Gardner AG, Gerald JNF, Menz J, Shepherd KA, Howarth DG, Jabaily RS. Characterizing floral symmetry in the Core Goodeniaceae with geometric morphometrics. PLoS One. 2016;11(5):e0154736. doi:10.1371/journal.pone.0154736

17. Shipunov AB, Bateman RM. Geometric morphometrics as a tool for understanding Dactylorhiza (Orchidaceae) diversity in European Russia. ‎Biol J Linn Soc. 2005;85(1):1-12. doi:10.1111/j.1095-8312.2005.00468.x

18. Baranzelli MC, Johnson LA, Cosacov A, Sérsic AN. Historical and ecological divergence among populations of Monttea chilensis (Plantaginaceae), an endemic endangered shrub bordering the Atacama Desert, Chile. Evol Ecol. 2014;28(4):751-774. doi:10.1007/s10682-014-9694-y

19. Kaczorowski RL, Seliger AR, Gaskett AC, Wigsten SK, Raguso RA. Corolla shape vs. size in flower choice by a nocturnal hawkmoth pollinator. Funct Ecol. 2012;26(3):577-587. doi:10.1111/j.1365-2435.2012.01982.x

20. Berger BA, Ricigliano VA, Savriama Y, Lim A, Thompson V, Howarth DG. Geometric morphometrics reveals shifts in flower shape symmetry and size following gene knockdown of CYCLOIDEA and ANTHOCYANIDIN SYNTHASE. BMC plant biol. 2017;17(1):205. doi:10.1186/s12870-017-1152-x

21. Sinjushin AA, Bagheri A, Maassoumi AA, Rahiminejad MR. Terata of two legume species with radialized corolla: some correlations in floral symmetry. Plant Syst. Evol. 2015;301(10):2387-2397. doi:10.1007/s00606-015-1235-9

22. Püschel TA, Espejo J, Sanzana MJ, Benítez HA. Analysing the floral elements of the lost tree of Easter Island: a morphometric comparison between the remaining ex-situ lines of the endemic extinct species Sophora toromiro. PloS One. 2014;9(12):e115548. doi:10.1371/journal.pone.0115548

23. Radović S, Urošević A, Hočevar K, Vuleta A, Manitašević Jovanović S, Tucić B. Geometric morphometrics of functionally distinct floral organs in Iris pumila: Analyzing patterns of symmetric and asymmetric shape variations. Arch Biol Sci. 2017;69(2):223-231. doi:10.2298/ABS160912086R

24. Tucić B, Budečević S, Manitašević Jovanović S, Vuleta A, Klingenberg CP. Phenotypic plasticity in response to environmental heterogeneity contributes to fluctuating asymmetry in plants: first empirical evidence. J Evolution Biol. 2018;31(2):197-210. doi:10.1111/jeb.13207

25. Dalayap RM, Torres MAJ, Demayo CG. Landmark and outline methods in describing petal, sepal and labellum shapes of the flower of Mokara orchid varieties. Int J Agric Biol. 2011;13:652-658. doi:11-106/AWB/2011/13-5-652-658

26. Feng X, Wilson Y, Bowers J, Kennaway R, Bangham A, Hannah A, et al. Evolution of allometry in Antirrhinum. Plant Cell. 2009;21(10):2999-3007. doi:10.1105/tpc.109.069054

27. Cui ML, Copsey L, Green AA, Bangham JA, Coen E. Quantitative control of organ shape by combinatorial gene activity. PLoS Biol. 2010;8(11):e1000538. doi:10.1371/journal.pbio.1000538

28. Wang CN, Hsu HC, Wang CC, Lee TK, Kuo YF. Quantifying floral shape variation in 3D using microcomputed tomography: a case study of a hybrid line between actinomorphic and zygomorphic flowers. Front Plant Sci. 2015;6:724. doi:10.3389/fpls.2015.00724

29. Hsu HC, Wang CN, Liang CH, Wang CC, Kuo YF. Association between petal form variation and CYC2-like genotype in a hybrid line of Sinningia speciosa. Front Plant Sci. 2017;8:558. doi:10.3389/fpls.2017.00558

30. van der Niet T, Zollikofer CP, de León MSP, Johnson SD, Linder HP. Three-dimensional geometric morphometrics for studying floral shape variation. Trends Plant Sci. 2010;15(8):423-426. doi:10.1016/j.tplants.2010.05.005
