# Supplementary material for: 3D revelation of phenotypic variation, evolutionary allometry, and ancestral states of corolla shape: a case study of clade Corytholoma (subtribe Ligeriinae, family Gesneriaceae)
Source: Gigascience. 2020 Jan 22;9(1):giz155. doi: 10.1093/gigascience/giz155 (PMC6974915; doi:10.1093/gigascience/giz155)
Supplement: giz155_Supplemental_Figures_and_Tables [file giz155_supplemental_figures_and_tables.zip › Table S2_6.2.docx]

Table S2. The specimen information and the scan parameters

| **Species** | **Isolate** | **Specimen ID** | **Scan parameters** | | | **Specimen use*** |
| --- | --- | --- | --- | --- | --- | --- |
|  |  |  | **Voltage (kV)** | **Current (μA)** | **Resolution (μm)** |  |
| *Sinningia aggregata* | K039091 | K039091_07 | 49 | 200 | 36.54704 | G, E, P, A |
|  |  | K039091_08 | 49 | 200 | 36.54704 | G, E |
|  |  | K039091_09 | 49 | 200 | 36.54704 | G, E, P, A |
|  | K039092 | K039092_01 | 40 | 250 | 36.54703 | G, E |
|  |  | K039092_02 | 40 | 250 | 36.54703 | G, E |
|  |  | K039092_03 | 40 | 250 | 36.54703 | G, E |
|  |  | K039092_04 | 49 | 200 | 36.54704 | G, E |
|  |  | K039092_05 | 49 | 200 | 36.54704 | G, E, P, A |
|  | K039093 | K039093_01 | 49 | 200 | 36.54704 | G, E |
|  |  | K039093_02 | 49 | 200 | 36.54704 | G, E, P, A |
|  |  | K039093_03 | 49 | 200 | 36.54704 | G, E, P, A |
| *Sinningia allagophylla* | HC0909-d | HC0909-d_09 | 40 | 250 | 36.54703 | G, E, P, A |
|  | K039099 | K039099_01 | 40 | 250 | 36.54703 | G, E |
|  |  | K039099_02 | 40 | 250 | 36.54703 | G, E |
|  |  | K039099_03 | 40 | 250 | 36.54703 | G, E, P, A |
|  |  | K039099_04 | 40 | 250 | 36.54703 | G, E, P, A |
|  |  | K039099_05 | 40 | 250 | 36.54703 | G, E |
|  | K039100 | K039100_07 | 40 | 250 | 36.54704 | G, E, P, A |
|  |  | K039100_08 | 40 | 250 | 36.54704 | G, E, P, A |
| *Sinningia barbata* | HC1206-a | HC1206-a_02 | 49 | 200 | 36.54703 | G, E, P, A |
|  |  | HC1206-a_03 | 49 | 200 | 36.54703 | G, E, P, A |
|  |  | HC1206-a_09 | 49 | 200 | 36.54703 | G, E |
|  | HC1206-d | HC1206-d_06 | 40 | 250 | 36.54704 | G, E |
|  |  | HC1206-d_07 | 40 | 250 | 36.54704 | G, E |
|  |  | HC1206-d_08 | 40 | 250 | 36.54704 | G, E |
|  |  | HC1206-d_09 | 40 | 250 | 36.54703 | G, E, P, A |
|  |  | HC1206-d_10 | 40 | 250 | 36.54703 | G, E, P, A |
|  | K039104 | K039104_02 | 49 | 200 | 36.54703 | G, E |
|  | K039105 | K039105_01 | 49 | 200 | 36.54703 | G, E |
|  |  | K039105_02 | 40 | 250 | 36.54704 | G, E |
|  |  | K039105_03 | 40 | 250 | 36.54704 | G, E, P, A |
|  |  | K039105_04 | 40 | 250 | 36.54704 | G, E |
|  |  | K039105_05 | 40 | 250 | 36.54704 | G, E |
| *Sinningia carangolensis* | HC1912-2 | HC1912-2_02 | 40 | 250 | 36.54703 | G, E, P, A |
|  |  | HC1912-2_03 | 40 | 250 | 36.54703 | G, E, P, A |
|  |  | HC1912-2_04 | 40 | 250 | 36.54703 | G, E, P, A |
|  | HC1912-b | HC1912-b_01 | 40 | 250 | 36.54703 | G, E |
|  |  | HC1912-b_08 | 40 | 250 | 36.54704 | G, E |
|  | K039112 | K039112_01 | 40 | 250 | 36.54704 | G, E, P, A |
|  |  | K039112_02 | 40 | 250 | 36.54704 | G, E |
|  |  | K039112_03 | 40 | 250 | 36.54704 | G, E, P, A |
|  |  | K039112_04 | 40 | 250 | 36.54704 | G, E |
|  |  | K039112_05 | 40 | 250 | 36.54704 | G, E |
| *Sinningia concinna* | HC2202-t | HC2202-t_04 | 40 | 250 | 36.54703 | G, E, P, A |
|  |  | HC2202-t_05 | 40 | 250 | 36.54703 | G, E |
|  | K039117 | K039117_01 | 40 | 250 | 36.54703 | G, E |
|  |  | K039117_03 | 40 | 250 | 36.54703 | G, E |
|  |  | K039117_06 | 40 | 250 | 36.54703 | G, E, P, A |
|  | K039118 | K039118_01 | 40 | 250 | 36.54703 | G, E |
|  |  | K039118_02 | 40 | 250 | 36.54703 | G, E |
|  |  | K039118_03 | 40 | 250 | 36.54703 | G, E, P, A |
|  |  | K039118_04 | 40 | 250 | 36.54703 | G, E, P, A |
|  |  | K039118_05 | 40 | 250 | 36.54703 | G, E, P, A |
| *Sinningia elatior* | K039126 | K039126_01 | 49 | 200 | 36.54703 | G, E |
|  |  | K039126_02 | 49 | 200 | 36.54703 | G, E |
|  | K039127 | K039127_01 | 49 | 200 | 36.54703 | G, E, P, A |
|  |  | K039127_02 | 49 | 200 | 36.54703 | G, E, P, A |
|  | K039129 | K039129_01 | 49 | 200 | 36.54703 | G, E, P, A |
|  |  | K039129_02 | 49 | 200 | 36.54703 | G, E |
|  |  | K039129_03 | 49 | 200 | 36.54703 | G, E, P, A |
|  | K039131 | K039131_01 | 49 | 200 | 36.54703 | G, E, P, A |
| *Sinningia harleyi* | HC3403-3 | HC3403-3_10 | 49 | 200 | 36.54703 | G, E, P, A |
|  |  | HC3403-3_16 | 49 | 200 | 36.54703 | G, E |
|  |  | HC3403-3_17 | 49 | 200 | 36.54703 | G, E |
|  | HC3403-8 | HC3403-8_01 | 40 | 250 | 36.54703 | G, E |
|  |  | HC3403-8_08 | 40 | 250 | 36.54703 | G, E |
|  |  | HC3403-8_09 | 40 | 250 | 36.54703 | G, E |
|  |  | HC3403-8_10 | 40 | 250 | 36.54703 | G, E, P, A |
|  |  | HC3403-8_11 | 40 | 250 | 36.54703 | G, E |
|  |  | HC3403-8_12 | 40 | 250 | 36.54703 | G, E, P, A |
|  |  | HC3403-8_13 | 40 | 250 | 36.54703 | G, E |
|  |  | HC3403-8_14 | 40 | 250 | 36.54703 | G, E |
|  | K039135 | K039135_01 | 40 | 250 | 36.54703 | G, E |
|  |  | K039135_02 | 40 | 250 | 36.54703 | G, E, P, A |
|  |  | K039135_03 | 40 | 250 | 36.54703 | G, E |
|  |  | K039135_04 | 40 | 250 | 36.54703 | G, E |
|  |  | K039135_05 | 40 | 250 | 36.54703 | G, E, P, A |
| *Sinningia nordestina* | HC5504-1 | HC5504-1_02 | 40 | 250 | 36.54703 | G, E |
|  |  | HC5504-1_03 | 40 | 250 | 36.54703 | G, E, P, A |
|  |  | HC5504-1_04 | 40 | 250 | 36.54703 | G, E |
|  | HC5504-3 | HC5504-3_03 | 40 | 250 | 36.54704 | G, E, P, A |
|  |  | HC5504-3_04 | 40 | 250 | 36.54704 | G, E |
|  | K039168 | K039168_01 | 40 | 250 | 36.54703 | G, E |
|  |  | K039168_02 | 40 | 250 | 36.54703 | G, E, P, A |
|  |  | K039168_03 | 40 | 250 | 36.54703 | G, E |
|  |  | K039168_04 | 40 | 250 | 36.54703 | G, E, P, A |
|  |  | K039168_05 | 40 | 250 | 36.54703 | G, E, P, A |
| *Sinningia pusilla* | HC5803-2 | HC5803-2_01 | 40 | 250 | 36.54703 | G, E |
|  | HC5803-7 | HC5803-7_09 | 40 | 250 | 36.54703 | G, E, P, A |
|  | K039170 | K039170_01 | 40 | 250 | 36.54703 | G, E, P, A |
|  |  | K039170_02 | 40 | 250 | 36.54703 | G, E |
|  |  | K039170_03 | 40 | 250 | 36.54703 | G, E |
|  |  | K039170_04 | 40 | 250 | 36.54703 | G, E |
|  |  | K039170_05 | 40 | 250 | 36.54703 | G, E, P, A |
|  | K039171 | K039171_01 | 40 | 250 | 36.54703 | G, E, P, A |
|  | K039172 | K039172_01 | 40 | 250 | 36.54703 | G, E, P, A |
| *Sinningia richii* | K039174 | K039174_01 | 49 | 200 | 36.54703 | G, E |
|  |  | K039174_02 | 40 | 250 | 36.54703 | G, E, P, A |
|  |  | K039174_03 | 40 | 250 | 36.54704 | G, E |
|  |  | K039174_04 | 40 | 250 | 36.54704 | G, E, P, A |
|  |  | K039174_05 | 40 | 250 | 36.54704 | G, E, P, A |
|  | K039175 | K039175_01 | 40 | 250 | 36.54704 | G, E, P, A |
|  | K039176 | K039176_01 | 40 | 250 | 36.54704 | G, E |
|  | K039177 | K039177_01 | 40 | 250 | 36.54704 | G, E, P, A |
| *Sinningia sceptrum* | K039178 | K039178_01 | 49 | 200 | 36.54703 | G, E |
|  |  | K039178_02 | 49 | 200 | 36.54703 | G, E, P, A |
|  |  | K039178_03 | 49 | 200 | 36.54703 | G, E |
|  |  | K039178_04 | 49 | 200 | 36.54703 | G, E, P, A |
|  |  | K039178_05 | 49 | 200 | 36.54703 | G, E, P, A |
|  | K039179 | K039179_01 | 49 | 200 | 36.54703 | G, E, P, A |
|  |  | K039179_06 | 49 | 200 | 36.54703 | G, E, P, A |
|  | K039181 | K039181_01 | 49 | 200 | 36.54703 | G, E |
|  |  | K039181_02 | 49 | 200 | 36.54703 | G, E |
| *Sinningia sellovii* | K039184 | K039184_01 | 49 | 200 | 36.54703 | G, E |
|  |  | K039184_02 | 49 | 200 | 36.54703 | G, E |
|  |  | K039184_03 | 49 | 200 | 36.54703 | G, E, P, A |
|  |  | K039184_04 | 49 | 200 | 36.54703 | G, E |
|  |  | K039184_05 | 49 | 200 | 36.54703 | G, E |
|  | K039185 | K039185_02 | 49 | 200 | 36.54703 | G, E |
|  | K039186 | K039186_11 | 49 | 200 | 36.54703 | G, E |
|  |  | K039186_12 | 49 | 200 | 36.54703 | G, E, P, A |
|  |  | K039186_13 | 49 | 200 | 36.54703 | G, E, P, A |
|  |  | K039186_14 | 49 | 200 | 36.54703 | G, E |
|  |  | K039186_15 | 49 | 200 | 36.54703 | G, E, P, A |
|  |  | K039186_16 | 49 | 200 | 36.54703 | G, E, P, A |
| *Sinningia tubiflora* | K039197 | K039197_01 | 49 | 200 | 36.54703 | G, E |
|  |  | K039197_02 | 49 | 200 | 36.54703 | G, E, P, A |
|  | K039198 | K039198_01 | 40 | 250 | 36.54704 | G, E, P, A |
|  |  | K039198_02 | 40 | 250 | 36.54704 | G, E, P, A |
|  | K039199 | K039199_02 | 40 | 250 | 36.54704 | G, E |
|  | K039200 | K039200_01 | 49 | 200 | 36.54703 | G, E, P, A |
|  |  | K039200_02 | 49 | 200 | 36.54703 | G, E |
|  |  | K039200_03 | 49 | 200 | 36.54703 | G, E, P, A |
|  | K039201 | K039201_04 | 40 | 250 | 36.54704 | G, E |
| *Sinningia valsuganensis* | K039203 | K039203_01 | 40 | 250 | 36.54704 | G, E, P, A |
|  |  | K039203_02 | 40 | 250 | 36.54704 | G, E, P, A |
|  |  | K039203_03 | 40 | 250 | 36.54704 | G, E, P, A |
|  |  | K039203_04 | 40 | 250 | 36.54704 | G, E, P, A |
|  |  | K039203_05 | 40 | 250 | 36.54704 | G, E, P, A |
|  | K039204 | K039204_06 | 40 | 250 | 36.54704 | G, E |
| *Sinningia warmingii* | K039205 | K039205_01 | 49 | 200 | 36.54703 | G, E, P, A |
|  |  | K039205_02 | 49 | 200 | 36.54703 | G, E |
|  |  | K039205_03 | 49 | 200 | 36.54703 | G, E, P, A |
|  |  | K039205_04 | 49 | 200 | 36.54704 | G, E |
|  |  | K039205_05 | 40 | 250 | 36.54704 | G, E |
|  | K039209 | K039209_03 | 40 | 250 | 36.54704 | G, E, P, A |
|  |  | K039209_09 | 40 | 250 | 36.54704 | G, E, P, A |
|  |  | K039209_10 | 40 | 250 | 36.54704 | G, E |
|  |  | K039209_11 | 40 | 250 | 36.54704 | G, E |
|  | K039216 | K039216_01 | 49 | 200 | 36.54704 | G, E |
|  |  | K039216_02 | 49 | 200 | 36.54704 | G, E, P, A |
|  |  | K039216_04 | 49 | 200 | 36.54704 | G, E |
|  |  | K039216_05 | 49 | 200 | 36.54704 | G, E |
| * The letter G denotes the analysis of 3D shape and form variation, the letter E denotes the analysis of evolutionary allometry, the letter P denotes the analysis of phylogenetic signal, and the letter A denotes the analysis of ancestral state reconstruction. | | | | | | |
