# Supplementary material for: 3D revelation of phenotypic variation, evolutionary allometry, and ancestral states of corolla shape: a case study of clade Corytholoma (subtribe Ligeriinae, family Gesneriaceae)
Source: Gigascience. 2020 Jan 22;9(1):giz155. doi: 10.1093/gigascience/giz155 (PMC6974915; doi:10.1093/gigascience/giz155)
Supplement: giz155_Supplemental_Figures_and_Tables [file giz155_supplemental_figures_and_tables.zip › Table S3_6.1.docx]

Table S3. The tests of normality and equal variance and the analysis of variance (ANOVA) of centroid size, shape PCs and form PCs.

|  | Shapiro-Wilk test  (test of normality) | | | Bartlett’s test  (test of equal variance) | | Kruskal-Wallis test  (ANOVA) | |
| --- | --- | --- | --- | --- | --- | --- | --- |
|  | Species | *W*-value | *p*-value | *T*-value | *p*-value | *H*-value | *p*-value |
| Centroid size | *Sinningia aggregata* | 0.93 | 3.88×10^–1^ | 97.68 | 1.32×10^–14^ | 147.52 | 2.25×10^–6^ |
|  | *Sinningia allagophylla* | 0.94 | 6.01×10^–1^ |  |  |  |  |
|  | *Sinningia barbata* | 0.89 | 6.80×10^–2^ |  |  |  |  |
|  | *Sinningia carangolensis* | 0.77 | 7.04×10^–3^ |  |  |  |  |
|  | *Sinningia concinna* | 0.69 | 2.24×10^–3^ |  |  |  |  |
|  | *Sinningia elatior* | 0.89 | 1.95×10^–1^ |  |  |  |  |
|  | *Sinningia harleyi* | 0.84 | 1.05×10^–2^ |  |  |  |  |
|  | *Sinningia nordestina* | 0.91 | 2.49×10^–1^ |  |  |  |  |
|  | *Sinningia pusilla* | 0.88 | 1.67×10^–1^ |  |  |  |  |
|  | *Sinningia richii* | 0.89 | 2.41×10^–1^ |  |  |  |  |
|  | *Sinningia sceptrum* | 0.80 | 2.04×10^–2^ |  |  |  |  |
|  | *Sinningia sellovii* | 0.86 | 5.11×10^–2^ |  |  |  |  |
|  | *Sinningia tubiflora* | 0.78 | 1.54×10^–2^ |  |  |  |  |
|  | *Sinningia valsuganensis* | 0.89 | 3.25×10^–1^ |  |  |  |  |
|  | *Sinningia warmingii* | 0.73 | 1.81×10^–3^ |  |  |  |  |
| sPC1 | *Sinningia aggregata* | 0.90 | 1.78×10^–1^ | 65.26 | 1.38×10^–8^ | 146.03 | 4.47×10^–6^ |
|  | *Sinningia allagophylla* | 0.88 | 1.69×10^–1^ |  |  |  |  |
|  | *Sinningia barbata* | 0.94 | 4.64×10^–1^ |  |  |  |  |
|  | *Sinningia carangolensis* | 0.85 | 6.13×10^–2^ |  |  |  |  |
|  | *Sinningia concinna* | 0.77 | 6.58×10^–3^ |  |  |  |  |
|  | *Sinningia elatior* | 0.78 | 2.01×10^–2^ |  |  |  |  |
|  | *Sinningia harleyi* | 0.89 | 4.78×10^–2^ |  |  |  |  |
|  | *Sinningia nordestina* | 0.93 | 4.21×10^–1^ |  |  |  |  |
|  | *Sinningia pusilla* | 0.74 | 4.04×10^–3^ |  |  |  |  |
|  | *Sinningia richii* | 0.84 | 8.26×10^–2^ |  |  |  |  |
|  | *Sinningia sceptrum* | 0.89 | 1.67×10^–1^ |  |  |  |  |
|  | *Sinningia sellovii* | 0.89 | 1.36×10^–1^ |  |  |  |  |
|  | *Sinningia tubiflora* | 0.97 | 8.55×10^–1^ |  |  |  |  |
|  | *Sinningia valsuganensis* | 0.86 | 2.06×10^–1^ |  |  |  |  |
|  | *Sinningia warmingii* | 0.82 | 1.42×10^–2^ |  |  |  |  |
| sPC2 | *Sinningia aggregata* | 0.85 | 3.98×10^–2^ | 63.23 | 3.16×10^–8^ | 140.98 | 4.53×10^–6^ |
|  | *Sinningia allagophylla* | 0.91 | 3.73×10^–1^ |  |  |  |  |
|  | *Sinningia barbata* | 0.93 | 2.96×10^–1^ |  |  |  |  |
|  | *Sinningia carangolensis* | 0.92 | 3.32×10^–1^ |  |  |  |  |
|  | *Sinningia concinna* | 0.94 | 5.15×10^–1^ |  |  |  |  |
|  | *Sinningia elatior* | 0.86 | 1.22×10^–1^ |  |  |  |  |
|  | *Sinningia harleyi* | 0.77 | 1.73×10^–3^ |  |  |  |  |
|  | *Sinningia nordestina* | 0.85 | 5.56×10^–2^ |  |  |  |  |
|  | *Sinningia pusilla* | 0.83 | 4.61×10^–2^ |  |  |  |  |
|  | *Sinningia richii* | 0.92 | 4.31×10^–1^ |  |  |  |  |
|  | *Sinningia sceptrum* | 0.76 | 1.04×10^–2^ |  |  |  |  |
|  | *Sinningia sellovii* | 0.83 | 2.24×10^–2^ |  |  |  |  |
|  | *Sinningia tubiflora* | 0.91 | 3.24×10^–1^ |  |  |  |  |
|  | *Sinningia valsuganensis* | 0.89 | 3.39×10^–1^ |  |  |  |  |
|  | *Sinningia warmingii* | 0.91 | 2.14×10^–1^ |  |  |  |  |
| sPC3 | *Sinningia aggregata* | 0.90 | 1.81×10^–1^ | 81.17 | 1.72×10^–11^ | 143.36 | 1.52×10^–6^ |
|  | *Sinningia allagophylla* | 0.91 | 3.30×10^–1^ |  |  |  |  |
|  | *Sinningia barbata* | 0.95 | 6.36×10^–1^ |  |  |  |  |
|  | *Sinningia carangolensis* | 0.80 | 1.56×10^–2^ |  |  |  |  |
|  | *Sinningia concinna* | 0.91 | 2.41×10^–1^ |  |  |  |  |
|  | *Sinningia elatior* | 0.88 | 1.81×10^–1^ |  |  |  |  |
|  | *Sinningia harleyi* | 0.95 | 4.80×10^–1^ |  |  |  |  |
|  | *Sinningia nordestina* | 0.93 | 4.73×10^–1^ |  |  |  |  |
|  | *Sinningia pusilla* | 0.92 | 3.66×10^–1^ |  |  |  |  |
|  | *Sinningia richii* | 0.89 | 2.49×10^–1^ |  |  |  |  |
|  | *Sinningia sceptrum* | 0.71 | 4.47×10^–3^ |  |  |  |  |
|  | *Sinningia sellovii* | 0.83 | 2.22×10^–2^ |  |  |  |  |
|  | *Sinningia tubiflora* | 0.98 | 9.78×10^–1^ |  |  |  |  |
|  | *Sinningia valsuganensis* | 0.97 | 8.75×10^–1^ |  |  |  |  |
|  | *Sinningia warmingii* | 0.95 | 6.26×10^–1^ |  |  |  |  |
| sPC4 | *Sinningia aggregata* | 0.89 | 1.29×10^–1^ | 82.35 | 1.03×10^–11^ | 135.74 | 4.97×10^–6^ |
|  | *Sinningia allagophylla* | 0.92 | 4.61×10^–1^ |  |  |  |  |
|  | *Sinningia barbata* | 0.93 | 2.65×10^–1^ |  |  |  |  |
|  | *Sinningia carangolensis* | 0.86 | 7.56×10^–2^ |  |  |  |  |
|  | *Sinningia concinna* | 0.81 | 2.09×10^–2^ |  |  |  |  |
|  | *Sinningia elatior* | 0.89 | 2.17×10^–1^ |  |  |  |  |
|  | *Sinningia harleyi* | 0.97 | 7.86×10^–1^ |  |  |  |  |
|  | *Sinningia nordestina* | 0.97 | 9.05×10^–1^ |  |  |  |  |
|  | *Sinningia pusilla* | 0.82 | 3.69×10^–2^ |  |  |  |  |
|  | *Sinningia richii* | 0.91 | 3.66×10^–1^ |  |  |  |  |
|  | *Sinningia sceptrum* | 0.73 | 6.63×10^–3^ |  |  |  |  |
|  | *Sinningia sellovii* | 0.81 | 1.23×10^–2^ |  |  |  |  |
|  | *Sinningia tubiflora* | 0.91 | 2.95×10^–1^ |  |  |  |  |
|  | *Sinningia valsuganensis* | 0.90 | 3.82×10^–1^ |  |  |  |  |
|  | *Sinningia warmingii* | 0.96 | 7.49×10^–1^ |  |  |  |  |
| fPC1 | *Sinningia aggregata* | 0.96 | 6.83×10^–1^ | 111.88 | 2.41×10^–17^ | 145.92 | 4.69×10^–6^ |
|  | *Sinningia allagophylla* | 0.97 | 9.13×10^–1^ |  |  |  |  |
|  | *Sinningia barbata* | 0.88 | 5.03×10^–2^ |  |  |  |  |
|  | *Sinningia carangolensis* | 0.82 | 2.24×10^–2^ |  |  |  |  |
|  | *Sinningia concinna* | 0.75 | 4.02×10^–3^ |  |  |  |  |
|  | *Sinningia elatior* | 0.95 | 6.65×10^–1^ |  |  |  |  |
|  | *Sinningia harleyi* | 0.88 | 3.57×10^–2^ |  |  |  |  |
|  | *Sinningia nordestina* | 0.94 | 5.53×10^–1^ |  |  |  |  |
|  | *Sinningia pusilla* | 0.89 | 1.78×10^–1^ |  |  |  |  |
|  | *Sinningia richii* | 0.85 | 8.77×10^–2^ |  |  |  |  |
|  | *Sinningia sceptrum* | 0.84 | 5.32×10^–2^ |  |  |  |  |
|  | *Sinningia sellovii* | 0.87 | 6.46×10^–2^ |  |  |  |  |
|  | *Sinningia tubiflora* | 0.76 | 1.02×10^–2^ |  |  |  |  |
|  | *Sinningia valsuganensis* | 0.90 | 3.66×10^–1^ |  |  |  |  |
|  | *Sinningia warmingii* | 0.76 | 2.90×10^–3^ |  |  |  |  |
| fPC2 | *Sinningia aggregata* | 0.92 | 3.05×10^–1^ | 82.55 | 9.50×10^–12^ | 147.97 | 1.83×10^–6^ |
|  | *Sinningia allagophylla* | 0.95 | 7.12×10^–1^ |  |  |  |  |
|  | *Sinningia barbata* | 0.95 | 5.39×10^–1^ |  |  |  |  |
|  | *Sinningia carangolensis* | 0.87 | 9.85×10^–2^ |  |  |  |  |
|  | *Sinningia concinna* | 0.78 | 9.35×10^–3^ |  |  |  |  |
|  | *Sinningia elatior* | 0.85 | 9.96×10^–2^ |  |  |  |  |
|  | *Sinningia harleyi* | 0.89 | 6.12×10^–2^ |  |  |  |  |
|  | *Sinningia nordestina* | 0.95 | 6.84×10^–1^ |  |  |  |  |
|  | *Sinningia pusilla* | 0.81 | 2.47×10^–2^ |  |  |  |  |
|  | *Sinningia richii* | 0.94 | 6.28×10^–1^ |  |  |  |  |
|  | *Sinningia sceptrum* | 0.79 | 1.73×10^–2^ |  |  |  |  |
|  | *Sinningia sellovii* | 0.88 | 9.51×10^–2^ |  |  |  |  |
|  | *Sinningia tubiflora* | 0.95 | 6.97×10^–1^ |  |  |  |  |
|  | *Sinningia valsuganensis* | 0.81 | 6.87×10^–2^ |  |  |  |  |
|  | *Sinningia warmingii* | 0.75 | 2.50×10^–3^ |  |  |  |  |
| fPC3 | *Sinningia aggregata* | 0.86 | 5.17×10^–2^ | 109.30 | 7.65×10^–17^ | 142.71 | 2.05×10^–6^ |
|  | *Sinningia allagophylla* | 0.94 | 6.32×10^–1^ |  |  |  |  |
|  | *Sinningia barbata* | 0.91 | 1.79×10^–1^ |  |  |  |  |
|  | *Sinningia carangolensis* | 0.88 | 1.40×10^–1^ |  |  |  |  |
|  | *Sinningia concinna* | 0.93 | 4.04×10^–1^ |  |  |  |  |
|  | *Sinningia elatior* | 0.84 | 6.71×10^–2^ |  |  |  |  |
|  | *Sinningia harleyi* | 0.67 | 1.99×10^–4^ |  |  |  |  |
|  | *Sinningia nordestina* | 0.87 | 1.06×10^–1^ |  |  |  |  |
|  | *Sinningia pusilla* | 0.90 | 2.38×10^–1^ |  |  |  |  |
|  | *Sinningia richii* | 0.93 | 4.95×10^–1^ |  |  |  |  |
|  | *Sinningia sceptrum* | 0.77 | 1.04×10^–2^ |  |  |  |  |
|  | *Sinningia sellovii* | 0.95 | 7.05×10^–1^ |  |  |  |  |
|  | *Sinningia tubiflora* | 0.93 | 4.40×10^–1^ |  |  |  |  |
|  | *Sinningia valsuganensis* | 0.93 | 6.09×10^–1^ |  |  |  |  |
|  | *Sinningia warmingii* | 0.92 | 2.18×10^–1^ |  |  |  |  |
| fPC4 | *Sinningia aggregata* | 0.83 | 2.92×10^–2^ | 110.40 | 4.67×10^–17^ | 145.63 | 5.37×10^–6^ |
|  | *Sinningia allagophylla* | 0.98 | 9.74×10^–1^ |  |  |  |  |
|  | *Sinningia barbata* | 0.87 | 3.85×10^–2^ |  |  |  |  |
|  | *Sinningia carangolensis* | 0.79 | 1.07×10^–2^ |  |  |  |  |
|  | *Sinningia concinna* | 0.83 | 3.10×10^–2^ |  |  |  |  |
|  | *Sinningia elatior* | 0.82 | 5.10×10^–2^ |  |  |  |  |
|  | *Sinningia harleyi* | 0.89 | 5.83×10^–2^ |  |  |  |  |
|  | *Sinningia nordestina* | 0.97 | 8.55×10^–1^ |  |  |  |  |
|  | *Sinningia pusilla* | 0.90 | 2.25×10^–1^ |  |  |  |  |
|  | *Sinningia richii* | 0.87 | 1.31×10^–1^ |  |  |  |  |
|  | *Sinningia sceptrum* | 0.97 | 9.33×10^–1^ |  |  |  |  |
|  | *Sinningia sellovii* | 0.91 | 1.88×10^–1^ |  |  |  |  |
|  | *Sinningia tubiflora* | 0.96 | 7.86×10^–1^ |  |  |  |  |
|  | *Sinningia valsuganensis* | 0.95 | 7.78×10^–1^ |  |  |  |  |
|  | *Sinningia warmingii* | 0.83 | 1.66×10^–2^ |  |  |  |  |
|  | | | | | | | |
