# Supplementary material for: 3D revelation of phenotypic variation, evolutionary allometry, and ancestral states of corolla shape: a case study of clade Corytholoma (subtribe Ligeriinae, family Gesneriaceae)
Source: Gigascience. 2020 Jan 22;9(1):giz155. doi: 10.1093/gigascience/giz155 (PMC6974915; doi:10.1093/gigascience/giz155)
Supplement: giz155_Supplemental_Figures_and_Tables [file giz155_supplemental_figures_and_tables.zip › Table S4_6.1.docx]

Table S3. Centroid size and four morphological traits of extant species and ancestral states.

|  | **Centroid size**  **(10^4^)** | **Traits (mean ± standard deviation)** | | | |
| --- | --- | --- | --- | --- | --- |
|  |  | **Tube curvature**  **(10^-4^)** | **Lobe area ratio**  **(10^-2^)** | **Tube dilation**  **(10^-1^)** | **Lobe recurvation**  **(10^2^)** |
| **Extant species** |  |  |  |  |  |
| *S. aggregata* | 10.34 ± 0.29 | −1.77 ± 0.99 | 7.59 ± 0.56 | 8.99 ± 0.32 | 1.70 ± 0.29 |
| *S. allagophylla* | 6.71 ± 0.35 | −0.42 ± 0.82 | 14.93 ± 2.56 | 17.12 ± 0.73 | 2.93 ± 0.41 |
| *S. barbata* | 16.73 ± 1.01 | 14.26 ± 1.73 | 3.97 ± 0.24 | 16.01 ± 0.31 | 5.80 ± 0.25 |
| *S. carangolensis* | 12.71 ± 0.39 | −1.48 ± 0.21 | 7.59 ± 0.32 | 8.87 ± 0.40 | 1.61 ± 0.32 |
| *S. concinna* | 6.93 ± 0.35 | 8.51 ± 2.60 | 14.90 ± 0.86 | 9.79 ± 0.21 | 3.06 ± 0.22 |
| *S. elatior* | 12.54 ± 0.50 | −3.88 ± 1.51 | 6.33 ± 0.92 | 10.66 ± 1.20 | 4.26 ± 0.54 |
| *S. harleyi* | 15.75 ± 0.20 | −1.99 ± 0.50 | 8.87 ± 0.20 | 11.39 ± 0.28 | 6.11 ± 0.45 |
| *S. nordestina* | 8.52 ± 0.10 | −9.57 ± 0.28 | 12.89 ± 0.42 | 12.76 ± 0.53 | 1.90 ± 0.08 |
| *S. pusilla* | 4.26 ± 0.30 | 7.10 ± 2.29 | 13.56 ± 1.30 | 10.08 ± 0.67 | 3.41 ± 0.23 |
| *S. richii* | 12.25 ± 0.75 | 6.30 ± 0.84 | 10.61 ± 0.81 | 18.61 ± 1.05 | 3.37 ± 0.77 |
| *S. sceptrum* | 14.91 ± 0.13 | −1.37 ± 0.22 | 6.87 ± 0.05 | 8.76 ± 0.06 | 3.26 ± 0.21 |
| *S. sellovii* | 9.04 ± 0.14 | 0.70 ± 0.96 | 8.10 ± 0.76 | 16.71 ± 1.55 | 1.80 ± 0.29 |
| *S. tubiflora* | 26.70 ± 1.19 | 2.43 ± 0.27 | 9.42 ± 0.66 | 6.06 ± 0.14 | 5.00 ± 1.01 |
| *S. valsuganensis* | 16.03 ± 0.25 | −2.20 ± 0.25 | 6.14 ± 0.42 | 7.40 ± 0.20 | 4.20 ± 0.10 |
| *S. warmingii* | 14.53 ± 0.24 | 0.14 ± 0.45 | 5.17 ± 0.27 | 5.88 ± 0.07 | 1.80 ± 0.19 |
| **Ancestral states** |  |  |  |  |  |
| Node 1 | 11.94 ± 0.11 | 6.18 ± 0.24 | 7.86 ± 0.18 | 15.45 ± 0.15 | 3.17 ± 0.21 |
| Node 2 | 11.91 ± 0.10 | 6.15 ± 0.26 | 7.60 ± 0.15 | 15.08 ± 0.12 | 3.16 ± 0.17 |
| Node 3 | 11.93 ± 0.10 | 5.64 ± 0.29 | 7.36 ± 0.13 | 15.05 ± 0.11 | 3.10 ± 0.15 |
| Node 4 | 11.47 ± 0.08 | 5.00 ± 0.23 | 7.51 ± 0.10 | 14.25 ± 0.10 | 2.72 ± 0.15 |
| Node 5 | 11.36 ± 0.06 | 2.97 ± 0.18 | 7.71 ± 0.08 | 13.32 ± 0.09 | 2.33 ± 0.12 |
| Node 6 | 11.80 ± 0.05 | 1.22 ± 0.13 | 7.28 ± 0.07 | 11.97 ± 0.08 | 2.15 ± 0.09 |
| Node 7 | 12.11 ± 0.05 | 1.15 ± 0.13 | 7.09 ± 0.08 | 11.75 ± 0.08 | 2.02 ± 0.05 |
| Node 8 | 10.80 ± 0.03 | 1.06 ± 0.11 | 7.21 ± 0.13 | 11.52 ± 0.22 | 1.78 ± 0.12 |
| Node 9 | 11.38 ± 0.05 | 1.39 ± 0.11 | 6.91 ± 0.12 | 9.88 ± 0.11 | 2.10 ± 0.11 |
| Node 10 | 12.15 ± 0.06 | 1.46 ± 0.12 | 6.38 ± .013 | 9.03 ± 0.08 | 2.12 ± 0.13 |
| Node 11 | 15.34 ± 0.11 | 1.96 ± 0.11 | 4.96 ± 0.14 | 7.16 ± 0.04 | 2.59 ± 0.23 |
| Node 12 | 11.69 ± 0.06 | 2.49 ± 0.19 | 8.10 ± 0.16 | 13.16 ± 0.08 | 3.90 ± 0.10 |
| Node 13 | 13.07 ± 0.07 | −1.71 ± 0.26 | 6.50 ± 0.14 | 10.18 ± 0.19 | 3.21 ± 0.10 |
| Node 14 | 13.23 ± 0.08 | −1.29 ± 0.06 | 6.80 ± 0.12 | 8.98 ± 0.09 | 2.39 ± 0.07 |
