# Supplementary material for: 3D revelation of phenotypic variation, evolutionary allometry, and ancestral states of corolla shape: a case study of clade Corytholoma (subtribe Ligeriinae, family Gesneriaceae)
Source: Gigascience. 2020 Jan 22;9(1):giz155. doi: 10.1093/gigascience/giz155 (PMC6974915; doi:10.1093/gigascience/giz155)
Supplement: giz155_Supplemental_Figures_and_Tables [file giz155_supplemental_figures_and_tables.zip › Table S5_6.1.docx]

Table S4. Species list and GenBank numbers.

| **Species** | ***trnS-trnG*** | ***ncpGS*** | ***rpl16*** | ***atpB-rbcL*** | ***trnL-trnF*** | ***trnT-trnL*** |
| --- | --- | --- | --- | --- | --- | --- |
| *S. aggregata* | AJ438364 | AJ459619 | AJ487715 | AJ439913 | AJ439757 | AJ439262 |
| *S. allagophylla* | AJ438407 | AJ459663 | AJ487758 | AJ439956 | AJ439801 | AJ439306 |
| *S. barbata* | AJ438386 | AJ459642 | AJ487738 | AJ439936 | AJ439780 | AJ439285 |
| *S. carangolensis* | AJ438391 | AJ459647 | AJ487743 | AJ439940 | AJ439785 | AJ439290 |
| *S. concinna* | AJ438393 | AJ459649 | AJ487745 | AJ439942 | AJ439787 | AJ439292 |
| *S. elatior* | AJ438398 | AJ459654 | AJ487749 | AJ439947 | AJ439792 | AJ439297 |
| *S. harleyi* | AJ438392 | AJ459648 | AJ487744 | AJ439941 | AJ439786 | AJ439291 |
| *S. nordestina* | AJ438387 | AJ459643 | AJ487739 | AJ439937 | AJ439781 | AJ439286 |
| *S. pusilla* | AJ438410 | AJ459666 | AJ487761 | AJ439959 | AJ439804 | AJ439309 |
| *S. richii* | AJ438403 | AJ459659 | AJ487754 | AJ439952 | AJ439797 | AJ439302 |
| *S. sceptrum* | AJ438399 | AJ459655 | AJ487750 | AJ439948 | AJ439793 | AJ439298 |
| *S. sellovii* | AJ438383 | AJ459639 | AJ487735 | AJ439933 | AJ439777 | AJ439282 |
| *S. tubiflora* | AJ438380 | AJ459636 | AJ487732 | AJ439930 | AJ439774 | AJ439279 |
| *S. valsuganensis* | AJ438401 | AJ459657 | AJ487752 | AJ439950 | AJ439795 | AJ439300 |
| *S. warmingii* | AJ438372 | AJ459627 | AJ487723 | AJ439921 | AJ439765 | AJ439270 |
