# Supplementary figures and images for: 3D revelation of phenotypic variation, evolutionary allometry, and ancestral states of corolla shape: a case study of clade Corytholoma (subtribe Ligeriinae, family Gesneriaceae)
Source: Gigascience. 2020 Jan 22;9(1):giz155. doi: 10.1093/gigascience/giz155 (PMC6974915; doi:10.1093/gigascience/giz155)

**A**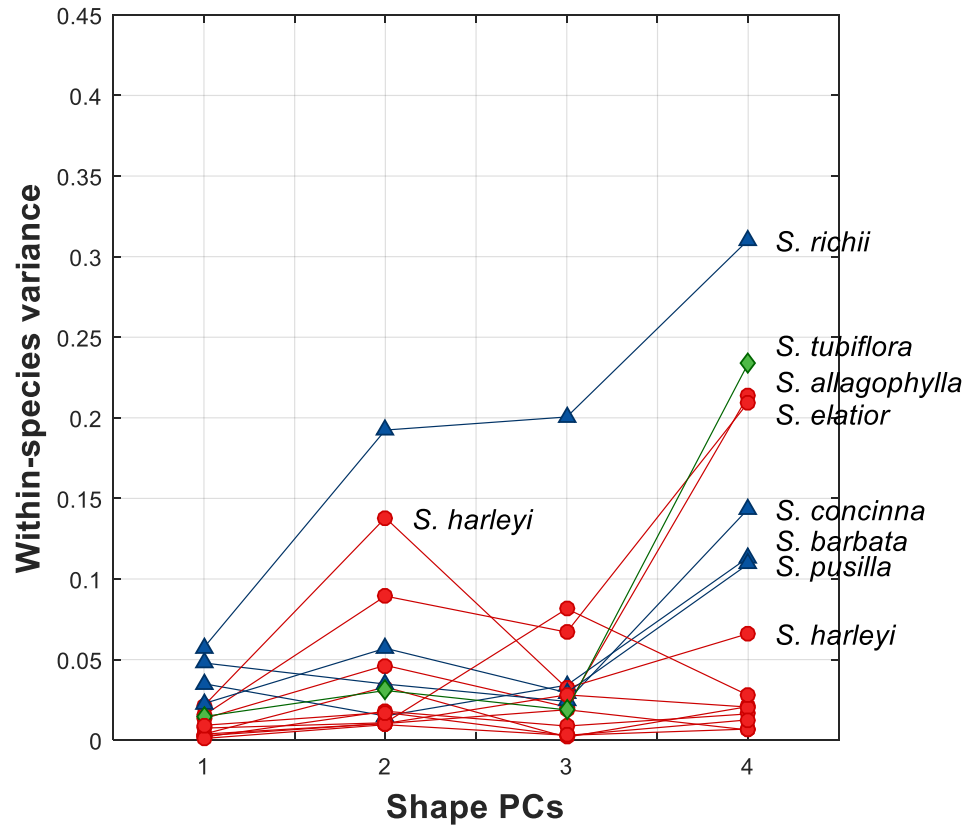**B**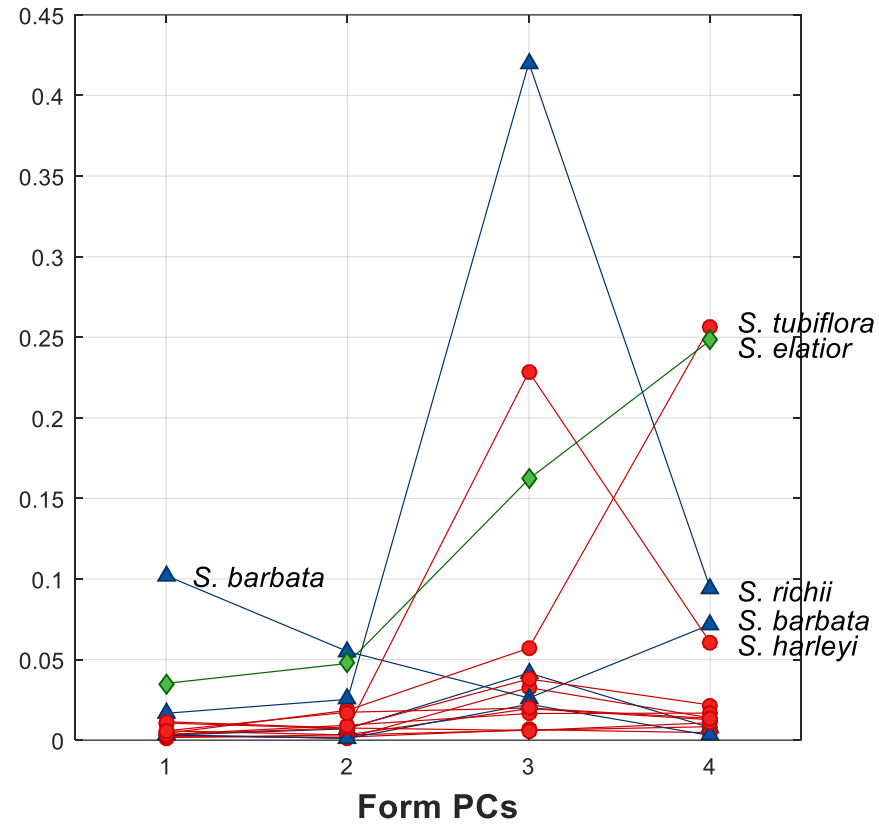

Supplement: giz155_Supplemental_Figures_and_Tables [file giz155_supplemental_figures_and_tables.zip › Fig.S1_6.2.pdf]

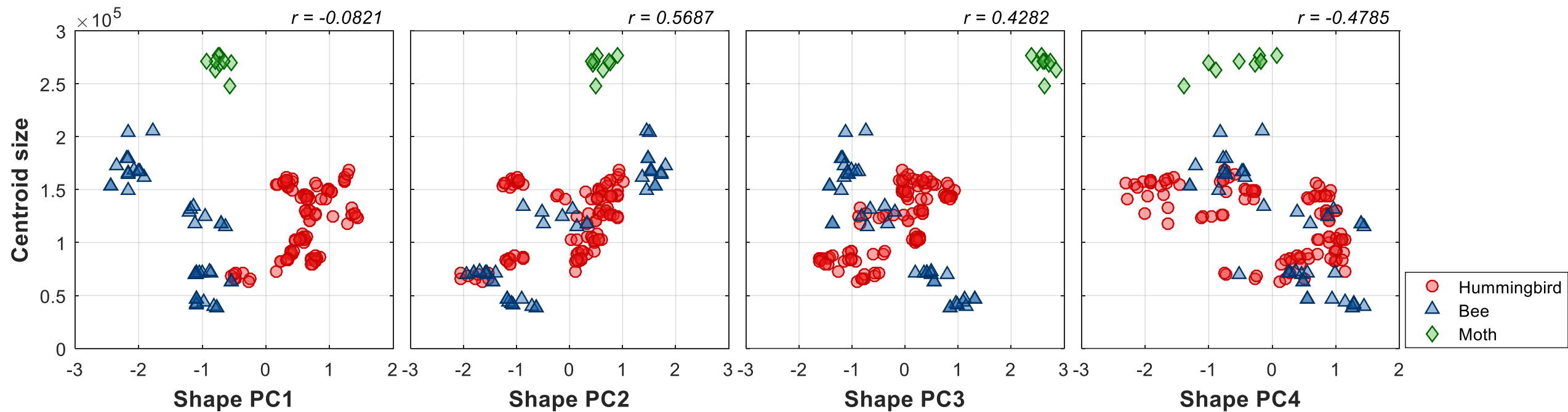

Supplement: giz155_Supplemental_Figures_and_Tables [file giz155_supplemental_figures_and_tables.zip › Fig.S2_6.2.pdf]
