# Supplementary material for: 3D revelation of phenotypic variation, evolutionary allometry, and ancestral states of corolla shape: a case study of clade Corytholoma (subtribe Ligeriinae, family Gesneriaceae)
Source: Gigascience. 2020 Jan 22;9(1):giz155. doi: 10.1093/gigascience/giz155 (PMC6974915; doi:10.1093/gigascience/giz155)
Supplement: giz155_Response_to_Reviewer_Comments_Original_Submission [file giz155_response_to_reviewer_comments_original_submission.pdf]

Author's Response To Reviewer Comments

Close

###Please find the "Person cover (response to reviewer)" for the complete response (with figures)###

Reviewer #1
In their manuscript "3D revelation of phenotypic variation, evolutionary allometry, and ancestral states of corolla shape: a case study of clade Corytholoma (subtribe Ligeriinae, family Gesneriaceae)", Hsu et al. present a thorough morphometric analysis of corolla shape. I very much enjoyed reading this manuscript. It integrates 2D and 3D geometric morphometrics with evolutionary theory in an unprecedented way using plant organs as an example. The morphometric analyses and the specimens are beautiful. The availability of 2D slice images, volumetric images, and surface images in the GigaScience Database repository is extremely valuable for the reproducibility of morphometric studies.

I only have minor suggestions to improve readability.
... Author: Many thanks for this very positive review.

The reader is often referred to references [8] and [11] for critical methods information. This includes: image processing information (page 10, lines 181-185), landmark selection procedure (page 28, lines 469-470), and visualization (page 29, line 505). While of course all the information doesn't need to be repeated here, it should be summarized, so that the reader can understand the gist of what is being done without having to refer back to these publications.

... Author: Thanks for the comment. The manuscript was revised accordingly. The image processing information was updated in Lines 166-179 of the revised manuscript. The landmark selection procedure was updated in Lines 188-211 of the revised manuscript. The visualization was updated in Lines 508-509 of the revised manuscript.

Can a full explanation of the primary landmarks be provided? Figure 13 helps, but it would be good to see the full 25 primary landmarks. It would also help to see a visualization of all secondary landmarks too, just so the reader has a visual understanding of what the data is based upon. It might help to do this for two morphologically distinct species. For those not familiar with corolla morphology in these species, a botanical/morphological/anatomical description of what the primary landmarks correspond to would help understanding.

... Author: Thanks for the insightful comment. The Fig. 3 of the revised manuscript was updated accordingly (Line 212). In the Fig. 3, two species with distinct corolla morphology were provided. The details corresponding to the corolla morphology, petal position, homologous features were provided in the revised manuscript (Lines 188-203).

Scaling is used for shape but not for form. Why?

... Author: Thanks for the comment. Please let us clarify further. Form variation is defined as the variation of shape and size together, and the scale (i.e., distances) between landmarks present the size. To retain form information in the landmarks, scaling cannot apply to the landmarks. Thus, when size is considered as a critical factor (e.g., [1, 2]), partial-GPA is used to investigate the form variation.

... [1] Klingenberg CP. Evolution and development of shape: integrating quantitative approaches. Nat Rev Genet. 2010;11(9):623. doi:10.1038/nrg2829
... [2] Klingenberg CP. Size, shape, and form: concepts of allometry in geometric morphometrics. Dev Genes Evol. 2016;226(3):113-137. doi:10.1007/s00427-016-0539-2

Also, I know "shape" and "form" have strict meanings in the morphometric community. But upon first usage, it might be good to just add "2D" and "3D" for clarification, not only for those not familiar with morphometrics, but also in some non-English languages the use of "shape" and "form" is confusing.

... Author: Thanks for the comment. Please let us clarify further. In this study, the shape and form analyses were both carried out in 3D using 3D landmarks. We also added “3D” before shape and form throughout the revised manuscript (highlighted in green) and hoping that this will help to clarify the use of shape and form.

The methods come after the results section. But at least for the landmarks, an explanation up front might be helpful for readers to understand the analysis and interpret it.

... Author: Thanks for the insightful comment. The manuscript was updated accordingly (Lines 187-220 of the revised manuscript).

Reviewer #2
In the manuscript "3D revelation of phenotypic variation, evolutionary allometry, and ancestral states of corolla shape: a case study of clade Corytholoma (subtribe Ligeriinae, family Gesneriaceae)", Hsu et al. applied 3D X-ray microcomputed tomography and landmark-based geometric morphometrics to study the corolla shape variation, evolutionary allometry, and some phylogenetic analysis. The manuscript is well written. The details are explained very clear. For their data, the analysis has been performed thoroughly.

... Author: Many thanks for this very positive review.

The main concern I have is the sample size. In this work, the authors studied 15 species. However, each species only has one or two plant individuals and also were collected from different years. Although five samples from this single or two plants were scanned, some of the analysis and statements cannot convince me. In my opinion, if this is a method or breakthrough technology paper, using small sample size to test and valid the method might be fine. But this manuscript is not a method paper because all the methods are existing and commonly used. For the work focusing on the biology, I would evaluate the result which should be supported by solid analysis with enough sample size. In this work, I was wondering whether this single (or two) individual plant are representative. Assume so, then I think the analysis for the major variation using PCA and the phylogenetic analysis based on the mean shape might be fine. But the result related the within-species variation cannot convince me. Can the variance in this particular plant represent the variance for the entire species? I don't think so. The variation basically is the within-plant variation. Thus the analysis and result such as Fig. 3A, Fig. 4B, 5B are supported well. In addition, Fig 3 and Fig 4B don't have the statistic.

... Author: Thanks for the insightful comment.

... In short, the specimen number increases from 75 to 153 in the revised manuscript. Please allow us to explain the reason of including only 75 specimens in the original manuscript below.

... We agree that a large sample size is always a good practice for analyzing the shape variations. However, 3D CT scanning is costly and time-consuming. The specimen used for 3D scanning was preselected according to corolla shape variation analysis using 2D side views (Fig. R1 below). In the past years, we only have collected a total of 153 corolla specimens with uneven specimen numbers for each species. To assess the within-species variation, all the 153 specimens were included in the analyses of 3D shape and form variation and of evolutionary allometry. To avoid the results may be dominated by the species with larger sample size or the species with larger morphological variation, five specimens of each species selected based on shape score were used in the analyses of phylogenetic signal and of ancestral state reconstruction (Lines 147-152 of the revised manuscript). The Table 1, 2, Fig. 4, 5B, 6B, 7, 8, 9, S1, and S2 of the revised manuscript were updated.

... (Please find the "Person cover [response to reviewer]" for the figure)
... Figure R1. Illustration of 2D side-view corolla shape variation analysis. The left top was the 2D side-view image, the left bottom was the selected landmark in the image, and the right was the scatter plot of PC scores obtained from GM analysis using 2D side-view landmarks.

... Per the reviewer’s suggestion, we include all the 153 specimens in the revised manuscript. Each species has at least two plant individuals. The updates are reflected in Lines 135-137 and 139-141 of the revised manuscript.

... The specimen for the same species was collected in the same flowering season (Lines 141-142 of the revised manuscript). Each species has its own habits; the flowering time of most species was not

synchronized.

... In Fig. 4 (Line 229), 5B (Line 256), and 6B (Line 285) of the revised manuscript, the multiple comparison tests for centroid size and PCs were provided. We hope that this will help to present the within-species variation.

Another point is about the landmark based method. First, in this work, 25 primary landmarks are selected manually first. Then the rest 390 secondary landmarks are derived computationally, but still highly rely on the manual landmarks. Thus this landmark dataset may also introduce error, and can be subjective and labor intensive. 3D X-ray images also has variance for each scan. This has the similar problem with other method such as the author mentioned for other work: (line 89) "2D images are usually acquired manually, which may introduce error or artifacts…"; (line 97) " distance-based traits are typically proposed based on manual observation and can be subjective"; (line 126) " the traits were proposed statistically rather than manually". Second, the landmark based method has been widely applied. However, it has a main limitation that you have to make sure the landmarks are homologous. I was wonder whether all the corollas have five petals. If not, how the authors made sure the landmark were homologous? Because of this limitation, similar work cannot be easily expanded to a wider range of species. It would be better to discuss a bit in the discussion.

... Author: Thanks for the insightful comment. We revised the paragraphs of 3D flower image data (Lines 166-179), landmark identification (Line 188-211) and updated the Fig. 2 (Line 181) and 3 (Fig. 212) of the revised manuscript.

... We agree that manual landmark selection may introduce error, is subjective, and is labor-intensive. CT images are at a very high resolution (36.547 micron). The details of the corollas are clearly visible. Although all the primary landmarks and the tube-tube rims were selected manually using a software Landmark, these anatomical features are obvious and can easily be identified (Fig. 3 of the revised manuscript). Thus, the error introduced by manual selection can be minimized. The secondary landmarks were automatically selected using a software developed by our team [1]. Thus, the selection of the secondary landmarks can be objective.

... The 3D imaging of each specimen was calibrated using a bar phantom (for calibrating the spatial resolution and the X-ray quality, Lines 167-168 of the revised manuscript). The scan parameters like source voltage, source current, and scan resolution were than determined (Table S2 of the revised manuscript).

... In our humble opinion, the 3D approach can overcome the “subjective” issues using 2D imaging. For example, the angle of capturing the image of the object is largely controlled by the operator. This is no doubt that the process is subjective and varies from one operator to another. The error and the so-called artifacts are introduced to the analysis.

... We also agree that it is critical to use homologous landmarks in landmark-based GM. Gesneriaceae is a family in the order Lamiales. In plant systematics, the Lamiales flower is gamopetalous (has lobe part and tube part) and consist of two dorsal petals, two lateral patels, and one ventral petal. The petal position (or the petal identity) can be identified according to the dorsal staminoid (the Lamiales flowers are alternipetalous and the dorsal stamen is underdeveloped and infertile). Thus, homologous anatomical features can be easily identified. The selected landmarks are also homologous.

... [1] Wang YH, Hsu HC, Chou WC, Kuo YF. Automatically Identifying floral contours and vascular bundles in 3D images. In 2018 ASABE Annual International Meeting, 2018. (p. 1). American Society of Agricultural and Biological Engineers.

Some minor points:
a) At the beginning, I was confused about the "shape" and "form". It got clear after reading several pages. It would be nice to clarify a bit at the beginning.

... Author: Thanks for the comment. We annotated the form refers to shape and size together at the beginning of the Background (Lines 51 of the revised manuscript).

b) (Line 78) Although this table is a very nice literature summary, I feel it is not directly related to this study. The authors could consider to move it to supplement.

... Author: Thanks for the comment. The table was moved to supplement in the revised manuscript (Line 770).

c) (Line 141) I cannot find Appendix 1.

... Author: The irrelevant text was remove in the revised manuscript.

d) (Line 167) From this summary, it looks like the color has strong correlation with the pollination types. If the authors have the 2D images as well, additional analysis to associate color and pollination types can be done.

... Author: Thanks for the insightful comment. Indeed, the previous study, Perret et al. (2003), indicated that, apart from the corolla shape, the corolla color would be one of the important factor corresponding to the pollination type. In this study, we focus on the variation of corolla shape and aim to identify its shape trait in 3D. We removed the color information in the revised manuscript.

... We have other in-progress research related to flower color and color pattern on these species (Fig. R2). The preliminary results showed that the corolla color and color pattern are also complex and their association with pollination type is not straightforward and not intuitive (Fig. R3). We would like to publish the finding on the corolla color as an independent research article.

... (Please find the "Person cover [response to reviewer]" for the figure)
... Figure R2. Foreground-background separation for ventral petals. The upper panel is the original image. The bottom panel is the foreground-background separated mask, the white region is foreground, and the black region is background.

... (Please find the "Person cover [response to reviewer]" for the figure)
... Figure R3. (a) Foreground color, (b) background color, and (c) contrast of the variegated species. Red: hummingbird-pollinated species; Blue: Bee-pollinated species.

e) (Line 200) No indication of "the average" in the figure.

... Author: The Fig. 4 were updated in the revised manuscript (Lines 225 and 229).

f) (Line 204) Fig 3B was not mentioned anywhere in the manuscript.

... Author: The figure was removed accordingly (Fig. 4 of the revised manuscript, Line 229).

g) (Line 274) I know "shape scores" will be explained in the method section later, but it appeared too suddenly here. Please either explain a bit here or guide readers to the method section.

... Author: Thanks for the comment. The manuscript was revised accordingly (Line 315-316 of the revised manuscript).

h) (Line 276), I know p=0.0625 is closed to 0.05, but instead of "significant", people normally say it is not statistical significant

... Author: Thanks for the insightful comment. Per your comment on the sample size, we increased the number of specimen from 75 to 153 and re-analyzed the evolutionary allometry. The p = 0.0031 in the revised manuscript (Lines 304-306).

i) (Line 305) the p value is only for the tube curvature.

... Author: Thanks for the comment. The manuscript was revised accordingly (Line 335-336 of the revised manuscript).

j) (Line 310) Cannot see the last column of the Table 3.

... Author: The column size was adjusted (Line 339 of the revised manuscript). The full table 2 was also submitted separately of the file inventory for this manuscript.

k) (Line 350) How to explain the difference of pollination types at nodes 10 and 12 between shape and form?

... Author: Thanks for the insightful comment. Due to the analyses of shape and form were based on the sPCs and fPCs, the difference between two PCs was expected to be the size variation. Thus, the intuitive explanation for the difference of pollination types at nodes 10 and 12 between shape and form would be the size difference (Line 380-381 of the revised manuscript).

l) In the method section, authors used different number of permutations (e.g. 10,000 times, 1000 times, 100 times) for different methods. I just wonder how authors picked these numbers.

... Author: The reshuffle times are increased to 10,000 to make p-value having four decimal places of precision for the three permutation tests in the revised manuscript. The relevant information was updated in the revised manuscript (Line 522, 569, and 599).

Close
