# Supplementary material for: 3D revelation of phenotypic variation, evolutionary allometry, and ancestral states of corolla shape: a case study of clade Corytholoma (subtribe Ligeriinae, family Gesneriaceae)
Source: Gigascience. 2020 Jan 22;9(1):giz155. doi: 10.1093/gigascience/giz155 (PMC6974915; doi:10.1093/gigascience/giz155)
Supplement: giz155_Response_to_Reviewer_Comments_Revision_1 [file giz155_response_to_reviewer_comments_revision_1.pdf]

Author's Response To Reviewer Comments

Close

###Please find the "Person cover (response to reviewer)" for the complete response###
Reviewer #2
Your manuscript "3D revelation of phenotypic variation, evolutionary allometry, and ancestral states of corolla shape: a case study of clade Corytholoma (subtribe Ligeriinae, family Gesneriaceae)" (GIGA-D-19-00247R1) has been assessed by our reviewers. Based on these reports, and my own assessment as Editor, I am pleased to inform you that it is potentially acceptable for publication in GigaScience, once you have carried out some essential revisions suggested by our reviewers.
...Author: Many thanks for this very positive review.

1) The authors performed "Scheffe's multiple comparison tests" (line 231) for the statistics. But this test should be run only if the null hypothesis is rejected in an ANOVA test. But to perform ANOVA, the ANOVA assumption tests have to perfomed to show those assumptions are satisfied. Therefore, authors have to perform test to verify the ANOVA assumption first. If the assumptions are satisfied, they then perform ANOVA. After that, if they got significant F-statistic, then perform Scheffe's test.

...Author: Thanks for the insightful comment. The ANOVA assumption test and the ANOVA were provided in Table S3 of the revised manuscript (Line 829). The F-statistic (H-values of Kruskal-Wallis test) were significant in centroid size, four sPCs and four fPCs.

2) "p=0" (line 335 and Table2) usually means p value is lower than the default minimum number of the program they used. I would suggest authors to find what the minimum number of their program is and write something like "p
...Author: Thanks for the comment. The minimum numbers were provided in Table 2 of the revised manuscript (Line 337-338, and 341).

Editor
Your manuscript "3D revelation of phenotypic variation, evolutionary allometry, and ancestral states of corolla shape: a case study of clade Corytholoma (subtribe Ligeriinae, family Gesneriaceae)" (GIGA-D-19-00247R1) has been assessed by our reviewers. Based on these reports, and my own assessment as Editor, I am pleased to inform you that it is potentially acceptable for publication in GigaScience, once you have carried out some essential revisions suggested by our reviewers.

...Author: Many thanks for this very positive review.

Reviewer #2 requests that you run ANOVA and ANOVA assumption tests to show that your assumptions are satisfied.
...Author: Thanks for the comment. The manuscript was revised accordingly.

Furthermore we require some additional formatting revisions with the addition of the following missing sections in the paper, to match our Research article format:
1) Before the Methods section, please add a section on "Potential Implications".
Potential implications
Authors should provide some additional comments about potential, more broad-ranging implications of their work that are not directly related to the current focus of their manuscript. This section is meant to promote discussion on possible ways the findings or data presented might be used in or have a relationship with other areas of research that may not be directly apparent in the work. It is not meant to provide ‘proof of importance’ of the work. Only to engender expansion of use to other areas.

Explicit personal opinions by the authors are permitted, but they should be made clear as such. References or related information to support the propositions should be included. These section should focus on work that can be done within the foreseeable future and specifically using the information

within the manuscript, not provide speculation on how it will relate to far-reaching goals of the research area.

...Author: Thanks for the comment. The section of “Potential Implications” was added in the revised manuscript (Line 499).

2) After the Methods section, please add a section on "Availability of Supporting Data":
Availability of supporting data and materials
GigaScience requires authors to deposit the data set(s) supporting the results reported in submitted manuscripts in a publicly-accessible data repository such as GigaDB (see GigaDB database terms of use for complete details). This section should be included when supporting data are available and must include the name of the repository and the permanent identifier or accession number and persistent hyperlinks for the data sets (if appropriate). The following format is recommended:

"The data set(s) supporting the results of this article is(are) available in the [repository name] repository, [cite unique persistent identifier]."

...Author: Thanks for the comment. The section of “Availability of Supporting Data” was added in the revised manuscript (Line 647).

3) Declarations:
3a) Abbreviations - please list all abbreviations used more than once in the main text here in alphabetical order.

3b) Competing Interests - please state any competing interests or write "The authors declare that they have no competing interests"

3c) After Funding section - please add "Authors' Contributions"
The individual contributions of authors to the manuscript should be specified in this section. Guidance and criteria for authorship can be found in our editorial policies. We would recommend you follow some kind of standardised taxonomy like the CASRAI CRediT (Contributor Roles Taxonomy).

...Author: Thanks for the comment. The section of “Abbreviations”, “Competing Interests”, and “Authors' Contributions” were added in the revised manuscript (Line 654, 661, and 668, respectively).

Close
