# Supplementary material for: 3D revelation of phenotypic variation, evolutionary allometry, and ancestral states of corolla shape: a case study of clade Corytholoma (subtribe Ligeriinae, family Gesneriaceae)
Source: Gigascience. 2020 Jan 22;9(1):giz155. doi: 10.1093/gigascience/giz155 (PMC6974915; doi:10.1093/gigascience/giz155)
Supplement: giz155_Reviewer_1_Report_Original_Submission — Daniel H Chitwood -- 7/21/2019 Reviewed [file giz155_reviewer_1_report_original_submission.pdf]

Reviewer Report

Title: 3D revelation of phenotypic variation, evolutionary allometry, and ancestral states of corolla shape: a case study of clade Corytholoma (subtribe Ligeriinae, family Gesneriaceae)

Version: Original Submission **Date:** 7/21/2019

Reviewer name: Daniel H Chitwood

Reviewer Comments to Author:

In their manuscript "3D revelation of phenotypic variation, evolutionary allometry, and ancestral states of corolla shape: a case study of clade Corytholoma (subtribe Ligeriinae, family Gesneriaceae)", Hsu et al. present a thorough morphometric analysis of corolla shape. I very much enjoyed reading this manuscript. It integrates 2D and 3D geometric morphometrics with evolutionary theory in an unprecedented way using plant organs as an example. The morphometric analyses and the specimens are beautiful. The availability of 2D slice images, volumetric images, and surface images in the GigaScience Database repository is extremely valuable for the reproducibility of morphometric studies.
I only have minor suggestions to improve readability.
The reader is often referred to references [8] and [11] for critical methods information. This includes: image processing information (page 10, lines 181-185), landmark selection procedure (page 28, lines 469-470), and visualization (page 29, line 505). While of course all the information doesn't need to be repeated here, it should be summarized, so that the reader can understand the gist of what is being done without having to refer back to these publications.
Can a full explanation of the primary landmarks be provided? Figure 13 helps, but it would be good to see the full 25 primary landmarks. It would also help to see a visualization of all secondary landmarks too, just so the reader has a visual understanding of what the data is based upon. It might help to do this for two morphologically distinct species. For those not familiar with corolla morphology in these species, a botanical/morphological/anatomical description of what the primary landmarks correspond to would help understanding.
Scaling is used for shape but not for form. Why?
Also, I know "shape" and "form" have strict meanings in the morphometric community. But upon first usage, it might be good to just add "2D" and "3D" for clarification, not only for those not familiar with morphometrics, but also in some non-English languages the use of "shape" and "form" is confusing.
The methods come after the results section. But at least for the landmarks, an explanation up front might be helpful for readers to understand the analysis and interpret it.

Methods

Are the methods appropriate to the aims of the study, are they well described, and are necessary controls included? Choose an item.

Conclusions

Are the conclusions adequately supported by the data shown? Choose an item.

Reporting Standards

Does the manuscript adhere to the journal's guidelines on minimum standards of reporting? Choose an item.

Choose an item.

Statistics

Are you able to assess all statistics in the manuscript, including the appropriateness of statistical tests used? Choose an item.

Quality of Written English

Please indicate the quality of language in the manuscript: Choose an item.

Declaration of Competing Interests

Please complete a declaration of competing interests, considering the following questions:

- Have you in the past five years received reimbursements, fees, funding, or salary from an organisation that may in any way gain or lose financially from the publication of this manuscript, either now or in the future?
- Do you hold any stocks or shares in an organisation that may in any way gain or lose financially from the publication of this manuscript, either now or in the future?
- Do you hold or are you currently applying for any patents relating to the content of the manuscript?
- Have you received reimbursements, fees, funding, or salary from an organization that holds or has applied for patents relating to the content of the manuscript?
- Do you have any other financial competing interests?
- Do you have any non-financial competing interests in relation to this paper?

If you can answer no to all of the above, write 'I declare that I have no competing interests' below. If your reply is yes to any, please give details below.

I declare that I have no competing interests.

I agree to the open peer review policy of the journal. I understand that my name will be included on my report to the authors and, if the manuscript is accepted for publication, my named report including any attachments I upload will be posted on the website along with the authors' responses. I agree for my report to be made available under an Open Access Creative Commons CC-BY license (http://creativecommons.org/licenses/by/4.0/). I understand that any comments which I do not wish to

be included in my named report can be included as confidential comments to the editors, which will not be published.

Choose an item.

To further support our reviewers, we have joined with Publons, where you can gain additional credit to further highlight your hard work (see: https://publons.com/journal/530/gigascience). On publication of this paper, your review will be automatically added to Publons, you can then choose whether or not to claim your Publons credit. I understand this statement.

Yes Choose an item.
