# Supplementary material for: 3D revelation of phenotypic variation, evolutionary allometry, and ancestral states of corolla shape: a case study of clade Corytholoma (subtribe Ligeriinae, family Gesneriaceae)
Source: Gigascience. 2020 Jan 22;9(1):giz155. doi: 10.1093/gigascience/giz155 (PMC6974915; doi:10.1093/gigascience/giz155)
Supplement: giz155_Reviewer_2_Report_Original_Submission — Mao Li -- 8/7/2019 Reviewed [file giz155_reviewer_2_report_original_submission.pdf]

Reviewer Report

Title: 3D revelation of phenotypic variation, evolutionary allometry, and ancestral states of corolla shape: a case study of clade Corytholoma (subtribe Ligeriinae, family Gesneriaceae)

Version: Original Submission **Date:** 8/7/2019

Reviewer name: Mao Li

Reviewer Comments to Author:

In the manuscript "3D revelation of phenotypic variation, evolutionary allometry, and ancestral states of corolla shape: a case study of clade Corytholoma (subtribe Ligeriinae, family Gesneriaceae)", Hsu et al. applied 3D X-ray microcomputed tomography and landmark-based geometric morphometrics to study the corolla shape variation, evolutionary allometry, and some phylogenetic analysis. The manuscript is well written. The details are explained very clear. For their data, the analysis has been performed thoroughly.

The main concern I have is the sample size. In this work, the authors studied 15 species. However, each species only has one or two plant individuals and also were collected from different years. Although five samples from this single or two plants were scanned, some of the analysis and statements cannot convince me. In my opinion, if this is a method or breakthrough technology paper, using small sample size to test and valid the method might be fine. But this manuscript is not a method paper because all the methods are existing and commonly used. For the work focusing on the biology, I would evaluate the result which should be supported by solid analysis with enough sample size. In this work, I was wondering whether this single (or two) individual plant are representative. Assume so, then I think the analysis for the major variation using PCA and the phylogenetic analysis based on the mean shape might be fine. But the result related the within-species variation cannot convince me. Can the variance in this particular plant represent the variance for the entire species? I don't think so. The variation basically is the within-plant variation. Thus the analysis and result such as Fig. 3A, Fig. 4B, 5B are supported well. In addition, Fig 3 and Fig 4B don't have the statistic.

Another point is about the landmark based method. First, in this work, 25 primary landmarks are selected manually first. Then the rest 390 secondary landmarks are derived computationally, but still highly rely on the manual landmarks. Thus this landmark dataset may also introduce error, and can be subjective and labor intensive. 3D X-ray images also has variance for each scan. This has the similar problem with other method such as the author mentioned for other work: (line 89) "2D images are usually acquired manually, which may introduce error or artifacts…"; (line 97) " distance-based traits are typically proposed based on manual observation and can be subjective"; (line 126) " the traits were proposed statistically rather than manually". Second, the landmark based method has been widely applied. However, it has a main limitation that you have to make sure the landmarks are homologous. I was wonder whether all the corollas have five petals. If not, how the authors made sure the landmark were homologous? Because of this limitation, similar work cannot be easily expanded to a wider range of species. It would be better to discuss a bit in the discussion.

Some minor points:

a) At the beginning, I was confused about the "shape" and "form". It got clear after reading several pages. It would be nice to clarify a bit at the beginning.
b) (Line 78) Although this table is a very nice literature summary, I feel it is not directly related to this study. The authors could consider to move it to supplement.
c) (Line 141) I cannot find Appendix 1.
d) (Line 167) From this summary, it looks like the color has strong correlation with the pollination types. If the authors have the 2D images as well, additional analysis to associate color and pollination types can be done.
e) (Line 200) No indication of "the average" in the figure.
f) (Line 204) Fig 3B was not mentioned anywhere in the manuscript.
g) (Line 274) I know "shape scores" will be explained in the method section later, but it appeared too suddenly here. Please either explain a bit here or guide readers to the method section.
h) (Line 276), I know p=0.0625 is closed to 0.05, but instead of "significant", people normally say it is not statistical significant
i) (Line 305) the p value is only for the tube curvature.
j) (Line 310) Cannot see the last column of the Table 3.
k) (Line 350) How to explain the difference of pollination types at nodes 10 and 12 between shape and form?
l) In the method section, authors used different number of permutations (e.g. 10,000 times, 1000 times, 100 times) for different methods. I just wonder how authors picked these numbers.

Methods

Are the methods appropriate to the aims of the study, are they well described, and are necessary controls included? Choose an item.

Conclusions

Are the conclusions adequately supported by the data shown? Choose an item.

Reporting Standards

Does the manuscript adhere to the journal's guidelines on minimum standards of reporting? Choose an item.

Choose an item.

Statistics

Are you able to assess all statistics in the manuscript, including the appropriateness of statistical tests used? Choose an item.

Quality of Written English

Please indicate the quality of language in the manuscript: Choose an item.

Declaration of Competing Interests

Please complete a declaration of competing interests, considering the following questions:

- Have you in the past five years received reimbursements, fees, funding, or salary from an organisation that may in any way gain or lose financially from the publication of this manuscript, either now or in the future?
- Do you hold any stocks or shares in an organisation that may in any way gain or lose financially from the publication of this manuscript, either now or in the future?
- Do you hold or are you currently applying for any patents relating to the content of the manuscript?
- Have you received reimbursements, fees, funding, or salary from an organization that holds or has applied for patents relating to the content of the manuscript?
- Do you have any other financial competing interests?
- Do you have any non-financial competing interests in relation to this paper?

If you can answer no to all of the above, write 'I declare that I have no competing interests' below. If your reply is yes to any, please give details below.

I declare that I have no competing interests.

I agree to the open peer review policy of the journal. I understand that my name will be included on my report to the authors and, if the manuscript is accepted for publication, my named report including any attachments I upload will be posted on the website along with the authors' responses. I agree for my report to be made available under an Open Access Creative Commons CC-BY license (http://creativecommons.org/licenses/by/4.0/). I understand that any comments which I do not wish to be included in my named report can be included as confidential comments to the editors, which will not be published.

Choose an item.

To further support our reviewers, we have joined with Publons, where you can gain additional credit to further highlight your hard work (see: https://publons.com/journal/530/gigascience). On publication of this paper, your review will be automatically added to Publons, you can then choose whether or not to claim your Publons credit. I understand this statement.

Yes Choose an item.
