# Supplementary material for: 3D revelation of phenotypic variation, evolutionary allometry, and ancestral states of corolla shape: a case study of clade Corytholoma (subtribe Ligeriinae, family Gesneriaceae)
Source: Gigascience. 2020 Jan 22;9(1):giz155. doi: 10.1093/gigascience/giz155 (PMC6974915; doi:10.1093/gigascience/giz155)
Supplement: giz155_Reviewer_2_Report_Revision_1 — Mao Li -- 9/24/2019 Reviewed [file giz155_reviewer_2_report_revision_1.pdf]

Reviewer Report

Title: 3D revelation of phenotypic variation, evolutionary allometry, and ancestral states of corolla shape: a case study of clade Corytholoma (subtribe Ligeriinae, family Gesneriaceae)

Version: Revision 1 **Date:** 9/24/2019

Reviewer name: Mao Li

Reviewer Comments to Author:

This is the review for the first revision of "3D revelation of phenotypic variation, evolutionary allometry, and ancestral states of corlla shape: a case study of clade Corytholoma (subtribe Ligerlinae, family Gesneriaceae)".
For most of the authors' response to my comments, I am satisfied.
But I would like to arise two minor points for this revision:
1) The authors performed "Scheffe's multiple comparison tests" (line 231) for the statistics. But this test should be run only if the null hypothesis is rejected in an ANOVA test.
But to perform ANOVA, the ANOVA assumption tests have to perfomed to show those assumptions are satisfied.
Therefore, authors have to perform test to verify the ANOVA assumption first. If the assumptions are satisfied, they then perform ANOVA. After that, if they got significant F-statistic, then perform Scheffe's test.
2) "p=0" (line 335 and Table2) usually means p value is lower than the default minimum number of the program they used. I would suggest authors to find what the minimum number of their program is and write something like "p

Methods

Are the methods appropriate to the aims of the study, are they well described, and are necessary controls included? Choose an item.

Conclusions

Are the conclusions adequately supported by the data shown? Choose an item.

Reporting Standards

Does the manuscript adhere to the journal's guidelines on minimum standards of reporting? Choose an item.

Choose an item.

Statistics

Are you able to assess all statistics in the manuscript, including the appropriateness of statistical tests used? Choose an item.

Quality of Written English

Please indicate the quality of language in the manuscript: Choose an item.

Declaration of Competing Interests

Please complete a declaration of competing interests, considering the following questions:

- Have you in the past five years received reimbursements, fees, funding, or salary from an organisation that may in any way gain or lose financially from the publication of this manuscript, either now or in the future?
- Do you hold any stocks or shares in an organisation that may in any way gain or lose financially from the publication of this manuscript, either now or in the future?
- Do you hold or are you currently applying for any patents relating to the content of the manuscript?
- Have you received reimbursements, fees, funding, or salary from an organization that holds or has applied for patents relating to the content of the manuscript?
- Do you have any other financial competing interests?
- Do you have any non-financial competing interests in relation to this paper?

If you can answer no to all of the above, write 'I declare that I have no competing interests' below. If your reply is yes to any, please give details below.

I declare that I have no competing interests

I agree to the open peer review policy of the journal. I understand that my name will be included on my report to the authors and, if the manuscript is accepted for publication, my named report including any attachments I upload will be posted on the website along with the authors' responses. I agree for my report to be made available under an Open Access Creative Commons CC-BY license (http://creativecommons.org/licenses/by/4.0/). I understand that any comments which I do not wish to be included in my named report can be included as confidential comments to the editors, which will not be published.

Choose an item.

To further support our reviewers, we have joined with Publons, where you can gain additional credit to further highlight your hard work (see: https://publons.com/journal/530/gigascience). On publication of this paper, your review will be automatically added to Publons, you can then choose whether or not to claim your Publons credit. I understand this statement.

Yes Choose an item.
